# Subcortical volumetric alterations in four major psychiatric disorders: a mega-analysis study of 5604 subjects and a volumetric data-driven approach for classification

**DOI:** 10.1038/s41380-023-02141-9

**Published:** 2023-08-04

**Authors:** Naohiro Okada, Masaki Fukunaga, Kenichiro Miura, Kiyotaka Nemoto, Junya Matsumoto, Naoki Hashimoto, Masahiro Kiyota, Kentaro Morita, Daisuke Koshiyama, Kazutaka Ohi, Tsutomu Takahashi, Michihiko Koeda, Hidenaga Yamamori, Michiko Fujimoto, Yuka Yasuda, Naomi Hasegawa, Hisashi Narita, Satoshi Yokoyama, Ryo Mishima, Takahiko Kawashima, Yuko Kobayashi, Daiki Sasabayashi, Kenichiro Harada, Maeri Yamamoto, Yoji Hirano, Takashi Itahashi, Masahito Nakataki, Ryu-ichiro Hashimoto, Khin K. Tha, Shinsuke Koike, Toshio Matsubara, Go Okada, Theo G. M. van Erp, Neda Jahanshad, Reiji Yoshimura, Osamu Abe, Toshiaki Onitsuka, Yoshiyuki Watanabe, Koji Matsuo, Hidenori Yamasue, Yasumasa Okamoto, Michio Suzuki, Jessica A. Turner, Paul M. Thompson, Norio Ozaki, Kiyoto Kasai, Ryota Hashimoto

**Affiliations:** 1 Department of Neuropsychiatry, Graduate School of Medicine, The University of Tokyo, Tokyo, Japan; 2 The International Research Center for Neurointelligence (WPI-IRCN), The University of Tokyo Institutes for Advanced Study (UTIAS), Tokyo, Japan; 3 Division of Cerebral Integration, National Institute for Physiological Sciences, Aichi, Japan; 4 Department of Pathology of Mental Diseases, National Institute of Mental Health, National Center of Neurology and Psychiatry, Tokyo, Japan; 5 Department of Psychiatry, Institute of Medicine, University of Tsukuba, Ibaraki, Japan; 6 Department of Psychiatry, Hokkaido University Graduate School of Medicine, Hokkaido, Japan; 7 Department of Rehabilitation, University of Tokyo Hospital, Tokyo, Japan; 8 Department of Psychiatry, Gifu University Graduate School of Medicine, Gifu, Japan; 9 Department of General Internal Medicine, Kanazawa Medical University, Ishikawa, Japan; 10 Department of Neuropsychiatry, University of Toyama Graduate School of Medicine and Pharmaceutical Sciences, Toyama, Japan; 11 Research Center for Idling Brain Science, University of Toyama, Toyama, Japan; 12 Department of Neuropsychiatry, Graduate School of Medicine, Nippon Medical School, Tokyo, Japan; 13 Department of Psychiatry, Graduate School of Medicine, Osaka University, Osaka, Japan; 14 Japan Community Health Care Organization Osaka Hospital, Osaka, Japan; 15 Life Grow Brilliant Mental Clinic, Medical Corporation Foster, Osaka, Japan; 16 Department of Psychiatry and Neuroscience, Hiroshima University, Hiroshima, Japan; 17 Department of Psychiatry, Graduate School of Medicine, Kyoto University, Kyoto, Japan; 18 Division of Neuropsychiatry, Department of Neuroscience, Yamaguchi University Graduate School of Medicine, Yamaguchi, Japan; 19 Department of Psychiatry, Graduate School of Medicine, Nagoya University, Aichi, Japan; 20 Department of Neuropsychiatry, Graduate School of Medical Sciences, Kyushu University, Fukuoka, Japan; 21 Department of Psychiatry, Division of Clinical Neuroscience, Faculty of Medicine, University of Miyazaki, Miyazaki, Japan; 22 Medical Institute of Developmental Disabilities Research, Showa University, Tokyo, Japan; 23 Department of Psychiatry, Graduate School of Biomedical Sciences, Tokushima University, Tokushima, Japan; 24 Department of Language Sciences, Graduate School of Humanities, Tokyo Metropolitan University, Tokyo, Japan; 25 Department of Diagnostic Imaging, Hokkaido University Faculty of Medicine, Hokkaido, Japan; 26 Global Center for Biomedical Science and Engineering, Hokkaido University Faculty of Medicine, Hokkaido, Japan; 27 University of Tokyo Institute for Diversity & Adaptation of Human Mind (UTIDAHM), Tokyo, Japan; 28 Center for Evolutionary Cognitive Sciences, Graduate School of Arts and Sciences, The University of Tokyo, Tokyo, Japan; 29 Clinical Translational Neuroscience Laboratory, Department of Psychiatry and Human Behavior, University of California Irvine, Irvine, CA USA; 30 Center for the Neurobiology of Learning and Memory, University of California Irvine, Irvine, CA USA; 31 Imaging Genetics Center, Stevens Neuroimaging and Informatics Institute, Keck School of Medicine of USC, Los Angeles, CA USA; 32 Department of Psychiatry, University of Occupational and Environmental Health, Fukuoka, Japan; 33 Department of Radiology, Graduate School of Medicine, The University of Tokyo, Tokyo, Japan; 34 National Hospital Organization Sakakibara Hospital, Mie, Japan; 35 Department of Radiology, Shiga University of Medical Science, Shiga, Japan; 36 Department of Psychiatry, Faculty of Medicine, Saitama Medical University, Saitama, Japan; 37 Department of Psychiatry, Hamamatsu University School of Medicine, Shizuoka, Japan; 38 Department of Psychiatry and Behavioral Health, Wexner Medical Center, The Ohio State University, Columbus, OH USA; 39 Pathophysiology of Mental Disorders, Graduate School of Medicine, Nagoya University, Aichi, Japan

**Keywords:** Neuroscience, Psychiatric disorders

## Abstract

Differential diagnosis is sometimes difficult in practical psychiatric settings, in terms of using the current diagnostic system based on presenting symptoms and signs. The creation of a novel diagnostic system using objective biomarkers is expected to take place. Neuroimaging studies and others reported that subcortical brain structures are the hubs for various psycho-behavioral functions, while there are so far no neuroimaging data-driven clinical criteria overcoming limitations of the current diagnostic system, which would reflect cognitive/social functioning. Prior to the main analysis, we conducted a large-scale multisite study of subcortical volumetric and lateralization alterations in schizophrenia, bipolar disorder, major depressive disorder, and autism spectrum disorder using T1-weighted images of 5604 subjects (3078 controls and 2526 patients). We demonstrated larger lateral ventricles volume in schizophrenia, bipolar disorder, and major depressive disorder, smaller hippocampus volume in schizophrenia and bipolar disorder, and schizophrenia-specific smaller amygdala, thalamus, and accumbens volumes and larger caudate, putamen, and pallidum volumes. In addition, we observed a leftward alteration of lateralization for pallidum volume specifically in schizophrenia. Moreover, as our main objective, we clustered the 5,604 subjects based on subcortical volumes, and explored whether data-driven clustering results can explain cognitive/social functioning in the subcohorts. We showed a four-biotype classification, namely extremely (Brain Biotype [BB] 1) and moderately smaller limbic regions (BB2), larger basal ganglia (BB3), and normal volumes (BB4), being associated with cognitive/social functioning. Specifically, BB1 and BB2–3 were associated with severe and mild cognitive/social impairment, respectively, while BB4 was characterized by normal cognitive/social functioning. Our results may lead to the future creation of novel biological data-driven psychiatric diagnostic criteria, which may be expected to be useful for prediction or therapeutic selection.

## Introduction

Symptoms and altered behaviors in psychiatric disorders such as schizophrenia (SZ), bipolar disorder (BP), major depressive disorder (MDD), and autism spectrum disorder (ASD) are various, but most of them relate to impaired cognitive, emotional, or volitional domains, which may cause social dysfunction and suffering in daily life. Some symptoms and altered behaviors are shared across multiple disorders [1], whereas others are disease-specific. In addition, even within one diagnostic group, different patients can have different types of manifestations [2]. Accurate diagnosis by clinicians is fundamentally required because treatment strategies, including medication, differ by diagnosis. However, in terms of using the current diagnostic system, differential diagnosis is sometimes difficult in practical psychiatric settings [3, 4], which may result in incorrect treatment selection or prognosis prediction. This is at least partly because the current diagnostic systems are based on presenting symptoms and signs and it may be hard to specify fundamental underlying pathophysiological mechanisms of dysfunction [5]. In this context, the National Institute of Mental Health developed the Research Domain Criteria (RDoC) framework, demonstrating a novel approach for data integration across multiple domains of psychological function and multiple units of analysis including biological measures with cutting across traditional diagnostic categories [5, 6]. In addition, in the context of computational psychiatry, there has recently been growing attention to data-driven approach, which seeks answers to specific questions about a given set of data [7]. Application of such data-driven hypothesis-free approach to psychiatric research is expected to lead to the creation of a novel diagnostic system using objective biomarkers, which may help provide reliable predictive, prognostic, and therapeutic information for an individual subject [8].

Neural substrates of psychiatric disorders are multi-layered and complex [9], and some relate to brain structural alterations and disrupted interregional connections [10]. Subcortical structures, including the basal ganglia and limbic system regions, are structurally and functionally inter-connected with other subcortical [11] and cortical structures [12–14], serving as the hubs not only for motor control [15], attention [16], and emotion [17] but also for learning [18], memory [19], and executive functions such as working memory and inhibitory control [20]. In addition, subcortical neural substrates of RDoC constructs were recently revealed [21]. The subcortical and cortico-subcortical circuits are associated with signal pathways of neurotransmitters such as monoamines and amino acids [22–24], and the circuit stemming from subcortical regions can dynamically influence and in turn be influenced by other brain circuits [25]. The dysfunction of such subcortical circuits can be associated with various psychiatric disorders [26, 27] and symptoms [28, 29]. In addition, a recent study reported subcortical circuit disruptions related to RDoC’s domains of function across multiple psychiatric disorders [30].

Many prior structural magnetic resonance imaging (MRI) studies revealed volumetric alterations in the subcortical regions in psychiatric disorders, which are believed to contribute to characteristic symptoms. Mega-analyses, where raw data are pooled across multiple studies, have recently been performed in MRI research in psychiatry to reach robust conclusions [31–36]. Subjects with SZ have smaller-than-normal hippocampus, amygdala, thalamus, and accumbens and larger-than-normal pallidum and lateral ventricles (LVs) [31, 32], those with BP have smaller-than-normal hippocampus and thalamus and larger-than-normal LVs [34], those with MDD have smaller-than-normal hippocampus [35], and those with ASD have smaller-than-normal amygdala, accumbens, putamen, and pallidum and larger-than-normal LVs [36]. Moreover, some prior studies reported the associations between subcortical volumes and cognitive/social functioning in various psychiatric disorders [37–39]. Larger-than-normal pallidum volumes in SZ are notable and are also seen even in early-onset psychosis [40]. Related to this, we recently reported a leftward alteration of lateralization for pallidum volume in subjects with SZ [31], which is associated with dose of antipsychotics [41]. In addition, a leftward alteration of lateralization for pallidum volume is found also in subjects with early-onset psychosis (but without detailed investigation of lateralization) [40], in subjects with at-risk mental state (ARMS) [42] and even in antipsychotics-naïve adolescents with subthreshold psychotic experiences [43]. Furthermore, volumetric lateralization of pallidum and thalamus explains individual hemispheric biases in the ability to modulate posterior alpha power, which is related to cognitive control [44].

To our knowledge, there are so far no MRI data-driven clinical criteria that could be used to overcome some of the limitations of the current diagnostic system for multiple major psychiatric disorders. This may be because MRI parameters differ across scanners and centers, making it more challenging to consistently detect relatively small differences in MRI data-derived indices between psychiatric disorders and healthy control (HC) subjects. In addition, while volumetric alterations are reported in psychiatric disorders and their extent differs by diagnosis, their effect sizes are still small to moderate, making it challenging to use them in diagnostic classifiers to reliably distinguish HCs from people with psychiatric disorders. However, as mentioned above, the creation of a novel diagnostic system using objective biomarkers is expected to take place. In particular, as subcortical brain structures are the hubs for various psycho-behavioral functions, it will be important to create a diagnostic algorithm based on subcortical volumes which may be widely used in the future. To do this, it would first be beneficial to cluster subcortical volume data across multiple psychiatric disorders and to subsequently explore whether the classification driven by subcortical volumes can possibly account for diagnosis. Ideally, such a study would use a large-scale multi-site dataset, with approaches to mitigate the known differences in MRI measures across imaging protocols. Moreover, it would also be valuable to determine whether the classifications driven by subcortical volumes are associated with cognitive/social functioning, which can influence patients’ quality of life across the current diagnostic categories. Furthermore, it would also be nice to create novel functioning-associated brain biotypes.

In the current study, as our main objective, we clustered a large number of subjects with SZ, BP, MDD, and ASD as well as HCs based on standardized subcortical volumes, explored whether the data-driven clustering results can contribute to explaining not only diagnosis but also cognitive/social functioning, and sought to create novel functioning-associated brain biotypes. Prior to the main analysis, to ensure the reliability of our dataset, we performed a large-scale multisite mega-analysis of subcortical volumetric and lateralization alterations in SZ, BP, MDD, and ASD compared to HC using methods similar to those in studies from the Enhancing Neuroimaging Genetics through Meta-Analysis (ENIGMA) consortium Working Groups (WGs) and our consortium named the Cognitive Genetics Collaborative Research Organization (COCORO). Most participants in our previous study (884 subjects with SZ and 1680 HCs) [31] were included in the current study, but new subjects with SZ and HCs were also included. Thus, the sample size was increased. None of the participants overlapped between the current study and any of the above-mentioned mega-analytical studies examining subcortical volumes in SZ, BP, MDD, and ASD.

## Materials And methods

### Sample subjects and imaging

Subjects from 14 COCORO participating sites in Japan were enrolled in the current large-scale cross-disorder cohort project. This study was approved by the institutional review board of Osaka University (approval number: 706-12), the institutional review board of the National Center of Neurology and Psychiatry (approval number A2019-036), and each local institutional review board. Written informed consent was obtained from each subject before participation. Some patients with SZ and some HCs had already been analyzed in our previous work [31], while participants did not overlap between our current study and any of the ENIGMA SZ/BP/MDD/ASD studies. Subject inclusion and exclusion criteria by site are described in Supplementary Method 1. Each participating site performed MRI scanning and obtained T1-weighted images with one or more scanner(s) and imaging protocol(s). The combination of one scanner and one imaging protocol was defined as one “protocol.” In addition, at only one site (Osaka), cognitive/social functioning was evaluated using Wechsler Adult Intelligence Scale 3rd edition (WAIS-III) [45], the University of California San Diego (UCSD) Performance-Based Skills Assessment-Brief Version (UPSA-B) [46], Social Functioning Scale (SFS) [47], and working hours per week (WHW), and medication information was collected for analysis.

### Imaging processing, quality control, and protocol selection

Detailed procedures are described in Supplementary Method 2. T1-weighted imaging data were processed using FreeSurfer software version 5.3 (http://surfer.nmr.mgh.harvard.edu), as described previously [31, 32, 34–36]. After quality control and protocol selection, a total of 5604 subjects scanned with 30 protocols were analyzed in the following cross-disorder mega-analysis. Participant demographics of the overall, SZ, BP, MDD, and ASD study populations are summarized in Table 1, and Supplementary Tables 1a, 1b, 1c, and 1d, respectively. Detailed parameters for each imaging protocol are listed in Supplementary Table 2.

**Table 1:** Participant demographics of the overall study population.

Protocol	HC	Sex		Age		SZ	Sex		Age		BP	Sex		Age		MDD	Sex		Age		ASD	Sex		Age	
	N	M	F	Mean	SD	N	M	F	Mean	SD	N	M	F	Mean	SD	N	M	F	Mean	SD	N	M	F	Mean	SD
Osaka A	413	193	220	35.8	12.7	174	102	72	36.6	12.9	-	-	-	-	-	14	9	5	27.6	10.2	45	29	16	24.4	10.2
Osaka C	419	226	193	32.6	15.2	94	50	44	34.3	12.2	5	5	0	50	19.8	19	9	10	48.6	13	18	10	8	27.8	9.8
Nippon Med	194	41	153	47.9	9	213	121	92	44.1	13.6	-	-	-	-	-	-	-	-	-	-	-	-	-	-	-
Hokkaido A	35	14	21	47.8	12.9	113	43	70	34.8	12.5	78	41	37	45.5	16.3	175	85	90	47.2	17.3	-	-	-	-	-
Tokyo A	232	142	90	34.4	11.5	99	55	44	33.3	9.6	-	-	-	-	-	-	-	-	-	-	-	-	-	-	-
Osaka B	227	125	102	30.8	12.9	57	22	35	34	13.2	-	-	-	-	-	9	4	5	58.3	19.4	12	8	4	25.8	10
Kanazawa Med	114	72	42	35.3	11.5	109	41	68	40.3	12.7	34	18	16	45.6	15	43	27	16	43.2	13.8	-	-	-	-	-
Toyama A	118	63	55	25.9	6.3	114	57	57	26.4	6.2	-	-	-	-	-	-	-	-	-	-	-	-	-	-	-
Nagoya A	121	74	47	36.5	9.8	54	30	24	43.3	10	19	8	11	49	14	-	-	-	-	-	12	12	0	30.7	9.5
Kyoto B	148	90	58	36.5	11.8	43	22	21	40.4	9.2	-	-	-	-	-	-	-	-	-	-	-	-	-	-	-
Kyoto A	111	63	48	31.9	10.6	77	41	36	36.4	9.1	-	-	-	-	-	-	-	-	-	-	-	-	-	-	-
Yamaguchi B	113	46	67	44.4	19	-	-	-	-	-	15	9	6	40.9	13.6	55	24	31	51.6	12.7	-	-	-	-	-
Tokyo B	80	54	26	28.6	5.6	41	27	14	31.4	9.2	-	-	-	-	-	-	-	-	-	-	34	34	0	30.2	6.8
Kyushu A	78	36	42	33.2	11.9	41	11	30	38.2	9.6	18	7	11	48.4	14.7	9	5	4	49	11.6	-	-	-	-	-
Yamaguchi A	90	18	72	49.3	16.1	27	5	22	55.4	8.2	8	0	8	50.3	11.2	21	9	12	50.2	12.1	-	-	-	-	-
Tokyo E	41	17	24	37.3	7.7	28	19	9	30.3	10.4	23	15	8	34.4	9.5	43	24	19	37.7	11.3	6	6	0	36.8	8.4
Hiroshima D	53	28	25	56	14.2	-	-	-	-	-	26	10	16	52.7	15.7	61	23	38	49.2	14.2	-	-	-	-	-
Hiroshima A	64	29	35	34.5	12.9	-	-	-	-	-	-	-	-	-	-	71	36	35	42.5	11.5	-	-	-	-	-
Showa	68	55	13	26.9	5.9	-	-	-	-	-	-	-	-	-	-	-	-	-	-	-	66	57	9	30.1	6.5
Toyama B	56	32	24	25.7	3.3	61	31	30	27.7	9.2	-	-	-	-	-	-	-	-	-	-	-	-	-	-	-
UOEH	54	36	18	36.6	12	15	6	9	28	13.4	-	-	-	-	-	23	10	13	43.3	13.7	-	-	-	-	-
Kyushu B	27	11	16	34.6	13.8	31	15	16	35.5	11.1	9	5	4	48.8	7.9	-	-	-	-	-	-	-	-	-	-
Hiroshima B	19	8	11	42.4	9.4	-	-	-	-	-	-	-	-	-	-	47	25	22	44.5	10.5	-	-	-	-	-
Tokyo D	47	17	30	38.8	9	11	6	5	38.4	4.2	-	-	-	-	-	-	-	-	-	-	-	-	-	-	-
Tokyo C	41	25	16	28.8	7.5	12	6	6	27.4	9.4	-	-	-	-	-	-	-	-	-	-	-	-	-	-	-
Hokkaido B	21	14	7	33.2	7.7	28	11	17	37.9	9.7	-	-	-	-	-	-	-	-	-	-	-	-	-	-	-
Hiroshima C	41	11	30	42.7	11.6	-	-	-	-	-	-	-	-	-	-	8	1	7	37.5	9.4	-	-	-	-	-
Tokushima B	19	10	9	41.6	10.7	21	11	10	42.9	10.3	-	-	-	-	-	-	-	-	-	-	-	-	-	-	-
Tokushima A	21	16	5	34.3	8.4	18	9	9	34.8	9.4	-	-	-	-	-	-	-	-	-	-	-	-	-	-	-
Nagoya B	13	8	5	66.8	4.3	19	9	10	40.4	11.5	-	-	-	-	-	-	-	-	-	-	-	-	-	-	-
Total	3078	1574	1504	36	13.8	1500	750	750	36.6	12.6	235	118	117	45.8	15.2	598	291	307	45.7	14.8	193	156	37	28.5	8.7

*HC* healthy control, *SZ* schizophrenia, *BP* bipolar disorder, *MDD* major depressive disorder, *ASD* autism spectrum disorder, *M* male, *F* female, *SD* standard deviation.

### Alterations of subcortical regional volumes in major psychiatric disorders

All linear regression analyses were conducted using SPSS version 27 (IBM), and all meta-analyses were performed using the R metafor package. To define statistical significance, we used two-sided tests and set the type-I error rate (*p*-value) to 0.05. Moreover, a Bonferroni correction was applied to the statistical results to reduce type-I errors generated by multiple comparisons. First, means and standard deviations (SDs) of subcortical regional volumes and intracranial volume (ICV) were calculated for each protocol, and for each diagnostic group. Second, we examined group differences in regional volumes within each protocol. Group differences in subcortical regional volumes were investigated using a univariate linear regression analysis including sex, age and ICV as nuisance covariates. For group differences in ICV, only sex and age were included as nuisance covariates in the regression analysis. Third, each group difference was divided by their pooled SD, yielding Cohen’s *d* effect sizes. Finally, we meta-analyzed effect sizes for group differences in regional volume. An effect size and its standard error for each protocol were entered into a random-effect model meta-analysis, and an overall group difference and its standard error were obtained. Meta-analytical procedures employed in the ENIGMA SZ/BP/MDD/ASD studies and the COCORO studies were followed in this analysis.

### Altered lateralization for subcortical volumes in major psychiatric disorders

Detailed procedures are described in Supplementary Method 3. To assess laterality for each regional volume, we used a laterality index (LI), defined as the hemispheric dominance ratio [(left − right)/(left + right)] [31, 48, 49]. Group differences in LIs were analyzed in a way similar to that for subcortical volumes.

### Creation of novel functioning-associated brain biotypes through MRI data-driven clustering

Detailed procedures are described in Supplementary Method 4. An X-means non-hierarchical clustering analysis was performed for standardized subcortical volumes [50] of all the 5604 subjects using PyClustering 0.10.1.2 library and it was examined whether clustering results were associated with diagnostic groups using a chi-square test. Next, using analysis of variance (ANOVA) or multivariate analysis of variance (MANOVA), it was investigated whether clustering results were associated with cognitive functioning (full intelligence quotient [FIQ] of the WAIS-III and WAIS-III subscales including verbal comprehension [VC], perceptual organization [PO], working memory [WM], and processing speed [PS]) [45] and social functioning (UPSA-B Financial and Communication subscales [46], SFS [47], and WHW) in subjects recruited at the Osaka site. Then, because functionally impaired subjects were one of the main focuses of our research, some clusters were, if possible, combined into one functionally normal group. The functionally normal group was defined as a multiple-cluster configuration whose average was above HCs’ average – 1 SD on all the cognitive/social functioning scales [51]. Thus, some funcitionally impaired clusters and one functionally normal group were obtained, which were defined as brain biotypes. Using data collected at all sites, linear discriminant analysis with leave-one-out cross-validation was performed to discriminate brain biotypes based on *z*-score for each of the subcortical regional volumes. Finally, it was investigated whether medication doses were different among brain biotypes in subjects recruited at the Osaka site.

## Results

### Alterations of subcortical regional volumes in major psychiatric disorders

**Fig. 1:**
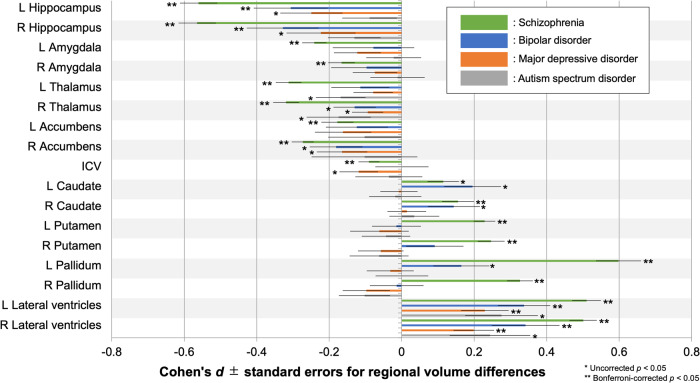
Meta-analytic overall effect sizes for subcortical regional volume differences between HCs and subjects with SZ, BP, MDD, and ASD. A positive effect size indicates that subjects with psychiatric disorders had larger volumes than HCs. *uncorrected *p* < 0.05 and **Bonferroni-corrected *p* < 0.05. ICV intracranial volume, L left, R right.

**Fig. 2:**
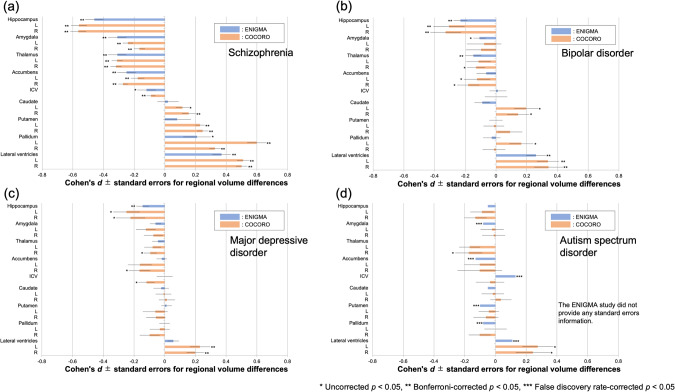
Merged results from the ENIGMA and COCORO consortiums for subcortical regional volume differences (effect sizes) between HCs and subjects with SZ, BP, MDD, and ASD. **a** Results for SZ. **b** Results for BP. **c** Results for MDD. **d** Results for ASD. * represents uncorrected *p* < 0.05, ** represents Bonferroni-corrected *p* < 0.05, and *** represents false positive rate corrected *p* < 0.05. L left, R right, ICV intracranial volume.

Participant demographics of the overall, SZ, BP, MDD, and ASD study populations are summarized in Table 1, and Supplementary Tables 1a, 1b, 1c, and 1d, respectively. Detailed parameters for each imaging protocol are listed in Supplementary Table 2. Means and SDs of regional volumes for each protocol, and for each diagnostic group, are shown in Supplementary Table 3. Group differences in regional volumes within each protocol and their corresponding Cohen’s *d* effect sizes are reported in Supplementary Tables 4 and 5, respectively. The meta-analysis of effect sizes for group differences in regional volume showed larger bilateral LV volume in SZ, BP, and MDD, smaller bilateral hippocampus volume in SZ and BP, and SZ-specific smaller bilateral amygdala, thalamus, and accumbens volumes as well as larger right caudate, bilateral putamen, and bilateral pallidum volumes (Bonferroni-corrected *p* < 0.05). In addition, the meta-analysis showed smaller right thalamus volume in BP, MDD, and ASD, smaller right accumbens volume in BP and MDD, larger left caudate volume in SZ and BP, larger right caudate and left pallidum volumes in BP, smaller bilateral hippocampus volume in MDD, and smaller left thalamus volume as well as larger bilateral LV volume in ASD (uncorrected *p* < 0.05). The effect sizes and standard errors for subcortical regional volume differences are shown in Fig. 1. In addition, the results from the ENIGMA WGs [32, 34–36] and those of the current study from COCORO are merged in Fig. 2. The *I*^2^ index, which represents the heterogeneity of effect sizes, varied between 0–73% in SZ, between 0–55% in BP, between 0–68% in MDD, and between 0–71% in ASD. Meta-analytic results for group differences in each subcortical regional volume are provided in Supplementary Figs. 1a–d and Supplementary Tables 6–9.

### Altered lateralization for subcortical volumes in major psychiatric disorders

**Fig. 3:**
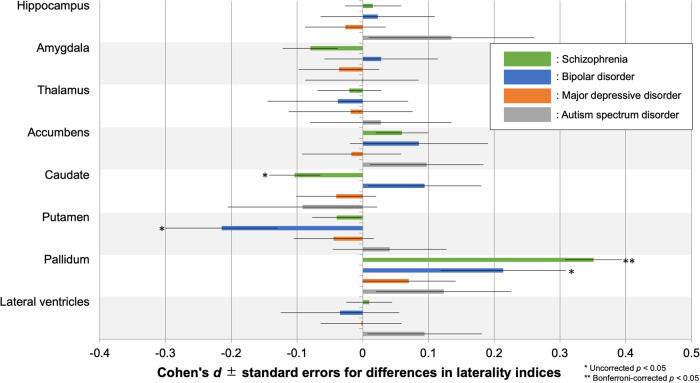
Meta-analytic overall effect sizes for differences in laterality indices for subcortical regional volume between HCs and subjects with SZ, BP, MDD, and ASD. A positive effect size indicates that subjects with psychiatric disorders had a leftward alteration of lateralization compared to HCs. *uncorrected *p* < 0.05 and **Bonferroni-corrected *p* < 0.05.

Means and SDs of LIs of regional volumes for each protocol, and for each group, are listed in Supplementary Table 10. Group differences in LIs within each protocol and their corresponding Cohen’s *d* effect sizes are reported in Supplementary Tables 11 and 12, respectively. The meta-analysis of group differences in LIs showed a lower caudate volume LI in SZ and a lower putamen volume LI in BP (uncorrected *p* < 0.05). The pallidum volume LI was higher in SZ (Bonferroni-corrected *p* < 0.05) and BP (uncorrected *p* < 0.05). The effect sizes and standard errors for differences in LIs of subcortical regional volumes are shown in Fig. 3. The *I*^2^ index varied between 0–39% in SZ, between 0–31% in BP, between 0–55% in MDD, and between 0–47% in ASD. Meta-analytic results for group differences in LI of each subcortical region are provided in Supplementary Figs. 2a–d and Supplementary Tables 13–16.

### Creation of novel functioning-associated brain biotypes through MRI data-driven clustering

**Fig. 4:**
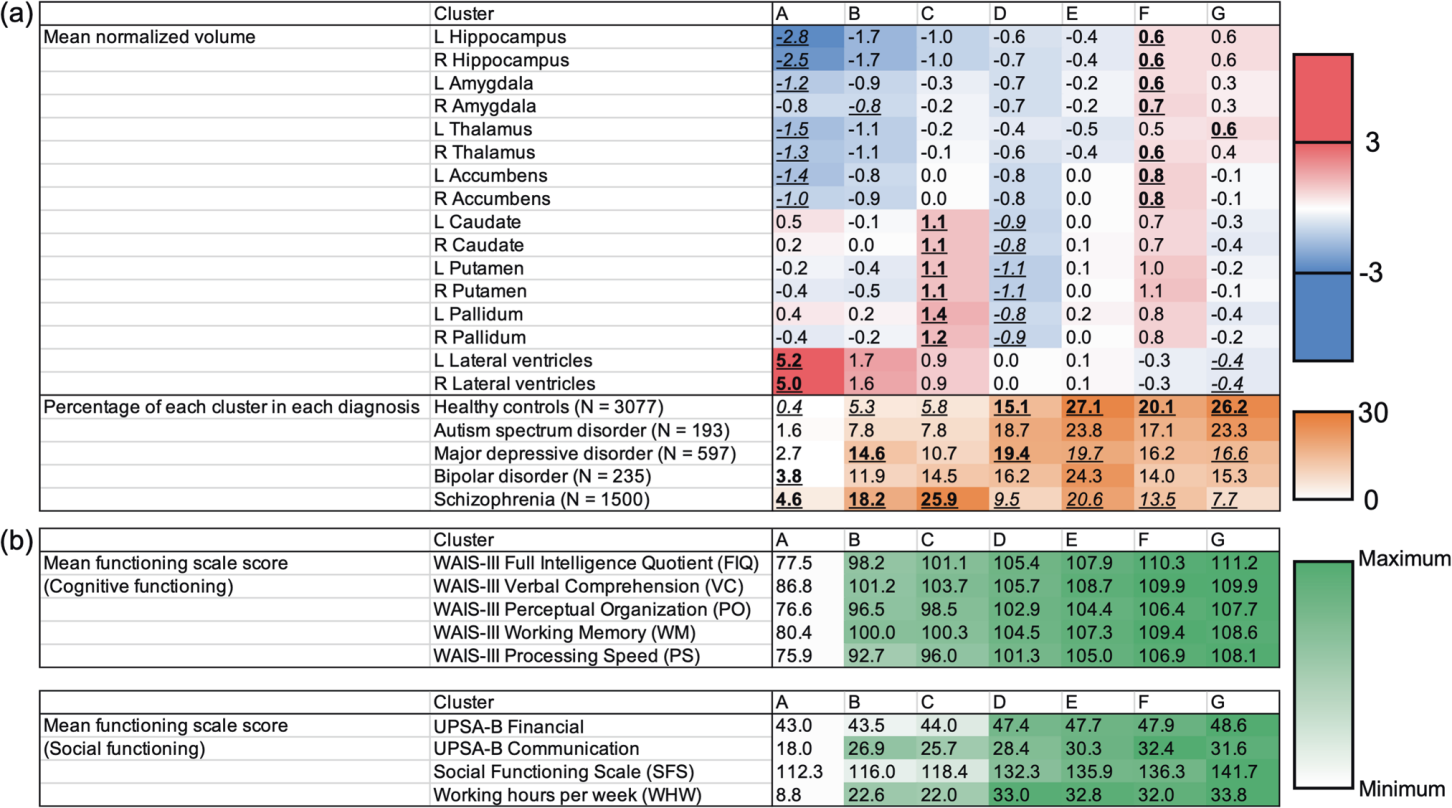
MRI data-driven clustering results and their association with diagnosis and cognitive/social functioning. **a** Mean normalized volumes of each subcortical region in each cluster are shown. An italic underlined number and a bold underlined number represent the minimum and maximum averaged normalized volume of each subcortical region across clusters, respectively. Moreover, percentages of each cluster in each diagnostic group are demonstrated. An italic underlined number and a bold underlined number represent a significantly lower and higher rate than expected, respectively. **b** Mean functioning scale scores in each cluster are displayed.

Subcortical regional volumes for each subject were standardized based on the distribution of HCs controlling for sex, age, and ICV. Then, an X-means clustering analysis was performed on *z*-scores. Supplementary Table 17 shows an association between clustering results and mean *z*-scores of each regional volume. After excluding two clusters with only one subject (Clusters H and I), Cluster A had the largest LV and the smallest hippocampus, amygdala (left), thalamus, and accumbens volumes, Cluster B had the smallest amygdala (right) volume, Cluster C had the largest caudate, putamen, and pallidum volumes, Cluster D had the smallest caudate, putamen, and pallidum volumes, Cluster F had the largest hippocampus, amygdala, thalamus (right), and accumbens volumes, and Cluster G had the smallest LV volume and the largest thalamus (left) volume (Fig. 4a). Supplementary Table 18 shows subject numbers based on diagnostic groups in each cluster. Percentages of each cluster (A–G) in each diagnostic group are shown in Fig. 4a. After excluding two clusters with only one subject (Clusters H and I), a chi-squared test found that clustering results were significantly associated with diagnostic groups (χ^2^ = 896, *p* = 1.0 × 10^−173^), and a post-hoc residual test revealed a significantly lower and higher number than expected in some cluster-diagnosis pairs. In Clusters A and B, patients with SZ and BP and those with SZ and MDD were significantly more likely to be found than expected, respectively. In Cluster C, only patients with SZ were significantly more likely to be found than expected. In Cluster D, patients with MDD and HCs were significantly more likely to be found than expected. In Clusters E, F, and G, only HCs were significantly more likely to be found than expected.

**Fig. 5:**
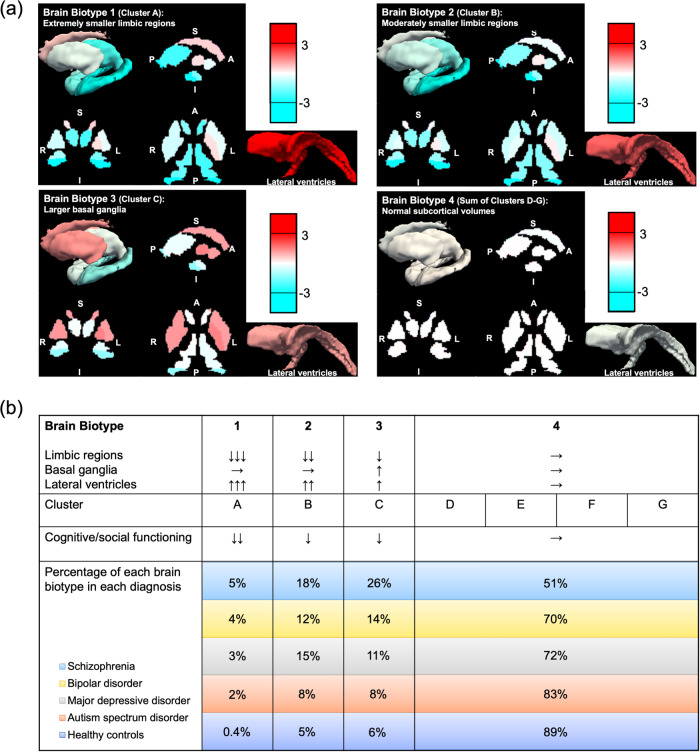
Four-biotype classification driven by subcortical regional volumes and its association with cognitive/social functioning. **a** Mean normalized volumes are illustrated with a color scale. **b** The characteristics of each brain biotype are summarized.

Next, an ANOVA revealed that, in subjects recruited at the Osaka site, clustering results had significant associations with the WAIS-III FIQ (*F* = 18.3, *p* = 1.9 × 10^−20^, *n* = 1218; Supplementary Table 19). In addition, a MANOVA for the WAIS-III subscales found their significant effects on VC (*F* = 10.2, *p* = 4.7 × 10^−11^), PO (*F* = 15.0, *p* = 1.4 × 10^−16^), WM (*F* = 11.2, *p* = 3.3 × 10^−12^), and PS (*F* = 18.1, *p* = 3.8 × 10^−20^) (*n* = 1218; Supplementary Table 19). Further, a MANOVA analysis for social functioning found that their significant effects on UPSA-B Financial (*F* = 7.0, *p* = 3.4 × 10^−7^), UPSA-B Communication (*F* = 6.2, *p* = 2.3 × 10^−6^), SFS (*F* = 8.2, *p* = 1.6 × 10^−8^) and WHW (*F* = 4.9, *p* = 7.0 × 10^−5^) (*n* = 616; Supplementary Table 20). Mean scores on these scales, for each cluster, are shown in Fig. 4b. In addition, post hoc Games-Howell tests revealed significant cognitive/social differences between clusters. The detailed results are shown in Supplementary Fig. 3. Finally, some clusters were combined into one functionally normal group, depending on cognitive/social functioning. The HCs’ means ± SDs of HCs for each scale are as follows: 112.7 ± 12.2 (WAIS-III FIQ, *n* = 937), 111.4 ± 13.1 (WAIS-III VC, *n* = 937), 108.2 ± 13.2 (WAIS-III PO, *n* = 937), 110.7 ± 15.5 (WAIS-III WM, *n* = 937), 110.1 ± 14.0 (WAIS-III PS, *n* = 937), 48.7 ± 3.3 (UPSA-B Financial, *n* = 477), 32.1 ± 8.3 (UPSA-B Communication, *n* = 477), 144.5 ± 17.4 (SFS, *n* = 477), and 36.3 ± 18.5 (WHW, *n* = 477). Thus, the functionally normal group, defined as a multiple-cluster configuration whose average was above HCs’ average – 1 SD on all the functioning scales, consisted of Clusters D, E, F, and G. A total of four brain biotypes (Brain Biotype [BB] 1 = Cluster A, BB2 = Cluster B, BB3 = Cluster C, and BB4 = Clusters D-G) were obtained. Figure 5a illustrates mean *z*-scores of each subcortical regional volume in the four brain biotypes. Characteristics of each brain biotype are summarized in Fig. 5b. Specifically, BB1 and BB2 are characterized by extremely and moderately smaller limbic volumes as well as larger LVs, resulting in severe and mild cognitive/social impairment, respectively. BB3 is characterized by larger basal ganglia, leading to mild cognitive/social impairment. BB4 is characterized by normal subcortical volumes and normal cognitive/social functioning. Percentages of each brain biotype in each diagnostic group are also shown in Fig. 5b.

Using data collected at all sites, linear discriminant analysis with leave-one-out cross-validation revealed that 89.8% of subjects were correctly classified to the original brain biotype according to *z*-score for each of the subcortical regional volumes. In addition, Kruskal–Wallis non-parametric ANOVA tests revealed that, in subjects recruited at the Osaka site (*n* = 1505), there were significant differences among brain biotypes in daily doses of antipsychotics (*H* = 192, *p* = 2.3 × 10^−41^), antidepressants (*H* = 28, *p* = 3.2 × 10^−6^), lithium carbonate (*H* = 27, *p* = 6.6 × 10^−6^), and sodium valproate (*H* = 66, *p* = 3.8 × 10^−14^). The distribution of medication doses in each brain biotype and the results of post hoc pairwise comparison tests after Kruskal–Wallis ANOVA are shown in Supplementary Fig. 4. Briefly, doses of antipsychotics, lithium carbonate, and sodium valproate in BB1, doses of antipsychotics, antidepressants, lithium carbonate, and sodium valproate in BB2, and doses of antipsychotics and lithium carbonate in BB3 were higher than those in BB4 (Bonferroni-corrected *p* < 0.05).

## Discussion

In the current large-scale cross-disorder mega-analysis study, we demonstrated larger lateral ventricles volume in SZ, BP, and MDD, smaller hippocampus volume in SZ and BP, and SZ-specific smaller amygdala, thalamus, and accumbens volumes and larger caudate, putamen, and pallidum volumes (Figs. 1, 2). In addition, we observed a leftward alteration of lateralization for pallidum volume specifically in SZ (Fig. 3). Moreover, we revealed the ability of classification driven by subcortical volume data to account for diagnosis and cognitive/social functioning, resulting in the suggestion of a new four-biotype classification (Figs. 4, 5). BB1 and BB2 are characterized by extremely and moderately smaller limbic volumes as well as larger LVs, associated with severe and mild cognitive/social impairment, respectively. BB3 is characterized by larger basal ganglia, associated with mild cognitive/social impairment. BB4 is characterized by normal subcortical volumes and normal cognitive/social functioning. Moreover, we revealed the ability of classification driven by subcortical volume data to account for diagnosis and cognitive/social functioning, resulting in the suggestion of a new four-biotype classification.

We demonstrated larger LV volume in SZ, BP, and MDD, smaller hippocampus volume in SZ and BP, and SZ-specific smaller amygdala, thalamus, and accumbens volumes and larger caudate, putamen, and pallidum volumes, using a conservative threshold of Bonferroni-corrected *p* < 0.05 (Fig. 1). In addition, we also found larger LV volume in ASD, smaller thalamus volume in BP, MDD, and ASD, smaller accumbens volume in BP, MDD, larger caudate and pallidum volumes in BP, and smaller hippocampus volume in MDD – although the results did not survive multiple corrections. Despite different numbers of participants, we were mostly successful in replicating the previous studies from ENIGMA WGs, in that group differences were similar between the current and previous studies (Fig. 2). The overall extent to which volumetric alterations occurred was the largest in SZ and this was followed by BP and MDD. ASD showed a tendency of fewer volumetric alterations compared to SZ and BP. This is in line with our previous diffusion tensor study [52]. SZ-specific smaller accumbens volume was found, which could be related to impaired dopaminergic reward and learning processes and possible subsequent onset of psychotic symptoms in SZ [53]. SZ-specific larger volumes were found in the caudate, putamen, and pallidum. Our previous study also reported larger volumes in the caudate, putamen, and pallidum in SZ [31], which the current study replicated with a larger sample. Prior mouse studies have revealed that behavioral, electrophysiological, and anatomical consequences of dopamine 2 receptor (D2R) perturbations are associated with striatal circuit function, and that D2Rs serve distinct physiological roles in different cell types and at different developmental time points, regulating motivated behaviors [54]. Larger pallidum volumes in SZ compared to controls have been reported in other large-scale studies [32, 55]. The larger pallidum volumes may be accounted for by the effects of antipsychotic medications [56] as well as by the chronicity of SZ [41]. In the future, it will be necessary to explore distinct effects of antipsychotics and chronicity on the pallidum volume using a large-scale longitudinal dataset. Hippocampus volume was smaller in SZ and BP. Inflammatory cytokine levels are negatively correlated with hippocampus volume in SZ [57] and BP [58]. This may be a candidate common mechanism for hippocampal volumetric deficiencies in these disorders.

We observed a leftward alteration of lateralization for pallidum volume in SZ (Bonferroni-corrected *p* < 0.05) and BP (uncorrected *p* < 0.05), and a rightward alteration of lateralization for caudate volume in SZ (uncorrected *p* < 0.05) and for putamen volume in BP (uncorrected *p* < 0.05) (Fig. 3). Our previous study reported a leftward alteration of lateralization for pallidum volume in SZ [31], which the current study replicated with a larger sample size. In addition, prior studies have shown a leftward alteration of lateralization for pallidum volume in subjects with early-onset psychosis [40], subjects with ARMS [42], and even adolescents with subthreshold psychotic experiences who were not on antipsychotics [43]. Thus, the leftward alteration of lateralization for pallidum volume may be a trait marker for SZ. Also, the leftward alteration of lateralization for pallidum volume was found in BP at a liberal significance threshold. While the mechanism is unknown, one possibility is that this may reflect a shared neural substrate between SZ and BP, possibly caused in part by such as common genetic factors [59, 60]. Another possibility is that antipsychotics, which are used not only for SZ but also for BP, have an influence on the leftward alteration of lateralization for pallidum volume. However, this possibility seems unlikely because, as noted, a leftward alteration of lateralization for pallidum volume was found even in adolescents with subthreshold psychotic experiences none of which are medicated with antipsychotics [43]. We also found a rightward alteration of lateralization for caudate volume in SZ and for putamen volume in BP (uncorrected *p* < 0.05). A prior mega-analysis study reported increased right, but not left, putamen volume in BP [61]. This is consistent with the current study’s findings.

We revealed that clustering-classification results driven by subcortical volumes could possibly account, to some extent, for diagnosis (Fig. 4a). The most frequent diagnostic group in Clusters E, F, and G was HC. Cluster E was characterized by the volumes close to the average of HCs. Clusters F and G were characterized by large hippocampus, amygdala, thalamus, and accumbens volumes and small LV volumes. Smaller LV volumes and larger hippocampus, amygdala, thalamus, and accumbens volumes may be an indicator for being psychiatrically healthy. The most frequent diagnostic group in Clusters A, B, and C was SZ. Cluster C was characterized by large caudate, putamen, and pallidum volumes, and Clusters A and B were characterized by large LV volume and small hippocampus, amygdala, thalamus, and accumbens volumes. This is in line with the theory of two distinct neuroanatomical subtypes of SZ, in which one subtype has larger basal ganglia volumes and the other has smaller gray matter volumes, especially in the thalamus and accumbens [62]. The most frequent diagnostic group in Cluster D was MDD and the least frequent was SZ. Cluster D was characterized by small caudate, putamen, and pallidum volumes as well as moderately small hippocampus, amygdala, thalamus, and accumbens volumes. This is in line with the theory of inflammation-related volumetric deficiencies in MDD [63]. Overall, clustering classification based on subcortical volumes may be a useful biomarker to assist diagnosis. Moreover, in the future, it may be possible to reconstruct a new diagnostic system based on subcortical volumes. In the current study, *z*-scores of regional volumes were calculated according to the distribution of HCs in each MRI protocol. Thus, one assumption of our method is that HCs’ data are available for each MRI protocol.

We also revealed that, across the current diagnostic categories, clustering-based classification results driven by subcortical volumes can possibly account for some of the variance in cognitive/social functioning in the subcohorts (Fig. 4b, Supplementary Fig. 3). This finding is in line with those of prior studies [37–39]. Clustering classification based on subcortical volumes may be a predictive biomarker for cognitive/social functioning. In addition, by combining some clusters with normal cognitive/social functioning into one group, a total of four brain biotypes (BB1, extremely smaller limbic regions; BB2, moderately smaller limbic regions; BB3, larger basal ganglia; and BB4, normal subcortical volumes) were obtained (Fig. 5). From a clinical standpoint, subjects who will be classified as belonging to BB1, BB2, or BB3 may possibly need psychiatric treatment or support from others, given their impaired functioning. Regarding this, it should be noted that a few of HCs were categorized not only in BB2 and BB3 but also in BB1. To our knowledge, the current study is the first large-scale study to report this finding. It is suggested that, although these subjects are clinically healthy now, they might be possibly vulnerable given a slight psychological burden. This point is important and may be the first step toward psychiatric prevention using a biological data-driven approach. Next, some subjects diagnosed as having a psychiatric disorder belonged to BB4. To our knowledge, the current study is the first large-scale study to report this finding. It is implied that normal subcortical volumes may be a biomarker of a better prognosis including higher treatment sensitivity and possibilities of recovery even after being diagnosed as having a psychiatric disorder. Overall, in the current study, we expanded the two-type neuroanatomical theory for SZ, developed by Chand et al. [62], to a four-type theory for multiple psychiatric disorders and clinically healthy subjects. Notably, we suggest that our current findings could lead to novel classification criteria for psychiatric disorders based on subcortical volumes. It may be possible in the future to reconstruct a new diagnostic system, based on multi-layer information including subcortical volumes and cognitive/social functioning, in accordance with the RDoC framework [5, 6]. Discriminant analysis revealed that almost 90% of subjects were correctly classified to the original brain biotype according to *z*-score for each of the subcortical regional volumes. This finding represents that any subjects with subcortical volumes standardized based on HCs’ distribution can be almost accurately classified to either brain biotype using the discrimination algorithm [50]. We thus expect that our four-biotype classification and its discrimination algorithm may have practical utility for each individual person in a clinical setting in the future. In addition, doses of antipsychotics, lithium carbonate, and sodium valproate in BB1, doses of antipsychotics, antidepressants, lithium carbonate, and sodium valproate in BB2, and doses of antipsychotics and lithium carbonate in BB3 were higher than in BB4, which was characterized by normal cognitive/social functioning (Supplementary Fig. 4). Given that most psychiatrists did their best to decide what kind of and how much medicine to prescribe for patients, this finding may possibly suggest that the prescription of psychotropic medicines which were found to be more likely prescribed in BB1, BB2, and BB3 than in BB4 might be recommended for future subjects categorized to BB1, BB2, and BB3, respectively. Future prospective investigations will be required to explore whether and how our four-biotype classification can contribute to selection of treatment including medication through focusing on outcomes of cognitive/social functioning. In addition, in the future, it will be necessary to explore how and when the anatomical differences occur and how these differences are associated with different clinical and cognitive/social outcomes – not only through multimodal human research but also translational research across species [64]. These strategies are expected to deepen our understanding of the mechanisms of volumetric alterations in patients with psychiatric disorders, that may also help reconstruct a novel diagnostic system.

The current study has some limitations. First, the current mega-analysis study is cross-sectional in nature; thus, the volumetric alterations over time in each psychiatric disorder were not examined. Some previous studies, most of which were not large-scale, reported differences in volumetric alterations between first episode and chronic stage. The collection and analysis of a large-scale longitudinal MRI data across psychiatric disorders would be of great value in the future. Second, the subject number ratio of diagnostic groups in the current study was different from that in the real world. Thus, our clustering analysis results should be carefully interpreted, especially if it is applied for practical use in the future. Moreover, a population-based cohort study may be necessary to strengthen our current results. Third, we did not directly compare any of two psychiatric disorders, because we did not have a sufficient number of MRI protocols. For example, we only had three protocols in which both subjects with BP and ASD were scanned. Fourth, by examining four different disorders together, the ability to relate imaging measures to clinical symptoms/severity was almost lost because most symptom assessment scales would be different by diagnosis. Fifth, the medication effects on subcortical brain volumes were not explored as they were beyond our scope in this study. As we have already discussed above, it will be necessary in the future to explore distinct effects of antipsychotics and chronicity on the subcortical volume using a large-scale longitudinal dataset. Sixth, almost all the subjects of this study were Japanese people. Further investigation is required to examine whether the results of this study can be shared in different races and ethnicities. Finally, no patients with anxiety-related psychiatric disorders were included in this study, reducing the generalizability of this study.

In the current large-scale cross-disorder mega-analysis study, we found shared and disease-specific alterations in subcortical volumes and their lateralization among SZ, BP, MDD, and ASD. Moreover, we revealed the ability of classification driven by subcortical volume data to account for diagnosis and cognitive/social functioning, resulting in the suggestion of a new four-biotype classification. Our results will contribute to the future creation of novel biological data-driven psychiatry diagnostic criteria, which is expected to support appropriate treatment selection.

### Supplementary information

Supplementary Information

## Data Availability

The datasets generated during and/or analyzed during the current study are not publicly available due to ethical reasons but are available from the corresponding author on reasonable request.
