# Supplementary material for: Subcortical volumetric alterations in four major psychiatric disorders: a mega-analysis study of 5604 subjects and a volumetric data-driven approach for classification
Source: Mol Psychiatry. 2023 Aug 4;28(12):5206–16. doi: 10.1038/s41380-023-02141-9 (PMC11041797; doi:10.1038/s41380-023-02141-9)
Supplement: Supplementary file 1 — Supplementary Information [file 41380_2023_2141_MOESM1_ESM.pdf]

Supplementary Information

Supplementary information for “Subcortical volumetric alterations in four major psychiatric disorders: A mega-analysis study of 5604 subjects and a volumetric data-driven approach for classification.”

Supplementary Methods

Supplementary Method 1 Subject inclusion and exclusion criteria by site

Participants recruited from the Osaka site had no biological relations, and all of them were of Japanese descent.[1-6] The subjects were excluded if they had neurological or medical conditions that could potentially affect the central nervous system, such as atypical headaches, head trauma with loss of consciousness, chronic lung disease, kidney disease, chronic hepatic disease, thyroid disease, active cancer, cerebrovascular disease, epilepsy, seizures, substance-related disorders, or mental retardation. Patients with schizophrenia (SZ), bipolar disorder (BP), major depressive disorder (MDD), and autism spectrum disorder (ASD) were recruited from the Osaka University Hospital. Each patient had been diagnosed by at least two trained psychiatrists according to the criteria from the Diagnostic and Statistical Manual of Mental Disorders (DSM), Fourth Edition (DSM-IV) based on the structured clinical interview for DSM-IV (SCID).[7] Healthy controls (HCs) were recruited through local advertisements at Osaka University. HC subjects were evaluated using the non-patient version of the SCID[8] to exclude individuals who had current or past contact with psychiatric services or who had received psychiatric medications.

Participants recruited at the Nippon Med site were excluded if they had a history of head injury, neurological illness, or a diagnosis of substance abuse or dependence. Patients with SZ were recruited from the Asai Hospital, and all patients were diagnosed by trained psychiatrists according to the DSM-IV or DSM Fifth Edition (DSM-5) criteria. HC subjects were excluded if they had a history of DSM-IV Axis I or Axis II disorders. These comparison subjects were recruited through local advertisements and from hospital staff at Asai Hospital.

Participants at the Hokkaido site were excluded if they had physical conditions that could potentially influence the central nervous system. Patients whose diagnosis fell under any of the following were recruited from the Hokkaido University Hospital; SZ, BP, and MDD. All patients were diagnosed by psychiatrists with at least six years of clinical experience according to the DSM-IV or DSM-5 criteria. HC subjects were recruited through local advertisements at Hokkaido University. All HC subjects were screened by trained psychiatrists to exclude any psychiatric disorders using the Japanese version of the Mini-International Neuropsychiatric Interview (M.I.N.I.).[9]

Inclusion and exclusion criteria for participants with SZ at the Tokyo A-C sites have been described elsewhere.[10-12] Briefly, the diagnosis of patients with SZ was determined according to the SCID-I clinical version.[7] HC subjects were screened for neuropsychiatric disorders through the SCID-I non-patient edition.[8] The exclusion criteria for these groups were current or past neurological illness, previous traumatic brain injury with any known cognitive consequences or loss of consciousness for more than five minutes, history of electroconvulsive therapy, ASD that met the DSM-IV criteria, and previous substance abuse or dependence based on clinical histories. Additional exclusion criteria for the HC subjects were a history of psychiatric disease in the subjects themselves or of Axis I disorders amongst their first-degree relatives. For participants with ASD, high-functioning

ASD males, who satisfied the eligibility criteria (firm ASD diagnosis, aged between 20 and 55 years, full-scale intelligence quotient [IQ] > 80, verbal-IQ > 85), were participated.[13-15] Briefly, participants with ASD were diagnosed according to the strict criteria included in the DSM-IV-Text Revision (DSM-IV-TR).[16] The diagnosis was further confirmed using the validated Japanese version of the Autism Diagnostic Interview-Revised (ADI-R).[17] For the participants not reaching the threshold in the ADI-R social domain, an evaluation with the Autism Diagnostic Observation Schedule[18] was employed and confirmed the diagnosis of ASD. A group of age- and IQ- matched, typically developed (TD) adult males were included as controls. All of the ASD and TD participants were interviewed by a trained psychiatrist (H.Y.) to screen for the presence of neuropsychiatric disorders using the SCID Axis I Disorder.[7] The exclusion criteria for both groups were: current or past neurological comorbidity, traumatic brain injury with any known cognitive consequences or loss of consciousness for more than five minutes, a history of electroconvulsive therapy, and substance abuse or addiction. An additional exclusion criterion for the control group was a history of psychiatric disease in the subjects themselves or a family history of an Axis I disorder in their first-degree relatives.

Participants at the Tokyo D site were excluded if they had physical conditions that could potentially influence the central nervous system. Patients with SZ were recruited from the University of Tokyo Hospital. HC subjects were sampled from the Japanese study of stratification, health, income, and neighborhood (J-SHINE) survey.[19] The diagnostic procedure was the same as that at Yaesu site described in our previous study.[20]

Participants at the Tokyo E site were excluded if they had a current or past neurological illness, history of electroconvulsive therapy, or previous substance abuse or dependence based on clinical histories. Individuals with SZ, BP, MDD, and ASD as well as HC subjects were recruited at the University of Tokyo Hospital. The diagnostic procedure was the same as that at the University of Tokyo, as described in our previous study.[21]

All subjects recruited at the Kanazawa site were of Japanese descent, and all were biologically unrelated to at least the second degree. Patients were recruited from both the outpatient and inpatient populations at Kanazawa Medical University Hospital.[22-26] Each patient with SZ, BP, or MDD had been diagnosed by at least two trained psychiatrists on the basis of unstructured clinical interviews, medical records and clinical conferences. Diagnoses were made according to criteria in the DSM-5. HC subjects were recruited through local advertisements and from among hospital staff at Kanazawa Medical University. HC subjects were evaluated using SCID non-patient version (SCID-NP) to exclude individuals who had had current or past contact with psychiatric services or who had received psychiatric medication. Subjects were excluded from analysis if they had neurological or medical conditions that could affect the central nervous system, including head trauma with loss of consciousness, chronic lung disease, chronic hepatic disease, kidney disease, active cancer, cerebrovascular disease, seizures, epilepsy, substance-related disorders or intellectual disorder.[22-26] Written informed consent was obtained from all subjects after the procedures were fully explained. This study was performed according to the World Medical

Association's Declaration of Helsinki and was approved by the research ethical committees of Kanazawa Medical University and Gifu University.

All subjects recruited from the Toyama site were Japanese and physically healthy at the time of the study. None had a lifetime history of serious head trauma, neurological illness, serious medical or surgical illness, or substance abuse disorders. Patients with SZ were recruited from the in- and outpatient clinics of the Department of Neuropsychiatry of Toyama University Hospital. The patients were diagnosed by experienced psychiatrists according to the International Classification of Diseases, Tenth Revision (ICD-10) research criteria[27] (Toyama A) and the DSM-IV-TR criteria using the SCID (Toyama B). Control subjects were recruited from members of the local community, hospital staff, and university students. Subjects at Toyama A and B were asked to complete a questionnaire consisting of 15 and 19 items, respectively, concerning their personal (13 and 17 items, respectively; including a history of obstetric complications, substantial head injury, seizures, neurological illness, impaired thyroid function, hypertension, diabetes, and substance abuse) and family (two items) histories of illness. Subjects with family history of psychiatric illness among their first-degree relatives were excluded.

Participants at the Nagoya site were excluded if they had physical conditions that might potentially influence the central nervous system. All patients (SZ, BP, and ASD) were recruited from Nagoya University Hospital and affiliated psychiatric hospitals. All patients were diagnosed by trained psychiatrists according to the DSM-IV criteria. HC subjects were recruited from the local community, hospital staff, and university students at Nagoya University and affiliated psychiatric hospitals. HC subjects were evaluated using the non-patient version of the SCID to exclude individuals who had current or past contact with psychiatric services or who had received psychiatric medications.

Patients with SZ recruited at the Kyoto site included diagnoses of SZ, schizoaffective disorder, and schizophreniform disorder. They were not comorbid with any other DSM-IV Axis I psychiatric disorders. HC subjects were recruited by local advertisements and word of mouth. They had no history of psychiatric illness. Exclusion criteria for all participants included a history of head trauma, neurological illness, mental retardation, and serious medical or surgical illness.

Participants at the Yamaguchi site were recruited from Yamaguchi University Hospital and healthy subjects were recruited from the local area. Patients met the DSM-IV-TR criteria by M.I.N.I., clinical interviews, and case conferences by senior psychiatrists. Healthy participants were recruited by advertisements and word-of-mouth in the surrounding community. Patients with current or history of substance abuse or dependence and other psychotic illnesses were excluded. HC subjects were screened during clinical interviews using the M.I.N.I. HC subjects with immediate family members having any psychiatric disorder were excluded. Based on interviews, blood tests and physical examinations, subjects with an endocrinological disease, head trauma, neurological disease, family history of any hereditary neurological disorder, or other medical conditions (e.g., hypertension, diabetes, active liver disease, kidney problems, or respiratory problems) were also excluded. This study protocol was approved by the institutional review board of Yamaguchi University

Hospital.

All subjects recruited from the Kyushu site were Japanese and physically healthy at the time of the study. The exclusion criteria were: 1) neurological illness or major head trauma; 2) electroconvulsive therapy; 3) alcohol or drug dependence; 4) alcohol or drug abuse within the past five years. HC subjects were screened using the SCID non-patient edition. No HC subjects had an Axis-I psychiatric disorder themselves or amongst their first-degree relatives. All patients were recruited from Hoaki Hospital and were diagnosed by at least two trained psychiatrists based on the SCID-DSM IV and medical records.

Participants recruited from the Hiroshima site were all right-handed, native speakers of Japanese. Patients with BP and MDD were recruited from Hiroshima University Hospital or local clinics in Hiroshima City. The patients were diagnosed by a senior psychiatrist according to the criteria from the DSM-IV or DSM-5 beforehand, and the M.I.N.I. was performed at the time of participation in the study to confirm the diagnosis. HC subjects were recruited through a newspaper advertisement. These participants were interviewed prior to enrollment by a trained psychiatrist using the M.I.N.I. Participants were excluded from all groups if they had diagnosis of SZ, alcohol and substance abuse/dependence, dementia, developmental disorders, eating disorders, personality disorder, or severe physical illness, or if they had high-level suicide risk, or if they were currently breast-feeding during pregnancy or in the postpartum period. Individuals who demonstrated a history of psychiatric illness or contact with psychiatric services were excluded from the HC subject group.

Participants recruited from the Showa site had no biological relations, and all of them were of Japanese descent. The subjects were excluded if they had neurological or medical conditions that could potentially affect the central nervous system. Individuals with ASD were recruited from the Showa University Karasuyama Hospital. The diagnostic procedure for individuals with ASD was the same as our previous studies.[28-31] Briefly, experienced psychiatrists carefully diagnosed the patients as ASD if there was a consensus between the psychiatrist and clinical psychologist who interviewed the patients independently based on the DSM-IV-TR. HC subjects were recruited by advertisements and acquaintances. None of the HC subjects reported any severe medical problem or any neurological or psychiatric history. Moreover, the M.I.N.I. was used to confirm that none of the HC subjects met the diagnostic criteria for any psychiatric disorder.

Participants at the UOEH site were excluded if they had physical conditions that might influence the central nervous system or if they had been previously diagnosed with epilepsy, seizures, substance-related disorders, or mental retardation. Patients with SZ and MDD were recruited from the in- and outpatient units at University of Occupational and Environmental Health. All patients were diagnosed by trained psychiatrists according to DSM-IV criteria based on the SCID. Controls were recruited through local advertisements and acquaintances. Healthy subjects were evaluated using the M.I.N.I. to confirm not meeting the diagnostic criteria for any psychiatric disorder.

Participants recruited from the Tokushima site had no biological relations, and all of them were of Japanese descent. The patients had been

diagnosed with SZ based on the criteria in the DSM-IV. At the time of enrollment, all patients were clinically stable, as judged by a therapeutic psychiatrist. The criteria for determining clinical stability were no schedule to change treatment contents; essentially no psychopathological changes; judgment of clinically stability by a therapeutic psychiatrist and patients themselves. Each patient's clinical stability was assessed from medical records, self-reports, and the observations of psychiatric staff and relatives. The exclusion criteria were a history or presence of any serious disorders affecting the brain or cognitive functioning, such as epilepsy, serious head injury, or brain tumor; alcohol abuse; active drug use in the past year; or pregnancy or intention to become pregnant during the study period. HC subjects were evaluated with structured clinical interviews to confirm the absence of SZ and had no history of neurological or psychiatric disorders or any first-degree relatives with psychotic episodes.

Supplementary Method 2 Imaging processing, quality control, and protocol selection

We excluded images with insufficient brain coverage (field-of-view problem), those with low signal-to-noise ratios or any artifacts (e.g., motion artifacts and magnetic susceptibility artifacts) and those with any abnormal organic findings (e.g., large cerebellar cysts and cavum septum pellucidum). Next, T1-weighted imaging data that had passed the first quality control step were processed using FreeSurfer software version 5.3 (http://surfer.nmr.mgh.harvard.edu), as described previously.[20,32-35] Through this procedure, we obtained images of subcortical segmentation and regional volumes (for the bilateral lateral ventricles [LVs], thalamus, caudate, putamen, globus pallidus, hippocampus, amygdala, accumbens and the intracranial volume [ICV]). Subsequently, two independent researchers visually inspected each segmentation image to exclude images with poor parcellation. To minimize undue effects of very small groups, protocols with less than five HCs and those with less than five subjects in each of all the four disorder groups were excluded from the following meta-analysis. Finally, a total of 5,604 subjects scanned with 30 protocols were analyzed in the following cross-disorder mega-analysis. They included 1,500 subjects with SZ, 235 with BP, 598 with MDD, 193 with ASD, and 3,078 HCs. Most of the participants in our previous study (884 subjects with SZ and 1,680 HCs)[20] were also included in the current study. Participant demographics of the overall, SZ, BP, MDD, and ASD study populations are summarized in Table 1, and Supplementary Tables 1a, 1b, 1c, and 1d, respectively. Detailed parameters for each imaging protocol are listed in Supplementary Table 2.

Supplementary Method 3 Altered lateralization for subcortical volumes in major psychiatric disorders

To assess laterality for each regional volume, we used a laterality index (LI), defined as the hemispheric dominance ratio [(left−right)/(left+right)]. This index is commonly used to evaluate brain structural asymmetry.[36,37] and was employed in our previous study.[20] LIs can range from −1 to 1, and a positive LI represents a leftward asymmetry.

First, the means and standard deviations (SDs) of LIs of subcortical regional volumes were calculated for each protocol, and for each diagnostic group. Second, group differences in LIs within each protocol were examined using a linear regression, which included sex and age as nuisance covariates. Third, each group difference was divided by their pooled SD, yielding Cohen's d effect sizes. Finally, we meta-analyzed effect sizes for group differences in LI. An effect size and its standard error for each protocol were entered into a random-effect model meta-analysis, and an overall group difference and its standard error were obtained.

Supplementary Method 4 Creation of novel functioning-associated brain biotypes through MRI data-driven clustering

The ability of classification driven by subcortical volume data to account for diagnosis, by using a large-scale multi-site dataset and minimizing scanner differences in parameters, was explored. As the volumes of left and right sides of each subcortical region were included in the classification model, laterality indices were not included in the model to avoid possible duplication of input data. First, in each of the 30 enrolled protocols, each subject's subcortical regional volumes (for the bilateral LVs, thalamus, caudate, putamen, globus pallidus, hippocampus, amygdala, and accumbens) were standardized according to the distribution of HCs, controlling for sex, age, and ICV using a linear regression model;[38] that is, sex-age-ICV adjusted volumes based on HCs' distribution in each protocol were divided by the SD of the HCs, yielding a z-score for each region of each subject. Second, an X-means non-hierarchical clustering analysis was performed for z-scores for subcortical volumes of all the 5,604 subjects using PyClustering 0.10.1.2 library. Finally, percentages of each cluster in each diagnostic group were calculated, and it was examined whether clustering results were associated with diagnostic groups using a chi-square test.

Next, it was investigated whether clustering results were associated with cognitive/social functioning in subjects recruited at the Osaka site. The effect of clustering results on the full intelligence quotient (FIQ) of the Wechsler Adult Intelligence Scale 3rd edition (WAIS-III),[39] an assessment scale for cognitive functioning, was evaluated using analysis of variance (ANOVA). In addition, their effects on the WAIS-III subscales including verbal comprehension (VC), perceptual organization (PO), working memory (WM), and processing speed (PS) as well as those on social functioning including the University of California San Diego (UCSD) Performance-Based Skills Assessment-Brief Version (UPSA-B) Financial and Communication subscales,[40] Social Functioning Scale (SFS),[41] and working hours per week (WHW) were assessed using multivariate analysis of variance (MANOVA). Then, because functionally impaired subjects were one of the main focuses of our research, some clusters were combined into one functionally normal group depending on cognitive and social functioning, if possible. The functionally normal group was defined as a multiple-cluster configuration whose average was above the HCs' average – 1SD in all the function scales above, as impaired function is usually defined as > 1 SD below the community normal mean.[42] Thus, some funcitionally impaired clusters and one functionally normal group were obtained, which were defined as brain biotypes.

Using data collected at all sites, linear discriminant analysis with leave-one-out cross-validation was performed to discriminate brain biotypes based on z-score for each of the subcortical regional volumes, which was expected to be useful for assessment of the practical utility of the discrimination algorithm. Finally, Kruskal-Wallis ANOVA and post hoc Bonferroni tests were performed to investigate whether daily doses of antipsychotics (chlorpromazine equivalent), antidepressants (imipramine equivalent), lithium carbonate, and sodium valproate were different among brain biotypes in subjects recruited at the Osaka

site, which might be informative for possible medication selection based on the brain biotype grouping.

References for Supplementary Methods

1. Hashimoto R, Ohi K, Yasuda Y, Fukumoto M, Yamamori H, Takahashi H *et al.* Variants of the RELA gene are associated with schizophrenia and their startle responses. *Neuropsychopharmacology* 2011; **36:** 1921-1931.
2. Ohi K, Hashimoto R, Yasuda Y, Nemoto K, Ohnishi T, Fukumoto M *et al.* Impact of the genome wide supported NRGN gene on anterior cingulate morphology in schizophrenia. *PLoS One* 2012; **7:** e29780.
3. Hashimoto R, Ohi K, Yasuda Y, Fukumoto M, Yamamori H, Kamino K *et al.* The KCNH2 gene is associated with neurocognition and the risk of schizophrenia. *World J Biol Psychiatry* 2013; **14:** 114-120.
4. Ohi K, Hashimoto R, Yasuda Y, Kiribayashi M, Iike N, Yoshida T *et al.* TATA box-binding protein gene is associated with risk for schizophrenia, age at onset and prefrontal function. *Genes Brain Behav* 2009; **8:** 473-480.
5. Hashimoto R, Ohi K, Yasuda Y, Fukumoto M, Iwase M, Iike N *et al.* The impact of a genome-wide supported psychosis variant in the ZNF804A gene on memory function in schizophrenia. *Am J Med Genet B Neuropsychiatr Genet* 2010; **153b:** 1459-1464.
6. Hashimoto R, Ikeda M, Yamashita F, Ohi K, Yamamori H, Yasuda Y *et al.* Common variants at 1p36 are associated with superior frontal gyrus volume. *Transl Psychiatry* 2014; **4:** e472.
7. First M, Spitzer R, Gibbon M, Williams J. *Structured Clinical Interview for DSM-IV Axis I Disorders, Clinical Version.* American Psychiatric Press: Washington DC, 1997.
8. First M, Spitzer R, Gibbon M, Williams J. *Structured Clinical Interview for DSM-IV Axis I disorders, Non-patient Edition.* New York State Psychiatric Institute: New York, 1996.
9. Otsubo T, Tanaka K, Koda R, Shinoda J, Sano N, Tanaka S *et al.* Reliability and validity of Japanese version of the Mini-International Neuropsychiatric Interview. *Psychiatry Clin Neurosci* 2005; **59:** 517-526.
10. Iwashiro N, Suga M, Takano Y, Inoue H, Natsubori T, Satomura Y *et al.* Localized gray matter volume reductions in the pars triangularis of the inferior frontal gyrus in individuals at clinical high-risk for psychosis and first episode for schizophrenia. *Schizophr Res* 2012; **137:** 124-131.
11. Natsubori T, Inoue H, Abe O, Takano Y, Iwashiro N, Aoki Y *et al.* Reduced frontal glutamate + glutamine and N-acetylaspartate levels in patients with chronic schizophrenia but not in those at clinical high risk for psychosis or

with first-episode schizophrenia. *Schizophr Bull* 2014; **40:** 1128-1139.

12. Natsubori T, Hashimoto R, Yahata N, Inoue H, Takano Y, Iwashiro N *et al.* An fMRI study of visual lexical decision in patients with schizophrenia and clinical high-risk individuals. *Schizophr Res* 2014; **157:** 218-224.
13. Watanabe T, Abe O, Kuwabara H, Yahata N, Takano Y, Iwashiro N *et al.* Mitigation of sociocommunicational deficits of autism through oxytocin-induced recovery of medial prefrontal activity: a randomized trial. *JAMA Psychiatry* 2014; **71:** 166-175.
14. Aoki Y, Yahata N, Watanabe T, Takano Y, Kawakubo Y, Kuwabara H *et al.* Oxytocin improves behavioural and neural deficits in inferring others' social emotions in autism. *Brain* 2014; **137:** 3073-3086.
15. Aoki Y, Watanabe T, Abe O, Kuwabara H, Yahata N, Takano Y *et al.* Oxytocin's neurochemical effects in the medial prefrontal cortex underlie recovery of task-specific brain activity in autism: a randomized controlled trial. *Mol Psychiatry* 2015; **20:** 447-453.
16. American Psychiatric Association. *Diagnostic and Statistical Manual of Mental Disorders, Fourth Edition: DSM-IV-TR®.* American Psychiatric Association: Washington DC, 2000.
17. Lord C, Rutter M, Le Couteur A. Autism Diagnostic Interview-Revised: a revised version of a diagnostic interview for caregivers of individuals with possible pervasive developmental disorders. *J Autism Dev Disord* 1994; **24:** 659-685.
18. Lord C, Rutter M, Goode S, Heemsbergen J, Jordan H, Mawhood L *et al.* Autism diagnostic observation schedule: a standardized observation of communicative and social behavior. *J Autism Dev Disord* 1989; **19:** 185-212.
19. Takada M, Kondo N, Hashimoto H. Japanese study on stratification, health, income, and neighborhood: study protocol and profiles of participants. *J Epidemiol* 2014; **24:** 334-344.
20. Okada N, Fukunaga M, Yamashita F, Koshiyama D, Yamamori H, Ohi K *et al.* Abnormal asymmetries in subcortical brain volume in schizophrenia. *Mol Psychiatry* 2016; **21:** 1460-1466.
21. Tanaka SC, Yamashita A, Yahata N, Itahashi T, Lisi G, Yamada T *et al.* A multi-site, multi-disorder resting-state magnetic resonance image database. *Sci Data* 2021; **8:** 227.
22. Ohi K, Shimada T, Kihara H, Yasuyama T, Sawai K, Matsuda Y *et al.*

Impact of Familial Loading on Prefrontal Activation in Major Psychiatric Disorders: A Near-Infrared Spectroscopy (NIRS) Study. *Sci Rep* 2017; **7:** 44268.

23. Ohi K, Shimada T, Nemoto K, Kataoka Y, Yasuyama T, Kimura K *et al.* Cognitive clustering in schizophrenia patients, their first-degree relatives and healthy subjects is associated with anterior cingulate cortex volume. *Neuroimage Clin* 2017; **16:** 248-256.
24. Yasuyama T, Ohi K, Shimada T, Uehara T, Kawasaki Y. Differences in social functioning among patients with major psychiatric disorders: Interpersonal communication is impaired in patients with schizophrenia and correlates with an increase in schizotypal traits. *Psychiatry Res* 2017; **249:** 30-34.
25. Ohi K, Kataoka Y, Shimada T, Kuwata A, Okubo H, Kimura K *et al.* Meta-analysis of physical activity and effects of social function and quality of life on the physical activity in patients with schizophrenia. *Eur Arch Psychiatry Clin Neurosci* 2019; **269:** 517-527.
26. Ohi K, Matsuda Y, Shimada T, Yasuyama T, Oshima K, Sawai K *et al.* Structural alterations of the superior temporal gyrus in schizophrenia: Detailed subregional differences. *Eur Psychiatry* 2016; **35:** 25-31.
27. World Health Organization. *The ICD-10 Classification of Mental and Behavioural Disorders: Diagnostic Criteria for Research*. World Health Organization: Geneva, 1993.
28. Itahashi T, Yamada T, Watanabe H, Nakamura M, Jimbo D, Shioda S *et al.* Altered network topologies and hub organization in adults with autism: a resting-state fMRI study. *PLoS One* 2014; **9:** e94115.
29. Lin IF, Kashino M, Ohta H, Yamada T, Tani M, Watanabe H *et al.* The effect of intranasal oxytocin versus placebo treatment on the autonomic responses to human sounds in autism: a single-blind, randomized, placebo-controlled, crossover design study. *Mol Autism* 2014; **5:** 20.
30. Ohta H, Yamada T, Watanabe H, Kanai C, Tanaka E, Ohno T *et al.* An fMRI study of reduced perceptual load-dependent modulation of task-irrelevant activity in adults with autism spectrum conditions. *Neuroimage* 2012; **61:** 1176-1187.
31. Watanabe H, Nakamura M, Ohno T, Itahashi T, Tanaka E, Ohta H *et al.* Altered orbitofrontal sulcogyral patterns in adult males with high-functioning autism spectrum disorders. *Soc Cogn Affect Neurosci* 2014; **9:**

520-528.
32. van Erp TG, Hibar DP, Rasmussen JM, Glahn DC, Pearlson GD, Andreassen OA *et al.* Subcortical brain volume abnormalities in 2028 individuals with schizophrenia and 2540 healthy controls via the ENIGMA consortium. *Mol Psychiatry* 2016; **21:** 547-553.
33. Hibar DP, Westlye LT, van Erp TG, Rasmussen J, Leonardo CD, Faskowitz J *et al.* Subcortical volumetric abnormalities in bipolar disorder. *Mol Psychiatry* 2016; **21:** 1710-1716.
34. Schmaal L, Veltman DJ, van Erp TG, Sämann PG, Frodl T, Jahanshad N *et al.* Subcortical brain alterations in major depressive disorder: findings from the ENIGMA Major Depressive Disorder working group. *Mol Psychiatry* 2016; **21:** 806-812.
35. van Rooij D, Anagnostou E, Arango C, Auzias G, Behrmann M, Busatto GF *et al.* Cortical and Subcortical Brain Morphometry Differences Between Patients With Autism Spectrum Disorder and Healthy Individuals Across the Lifespan: Results From the ENIGMA ASD Working Group. *Am J Psychiatry* 2018; **175:** 359-369.
36. Guadalupe T, Mathias SR, vanErp TGM, Whelan CD, Zwiers MP, Abe Y *et al.* Human subcortical brain asymmetries in 15,847 people worldwide reveal effects of age and sex. *Brain Imaging Behav* 2017; **11:** 1497-1514.
37. Kong X-Z, Mathias Samuel R, Guadalupe T, null n, Glahn David C, Franke B *et al.* Mapping cortical brain asymmetry in 17,141 healthy individuals worldwide via the ENIGMA Consortium. *Proc Natl Acad Sci USA* 2018; **115:** E5154-E5163.
38. Nemoto K, Shimokawa T, Fukunaga M, Yamashita F, Tamura M, Yamamori H *et al.* Differentiation of schizophrenia using structural MRI with consideration of scanner differences: A real-world multisite study. *Psychiatry Clin Neurosci* 2020; **74:** 56-63.
39. Wechsler D. *Wechsler Adult Intelligence Scale - 3rd Edition.* Psychological Corporation: San Antonio, 1997.
40. Mausbach BT, Harvey PD, Goldman SR, Jeste DV, Patterson TL. Development of a brief scale of everyday functioning in persons with serious mental illness. *Schizophr Bull* 2007; **33:** 1364-1372.
41. Birchwood M, Smith J, Cochrane R, Wetton S, Copestake S. The Social Functioning Scale. The development and validation of a new scale of social adjustment for use in family intervention programmes with

schizophrenic patients. *Br J Psychiatry* 1990; **157:** 853-859.

42. Saperstein AM, Fuller RL, Avila MT, Adami H, McMahon RP, Thaker GK *et al.* Spatial working memory as a cognitive endophenotype of schizophrenia: assessing risk for pathophysiological dysfunction. *Schizophr Bull* 2006; **32:** 498-506.

Supplementary Figures

Supplementary Fig. 1: Mega-analytic forest plots for group differences in each regional volume between subjects with psychiatric disorders and healthy controls.

(a) Forest plots of all 24 included protocols evaluating effect sizes for group differences in each regional volume between subjects with schizophrenia and healthy controls. **(b)** Forest plots of all 10 included protocols evaluating effect sizes for group differences in each regional volume between subjects with bipolar disorder and healthy controls. **(c)** Forest plots of all 14 included protocols evaluating effect sizes for group differences in each regional volume between subjects with major depressive disorder and healthy controls. **(d)** Forest plots of all 7 included protocols evaluating effect sizes for group differences in each regional volume between subjects with autism spectrum disorder and healthy controls.

(a)

L Hippocampus

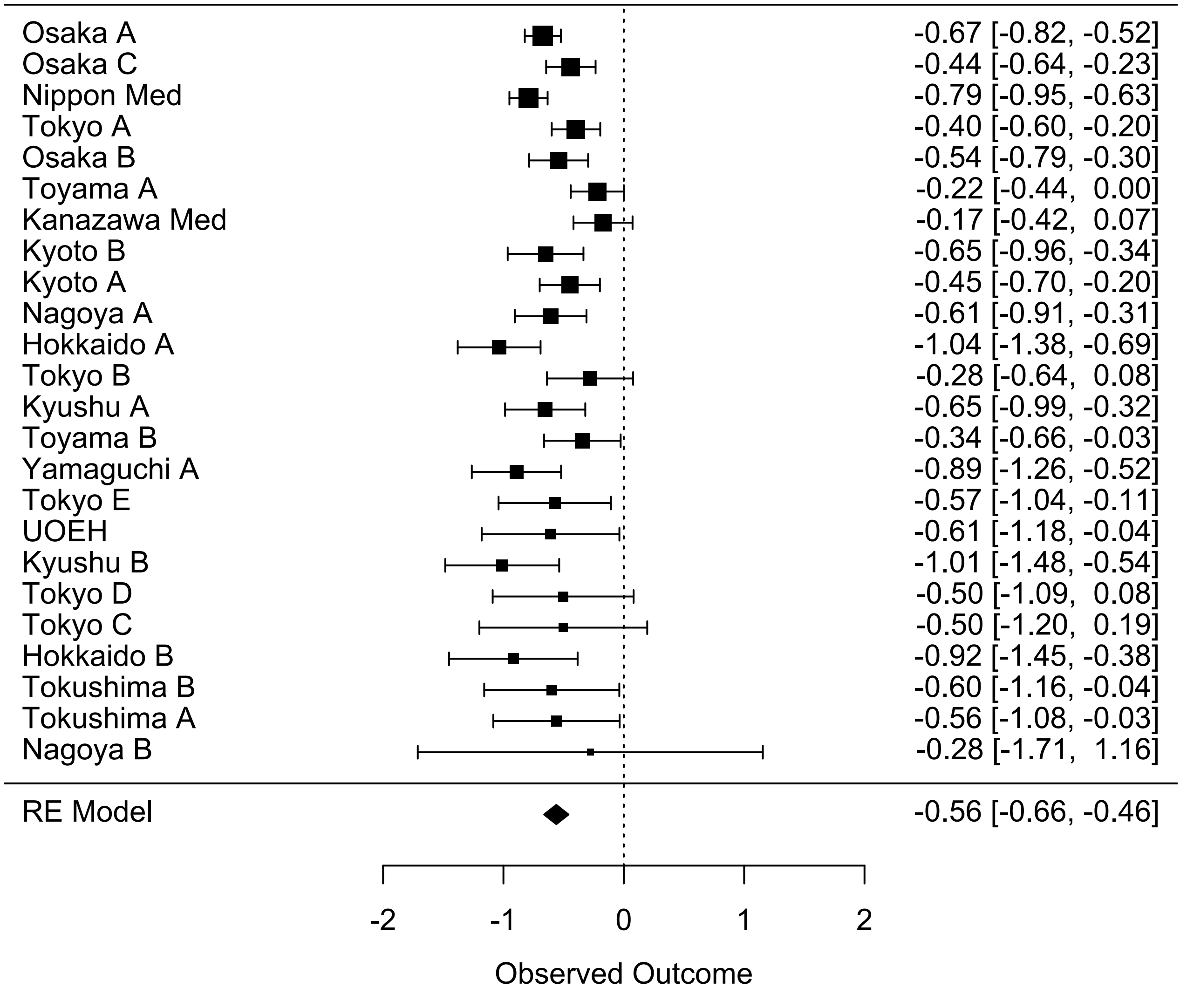

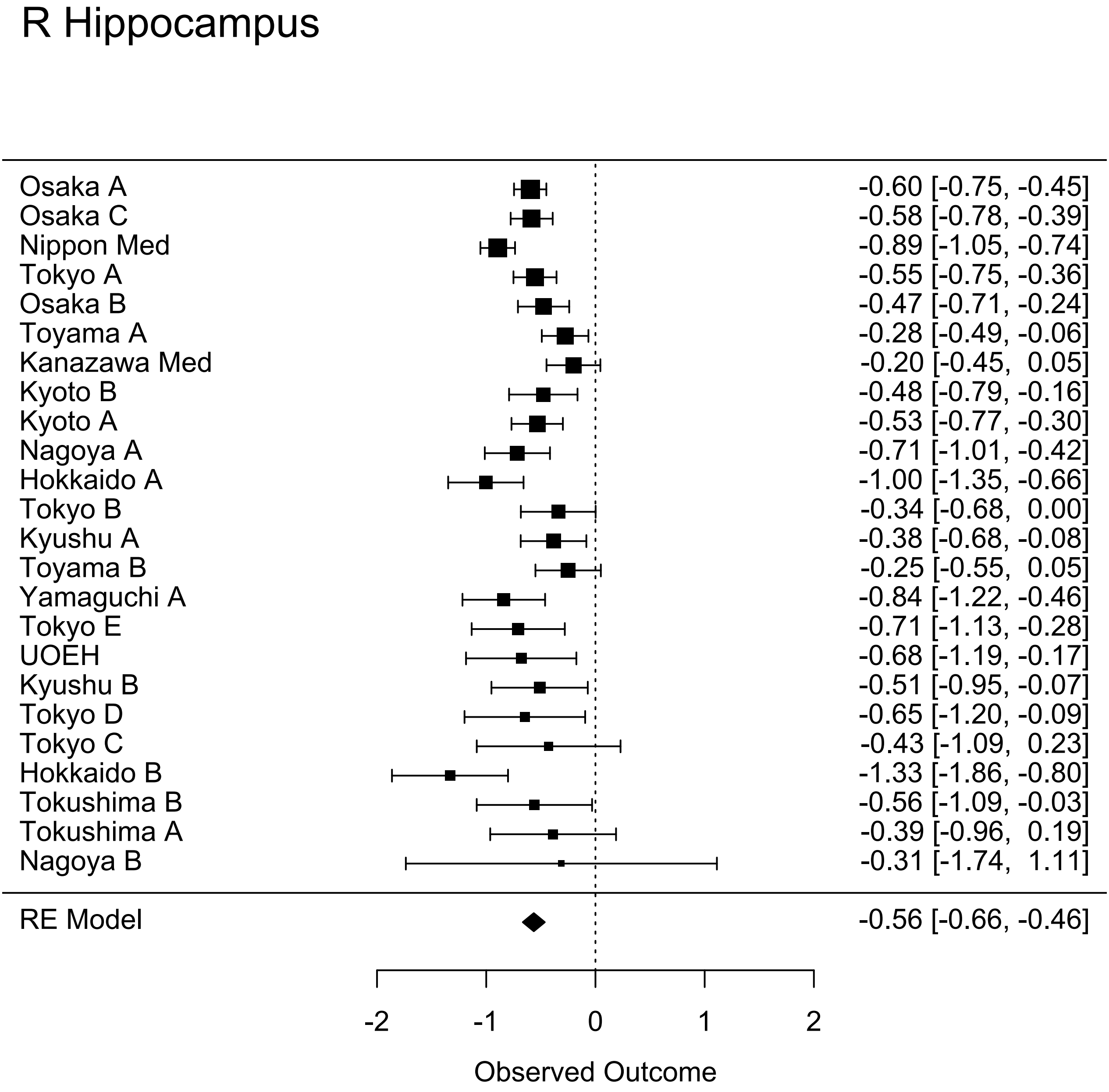
R Hippocampus
Osaka A -0.60 [-0.75, -0.45]
Osaka C -0.58 [-0.78, -0.39]
Nippon Med -0.89 [-1.05, -0.74]
Tokyo A -0.55 [-0.75, -0.36]
Osaka B -0.47 [-0.71, -0.24]
Toyama A -0.28 [-0.49, -0.06]
Kanazawa Med -0.20 [-0.45, 0.05]
Kyoto B -0.48 [-0.79, -0.16]
Kyoto A -0.53 [-0.77, -0.30]
Nagoya A -0.71 [-1.01, -0.42]
Hokkaido A -1.00 [-1.35, -0.66]
Tokyo B -0.34 [-0.68, 0.00]
Kyushu A -0.38 [-0.68, -0.08]
Toyama B -0.25 [-0.55, 0.05]
Yamaguchi A -0.84 [-1.22, -0.46]
Tokyo E -0.71 [-1.13, -0.28]
UOEH -0.68 [-1.19, -0.17]
Kyushu B -0.51 [-0.95, -0.07]
Tokyo D -0.65 [-1.20, -0.09]
Tokyo C -0.43 [-1.09, 0.23]
Hokkaido B -1.33 [-1.86, -0.80]
Tokushima B -0.56 [-1.09, -0.03]
Tokushima A -0.39 [-0.96, 0.19]
Nagoya B -0.31 [-1.74, 1.11]
RE Model -0.56 [-0.66, -0.46]
-2
-1
0
1
2
Observed Outcome

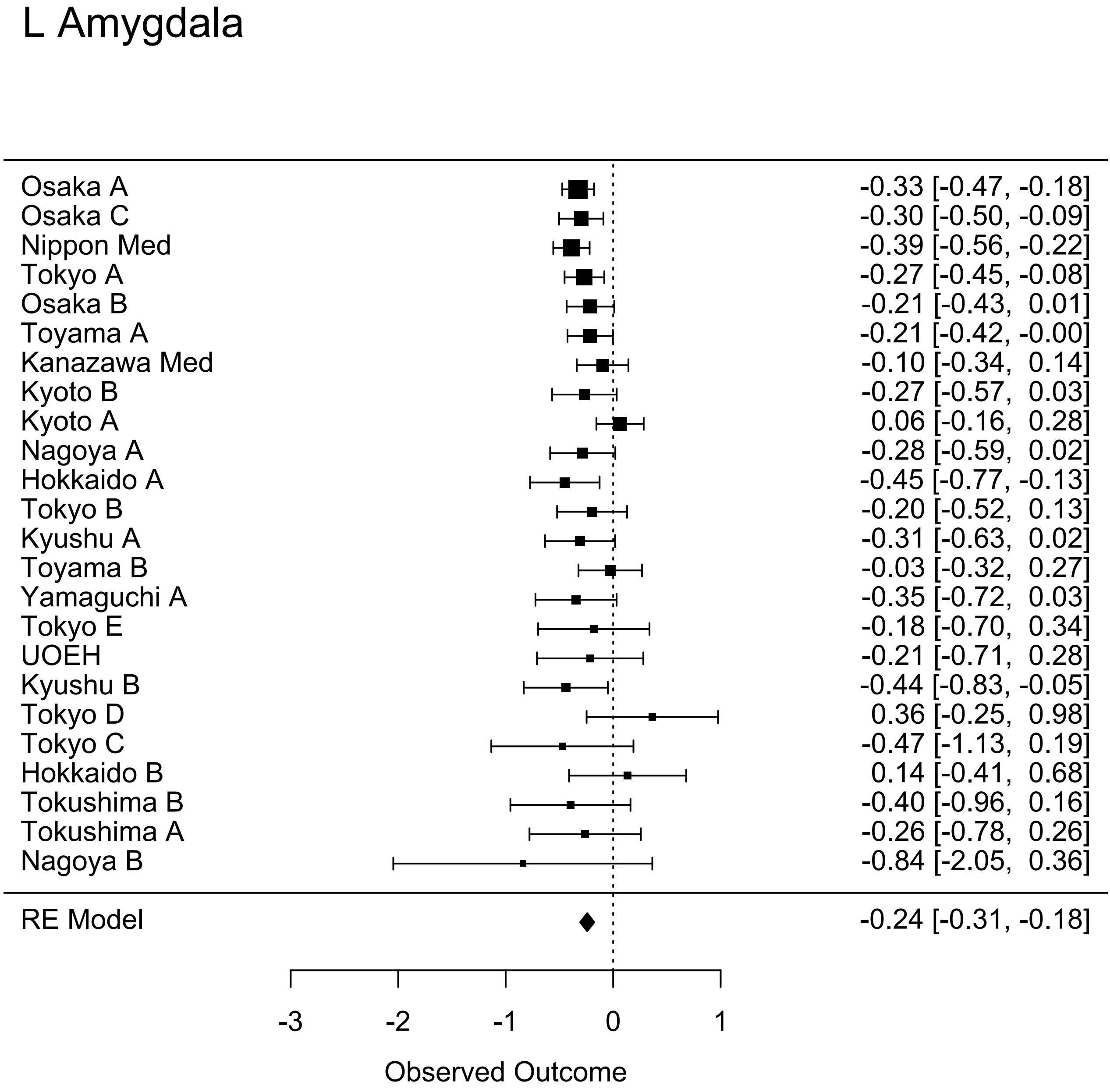
L Amygdala
Osaka A -0.33 [-0.47, -0.18]
Osaka C -0.30 [-0.50, -0.09]
Nippon Med -0.39 [-0.56, -0.22]
Tokyo A -0.27 [-0.45, -0.08]
Osaka B -0.21 [-0.43, 0.01]
Toyama A -0.21 [-0.42, -0.00]
Kanazawa Med -0.10 [-0.34, 0.14]
Kyoto B -0.27 [-0.57, 0.03]
Kyoto A 0.06 [-0.16, 0.28]
Nagoya A -0.28 [-0.59, 0.02]
Hokkaido A -0.45 [-0.77, -0.13]
Tokyo B -0.20 [-0.52, 0.13]
Kyushu A -0.31 [-0.63, 0.02]
Toyama B -0.03 [-0.32, 0.27]
Yamaguchi A -0.35 [-0.72, 0.03]
Tokyo E -0.18 [-0.70, 0.34]
UOEH -0.21 [-0.71, 0.28]
Kyushu B -0.44 [-0.83, -0.05]
Tokyo D 0.36 [-0.25, 0.98]
Tokyo C -0.47 [-1.13, 0.19]
Hokkaido B 0.14 [-0.41, 0.68]
Tokushima B -0.40 [-0.96, 0.16]
Tokushima A -0.26 [-0.78, 0.26]
Nagoya B -0.84 [-2.05, 0.36]
RE Model -0.24 [-0.31, -0.18]
-3
-2
-1
0
1
Observed Outcome

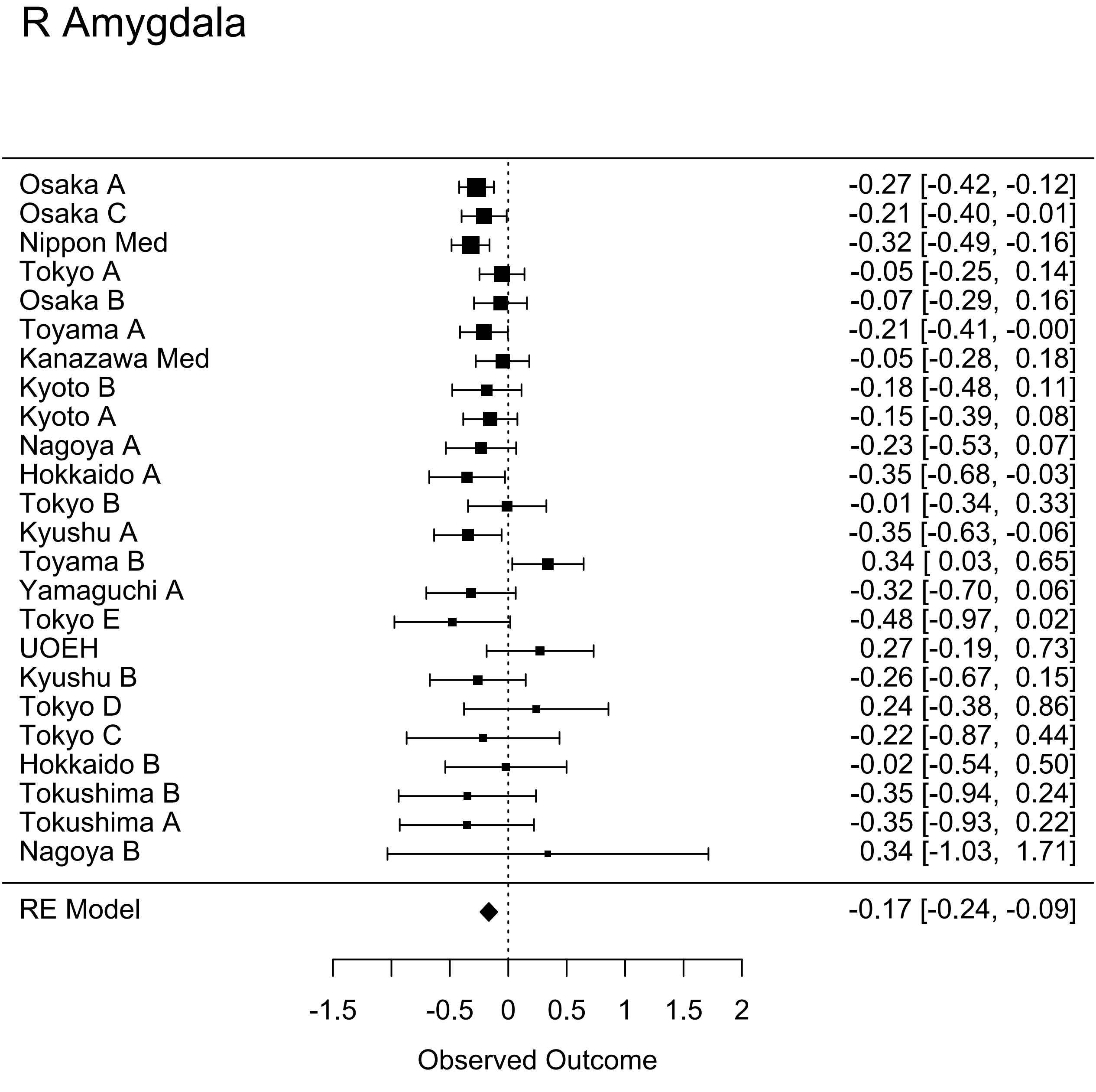
R Amygdala
Osaka A -0.27 [-0.42, -0.12]
Osaka C -0.21 [-0.40, -0.01]
Nippon Med -0.32 [-0.49, -0.16]
Tokyo A -0.05 [-0.25, 0.14]
Osaka B -0.07 [-0.29, 0.16]
Toyama A -0.21 [-0.41, -0.00]
Kanazawa Med -0.05 [-0.28, 0.18]
Kyoto B -0.18 [-0.48, 0.11]
Kyoto A -0.15 [-0.39, 0.08]
Nagoya A -0.23 [-0.53, 0.07]
Hokkaido A -0.35 [-0.68, -0.03]
Tokyo B -0.01 [-0.34, 0.33]
Kyushu A -0.35 [-0.63, -0.06]
Toyama B 0.34 [0.03, 0.65]
Yamaguchi A -0.32 [-0.70, 0.06]
Tokyo E -0.48 [-0.97, 0.02]
UOEH 0.27 [-0.19, 0.73]
Kyushu B -0.26 [-0.67, 0.15]
Tokyo D 0.24 [-0.38, 0.86]
Tokyo C -0.22 [-0.87, 0.44]
Hokkaido B -0.02 [-0.54, 0.50]
Tokushima B -0.35 [-0.94, 0.24]
Tokushima A -0.35 [-0.93, 0.22]
Nagoya B 0.34 [-1.03, 1.71]
RE Model -0.17 [-0.24, -0.09]
-1.5
-0.5
0
0.5
1
1.5
2
Observed Outcome

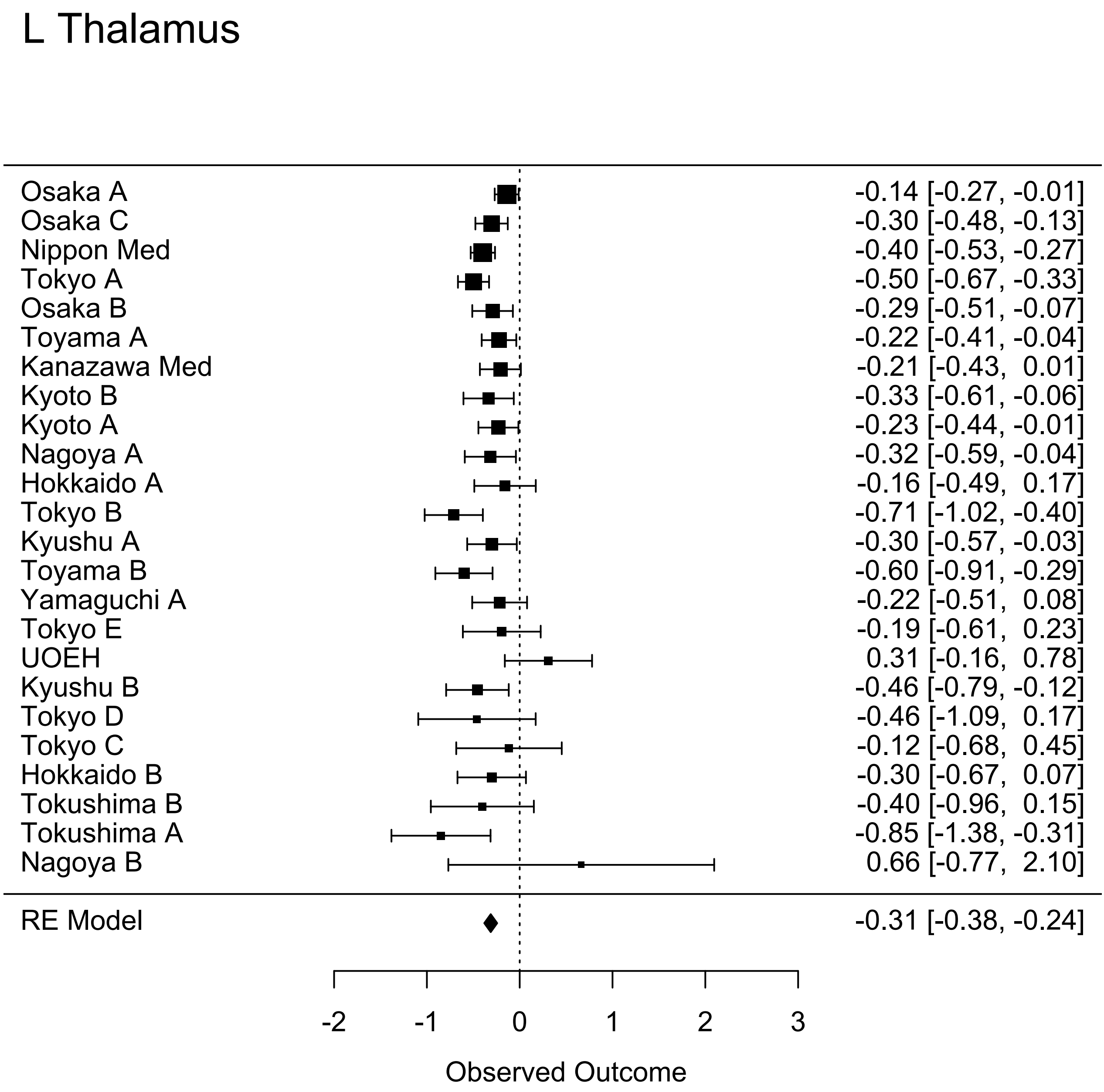
L Thalamus
Osaka A -0.14 [-0.27, -0.01]
Osaka C -0.30 [-0.48, -0.13]
Nippon Med -0.40 [-0.53, -0.27]
Tokyo A -0.50 [-0.67, -0.33]
Osaka B -0.29 [-0.51, -0.07]
Toyama A -0.22 [-0.41, -0.04]
Kanazawa Med -0.21 [-0.43, 0.01]
Kyoto B -0.33 [-0.61, -0.06]
Kyoto A -0.23 [-0.44, -0.01]
Nagoya A -0.32 [-0.59, -0.04]
Hokkaido A -0.16 [-0.49, 0.17]
Tokyo B -0.71 [-1.02, -0.40]
Kyushu A -0.30 [-0.57, -0.03]
Toyama B -0.60 [-0.91, -0.29]
Yamaguchi A -0.22 [-0.51, 0.08]
Tokyo E -0.19 [-0.61, 0.23]
UOEH 0.31 [-0.16, 0.78]
Kyushu B -0.46 [-0.79, -0.12]
Tokyo D -0.46 [-1.09, 0.17]
Tokyo C -0.12 [-0.68, 0.45]
Hokkaido B -0.30 [-0.67, 0.07]
Tokushima B -0.40 [-0.96, 0.15]
Tokushima A -0.85 [-1.38, -0.31]
Nagoya B 0.66 [-0.77, 2.10]
RE Model -0.31 [-0.38, -0.24]
-2
-1
0
1
2
3
Observed Outcome

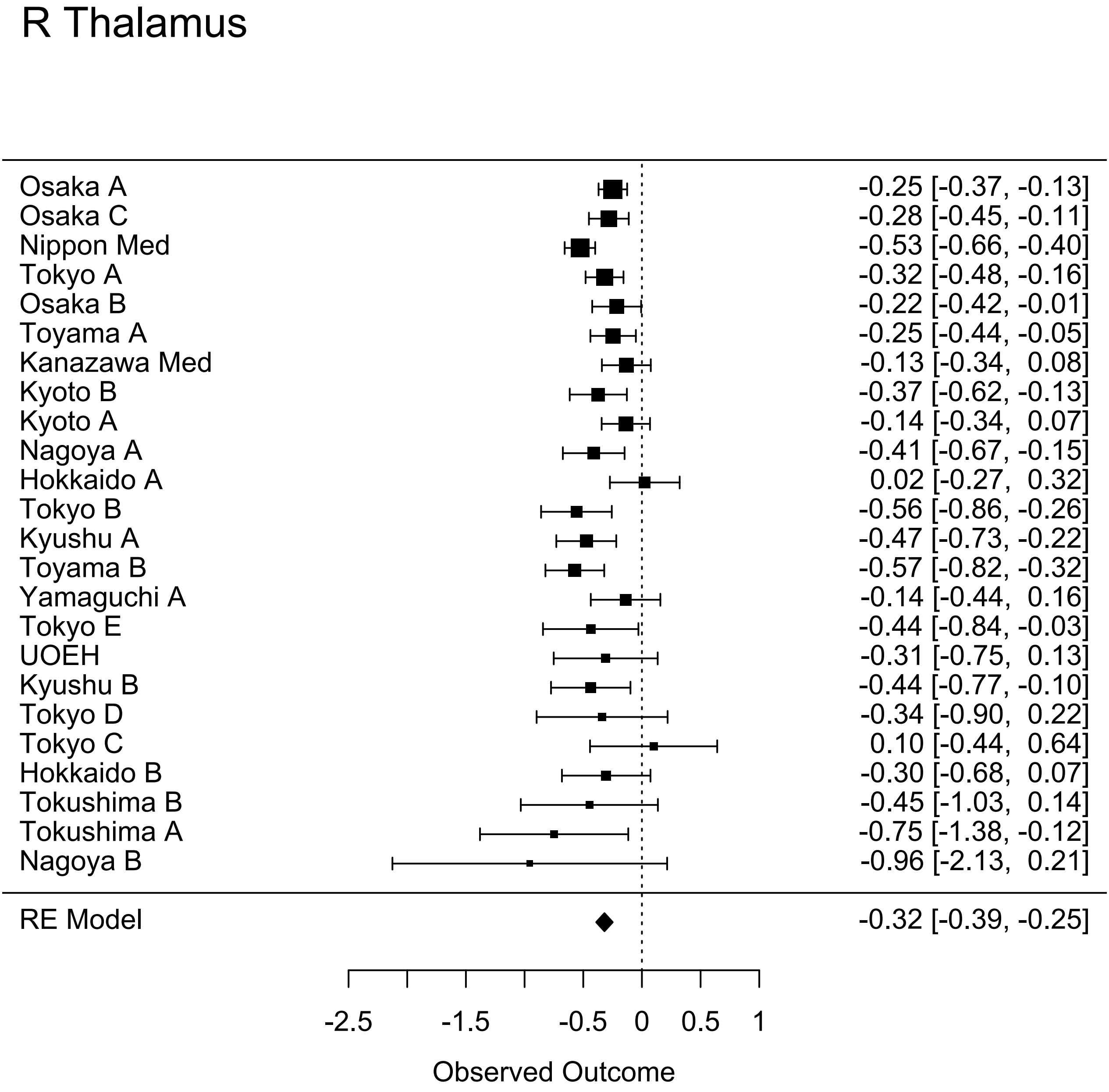
R Thalamus
Osaka A -0.25 [-0.37, -0.13]
Osaka C -0.28 [-0.45, -0.11]
Nippon Med -0.53 [-0.66, -0.40]
Tokyo A -0.32 [-0.48, -0.16]
Osaka B -0.22 [-0.42, -0.01]
Toyama A -0.25 [-0.44, -0.05]
Kanazawa Med -0.13 [-0.34, 0.08]
Kyoto B -0.37 [-0.62, -0.13]
Kyoto A -0.14 [-0.34, 0.07]
Nagoya A -0.41 [-0.67, -0.15]
Hokkaido A 0.02 [-0.27, 0.32]
Tokyo B -0.56 [-0.86, -0.26]
Kyushu A -0.47 [-0.73, -0.22]
Toyama B -0.57 [-0.82, -0.32]
Yamaguchi A -0.14 [-0.44, 0.16]
Tokyo E -0.44 [-0.84, -0.03]
UOEH -0.31 [-0.75, 0.13]
Kyushu B -0.44 [-0.77, -0.10]
Tokyo D -0.34 [-0.90, 0.22]
Tokyo C 0.10 [-0.44, 0.64]
Hokkaido B -0.30 [-0.68, 0.07]
Tokushima B -0.45 [-1.03, 0.14]
Tokushima A -0.75 [-1.38, -0.12]
Nagoya B -0.96 [-2.13, 0.21]
RE Model -0.32 [-0.39, -0.25]
-2.5
-1.5
-0.5
0
0.5
1
Observed Outcome

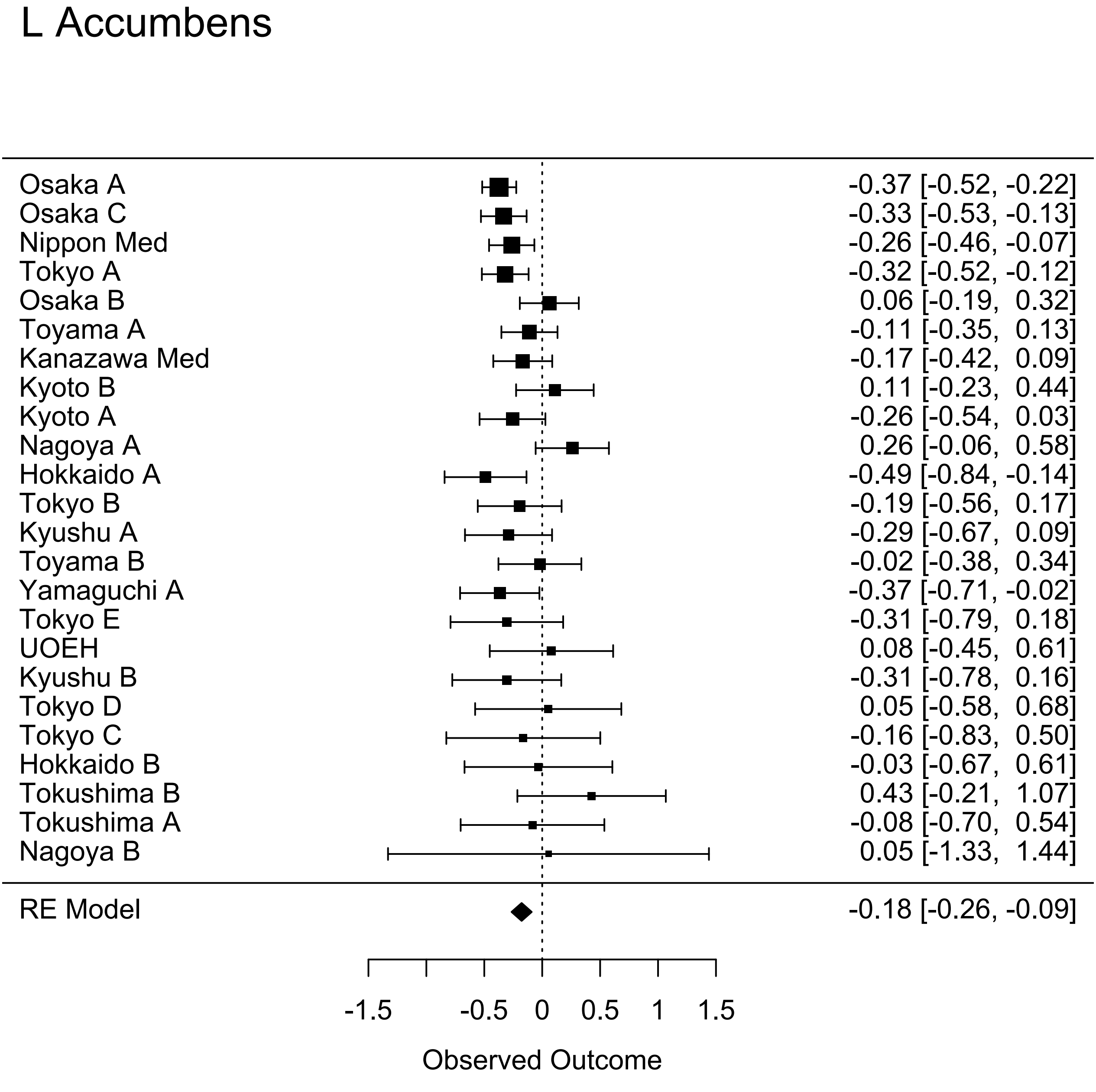
L Accumbens
Osaka A -0.37 [-0.52, -0.22]
Osaka C -0.33 [-0.53, -0.13]
Nippon Med -0.26 [-0.46, -0.07]
Tokyo A -0.32 [-0.52, -0.12]
Osaka B 0.06 [-0.19, 0.32]
Toyama A -0.11 [-0.35, 0.13]
Kanazawa Med -0.17 [-0.42, 0.09]
Kyoto B 0.11 [-0.23, 0.44]
Kyoto A -0.26 [-0.54, 0.03]
Nagoya A 0.26 [-0.06, 0.58]
Hokkaido A -0.49 [-0.84, -0.14]
Tokyo B -0.19 [-0.56, 0.17]
Kyushu A -0.29 [-0.67, 0.09]
Toyama B -0.02 [-0.38, 0.34]
Yamaguchi A -0.37 [-0.71, -0.02]
Tokyo E -0.31 [-0.79, 0.18]
UOEH 0.08 [-0.45, 0.61]
Kyushu B -0.31 [-0.78, 0.16]
Tokyo D 0.05 [-0.58, 0.68]
Tokyo C -0.16 [-0.83, 0.50]
Hokkaido B -0.03 [-0.67, 0.61]
Tokushima B 0.43 [-0.21, 1.07]
Tokushima A -0.08 [-0.70, 0.54]
Nagoya B 0.05 [-1.33, 1.44]
RE Model -0.18 [-0.26, -0.09]
-1.5
-0.5
0
0.5
1
1.5
Observed Outcome

R Accumbens

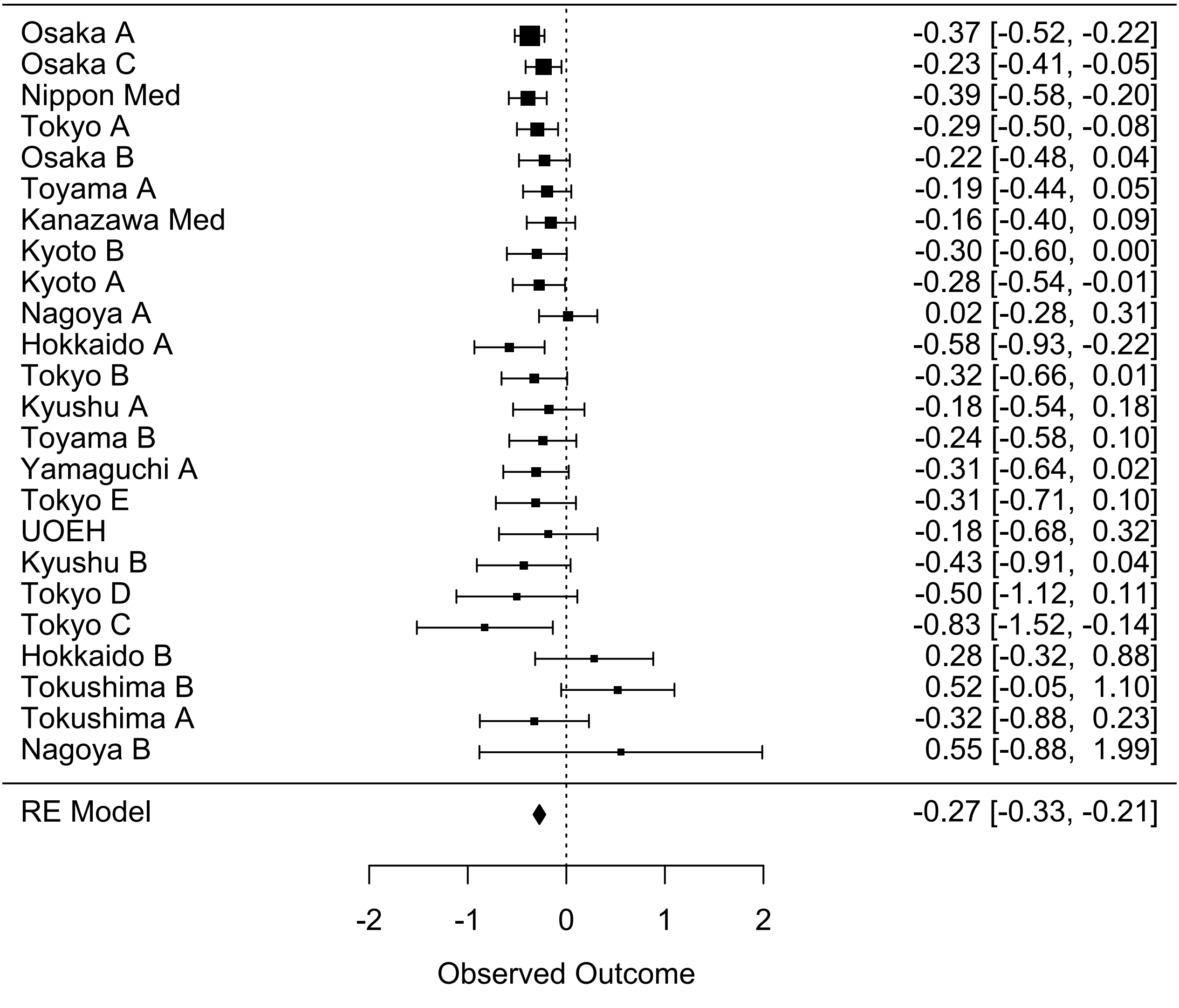

Osaka A -0.37 [-0.52, -0.22]
Osaka C -0.23 [-0.41, -0.05]
Nippon Med -0.39 [-0.58, -0.20]
Tokyo A -0.29 [-0.50, -0.08]
Osaka B -0.22 [-0.48, 0.04]
Toyama A -0.19 [-0.44, 0.05]
Kanazawa Med -0.16 [-0.40, 0.09]
Kyoto B -0.30 [-0.60, 0.00]
Kyoto A -0.28 [-0.54, -0.01]
Nagoya A 0.02 [-0.28, 0.31]
Hokkaido A -0.58 [-0.93, -0.22]
Tokyo B -0.32 [-0.66, 0.01]
Kyushu A -0.18 [-0.54, 0.18]
Toyama B -0.24 [-0.58, 0.10]
Yamaguchi A -0.31 [-0.64, 0.02]
Tokyo E -0.31 [-0.71, 0.10]
UOEH -0.18 [-0.68, 0.32]
Kyushu B -0.43 [-0.91, 0.04]
Tokyo D -0.50 [-1.12, 0.11]
Tokyo C -0.83 [-1.52, -0.14]
Hokkaido B 0.28 [-0.32, 0.88]
Tokushima B 0.52 [-0.05, 1.10]
Tokushima A -0.32 [-0.88, 0.23]
Nagoya B 0.55 [-0.88, 1.99]
RE Model -0.27 [-0.33, -0.21]
-2
-1
0
1
2
Observed Outcome

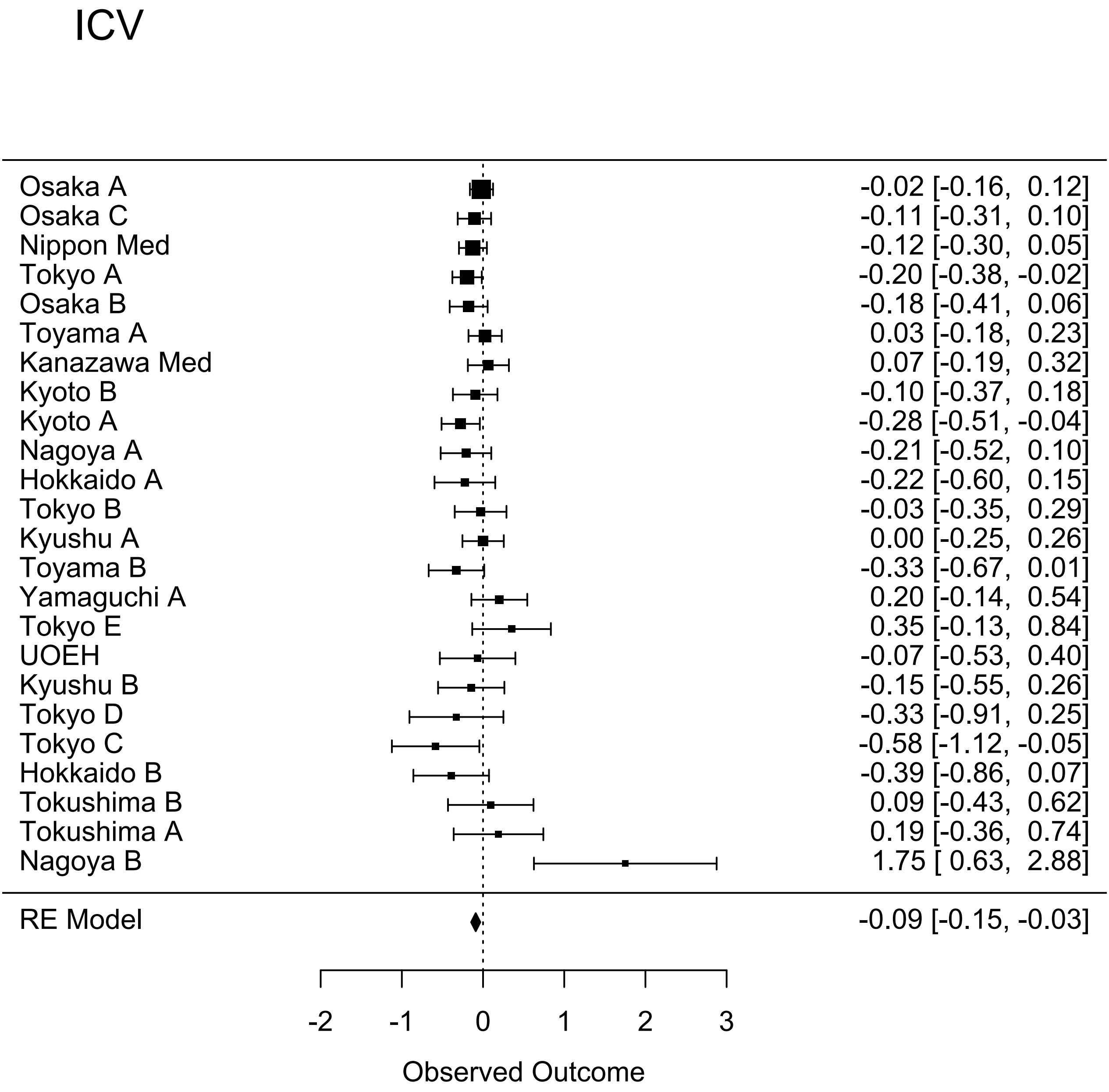
ICV
Osaka A -0.02 [-0.16, 0.12]
Osaka C -0.11 [-0.31, 0.10]
Nippon Med -0.12 [-0.30, 0.05]
Tokyo A -0.20 [-0.38, -0.02]
Osaka B -0.18 [-0.41, 0.06]
Toyama A 0.03 [-0.18, 0.23]
Kanazawa Med 0.07 [-0.19, 0.32]
Kyoto B -0.10 [-0.37, 0.18]
Kyoto A -0.28 [-0.51, -0.04]
Nagoya A -0.21 [-0.52, 0.10]
Hokkaido A -0.22 [-0.60, 0.15]
Tokyo B -0.03 [-0.35, 0.29]
Kyushu A 0.00 [-0.25, 0.26]
Toyama B -0.33 [-0.67, 0.01]
Yamaguchi A 0.20 [-0.14, 0.54]
Tokyo E 0.35 [-0.13, 0.84]
UOEH -0.07 [-0.53, 0.40]
Kyushu B -0.15 [-0.55, 0.26]
Tokyo D -0.33 [-0.91, 0.25]
Tokyo C -0.58 [-1.12, -0.05]
Hokkaido B -0.39 [-0.86, 0.07]
Tokushima B 0.09 [-0.43, 0.62]
Tokushima A 0.19 [-0.36, 0.74]
Nagoya B 1.75 [0.63, 2.88]
RE Model -0.09 [-0.15, -0.03]
-2
-1
0
1
2
3
Observed Outcome

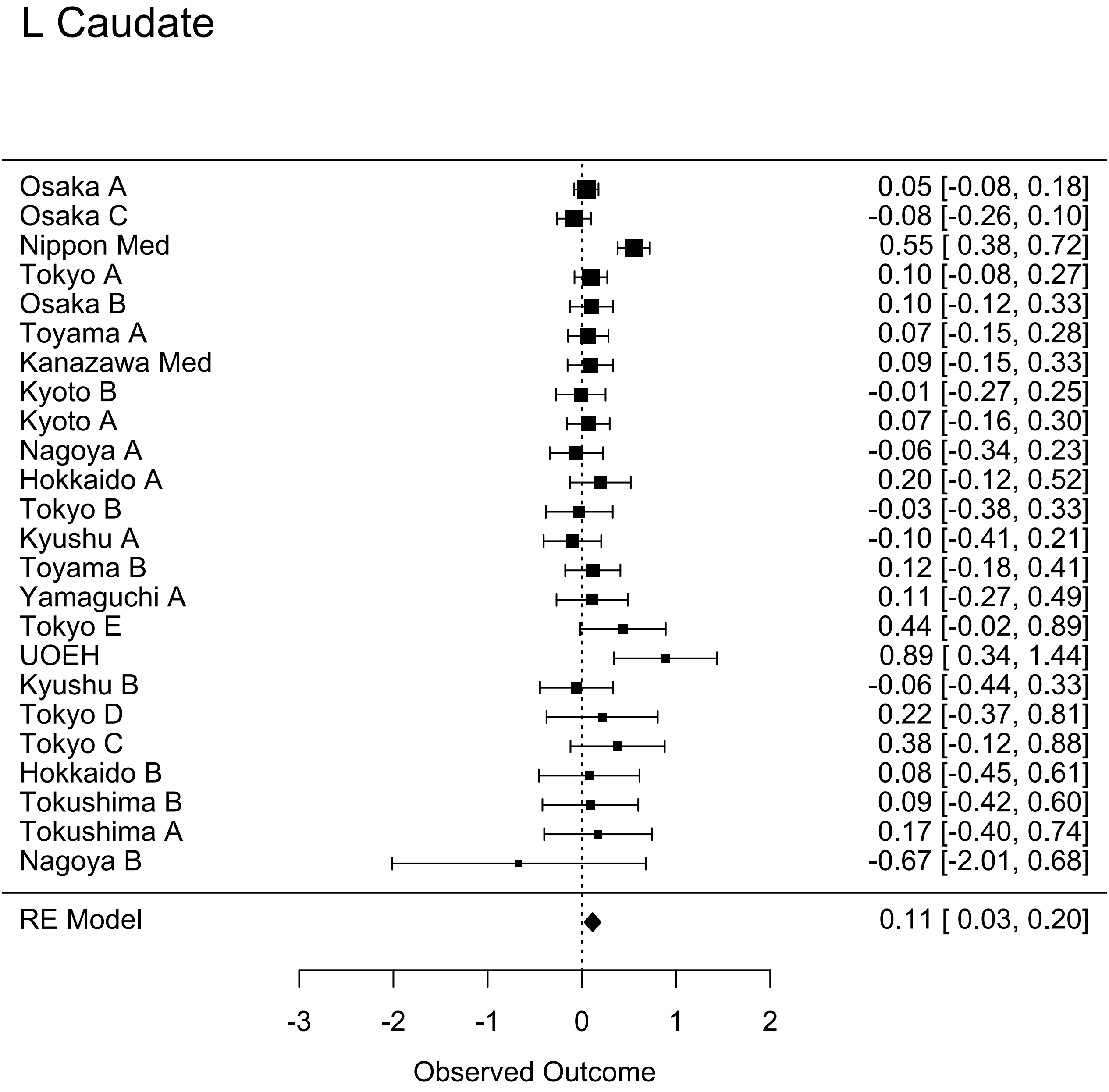
L Caudate
Osaka A 0.05 [-0.08, 0.18]
Osaka C -0.08 [-0.26, 0.10]
Nippon Med 0.55 [0.38, 0.72]
Tokyo A 0.10 [-0.08, 0.27]
Osaka B 0.10 [-0.12, 0.33]
Toyama A 0.07 [-0.15, 0.28]
Kanazawa Med 0.09 [-0.15, 0.33]
Kyoto B -0.01 [-0.27, 0.25]
Kyoto A 0.07 [-0.16, 0.30]
Nagoya A -0.06 [-0.34, 0.23]
Hokkaido A 0.20 [-0.12, 0.52]
Tokyo B -0.03 [-0.38, 0.33]
Kyushu A -0.10 [-0.41, 0.21]
Toyama B 0.12 [-0.18, 0.41]
Yamaguchi A 0.11 [-0.27, 0.49]
Tokyo E 0.44 [-0.02, 0.89]
UOEH 0.89 [0.34, 1.44]
Kyushu B -0.06 [-0.44, 0.33]
Tokyo D 0.22 [-0.37, 0.81]
Tokyo C 0.38 [-0.12, 0.88]
Hokkaido B 0.08 [-0.45, 0.61]
Tokushima B 0.09 [-0.42, 0.60]
Tokushima A 0.17 [-0.40, 0.74]
Nagoya B -0.67 [-2.01, 0.68]
RE Model 0.11 [0.03, 0.20]
-3
-2
-1
0
1
2
Observed Outcome

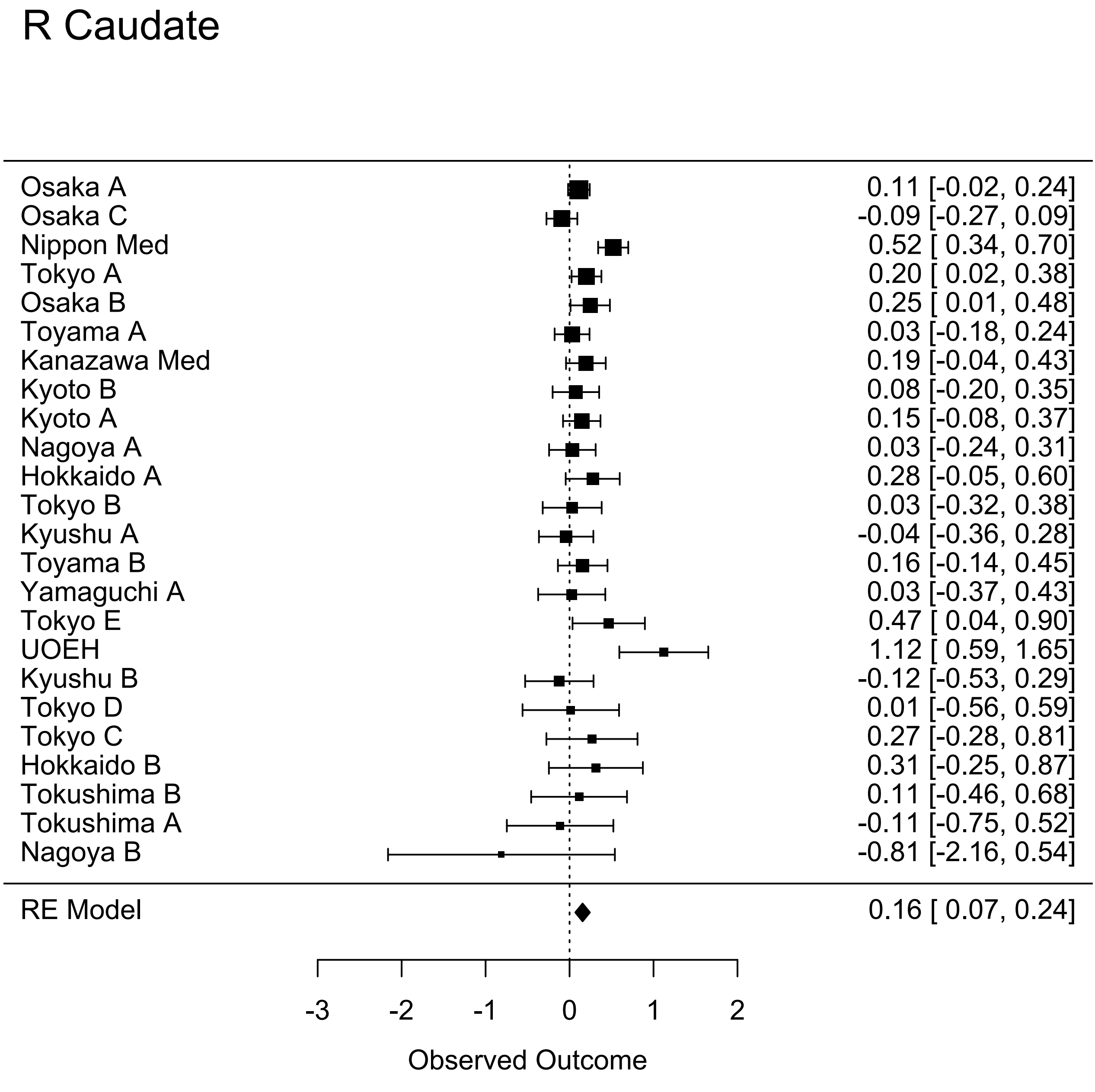
R Caudate
Osaka A 0.11 [-0.02, 0.24]
Osaka C -0.09 [-0.27, 0.09]
Nippon Med 0.52 [0.34, 0.70]
Tokyo A 0.20 [0.02, 0.38]
Osaka B 0.25 [0.01, 0.48]
Toyama A 0.03 [-0.18, 0.24]
Kanazawa Med 0.19 [-0.04, 0.43]
Kyoto B 0.08 [-0.20, 0.35]
Kyoto A 0.15 [-0.08, 0.37]
Nagoya A 0.03 [-0.24, 0.31]
Hokkaido A 0.28 [-0.05, 0.60]
Tokyo B 0.03 [-0.32, 0.38]
Kyushu A -0.04 [-0.36, 0.28]
Toyama B 0.16 [-0.14, 0.45]
Yamaguchi A 0.03 [-0.37, 0.43]
Tokyo E 0.47 [0.04, 0.90]
UOEH 1.12 [0.59, 1.65]
Kyushu B -0.12 [-0.53, 0.29]
Tokyo D 0.01 [-0.56, 0.59]
Tokyo C 0.27 [-0.28, 0.81]
Hokkaido B 0.31 [-0.25, 0.87]
Tokushima B 0.11 [-0.46, 0.68]
Tokushima A -0.11 [-0.75, 0.52]
Nagoya B -0.81 [-2.16, 0.54]
RE Model 0.16 [0.07, 0.24]
-3
-2
-1
0
1
2
Observed Outcome

L Putamen

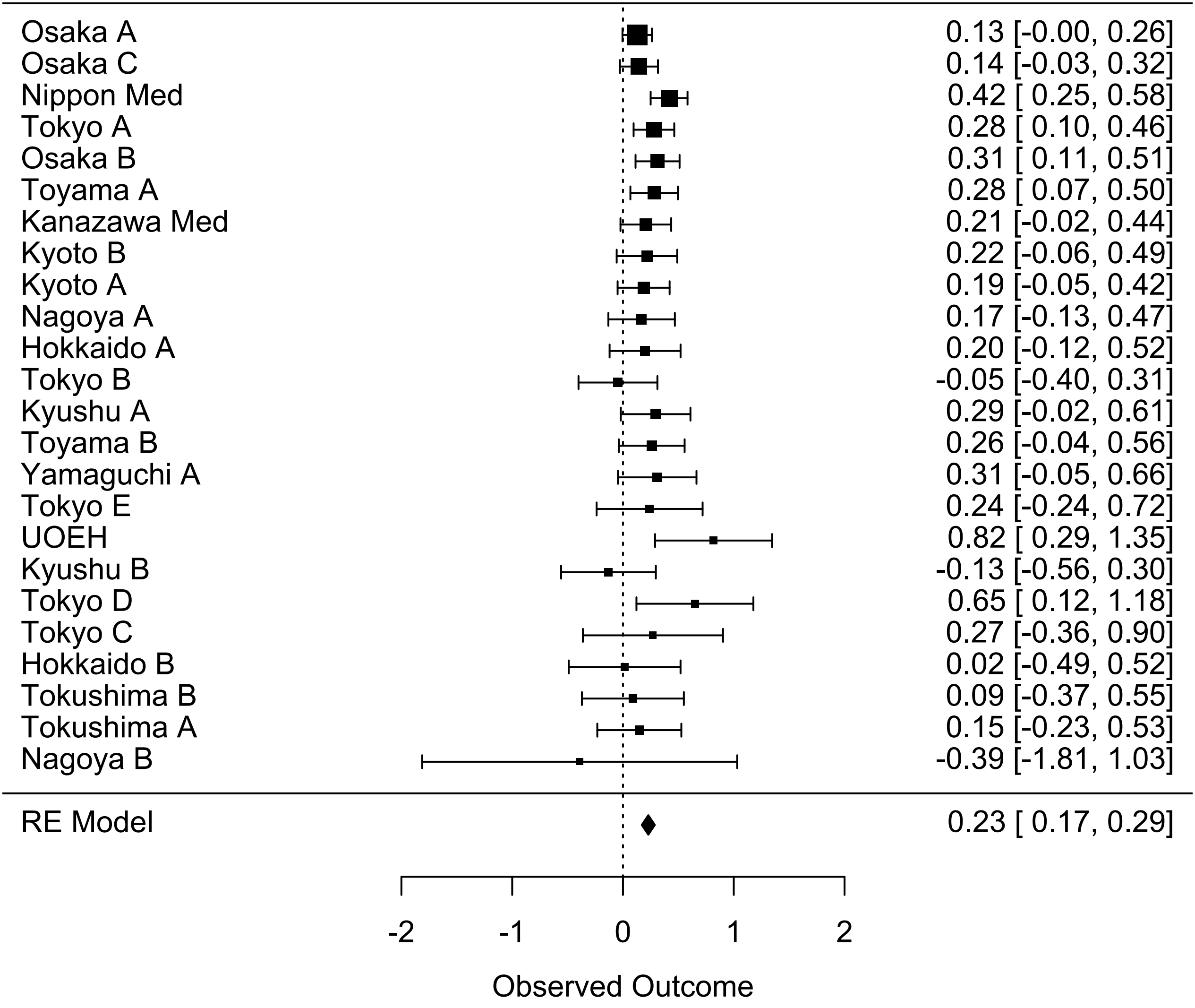

Osaka A 0.13 [-0.00, 0.26]
Osaka C 0.14 [-0.03, 0.32]
Nippon Med 0.42 [0.25, 0.58]
Tokyo A 0.28 [0.10, 0.46]
Osaka B 0.31 [0.11, 0.51]
Toyama A 0.28 [0.07, 0.50]
Kanazawa Med 0.21 [-0.02, 0.44]
Kyoto B 0.22 [-0.06, 0.49]
Kyoto A 0.19 [-0.05, 0.42]
Nagoya A 0.17 [-0.13, 0.47]
Hokkaido A 0.20 [-0.12, 0.52]
Tokyo B -0.05 [-0.40, 0.31]
Kyushu A 0.29 [-0.02, 0.61]
Toyama B 0.26 [-0.04, 0.56]
Yamaguchi A 0.31 [-0.05, 0.66]
Tokyo E 0.24 [-0.24, 0.72]
UOEH 0.82 [0.29, 1.35]
Kyushu B -0.13 [-0.56, 0.30]
Tokyo D 0.65 [0.12, 1.18]
Tokyo C 0.27 [-0.36, 0.90]
Hokkaido B 0.02 [-0.49, 0.52]
Tokushima B 0.09 [-0.37, 0.55]
Tokushima A 0.15 [-0.23, 0.53]
Nagoya B -0.39 [-1.81, 1.03]
RE Model 0.23 [0.17, 0.29]
-2
-1
0
1
2
Observed Outcome

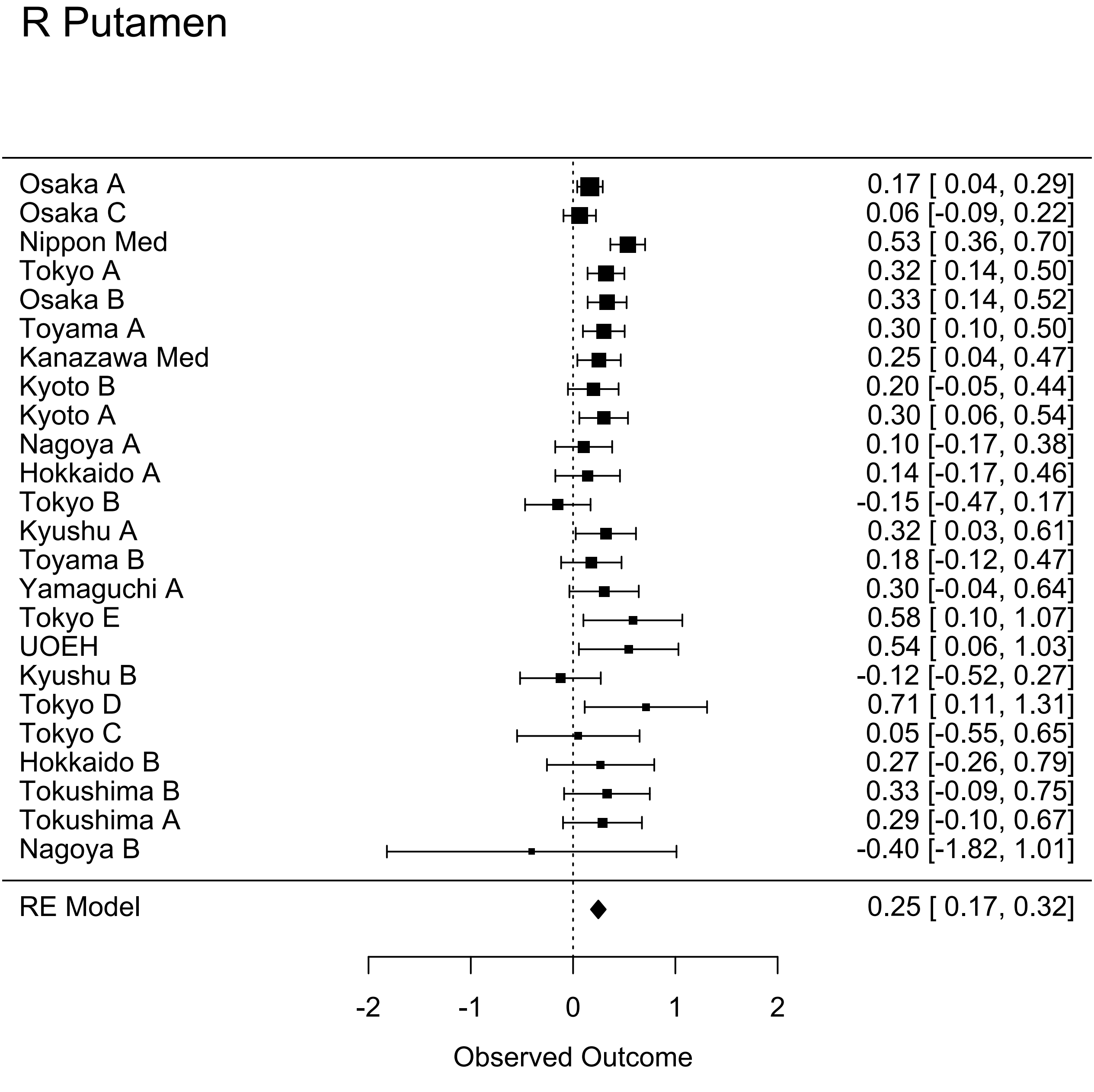
R Putamen
Osaka A 0.17 [0.04, 0.29]
Osaka C 0.06 [-0.09, 0.22]
Nippon Med 0.53 [0.36, 0.70]
Tokyo A 0.32 [0.14, 0.50]
Osaka B 0.33 [0.14, 0.52]
Toyama A 0.30 [0.10, 0.50]
Kanazawa Med 0.25 [0.04, 0.47]
Kyoto B 0.20 [-0.05, 0.44]
Kyoto A 0.30 [0.06, 0.54]
Nagoya A 0.10 [-0.17, 0.38]
Hokkaido A 0.14 [-0.17, 0.46]
Tokyo B -0.15 [-0.47, 0.17]
Kyushu A 0.32 [0.03, 0.61]
Toyama B 0.18 [-0.12, 0.47]
Yamaguchi A 0.30 [-0.04, 0.64]
Tokyo E 0.58 [0.10, 1.07]
UOEH 0.54 [0.06, 1.03]
Kyushu B -0.12 [-0.52, 0.27]
Tokyo D 0.71 [0.11, 1.31]
Tokyo C 0.05 [-0.55, 0.65]
Hokkaido B 0.27 [-0.26, 0.79]
Tokushima B 0.33 [-0.09, 0.75]
Tokushima A 0.29 [-0.10, 0.67]
Nagoya B -0.40 [-1.82, 1.01]
RE Model 0.25 [0.17, 0.32]
-2
-1
0
1
2
Observed Outcome

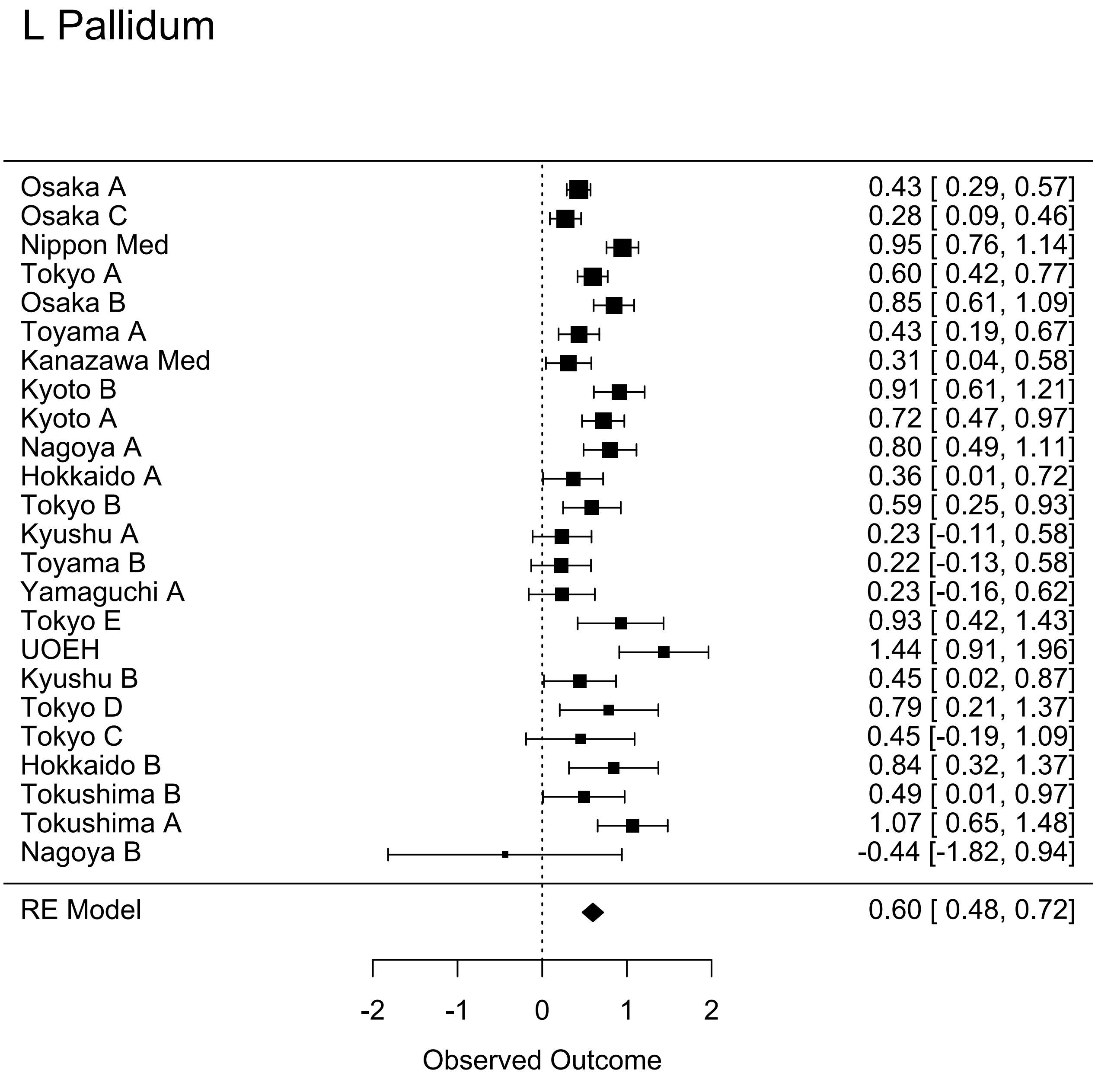
L Pallidum
Osaka A 0.43 [0.29, 0.57]
Osaka C 0.28 [0.09, 0.46]
Nippon Med 0.95 [0.76, 1.14]
Tokyo A 0.60 [0.42, 0.77]
Osaka B 0.85 [0.61, 1.09]
Toyama A 0.43 [0.19, 0.67]
Kanazawa Med 0.31 [0.04, 0.58]
Kyoto B 0.91 [0.61, 1.21]
Kyoto A 0.72 [0.47, 0.97]
Nagoya A 0.80 [0.49, 1.11]
Hokkaido A 0.36 [0.01, 0.72]
Tokyo B 0.59 [0.25, 0.93]
Kyushu A 0.23 [-0.11, 0.58]
Toyama B 0.22 [-0.13, 0.58]
Yamaguchi A 0.23 [-0.16, 0.62]
Tokyo E 0.93 [0.42, 1.43]
UOEH 1.44 [0.91, 1.96]
Kyushu B 0.45 [0.02, 0.87]
Tokyo D 0.79 [0.21, 1.37]
Tokyo C 0.45 [-0.19, 1.09]
Hokkaido B 0.84 [0.32, 1.37]
Tokushima B 0.49 [0.01, 0.97]
Tokushima A 1.07 [0.65, 1.48]
Nagoya B -0.44 [-1.82, 0.94]
RE Model 0.60 [0.48, 0.72]
-2
-1
0
1
2
Observed Outcome

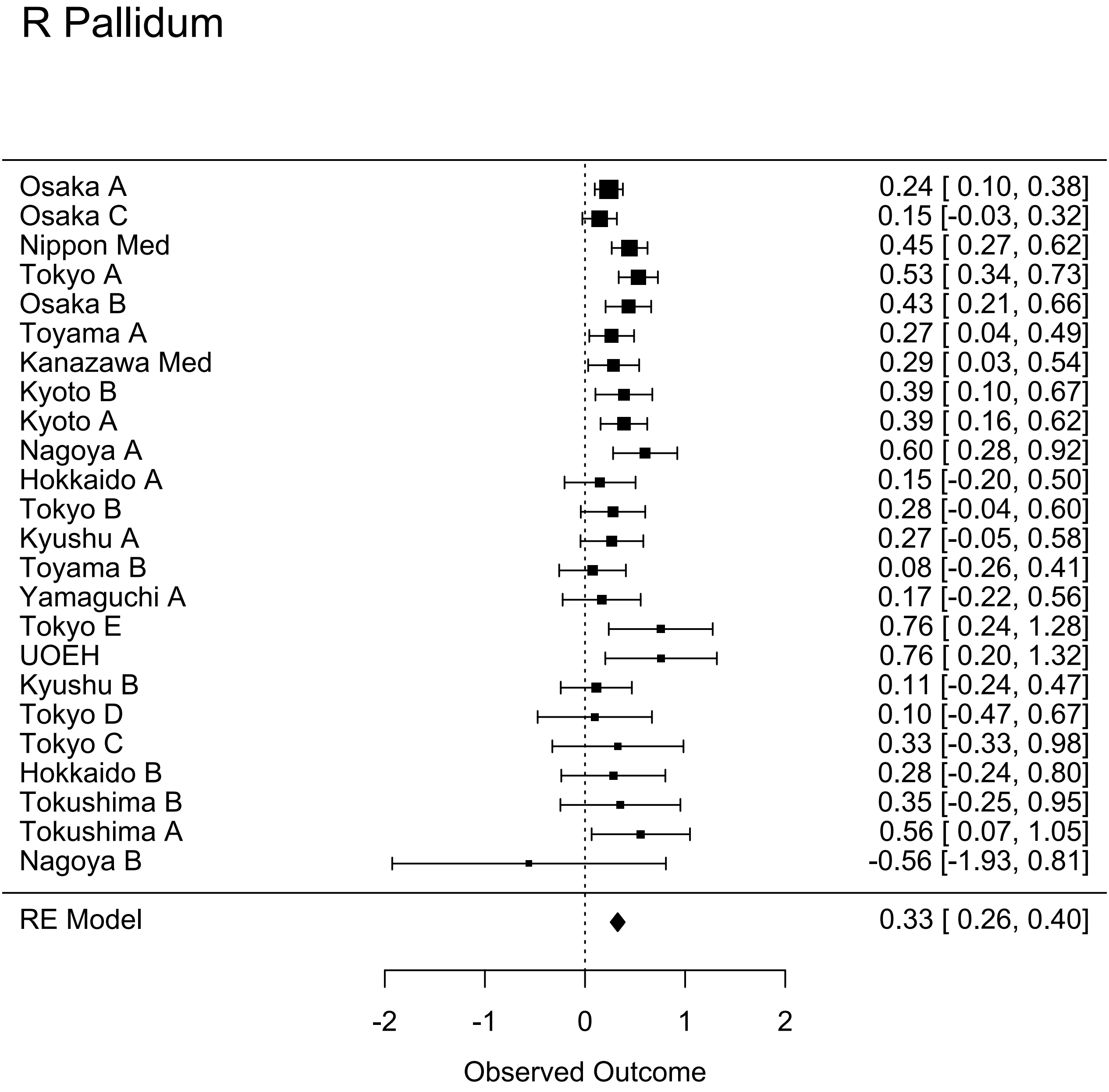
R Pallidum
Osaka A 0.24 [0.10, 0.38]
Osaka C 0.15 [-0.03, 0.32]
Nippon Med 0.45 [0.27, 0.62]
Tokyo A 0.53 [0.34, 0.73]
Osaka B 0.43 [0.21, 0.66]
Toyama A 0.27 [0.04, 0.49]
Kanazawa Med 0.29 [0.03, 0.54]
Kyoto B 0.39 [0.10, 0.67]
Kyoto A 0.39 [0.16, 0.62]
Nagoya A 0.60 [0.28, 0.92]
Hokkaido A 0.15 [-0.20, 0.50]
Tokyo B 0.28 [-0.04, 0.60]
Kyushu A 0.27 [-0.05, 0.58]
Toyama B 0.08 [-0.26, 0.41]
Yamaguchi A 0.17 [-0.22, 0.56]
Tokyo E 0.76 [0.24, 1.28]
UOEH 0.76 [0.20, 1.32]
Kyushu B 0.11 [-0.24, 0.47]
Tokyo D 0.10 [-0.47, 0.67]
Tokyo C 0.33 [-0.33, 0.98]
Hokkaido B 0.28 [-0.24, 0.80]
Tokushima B 0.35 [-0.25, 0.95]
Tokushima A 0.56 [0.07, 1.05]
Nagoya B -0.56 [-1.93, 0.81]
RE Model 0.33 [0.26, 0.40]
-2
-1
0
1
2
Observed Outcome

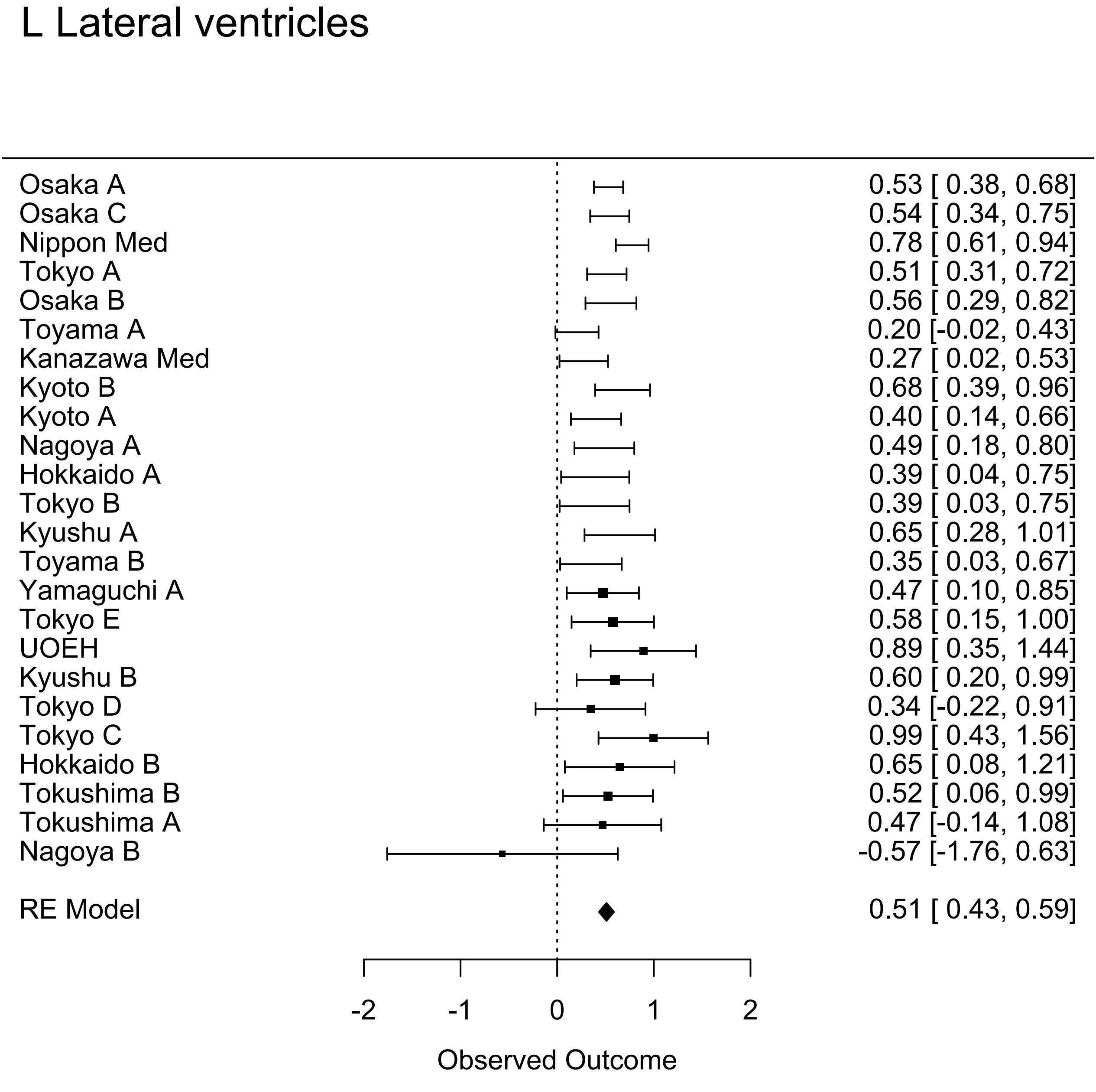

L Lateral ventricles
Osaka A 0.53 [0.38, 0.68]
Osaka C 0.54 [0.34, 0.75]
Nippon Med 0.78 [0.61, 0.94]
Tokyo A 0.51 [0.31, 0.72]
Osaka B 0.56 [0.29, 0.82]
Toyama A 0.20 [-0.02, 0.43]
Kanazawa Med 0.27 [0.02, 0.53]
Kyoto B 0.68 [0.39, 0.96]
Kyoto A 0.40 [0.14, 0.66]
Nagoya A 0.49 [0.18, 0.80]
Hokkaido A 0.39 [0.04, 0.75]
Tokyo B 0.39 [0.03, 0.75]
Kyushu A 0.65 [0.28, 1.01]
Toyama B 0.35 [0.03, 0.67]
Yamaguchi A 0.47 [0.10, 0.85]
Tokyo E 0.58 [0.15, 1.00]
UOEH 0.89 [0.35, 1.44]
Kyushu B 0.60 [0.20, 0.99]
Tokyo D 0.34 [-0.22, 0.91]
Tokyo C 0.99 [0.43, 1.56]
Hokkaido B 0.65 [0.08, 1.21]
Tokushima B 0.52 [0.06, 0.99]
Tokushima A 0.47 [-0.14, 1.08]
Nagoya B -0.57 [-1.76, 0.63]
RE Model 0.51 [0.43, 0.59]
-2
-1
0
1
2
Observed Outcome

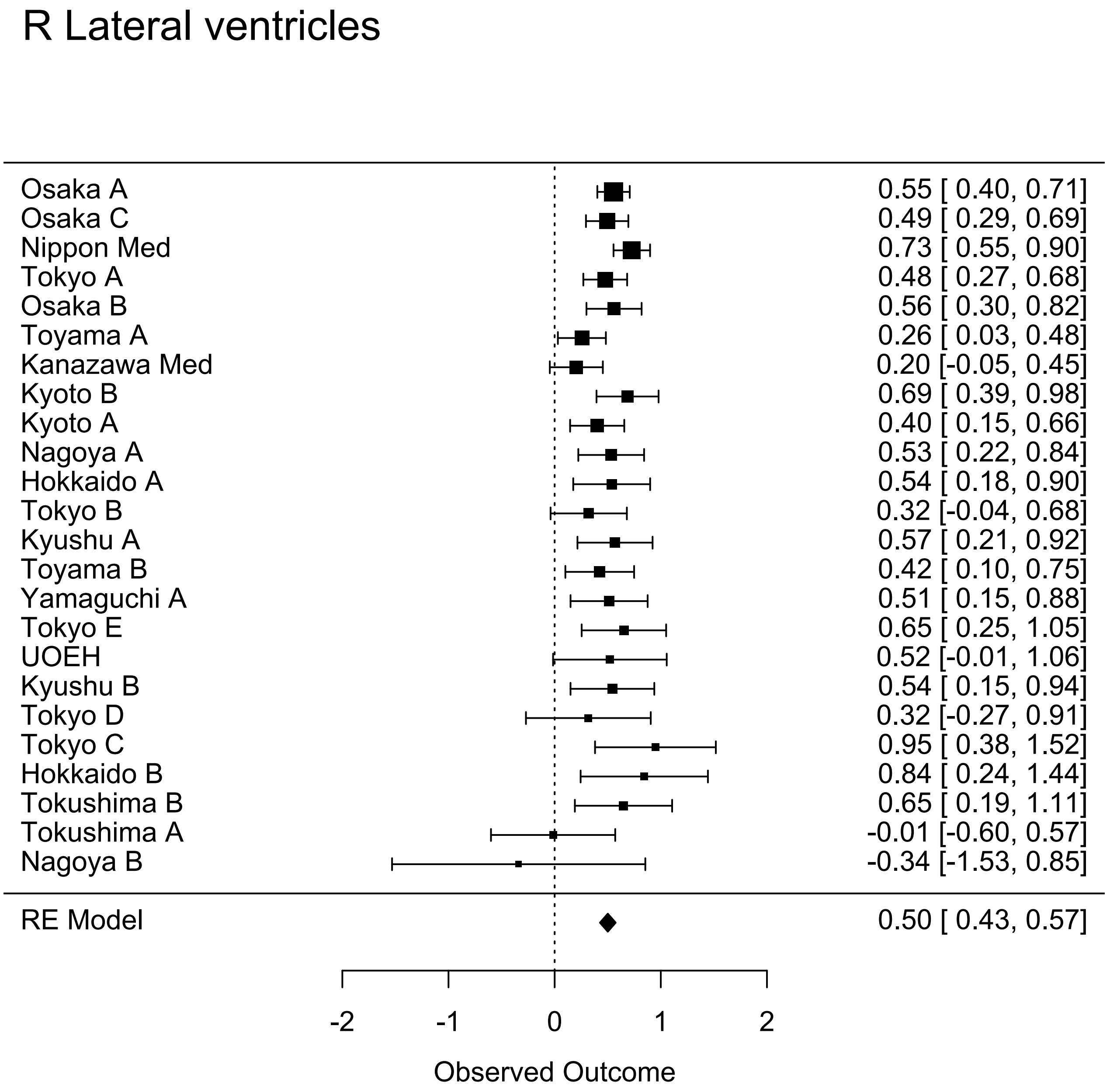
R Lateral ventricles
Osaka A 0.55 [0.40, 0.71]
Osaka C 0.49 [0.29, 0.69]
Nippon Med 0.73 [0.55, 0.90]
Tokyo A 0.48 [0.27, 0.68]
Osaka B 0.56 [0.30, 0.82]
Toyama A 0.26 [0.03, 0.48]
Kanazawa Med 0.20 [-0.05, 0.45]
Kyoto B 0.69 [0.39, 0.98]
Kyoto A 0.40 [0.15, 0.66]
Nagoya A 0.53 [0.22, 0.84]
Hokkaido A 0.54 [0.18, 0.90]
Tokyo B 0.32 [-0.04, 0.68]
Kyushu A 0.57 [0.21, 0.92]
Toyama B 0.42 [0.10, 0.75]
Yamaguchi A 0.51 [0.15, 0.88]
Tokyo E 0.65 [0.25, 1.05]
UOEH 0.52 [-0.01, 1.06]
Kyushu B 0.54 [0.15, 0.94]
Tokyo D 0.32 [-0.27, 0.91]
Tokyo C 0.95 [0.38, 1.52]
Hokkaido B 0.84 [0.24, 1.44]
Tokushima B 0.65 [0.19, 1.11]
Tokushima A -0.01 [-0.60, 0.57]
Nagoya B -0.34 [-1.53, 0.85]
RE Model 0.50 [0.43, 0.57]
-2
-1
0
1
2
Observed Outcome

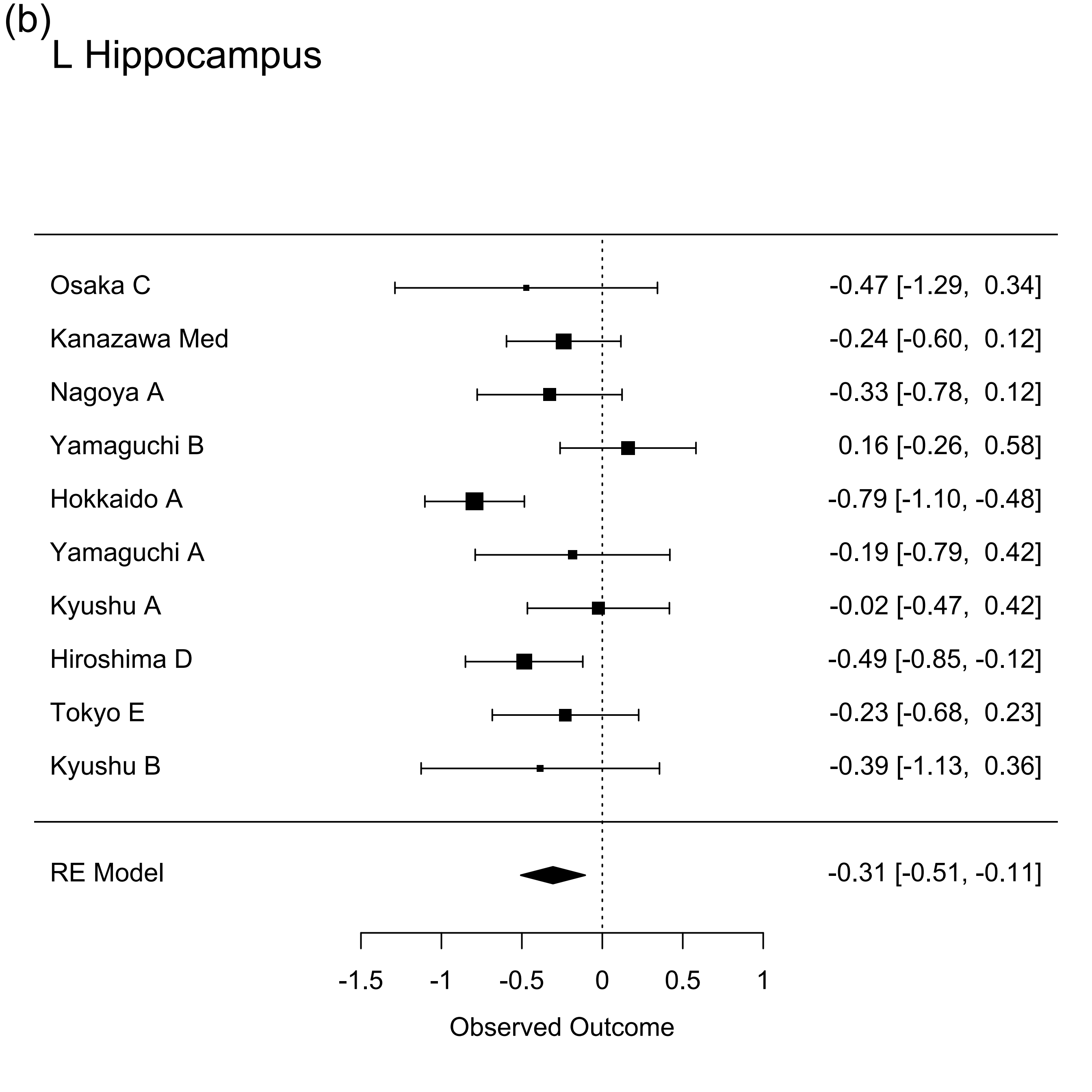
(b)
L Hippocampus
Osaka C
Kanazawa Med
Nagoya A
Yamaguchi B
Hokkaido A
Yamaguchi A
Kyushu A
Hiroshima D
Tokyo E
Kyushu B
RE Model
-0.47 [-1.29, 0.34]
-0.24 [-0.60, 0.12]
-0.33 [-0.78, 0.12]
0.16 [-0.26, 0.58]
-0.79 [-1.10, -0.48]
-0.19 [-0.79, 0.42]
-0.02 [-0.47, 0.42]
-0.49 [-0.85, -0.12]
-0.23 [-0.68, 0.23]
-0.39 [-1.13, 0.36]
-0.31 [-0.51, -0.11]
-1.5
-1
-0.5
0
0.5
1
Observed Outcome

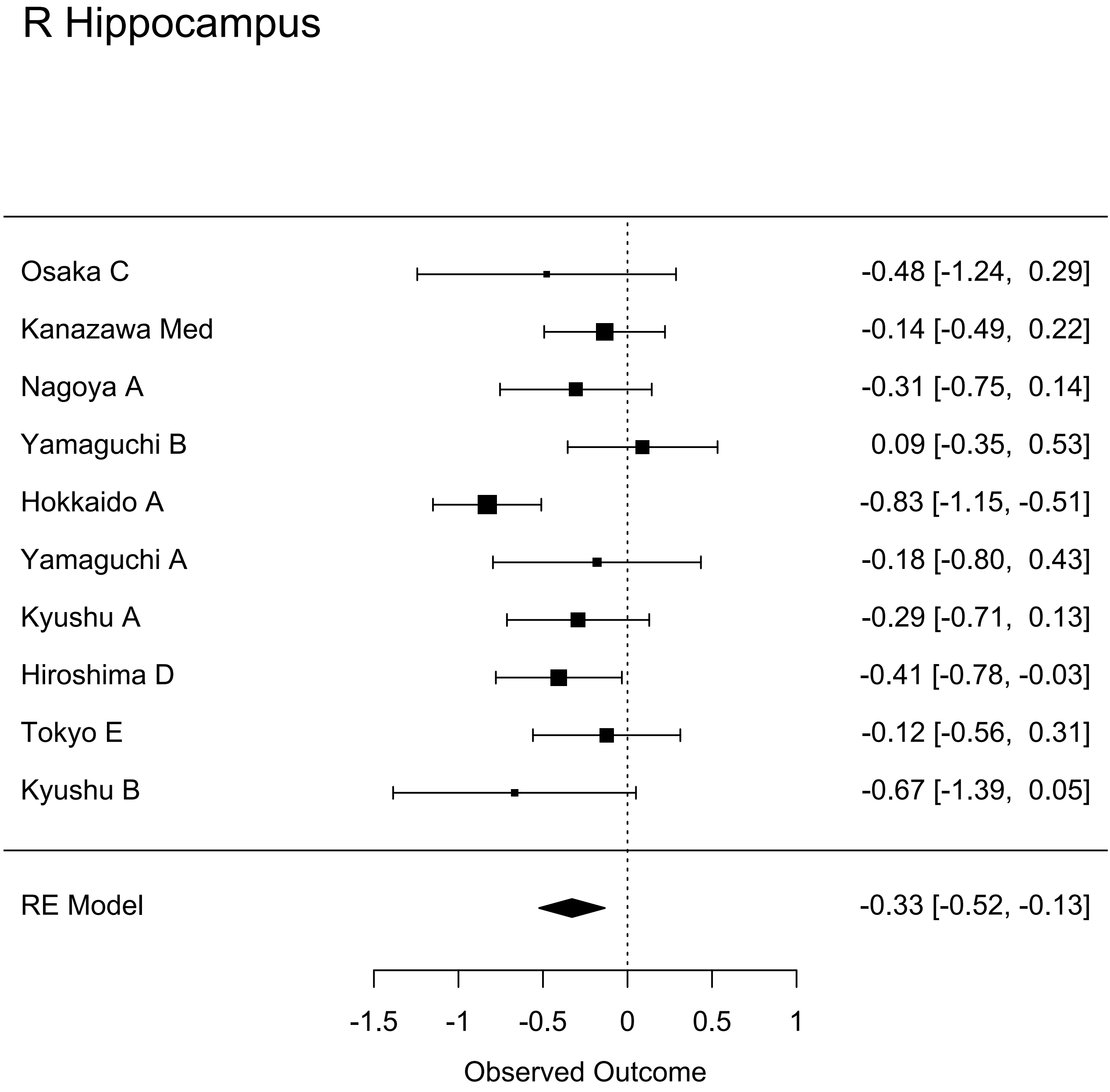
R Hippocampus
Osaka C -0.48 [-1.24, 0.29]
Kanazawa Med -0.14 [-0.49, 0.22]
Nagoya A -0.31 [-0.75, 0.14]
Yamaguchi B 0.09 [-0.35, 0.53]
Hokkaido A -0.83 [-1.15, -0.51]
Yamaguchi A -0.18 [-0.80, 0.43]
Kyushu A -0.29 [-0.71, 0.13]
Hiroshima D -0.41 [-0.78, -0.03]
Tokyo E -0.12 [-0.56, 0.31]
Kyushu B -0.67 [-1.39, 0.05]
RE Model -0.33 [-0.52, -0.13]
-1.5
-1
-0.5
0
0.5
1
Observed Outcome

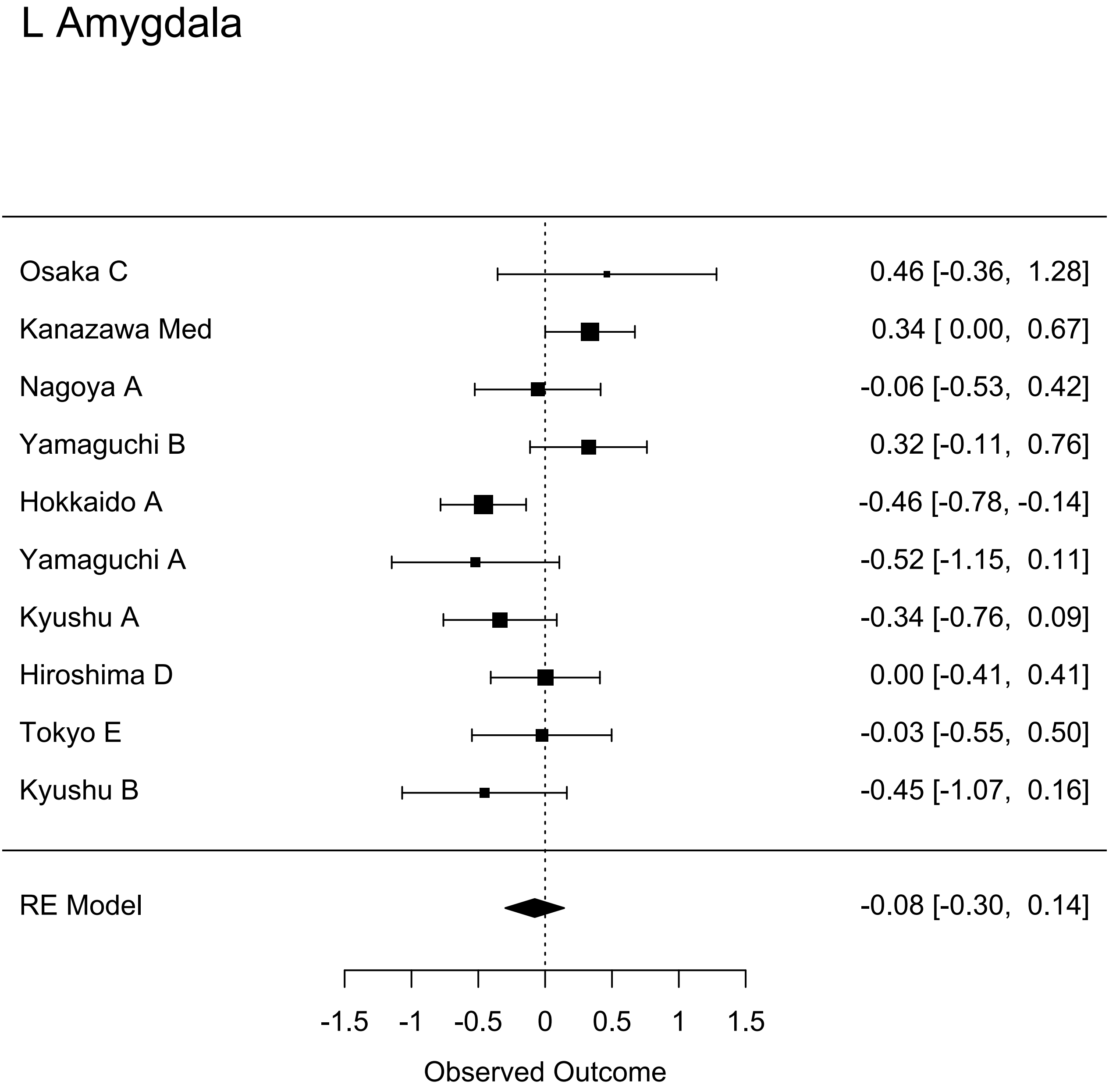
L Amygdala
Osaka C
Kanazawa Med
Nagoya A
Yamaguchi B
Hokkaido A
Yamaguchi A
Kyushu A
Hiroshima D
Tokyo E
Kyushu B
RE Model
0.46 [-0.36, 1.28]
0.34 [0.00, 0.67]
-0.06 [-0.53, 0.42]
0.32 [-0.11, 0.76]
-0.46 [-0.78, -0.14]
-0.52 [-1.15, 0.11]
-0.34 [-0.76, 0.09]
0.00 [-0.41, 0.41]
-0.03 [-0.55, 0.50]
-0.45 [-1.07, 0.16]
-0.08 [-0.30, 0.14]
-1.5
-1
-0.5
0
0.5
1
1.5
Observed Outcome

R Amygdala

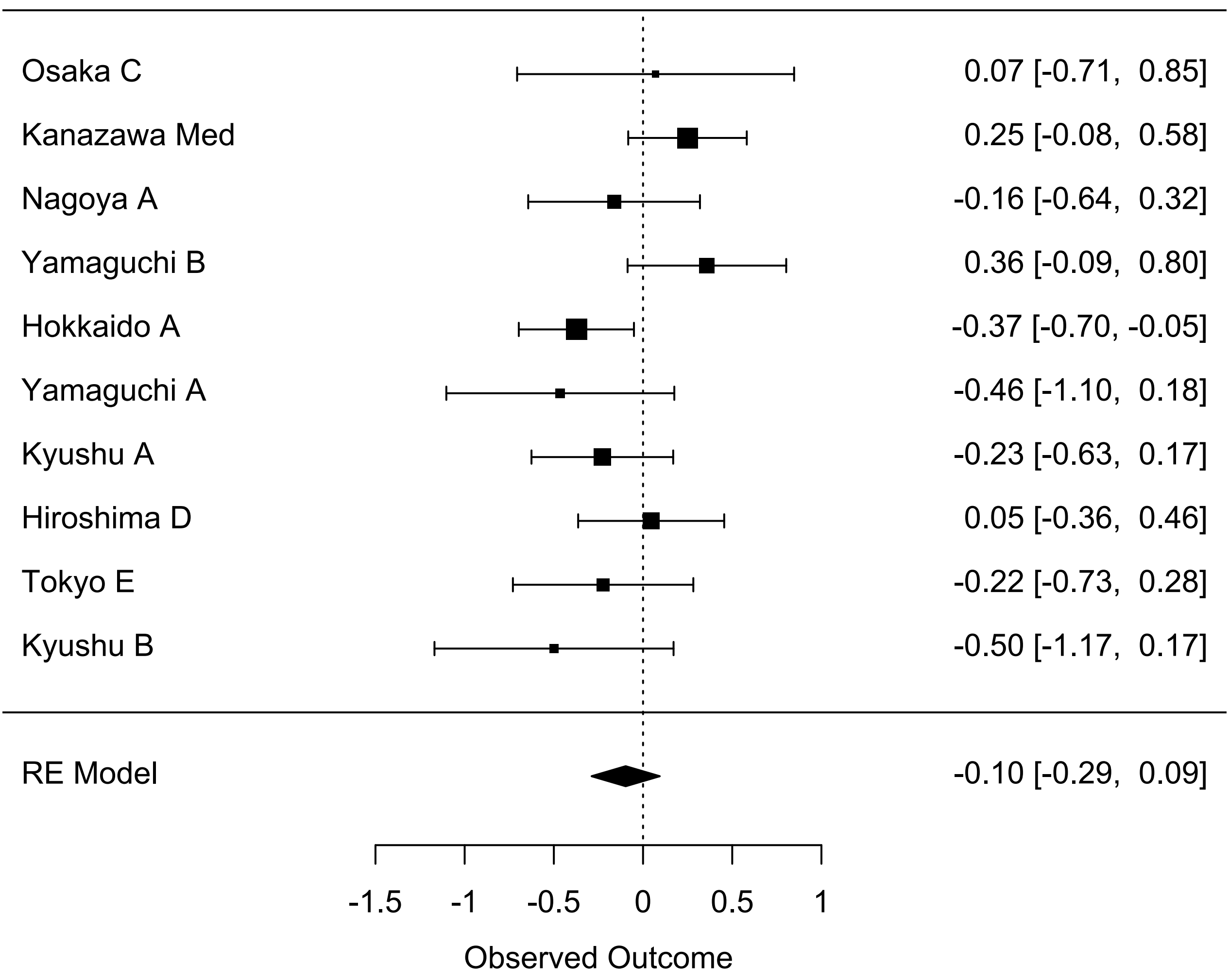
Osaka C
Kanazawa Med
Nagoya A
Yamaguchi B
Hokkaido A
Yamaguchi A
Kyushu A
Hiroshima D
Tokyo E
Kyushu B
RE Model
0.07 [-0.71, 0.85]
0.25 [-0.08, 0.58]
-0.16 [-0.64, 0.32]
0.36 [-0.09, 0.80]
-0.37 [-0.70, -0.05]
-0.46 [-1.10, 0.18]
-0.23 [-0.63, 0.17]
0.05 [-0.36, 0.46]
-0.22 [-0.73, 0.28]
-0.50 [-1.17, 0.17]
-0.10 [-0.29, 0.09]
-1.5
-1
-0.5
0
0.5
1
Observed Outcome

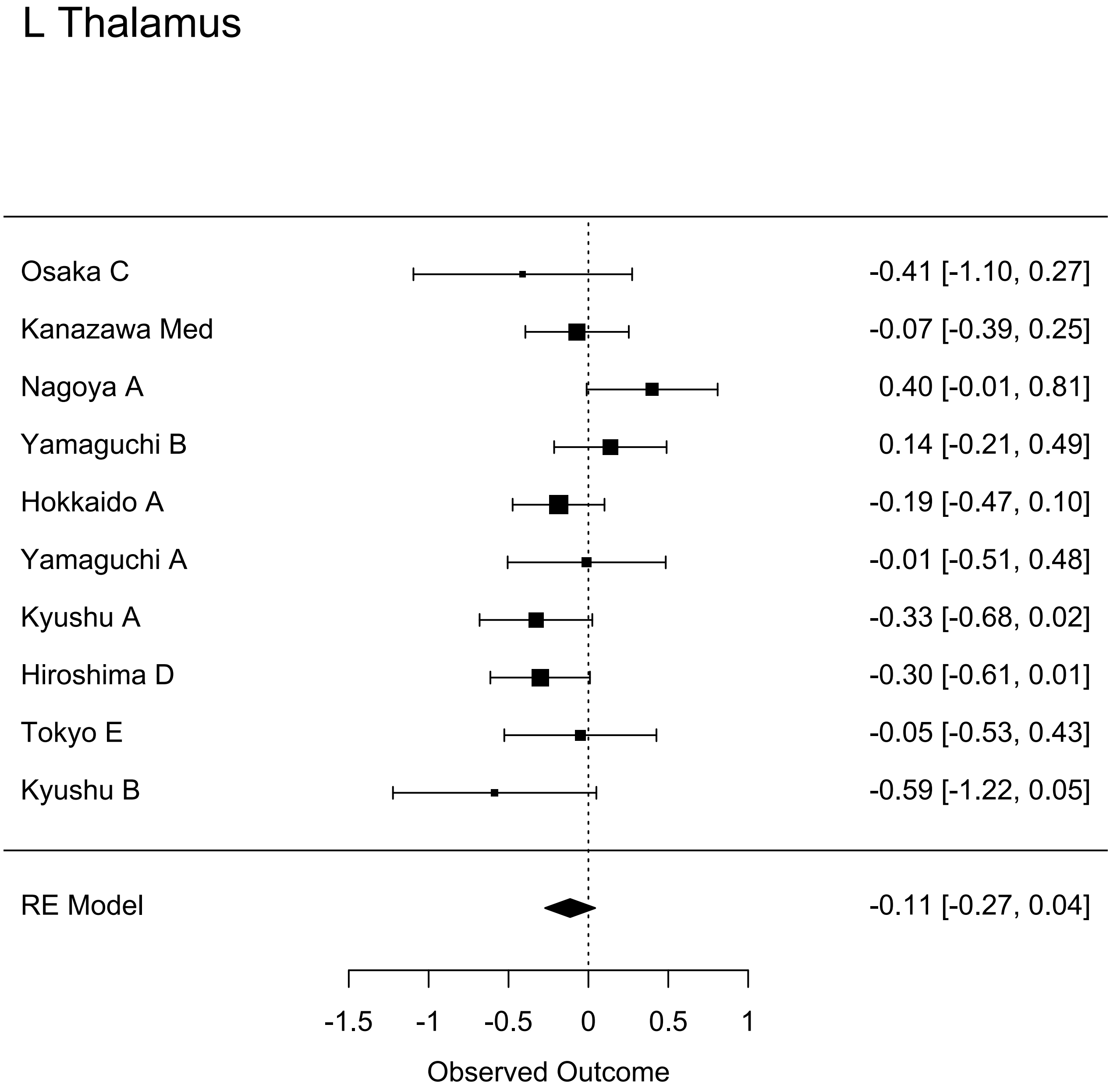
L Thalamus
Osaka C
-0.41 [-1.10, 0.27]
Kanazawa Med
-0.07 [-0.39, 0.25]
Nagoya A
0.40 [-0.01, 0.81]
Yamaguchi B
0.14 [-0.21, 0.49]
Hokkaido A
-0.19 [-0.47, 0.10]
Yamaguchi A
-0.01 [-0.51, 0.48]
Kyushu A
-0.33 [-0.68, 0.02]
Hiroshima D
-0.30 [-0.61, 0.01]
Tokyo E
-0.05 [-0.53, 0.43]
Kyushu B
-0.59 [-1.22, 0.05]
RE Model
-0.11 [-0.27, 0.04]
-1.5
-1
-0.5
0
0.5
1
Observed Outcome

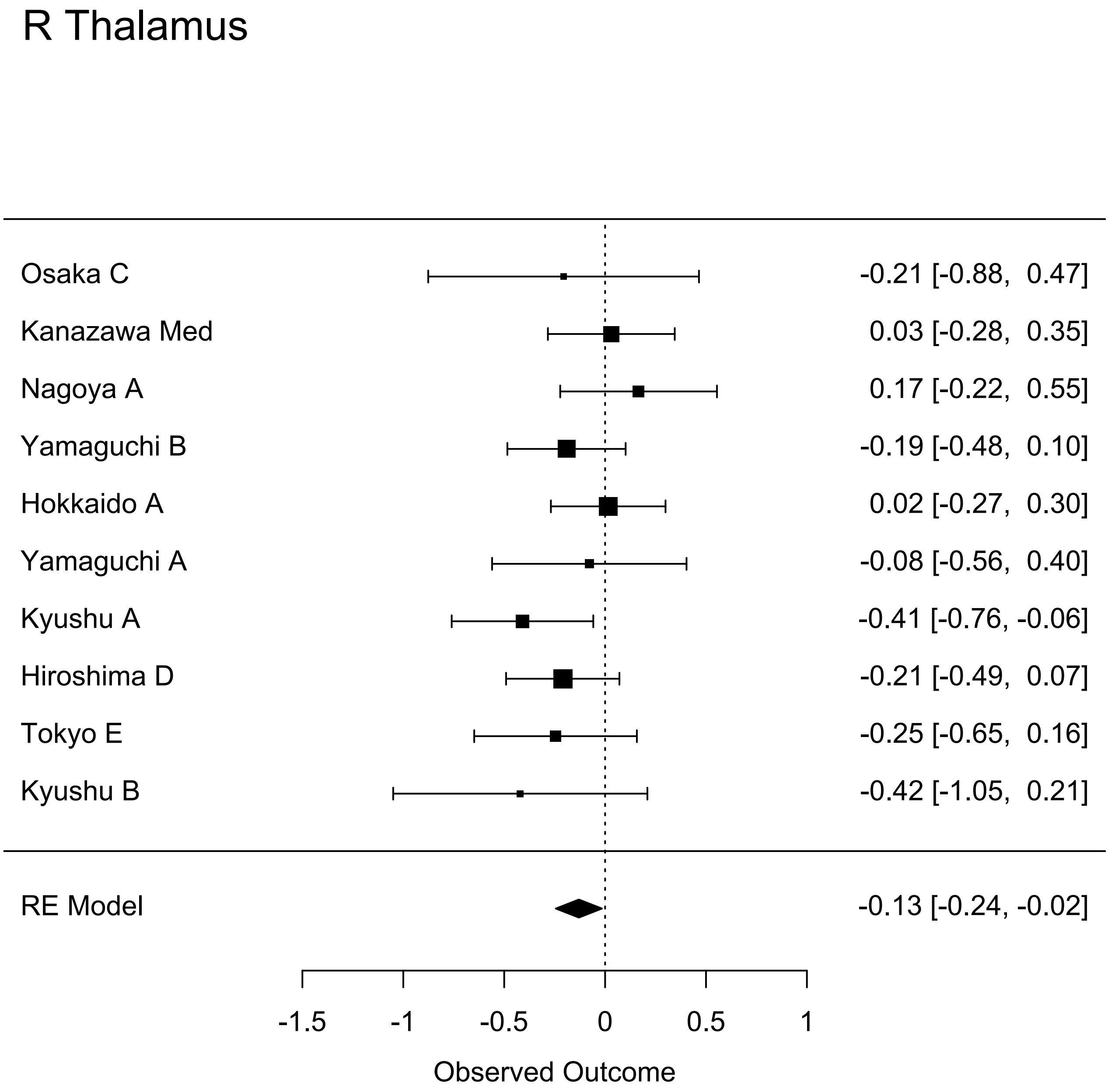
R Thalamus
Osaka C -0.21 [-0.88, 0.47]
Kanazawa Med 0.03 [-0.28, 0.35]
Nagoya A 0.17 [-0.22, 0.55]
Yamaguchi B -0.19 [-0.48, 0.10]
Hokkaido A 0.02 [-0.27, 0.30]
Yamaguchi A -0.08 [-0.56, 0.40]
Kyushu A -0.41 [-0.76, -0.06]
Hiroshima D -0.21 [-0.49, 0.07]
Tokyo E -0.25 [-0.65, 0.16]
Kyushu B -0.42 [-1.05, 0.21]
RE Model -0.13 [-0.24, -0.02]
-1.5
-1
-0.5
0
0.5
1
Observed Outcome

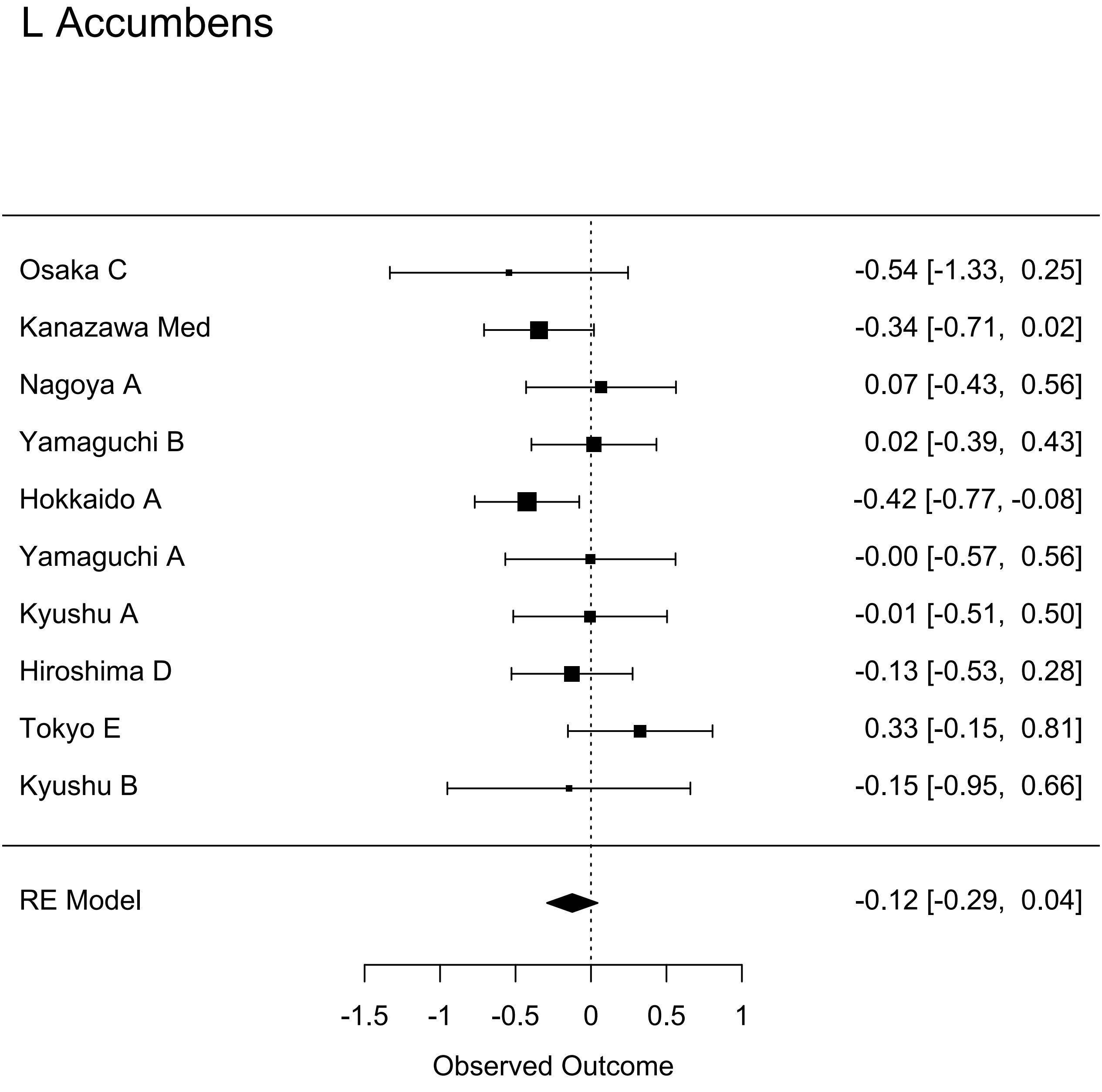

L Accumbens
Osaka C -0.54 [-1.33, 0.25]
Kanazawa Med -0.34 [-0.71, 0.02]
Nagoya A 0.07 [-0.43, 0.56]
Yamaguchi B 0.02 [-0.39, 0.43]
Hokkaido A -0.42 [-0.77, -0.08]
Yamaguchi A -0.00 [-0.57, 0.56]
Kyushu A -0.01 [-0.51, 0.50]
Hiroshima D -0.13 [-0.53, 0.28]
Tokyo E 0.33 [-0.15, 0.81]
Kyushu B -0.15 [-0.95, 0.66]
RE Model -0.12 [-0.29, 0.04]
-1.5
-1
-0.5
0
0.5
1
Observed Outcome

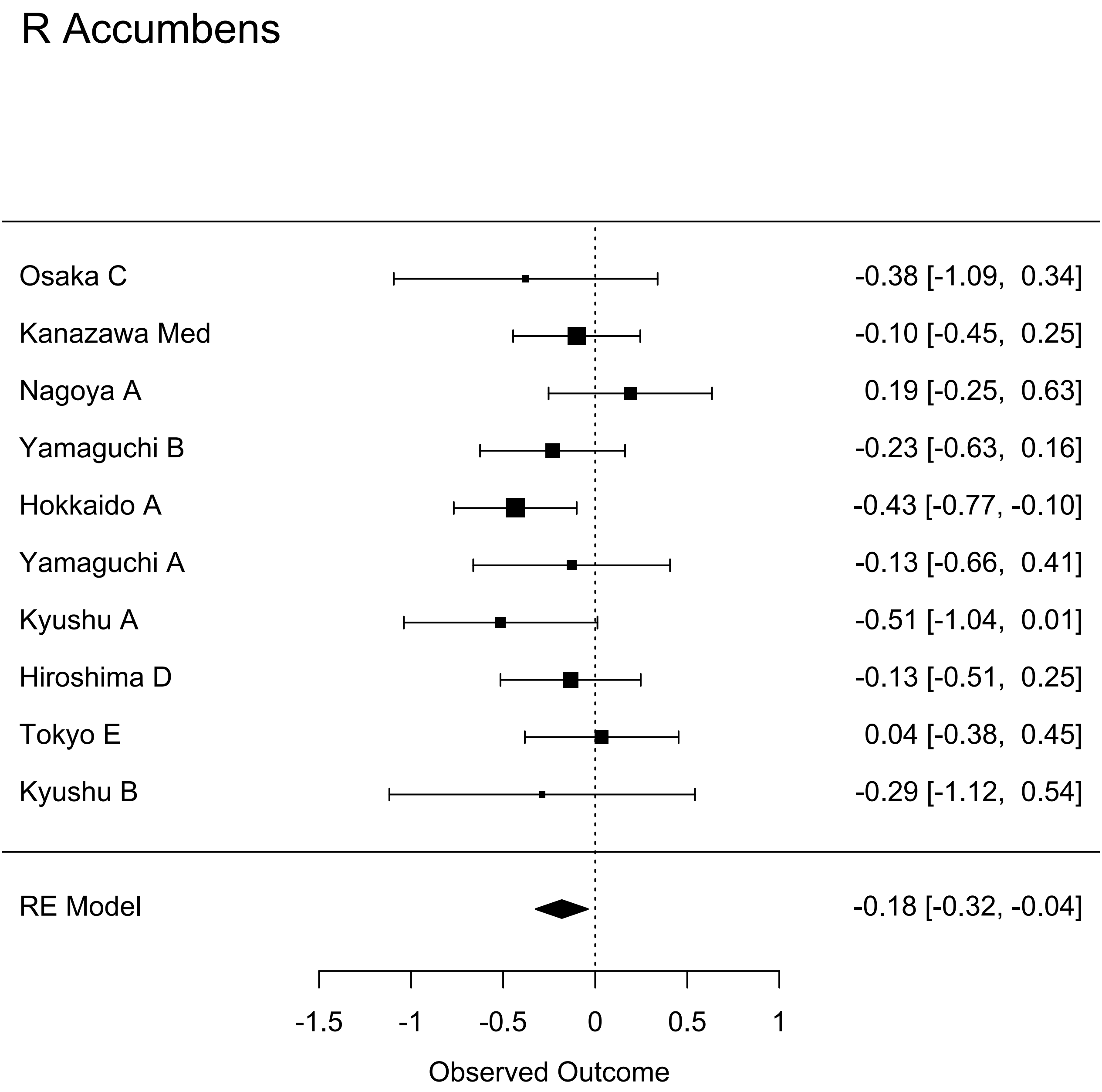
R Accumbens
Osaka C
Kanazawa Med
Nagoya A
Yamaguchi B
Hokkaido A
Yamaguchi A
Kyushu A
Hiroshima D
Tokyo E
Kyushu B
RE Model
-0.38 [-1.09, 0.34]
-0.10 [-0.45, 0.25]
0.19 [-0.25, 0.63]
-0.23 [-0.63, 0.16]
-0.43 [-0.77, -0.10]
-0.13 [-0.66, 0.41]
-0.51 [-1.04, 0.01]
-0.13 [-0.51, 0.25]
0.04 [-0.38, 0.45]
-0.29 [-1.12, 0.54]
-0.18 [-0.32, -0.04]
-1.5
-1
-0.5
0
0.5
1
Observed Outcome

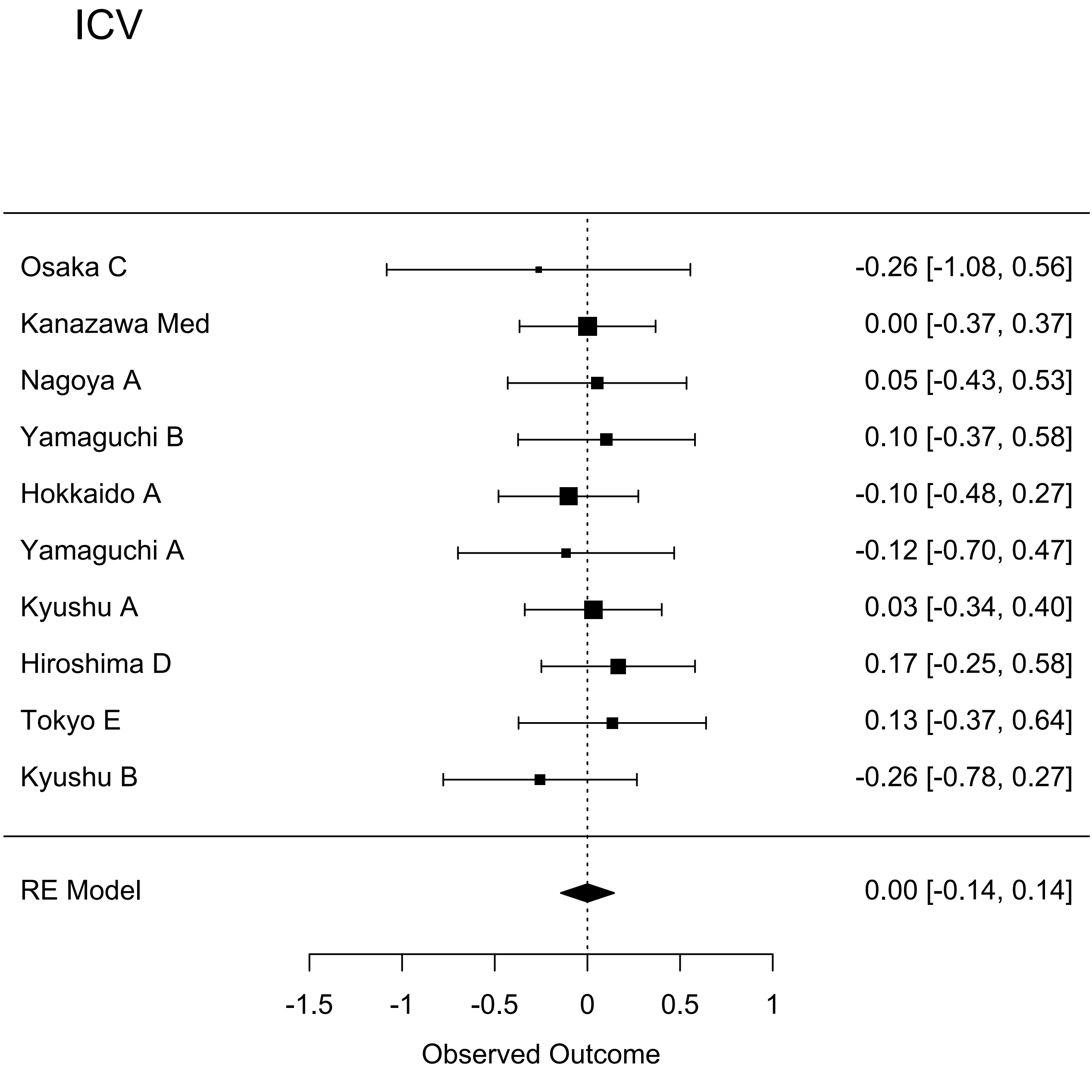
ICV
Osaka C
Kanazawa Med
Nagoya A
Yamaguchi B
Hokkaido A
Yamaguchi A
Kyushu A
Hiroshima D
Tokyo E
Kyushu B
RE Model
-0.26 [-1.08, 0.56]
0.00 [-0.37, 0.37]
0.05 [-0.43, 0.53]
0.10 [-0.37, 0.58]
-0.10 [-0.48, 0.27]
-0.12 [-0.70, 0.47]
0.03 [-0.34, 0.40]
0.17 [-0.25, 0.58]
0.13 [-0.37, 0.64]
-0.26 [-0.78, 0.27]
0.00 [-0.14, 0.14]
-1.5
-1
-0.5
0
0.5
1
Observed Outcome

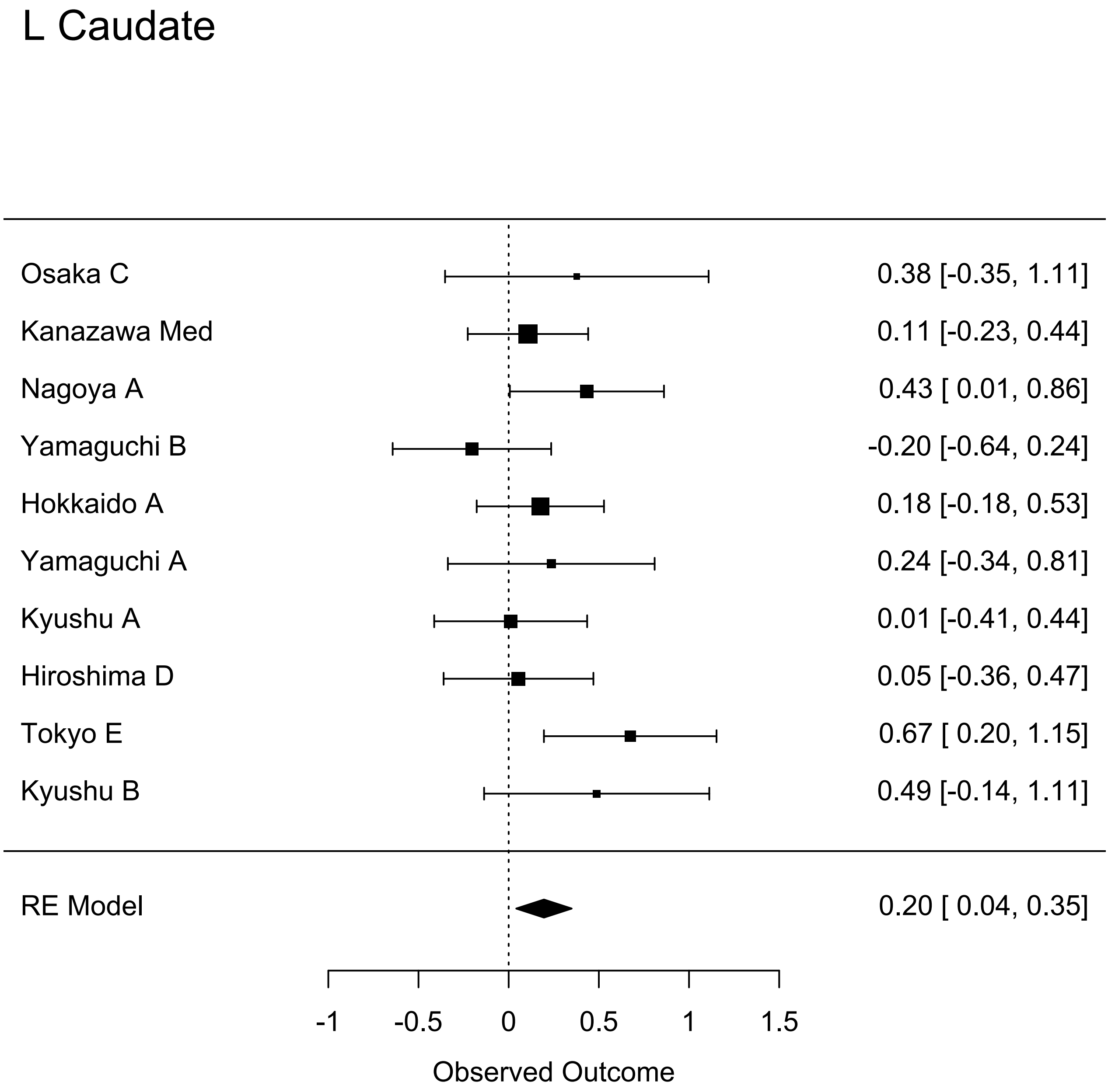
L Caudate
Osaka C
Kanazawa Med
Nagoya A
Yamaguchi B
Hokkaido A
Yamaguchi A
Kyushu A
Hiroshima D
Tokyo E
Kyushu B
RE Model
0.38 [-0.35, 1.11]
0.11 [-0.23, 0.44]
0.43 [0.01, 0.86]
-0.20 [-0.64, 0.24]
0.18 [-0.18, 0.53]
0.24 [-0.34, 0.81]
0.01 [-0.41, 0.44]
0.05 [-0.36, 0.47]
0.67 [0.20, 1.15]
0.49 [-0.14, 1.11]
0.20 [0.04, 0.35]
-1
-0.5
0
0.5
1
1.5
Observed Outcome

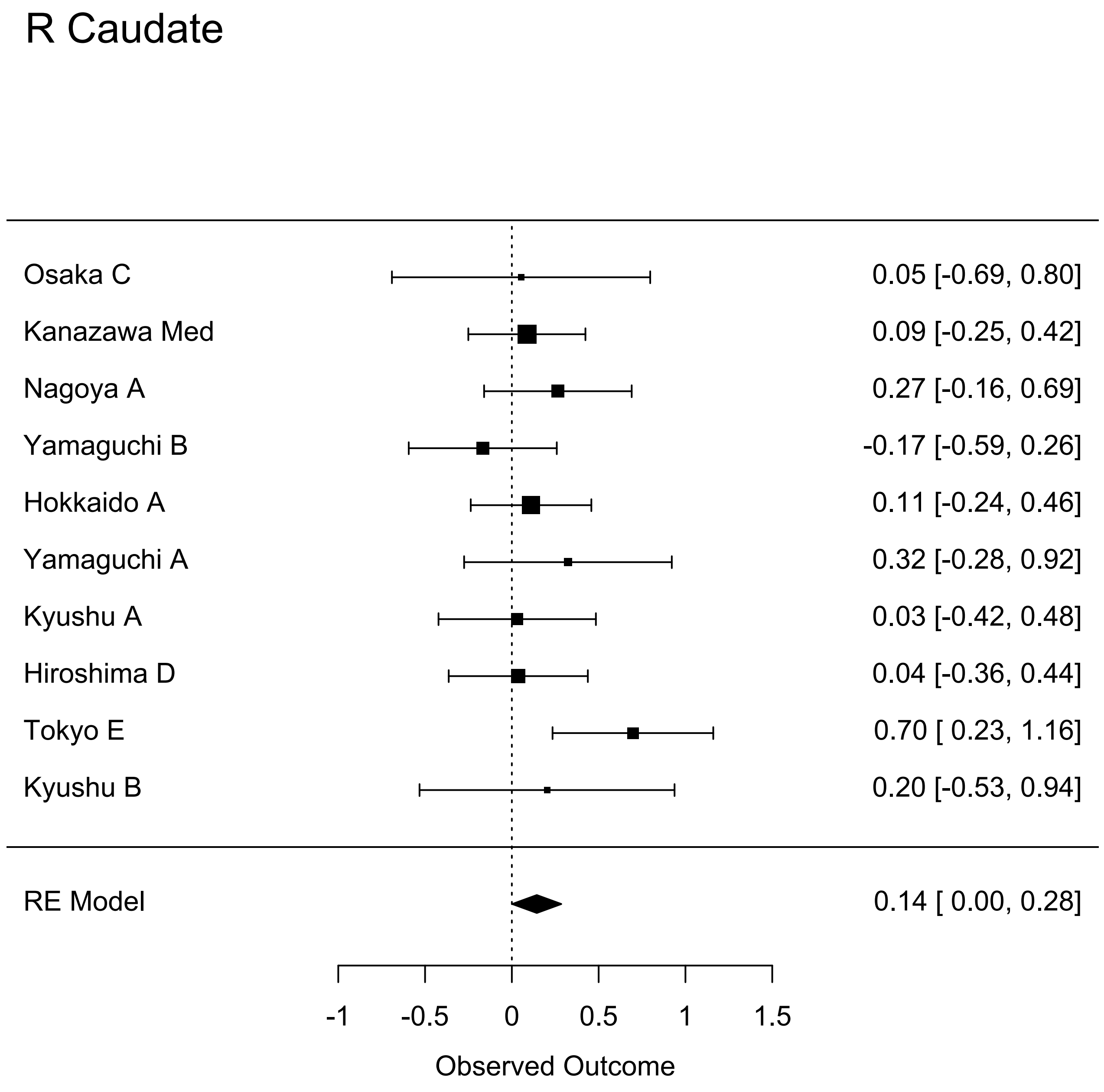
R Caudate
Osaka C
0.05 [-0.69, 0.80]
Kanazawa Med
0.09 [-0.25, 0.42]
Nagoya A
0.27 [-0.16, 0.69]
Yamaguchi B
-0.17 [-0.59, 0.26]
Hokkaido A
0.11 [-0.24, 0.46]
Yamaguchi A
0.32 [-0.28, 0.92]
Kyushu A
0.03 [-0.42, 0.48]
Hiroshima D
0.04 [-0.36, 0.44]
Tokyo E
0.70 [0.23, 1.16]
Kyushu B
0.20 [-0.53, 0.94]
RE Model
0.14 [0.00, 0.28]
-1
-0.5
0
0.5
1
1.5
Observed Outcome

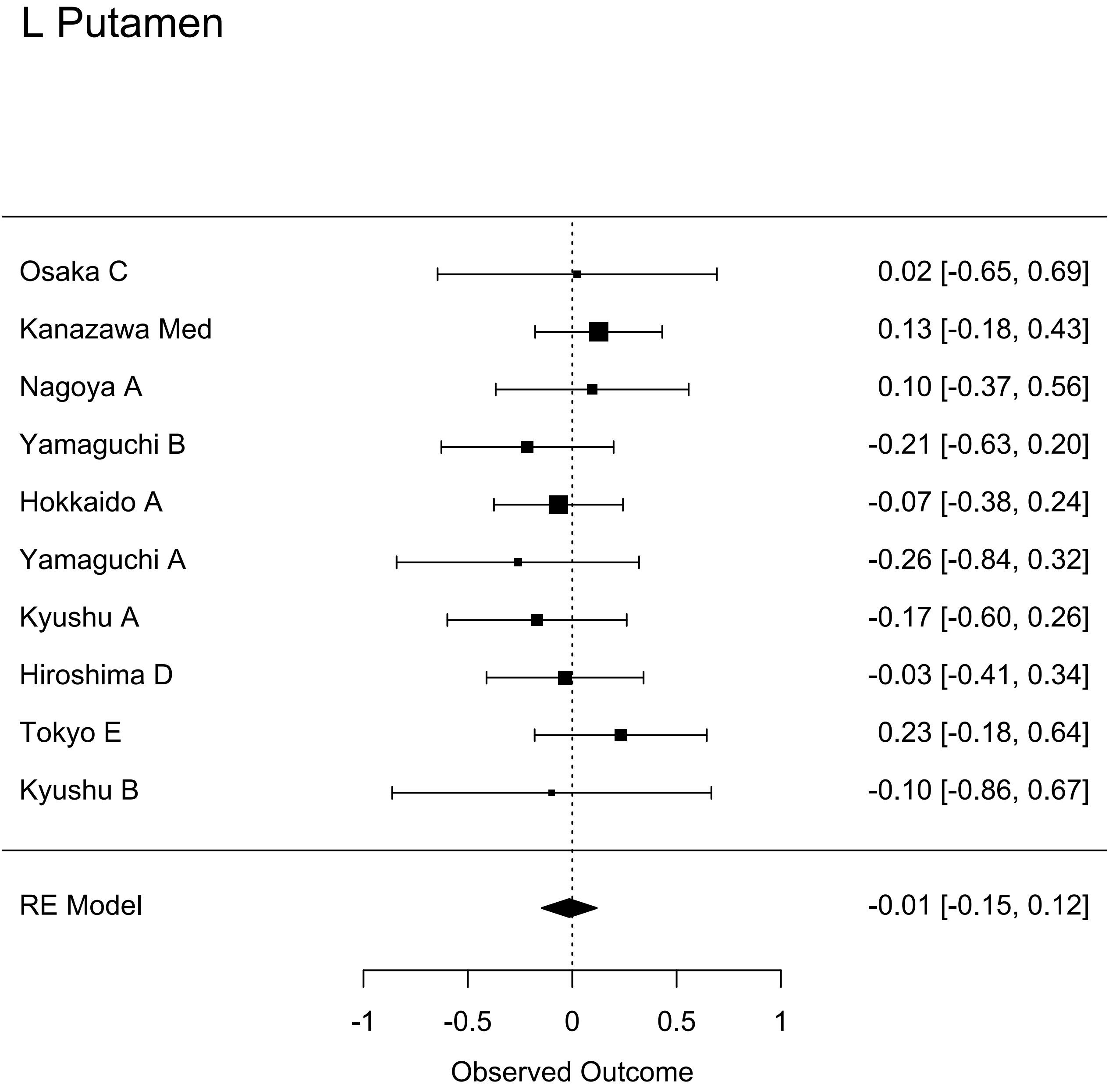
L Putamen
Osaka C
0.02 [-0.65, 0.69]
Kanazawa Med
0.13 [-0.18, 0.43]
Nagoya A
0.10 [-0.37, 0.56]
Yamaguchi B
-0.21 [-0.63, 0.20]
Hokkaido A
-0.07 [-0.38, 0.24]
Yamaguchi A
-0.26 [-0.84, 0.32]
Kyushu A
-0.17 [-0.60, 0.26]
Hiroshima D
-0.03 [-0.41, 0.34]
Tokyo E
0.23 [-0.18, 0.64]
Kyushu B
-0.10 [-0.86, 0.67]
RE Model
-0.01 [-0.15, 0.12]
-1
-0.5
0
0.5
1
Observed Outcome

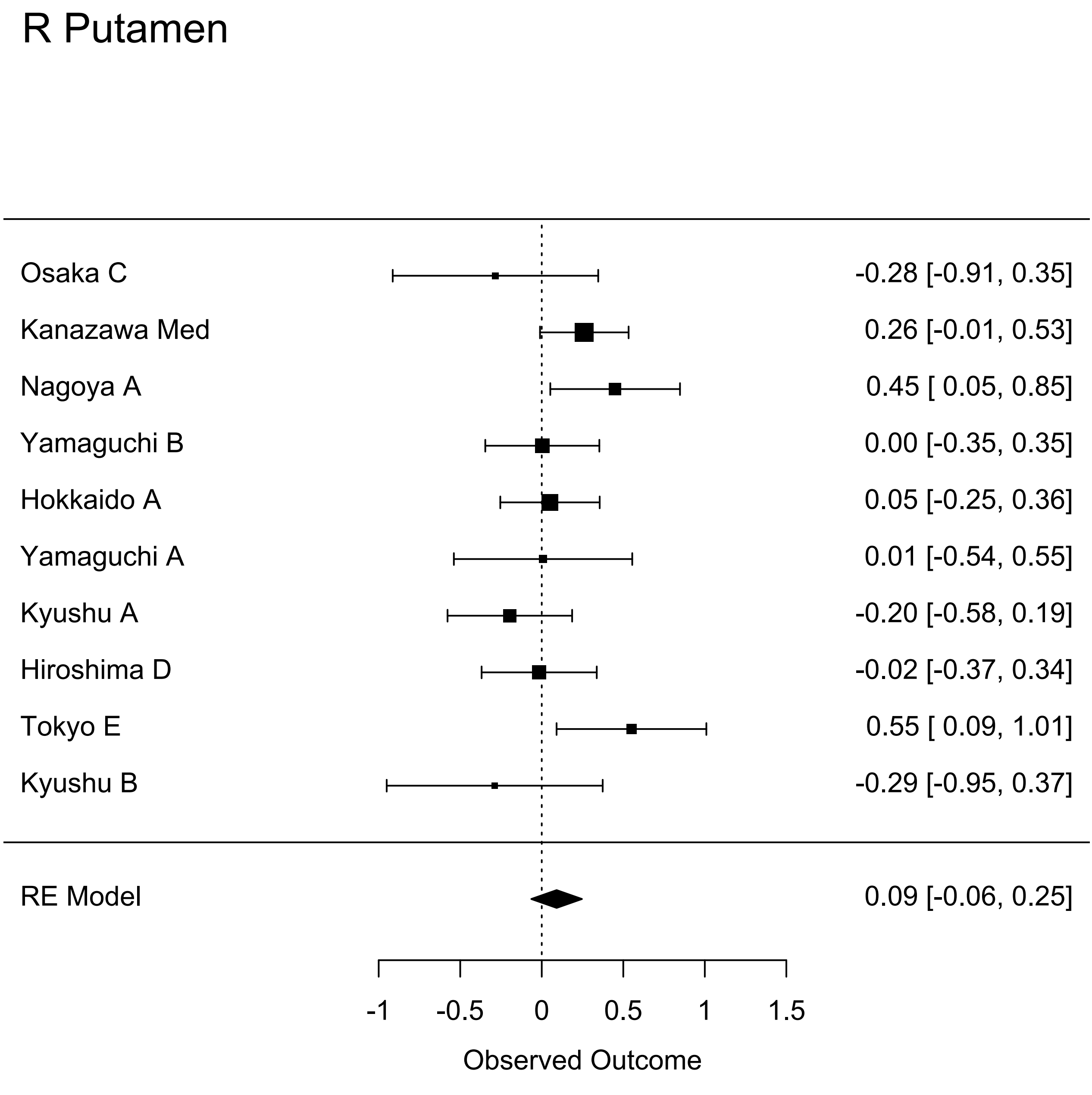
R Putamen
Osaka C
Kanazawa Med
Nagoya A
Yamaguchi B
Hokkaido A
Yamaguchi A
Kyushu A
Hiroshima D
Tokyo E
Kyushu B
RE Model
-0.28 [-0.91, 0.35]
0.26 [-0.01, 0.53]
0.45 [0.05, 0.85]
0.00 [-0.35, 0.35]
0.05 [-0.25, 0.36]
0.01 [-0.54, 0.55]
-0.20 [-0.58, 0.19]
-0.02 [-0.37, 0.34]
0.55 [0.09, 1.01]
-0.29 [-0.95, 0.37]
0.09 [-0.06, 0.25]
-1
-0.5
0
0.5
1
1.5
Observed Outcome

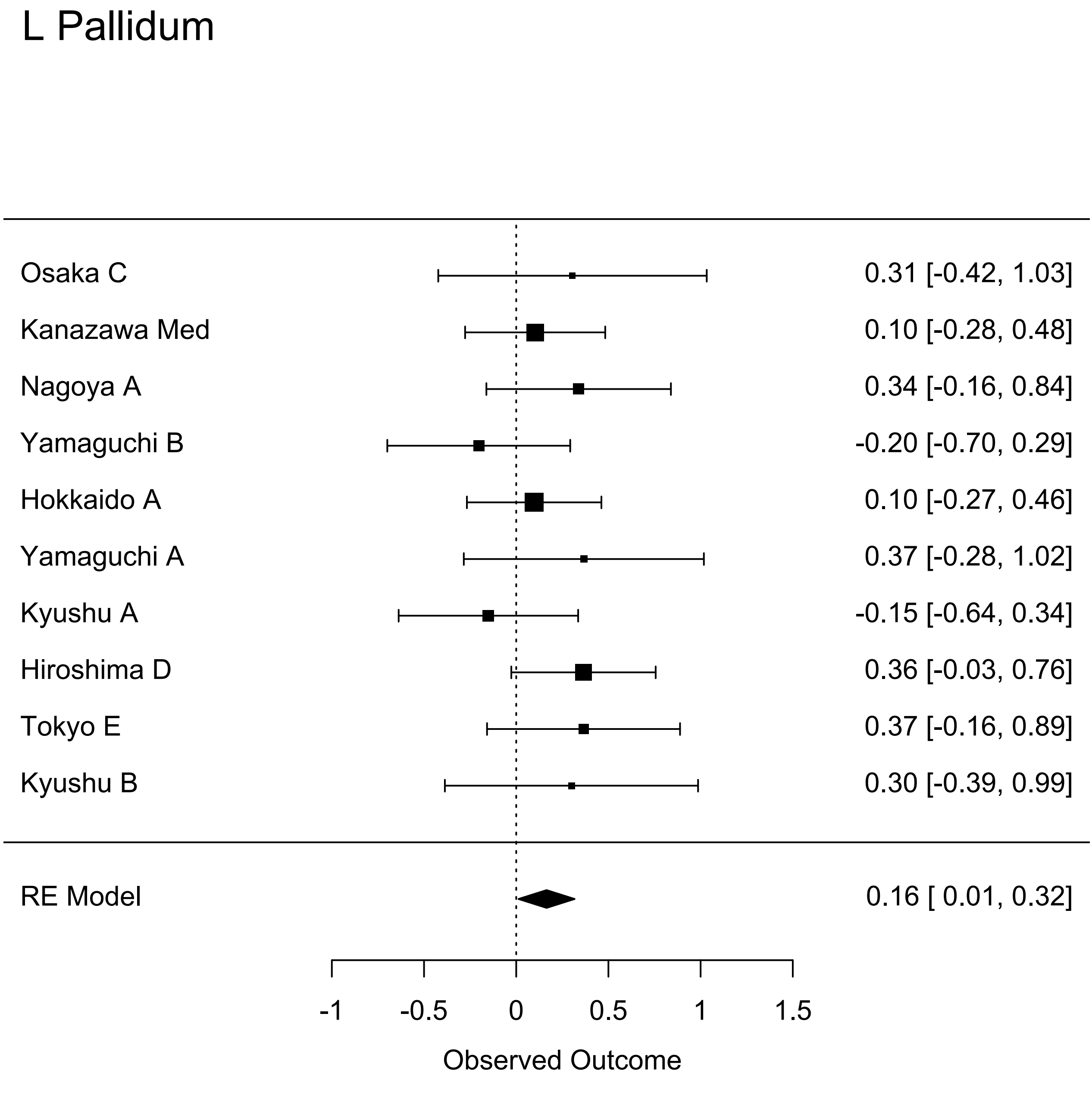

L Pallidum
Osaka C
Kanazawa Med
Nagoya A
Yamaguchi B
Hokkaido A
Yamaguchi A
Kyushu A
Hiroshima D
Tokyo E
Kyushu B
RE Model
0.31 [-0.42, 1.03]
0.10 [-0.28, 0.48]
0.34 [-0.16, 0.84]
-0.20 [-0.70, 0.29]
0.10 [-0.27, 0.46]
0.37 [-0.28, 1.02]
-0.15 [-0.64, 0.34]
0.36 [-0.03, 0.76]
0.37 [-0.16, 0.89]
0.30 [-0.39, 0.99]
0.16 [0.01, 0.32]
-1
-0.5
0
0.5
1
1.5
Observed Outcome

R Pallidum

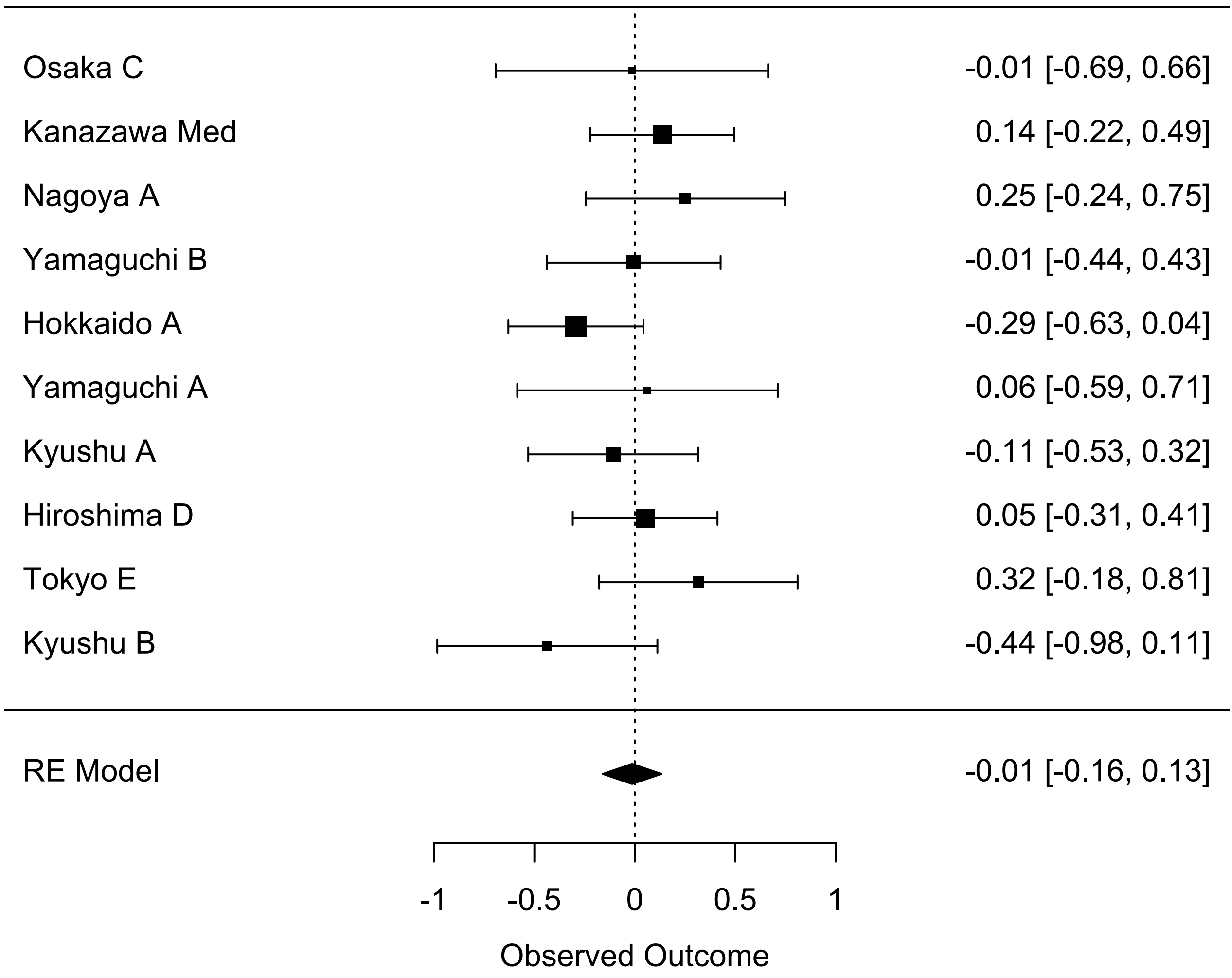

Osaka C
-0.01 [-0.69, 0.66]
Kanazawa Med
0.14 [-0.22, 0.49]
Nagoya A
0.25 [-0.24, 0.75]
Yamaguchi B
-0.01 [-0.44, 0.43]
Hokkaido A
-0.29 [-0.63, 0.04]
Yamaguchi A
0.06 [-0.59, 0.71]
Kyushu A
-0.11 [-0.53, 0.32]
Hiroshima D
0.05 [-0.31, 0.41]
Tokyo E
0.32 [-0.18, 0.81]
Kyushu B
-0.44 [-0.98, 0.11]
RE Model
-0.01 [-0.16, 0.13]
-1
-0.5
0
0.5
1
Observed Outcome

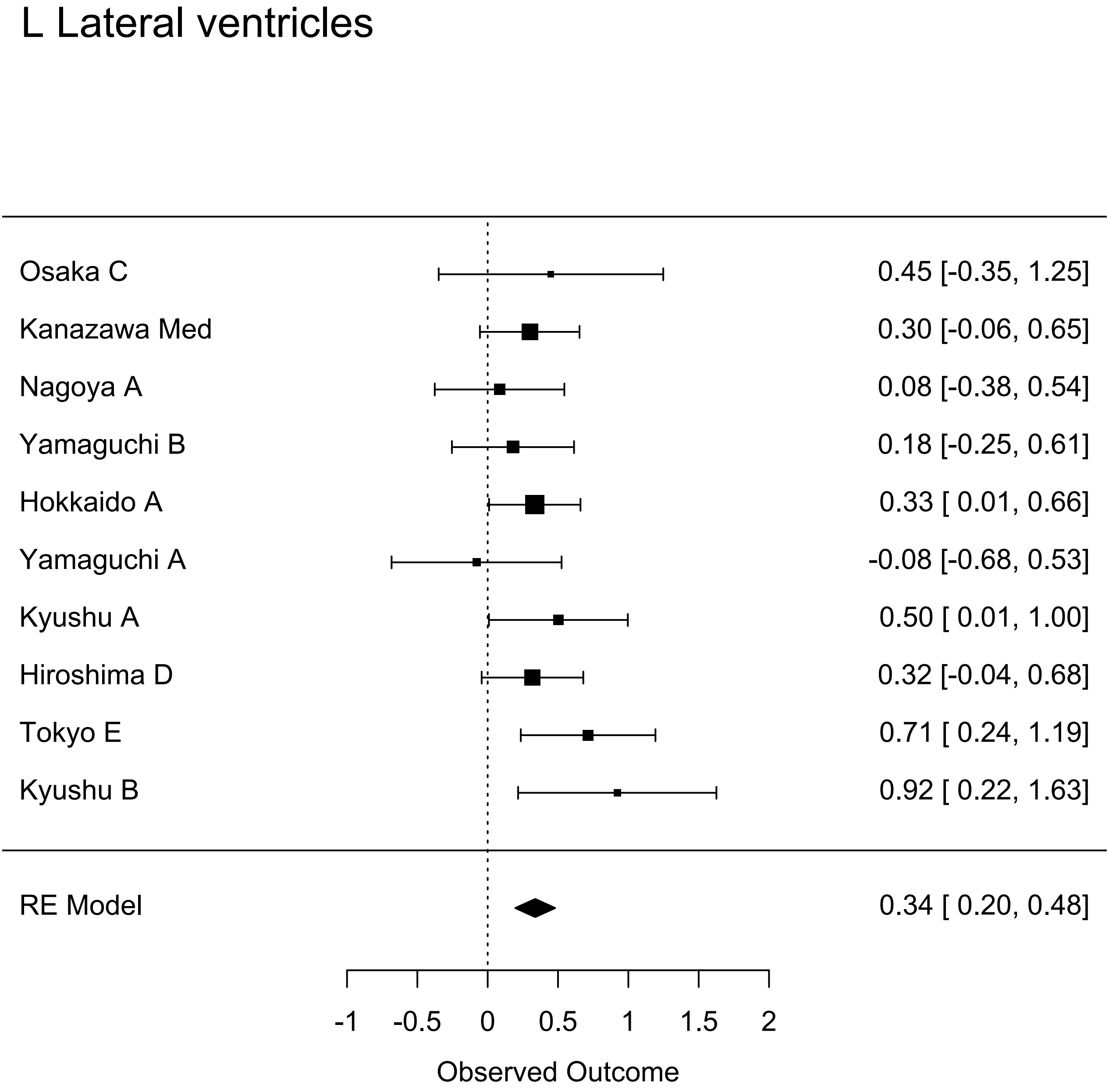

L Lateral ventricles
Osaka C 0.45 [-0.35, 1.25]
Kanazawa Med 0.30 [-0.06, 0.65]
Nagoya A 0.08 [-0.38, 0.54]
Yamaguchi B 0.18 [-0.25, 0.61]
Hokkaido A 0.33 [0.01, 0.66]
Yamaguchi A -0.08 [-0.68, 0.53]
Kyushu A 0.50 [0.01, 1.00]
Hiroshima D 0.32 [-0.04, 0.68]
Tokyo E 0.71 [0.24, 1.19]
Kyushu B 0.92 [0.22, 1.63]
RE Model 0.34 [0.20, 0.48]
-1
-0.5
0
0.5
1
1.5
2
Observed Outcome

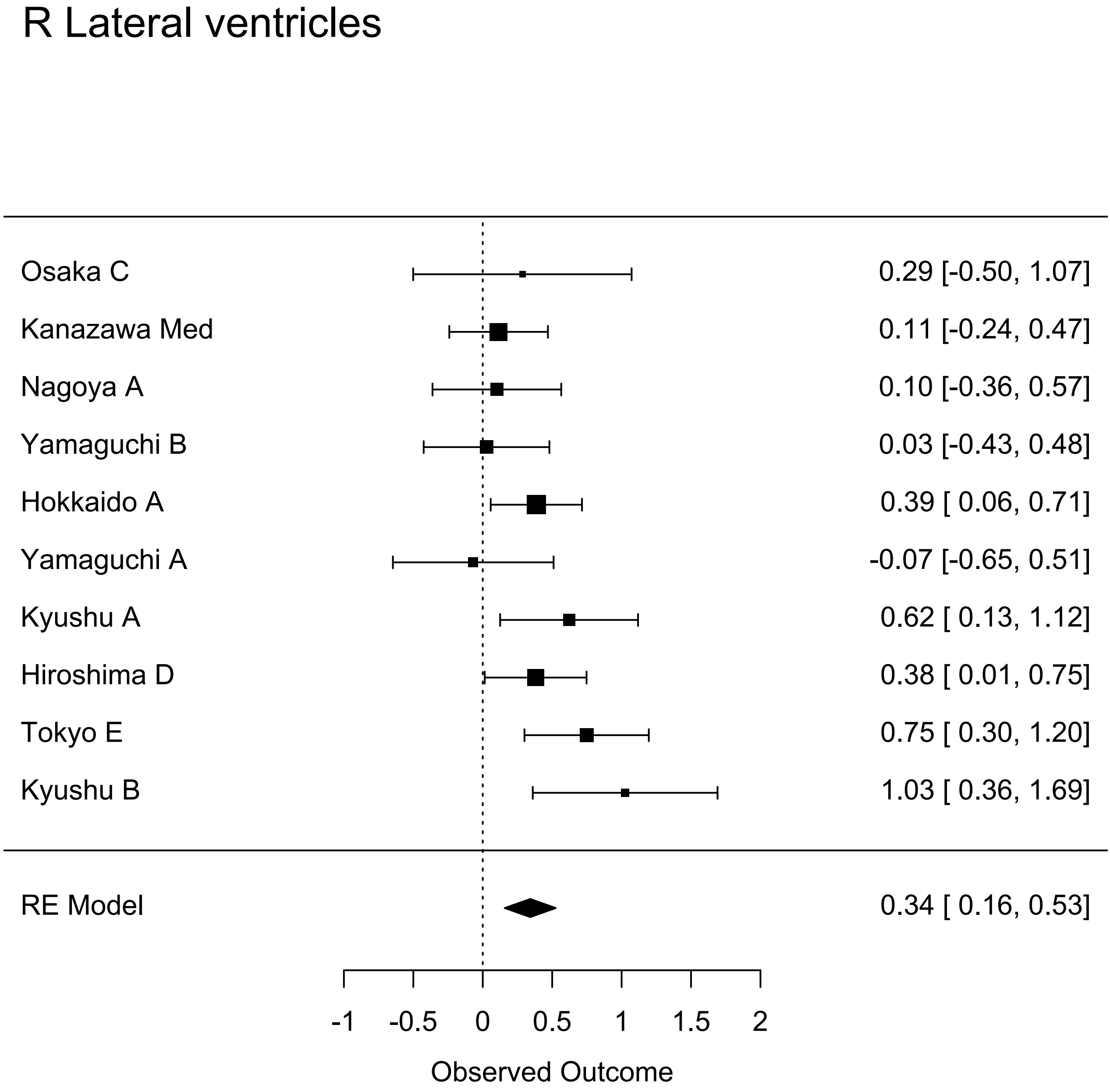
R Lateral ventricles
Osaka C 0.29 [-0.50, 1.07]
Kanazawa Med 0.11 [-0.24, 0.47]
Nagoya A 0.10 [-0.36, 0.57]
Yamaguchi B 0.03 [-0.43, 0.48]
Hokkaido A 0.39 [0.06, 0.71]
Yamaguchi A -0.07 [-0.65, 0.51]
Kyushu A 0.62 [0.13, 1.12]
Hiroshima D 0.38 [0.01, 0.75]
Tokyo E 0.75 [0.30, 1.20]
Kyushu B 1.03 [0.36, 1.69]
RE Model 0.34 [0.16, 0.53]
-1
-0.5
0
0.5
1
1.5
2
Observed Outcome

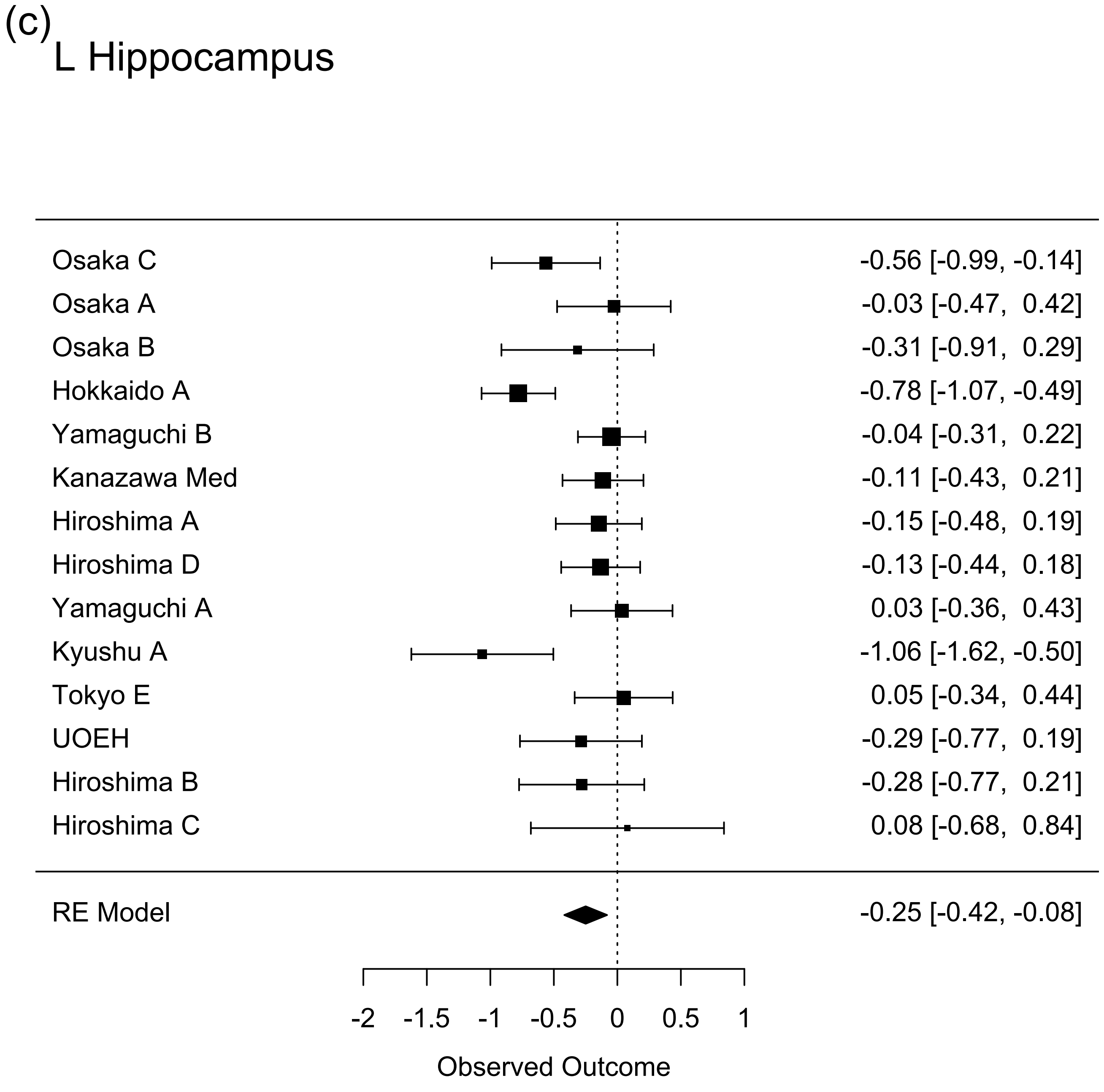
(c)
L Hippocampus
Osaka C
Osaka A
Osaka B
Hokkaido A
Yamaguchi B
Kanazawa Med
Hiroshima A
Hiroshima D
Yamaguchi A
Kyushu A
Tokyo E
UOEH
Hiroshima B
Hiroshima C
RE Model
-0.56 [-0.99, -0.14]
-0.03 [-0.47, 0.42]
-0.31 [-0.91, 0.29]
-0.78 [-1.07, -0.49]
-0.04 [-0.31, 0.22]
-0.11 [-0.43, 0.21]
-0.15 [-0.48, 0.19]
-0.13 [-0.44, 0.18]
0.03 [-0.36, 0.43]
-1.06 [-1.62, -0.50]
0.05 [-0.34, 0.44]
-0.29 [-0.77, 0.19]
-0.28 [-0.77, 0.21]
0.08 [-0.68, 0.84]
-0.25 [-0.42, -0.08]
-2
-1.5
-1
-0.5
0
0.5
1
Observed Outcome

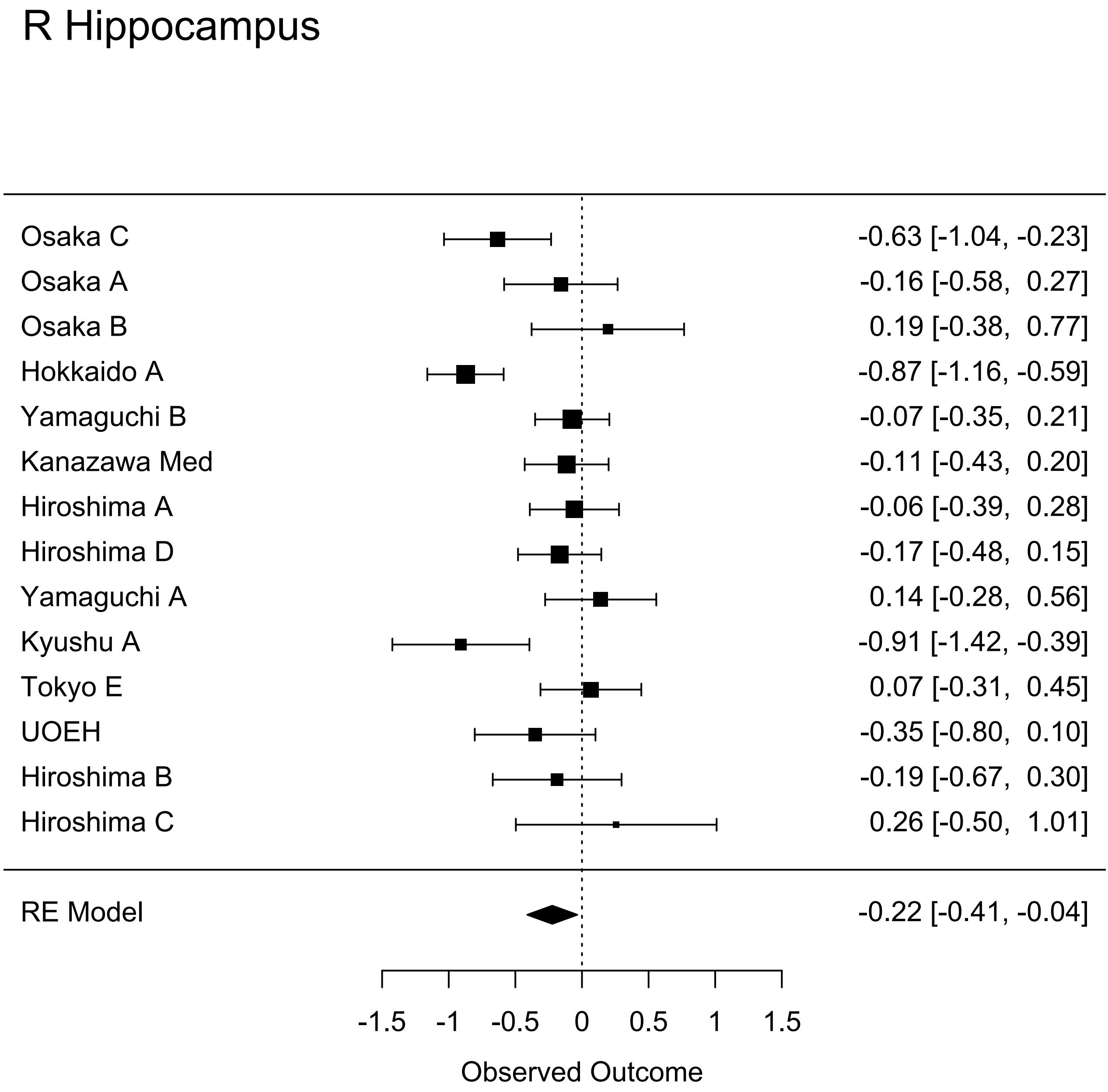
R Hippocampus
Osaka C
Osaka A
Osaka B
Hokkaido A
Yamaguchi B
Kanazawa Med
Hiroshima A
Hiroshima D
Yamaguchi A
Kyushu A
Tokyo E
UOEH
Hiroshima B
Hiroshima C
RE Model
-0.63 [-1.04, -0.23]
-0.16 [-0.58, 0.27]
0.19 [-0.38, 0.77]
-0.87 [-1.16, -0.59]
-0.07 [-0.35, 0.21]
-0.11 [-0.43, 0.20]
-0.06 [-0.39, 0.28]
-0.17 [-0.48, 0.15]
0.14 [-0.28, 0.56]
-0.91 [-1.42, -0.39]
0.07 [-0.31, 0.45]
-0.35 [-0.80, 0.10]
-0.19 [-0.67, 0.30]
0.26 [-0.50, 1.01]
-0.22 [-0.41, -0.04]
-1.5
-1
-0.5
0
0.5
1
1.5
Observed Outcome

L Amygdala

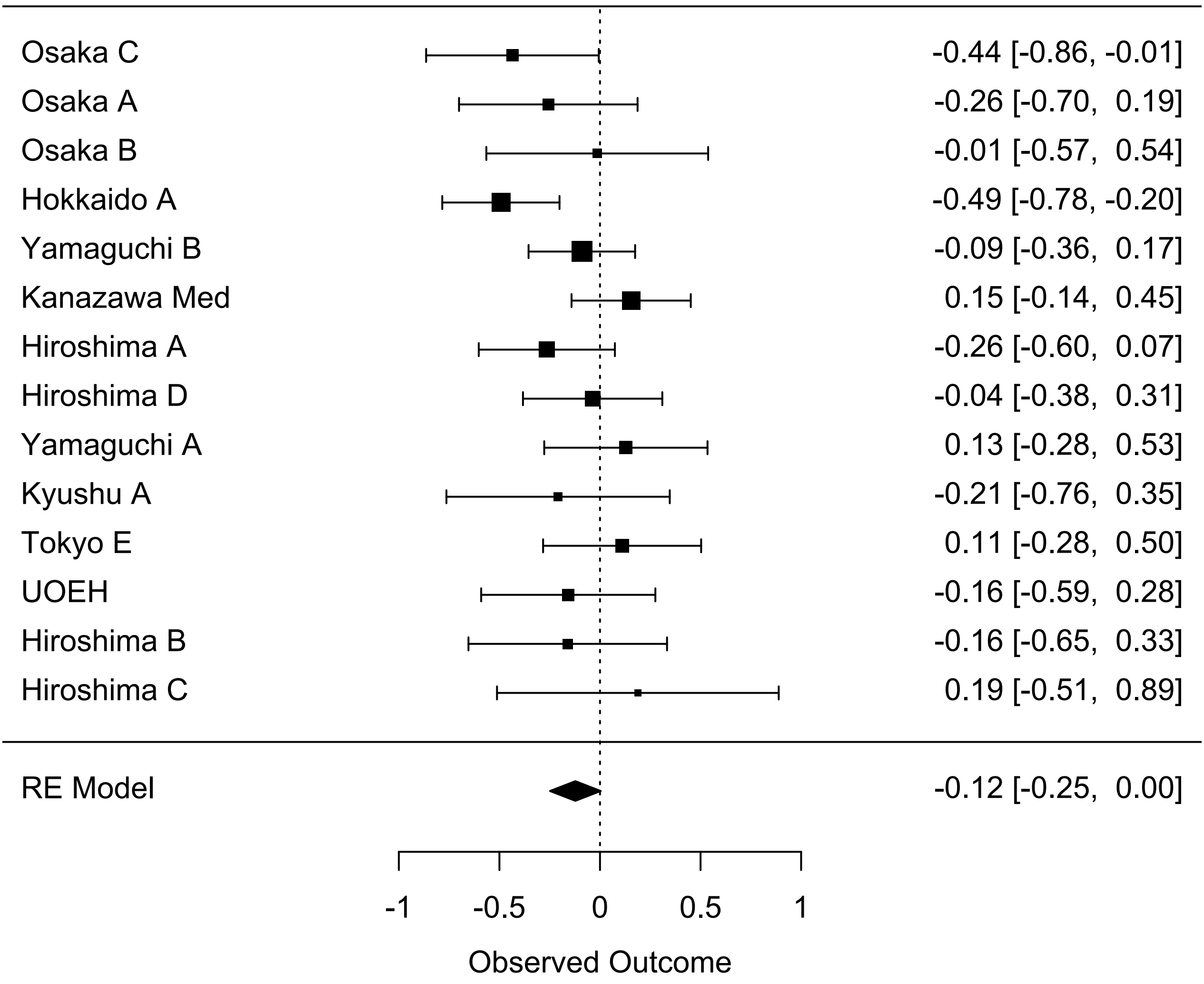

Osaka C -0.44 [-0.86, -0.01]
Osaka A -0.26 [-0.70, 0.19]
Osaka B -0.01 [-0.57, 0.54]
Hokkaido A -0.49 [-0.78, -0.20]
Yamaguchi B -0.09 [-0.36, 0.17]
Kanazawa Med 0.15 [-0.14, 0.45]
Hiroshima A -0.26 [-0.60, 0.07]
Hiroshima D -0.04 [-0.38, 0.31]
Yamaguchi A 0.13 [-0.28, 0.53]
Kyushu A -0.21 [-0.76, 0.35]
Tokyo E 0.11 [-0.28, 0.50]
UOEH -0.16 [-0.59, 0.28]
Hiroshima B -0.16 [-0.65, 0.33]
Hiroshima C 0.19 [-0.51, 0.89]
RE Model -0.12 [-0.25, 0.00]
-1
-0.5
0
0.5
1
Observed Outcome

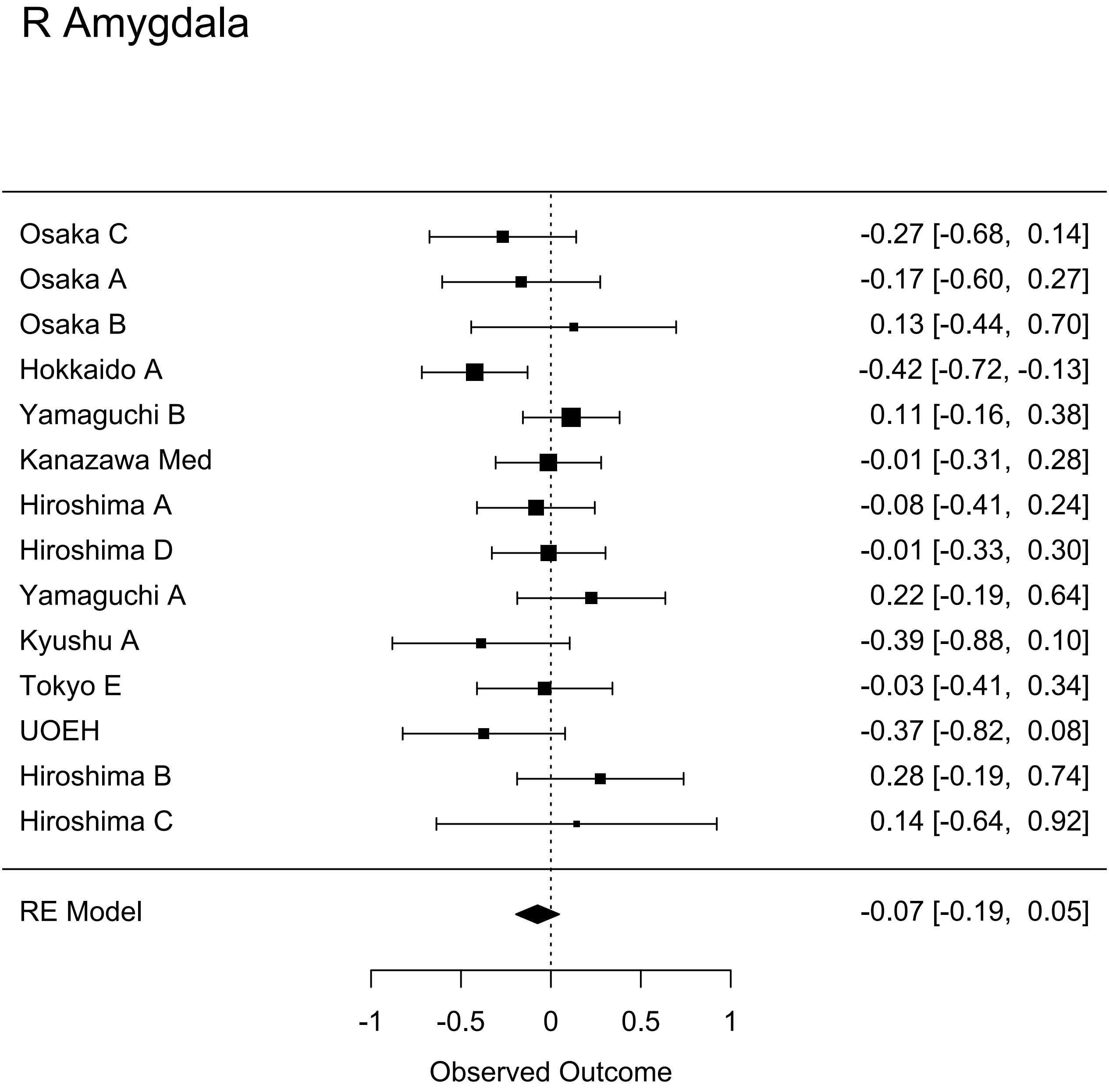
R Amygdala
Osaka C -0.27 [-0.68, 0.14]
Osaka A -0.17 [-0.60, 0.27]
Osaka B 0.13 [-0.44, 0.70]
Hokkaido A -0.42 [-0.72, -0.13]
Yamaguchi B 0.11 [-0.16, 0.38]
Kanazawa Med -0.01 [-0.31, 0.28]
Hiroshima A -0.08 [-0.41, 0.24]
Hiroshima D -0.01 [-0.33, 0.30]
Yamaguchi A 0.22 [-0.19, 0.64]
Kyushu A -0.39 [-0.88, 0.10]
Tokyo E -0.03 [-0.41, 0.34]
UOEH -0.37 [-0.82, 0.08]
Hiroshima B 0.28 [-0.19, 0.74]
Hiroshima C 0.14 [-0.64, 0.92]
RE Model -0.07 [-0.19, 0.05]
-1
-0.5
0
0.5
1
Observed Outcome

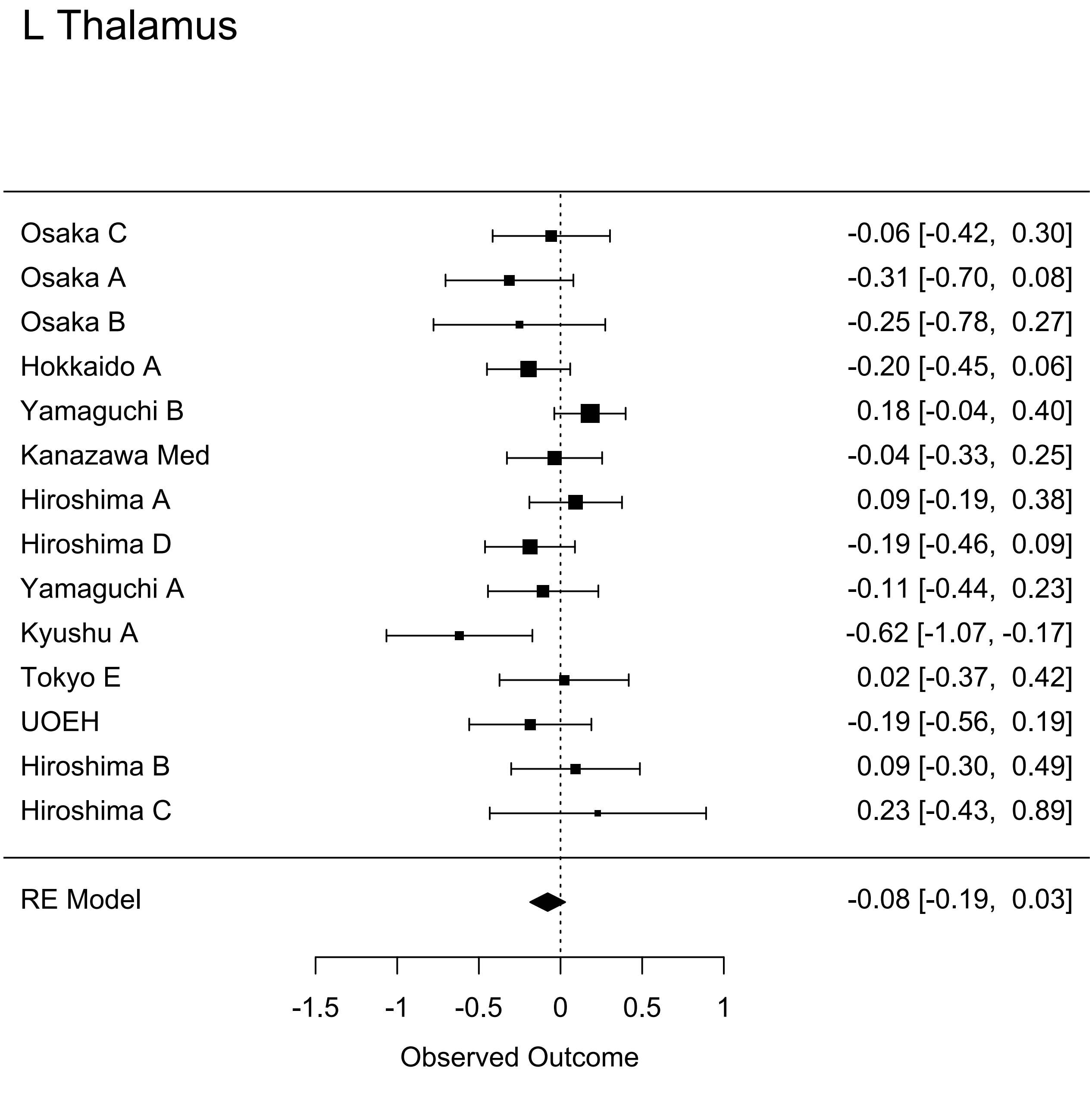
L Thalamus
Osaka C -0.06 [-0.42, 0.30]
Osaka A -0.31 [-0.70, 0.08]
Osaka B -0.25 [-0.78, 0.27]
Hokkaido A -0.20 [-0.45, 0.06]
Yamaguchi B 0.18 [-0.04, 0.40]
Kanazawa Med -0.04 [-0.33, 0.25]
Hiroshima A 0.09 [-0.19, 0.38]
Hiroshima D -0.19 [-0.46, 0.09]
Yamaguchi A -0.11 [-0.44, 0.23]
Kyushu A -0.62 [-1.07, -0.17]
Tokyo E 0.02 [-0.37, 0.42]
UOEH -0.19 [-0.56, 0.19]
Hiroshima B 0.09 [-0.30, 0.49]
Hiroshima C 0.23 [-0.43, 0.89]
RE Model -0.08 [-0.19, 0.03]
-1.5
-1
-0.5
0
0.5
1
Observed Outcome

R Thalamus

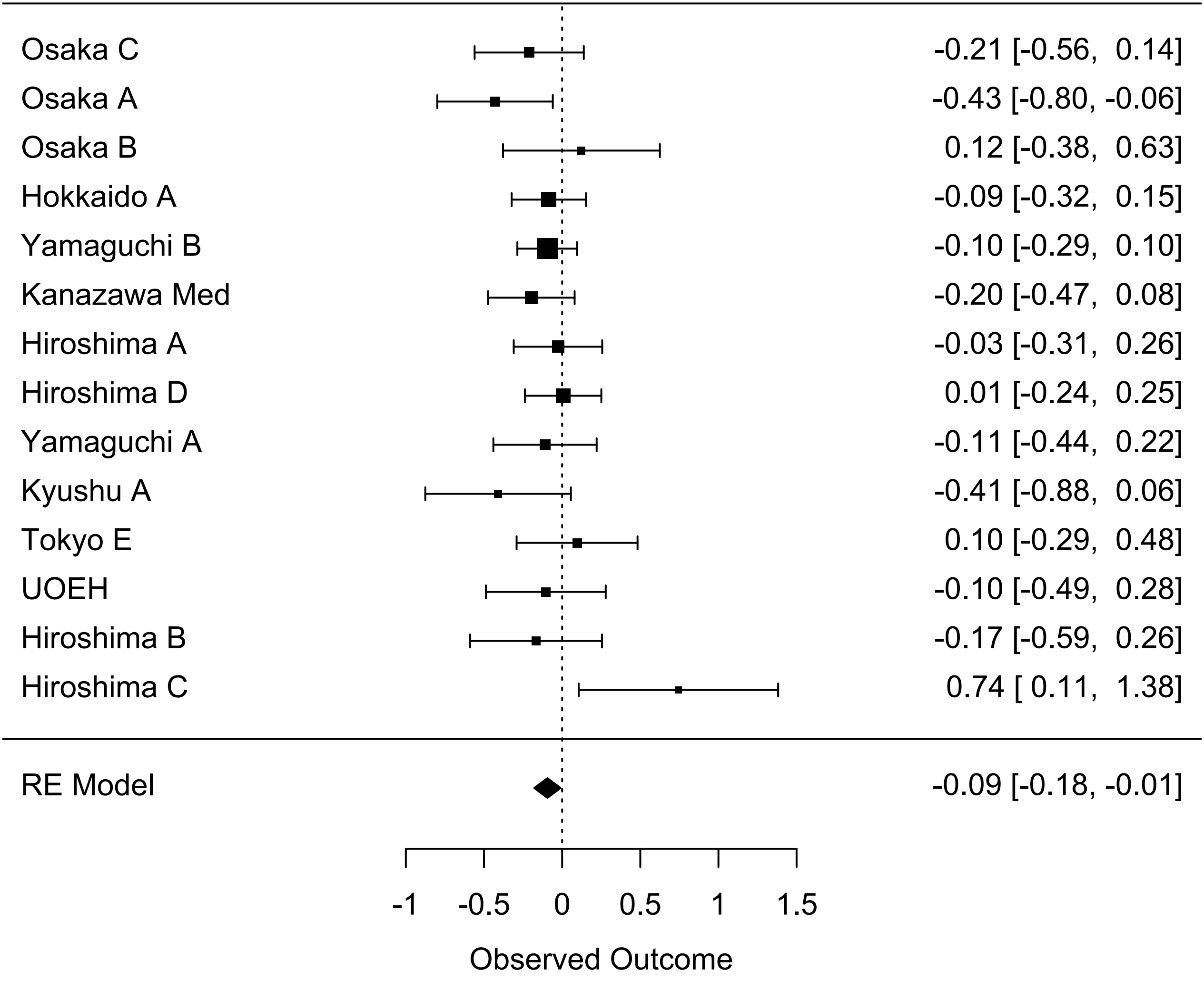

Osaka C
Osaka A
Osaka B
Hokkaido A
Yamaguchi B
Kanazawa Med
Hiroshima A
Hiroshima D
Yamaguchi A
Kyushu A
Tokyo E
UOEH
Hiroshima B
Hiroshima C
RE Model
-0.21 [-0.56, 0.14]
-0.43 [-0.80, -0.06]
0.12 [-0.38, 0.63]
-0.09 [-0.32, 0.15]
-0.10 [-0.29, 0.10]
-0.20 [-0.47, 0.08]
-0.03 [-0.31, 0.26]
0.01 [-0.24, 0.25]
-0.11 [-0.44, 0.22]
-0.41 [-0.88, 0.06]
0.10 [-0.29, 0.48]
-0.10 [-0.49, 0.28]
-0.17 [-0.59, 0.26]
0.74 [0.11, 1.38]
-0.09 [-0.18, -0.01]
-1
-0.5
0
0.5
1
1.5
Observed Outcome

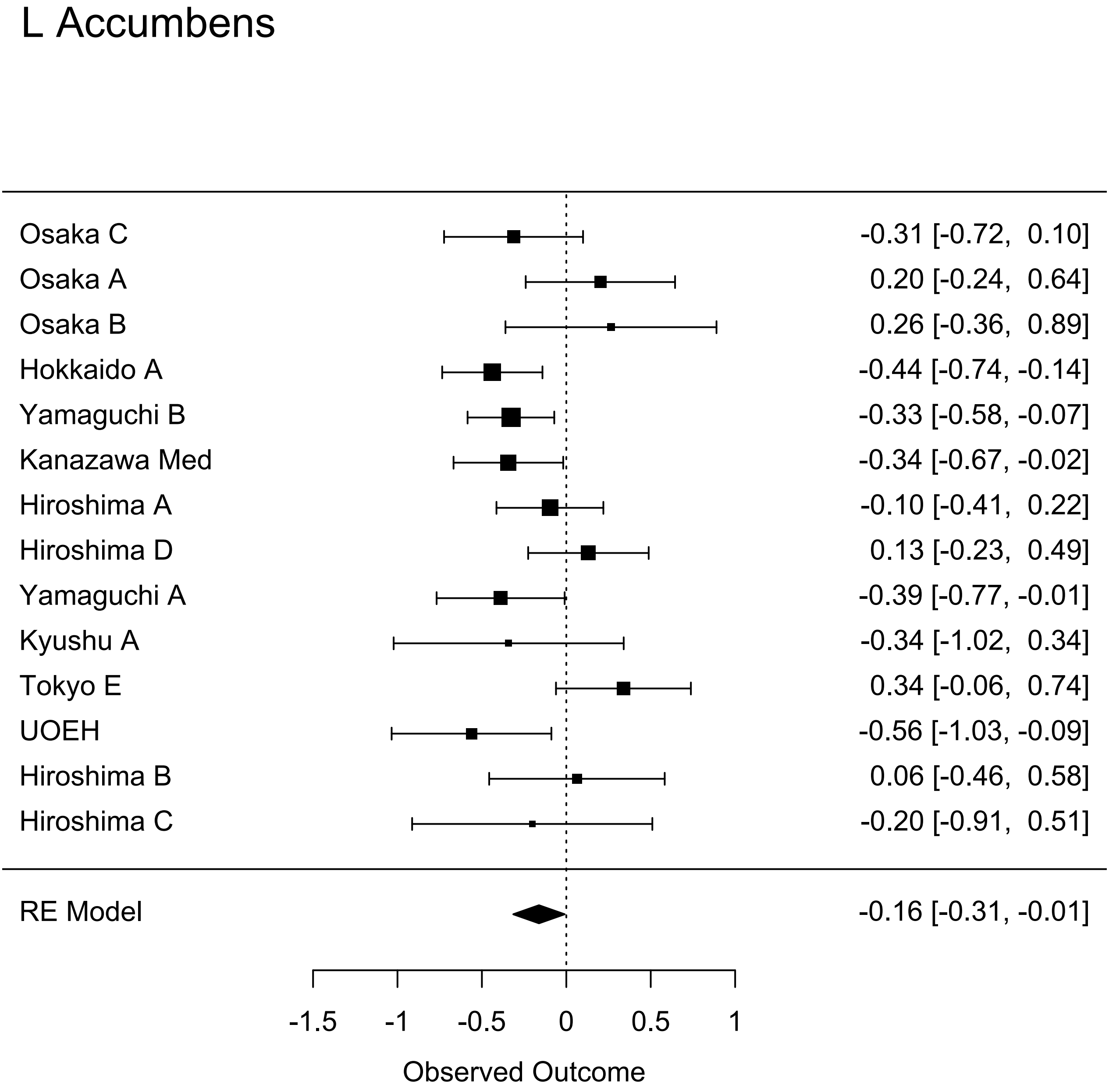
L Accumbens
Osaka C
Osaka A
Osaka B
Hokkaido A
Yamaguchi B
Kanazawa Med
Hiroshima A
Hiroshima D
Yamaguchi A
Kyushu A
Tokyo E
UOEH
Hiroshima B
Hiroshima C
RE Model
-0.31 [-0.72, 0.10]
0.20 [-0.24, 0.64]
0.26 [-0.36, 0.89]
-0.44 [-0.74, -0.14]
-0.33 [-0.58, -0.07]
-0.34 [-0.67, -0.02]
-0.10 [-0.41, 0.22]
0.13 [-0.23, 0.49]
-0.39 [-0.77, -0.01]
-0.34 [-1.02, 0.34]
0.34 [-0.06, 0.74]
-0.56 [-1.03, -0.09]
0.06 [-0.46, 0.58]
-0.20 [-0.91, 0.51]
-0.16 [-0.31, -0.01]
-1.5
-1
-0.5
0
0.5
1
Observed Outcome

R Accumbens

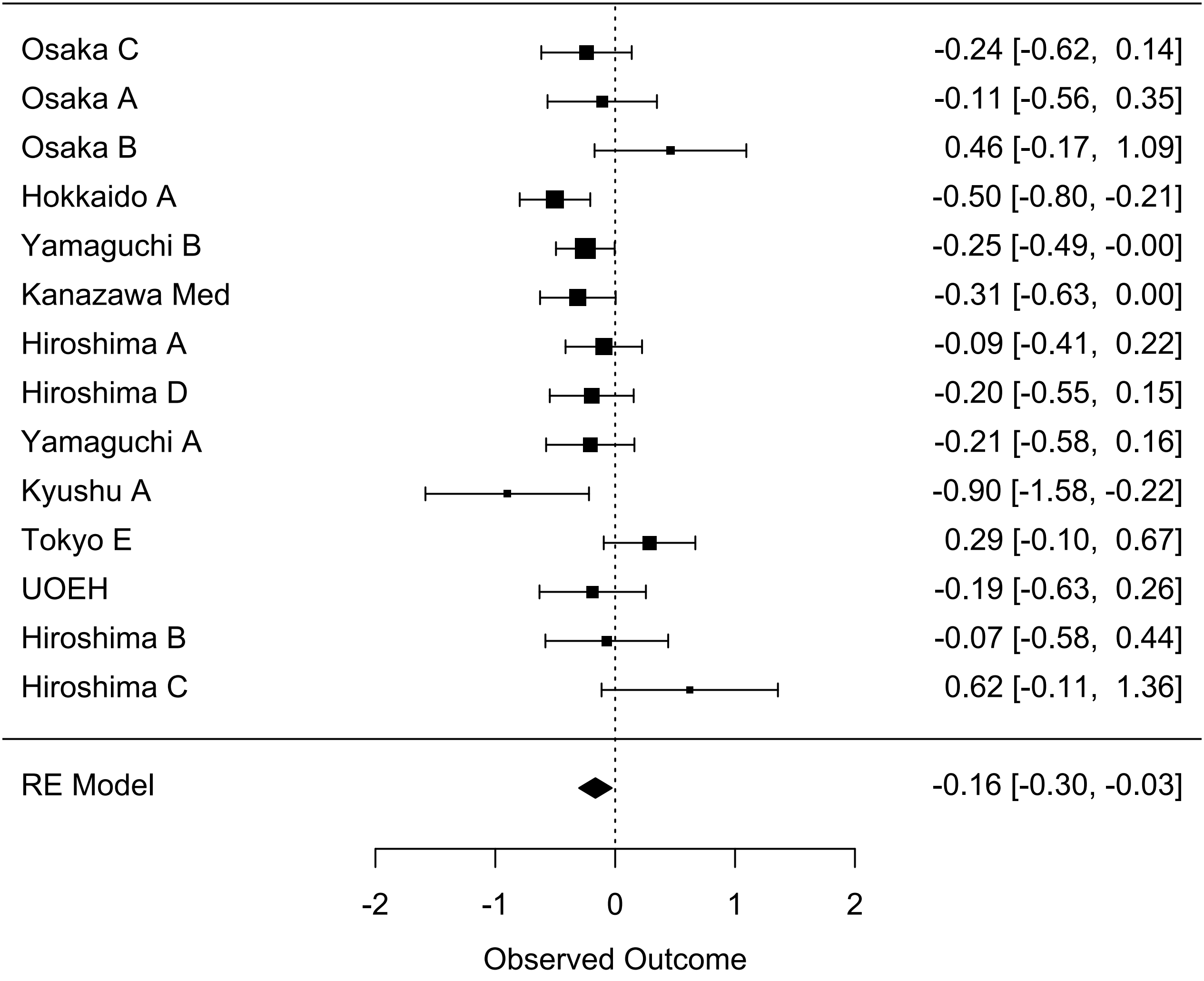

Osaka C
-0.24 [-0.62, 0.14]
Osaka A
-0.11 [-0.56, 0.35]
Osaka B
0.46 [-0.17, 1.09]
Hokkaido A
-0.50 [-0.80, -0.21]
Yamaguchi B
-0.25 [-0.49, -0.00]
Kanazawa Med
-0.31 [-0.63, 0.00]
Hiroshima A
-0.09 [-0.41, 0.22]
Hiroshima D
-0.20 [-0.55, 0.15]
Yamaguchi A
-0.21 [-0.58, 0.16]
Kyushu A
-0.90 [-1.58, -0.22]
Tokyo E
0.29 [-0.10, 0.67]
UOEH
-0.19 [-0.63, 0.26]
Hiroshima B
-0.07 [-0.58, 0.44]
Hiroshima C
0.62 [-0.11, 1.36]
RE Model
-0.16 [-0.30, -0.03]
-2
-1
0
1
2
Observed Outcome

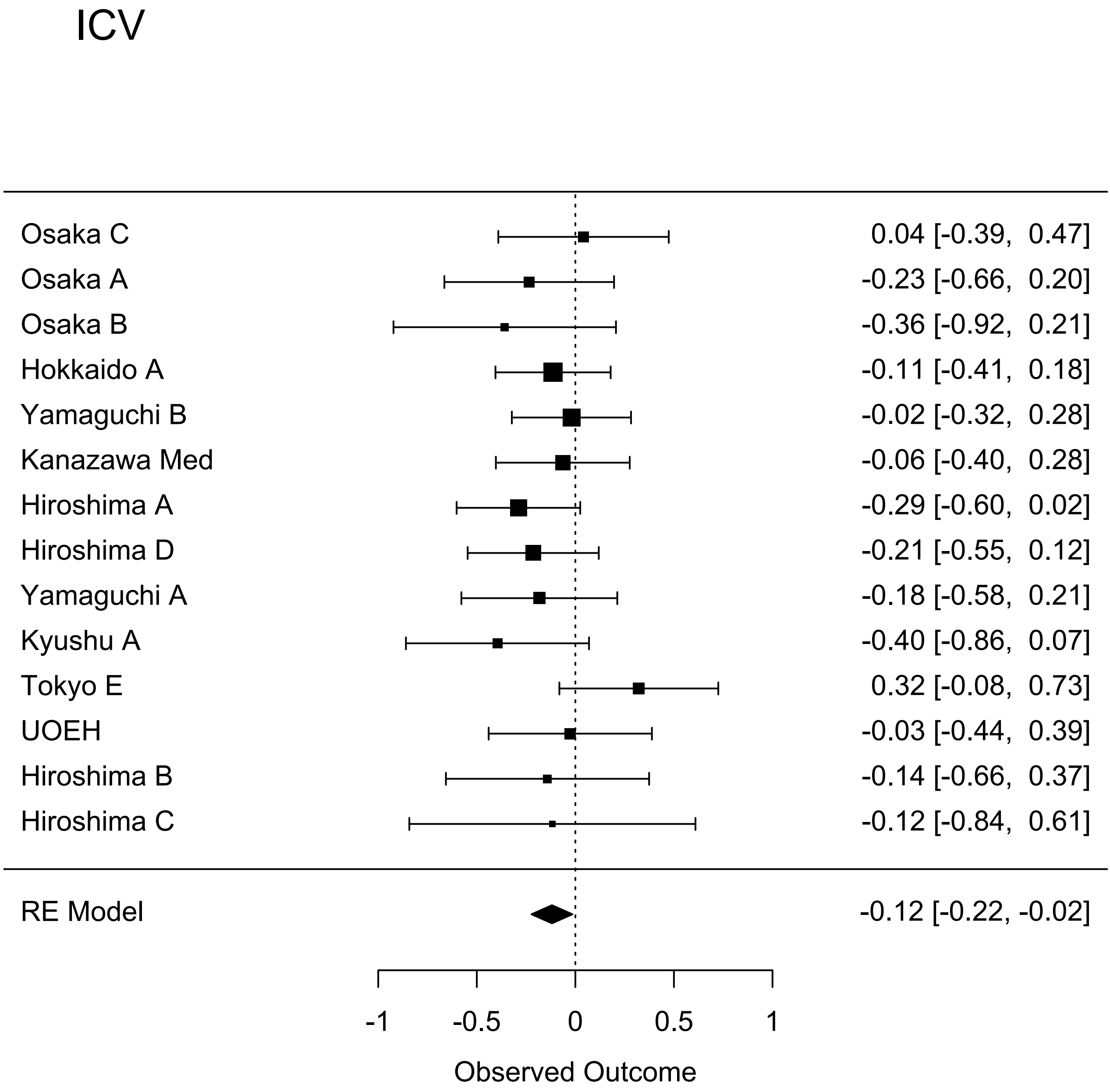
ICV
Osaka C
Osaka A
Osaka B
Hokkaido A
Yamaguchi B
Kanazawa Med
Hiroshima A
Hiroshima D
Yamaguchi A
Kyushu A
Tokyo E
UOEH
Hiroshima B
Hiroshima C
RE Model
0.04 [-0.39, 0.47]
-0.23 [-0.66, 0.20]
-0.36 [-0.92, 0.21]
-0.11 [-0.41, 0.18]
-0.02 [-0.32, 0.28]
-0.06 [-0.40, 0.28]
-0.29 [-0.60, 0.02]
-0.21 [-0.55, 0.12]
-0.18 [-0.58, 0.21]
-0.40 [-0.86, 0.07]
0.32 [-0.08, 0.73]
-0.03 [-0.44, 0.39]
-0.14 [-0.66, 0.37]
-0.12 [-0.84, 0.61]
-0.12 [-0.22, -0.02]
-1
-0.5
0
0.5
1
Observed Outcome

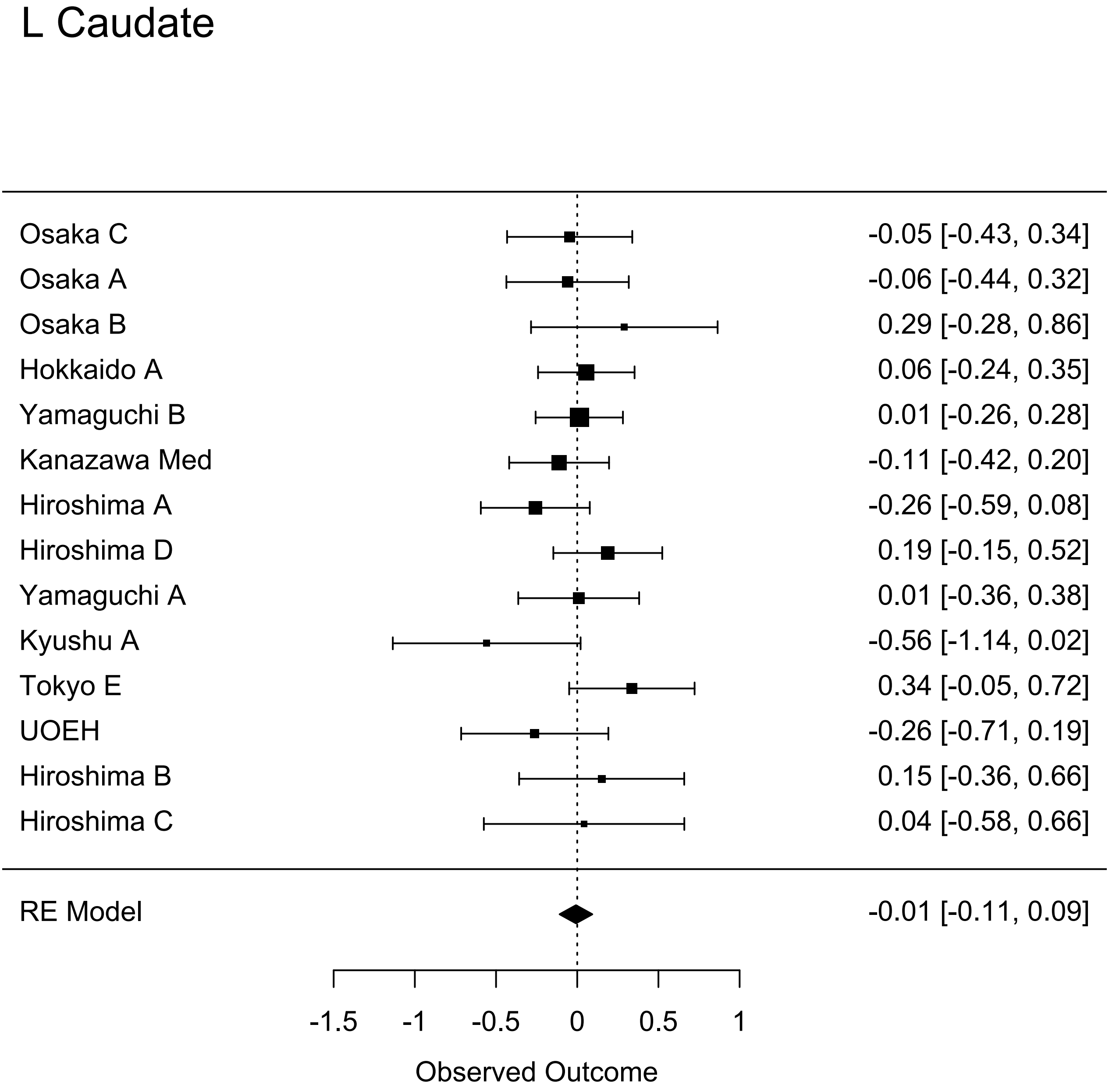
L Caudate
Osaka C -0.05 [-0.43, 0.34]
Osaka A -0.06 [-0.44, 0.32]
Osaka B 0.29 [-0.28, 0.86]
Hokkaido A 0.06 [-0.24, 0.35]
Yamaguchi B 0.01 [-0.26, 0.28]
Kanazawa Med -0.11 [-0.42, 0.20]
Hiroshima A -0.26 [-0.59, 0.08]
Hiroshima D 0.19 [-0.15, 0.52]
Yamaguchi A 0.01 [-0.36, 0.38]
Kyushu A -0.56 [-1.14, 0.02]
Tokyo E 0.34 [-0.05, 0.72]
UOEH -0.26 [-0.71, 0.19]
Hiroshima B 0.15 [-0.36, 0.66]
Hiroshima C 0.04 [-0.58, 0.66]
RE Model -0.01 [-0.11, 0.09]
-1.5
-1
-0.5
0
0.5
1
Observed Outcome

R Caudate

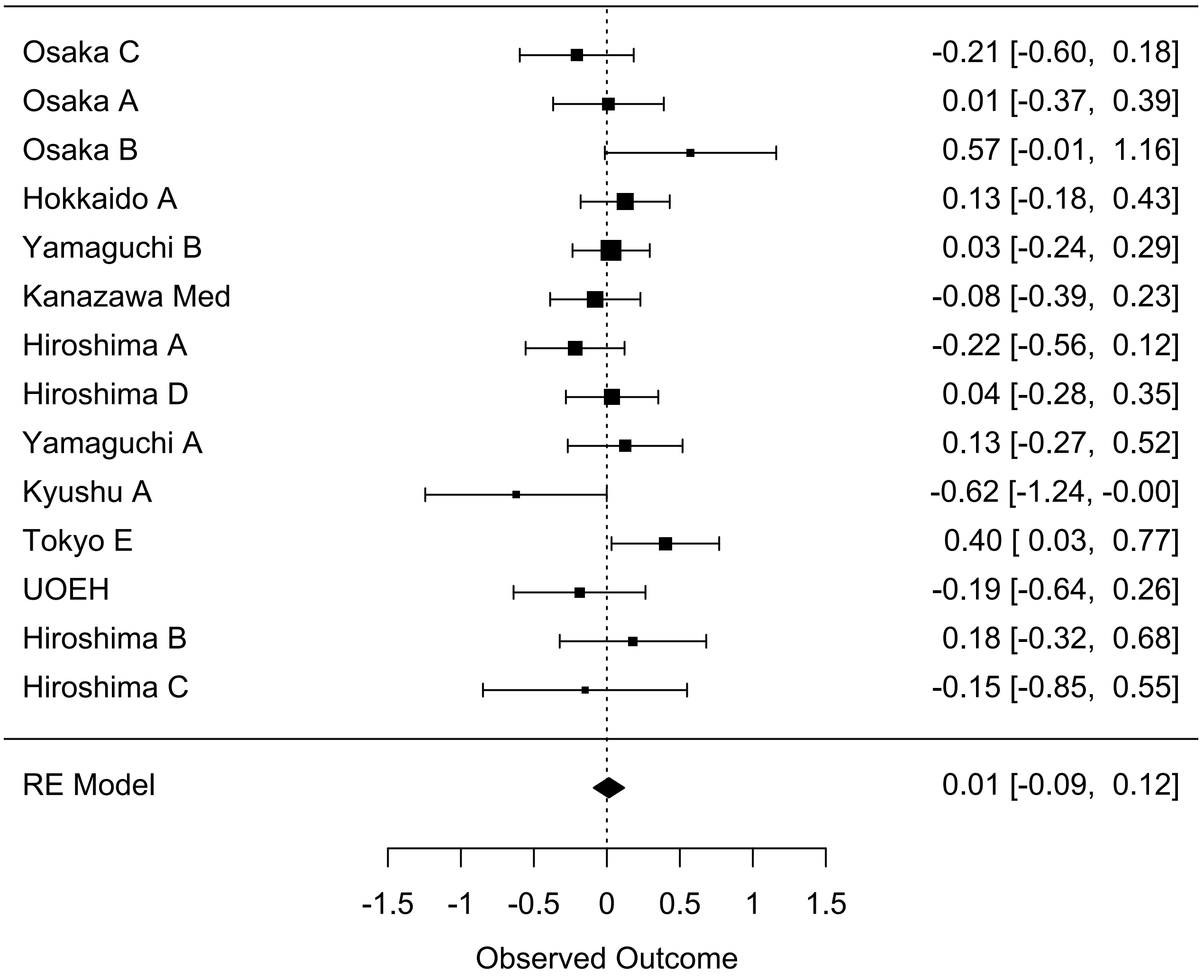

Osaka C -0.21 [-0.60, 0.18]
Osaka A 0.01 [-0.37, 0.39]
Osaka B 0.57 [-0.01, 1.16]
Hokkaido A 0.13 [-0.18, 0.43]
Yamaguchi B 0.03 [-0.24, 0.29]
Kanazawa Med -0.08 [-0.39, 0.23]
Hiroshima A -0.22 [-0.56, 0.12]
Hiroshima D 0.04 [-0.28, 0.35]
Yamaguchi A 0.13 [-0.27, 0.52]
Kyushu A -0.62 [-1.24, -0.00]
Tokyo E 0.40 [0.03, 0.77]
UOEH -0.19 [-0.64, 0.26]
Hiroshima B 0.18 [-0.32, 0.68]
Hiroshima C -0.15 [-0.85, 0.55]
RE Model 0.01 [-0.09, 0.12]
-1.5
-1
-0.5
0
0.5
1
1.5
Observed Outcome

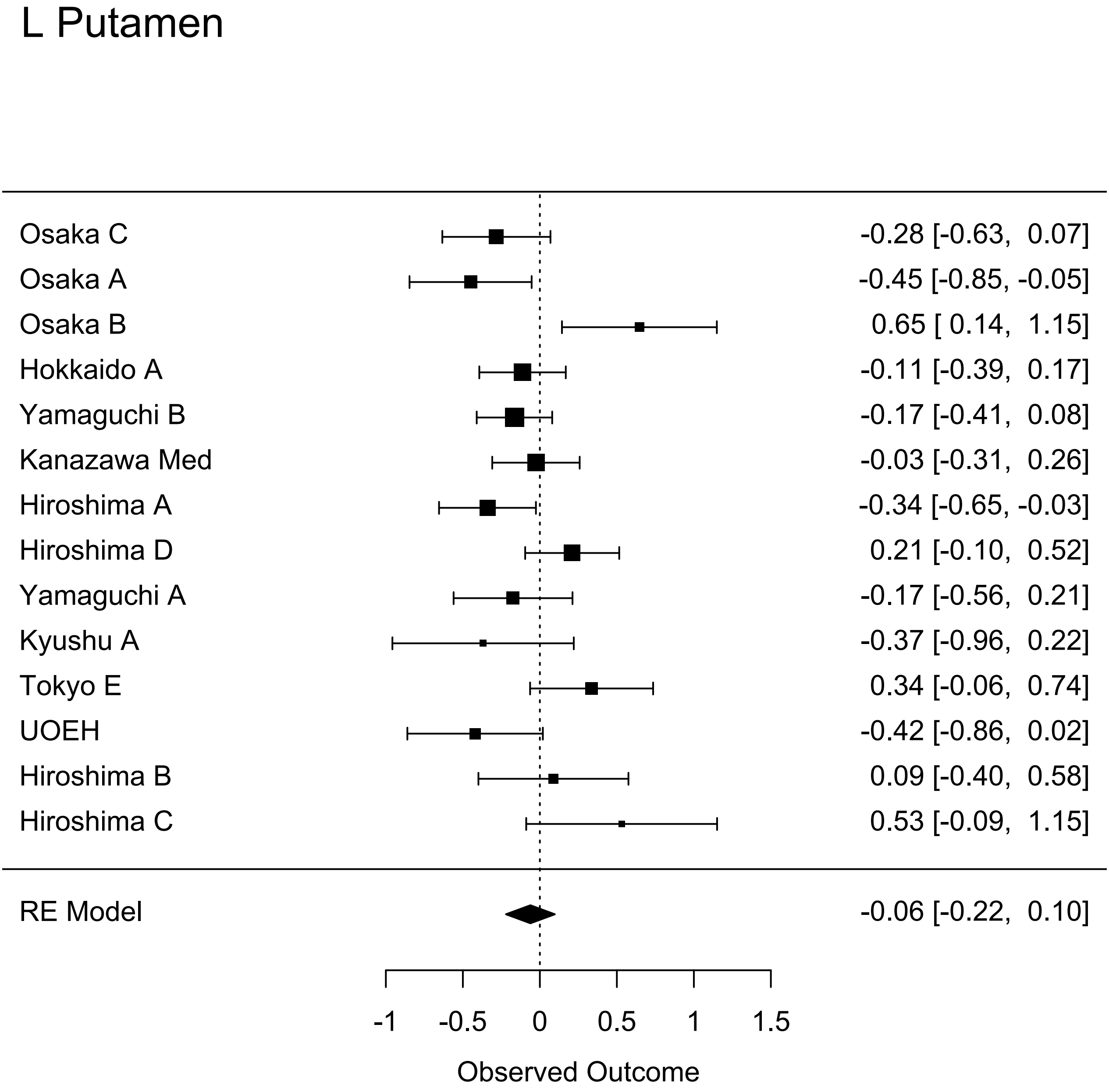

L Putamen
Osaka C -0.28 [-0.63, 0.07]
Osaka A -0.45 [-0.85, -0.05]
Osaka B 0.65 [0.14, 1.15]
Hokkaido A -0.11 [-0.39, 0.17]
Yamaguchi B -0.17 [-0.41, 0.08]
Kanazawa Med -0.03 [-0.31, 0.26]
Hiroshima A -0.34 [-0.65, -0.03]
Hiroshima D 0.21 [-0.10, 0.52]
Yamaguchi A -0.17 [-0.56, 0.21]
Kyushu A -0.37 [-0.96, 0.22]
Tokyo E 0.34 [-0.06, 0.74]
UOEH -0.42 [-0.86, 0.02]
Hiroshima B 0.09 [-0.40, 0.58]
Hiroshima C 0.53 [-0.09, 1.15]
RE Model -0.06 [-0.22, 0.10]
-1
-0.5
0
0.5
1
1.5
Observed Outcome

R Putamen

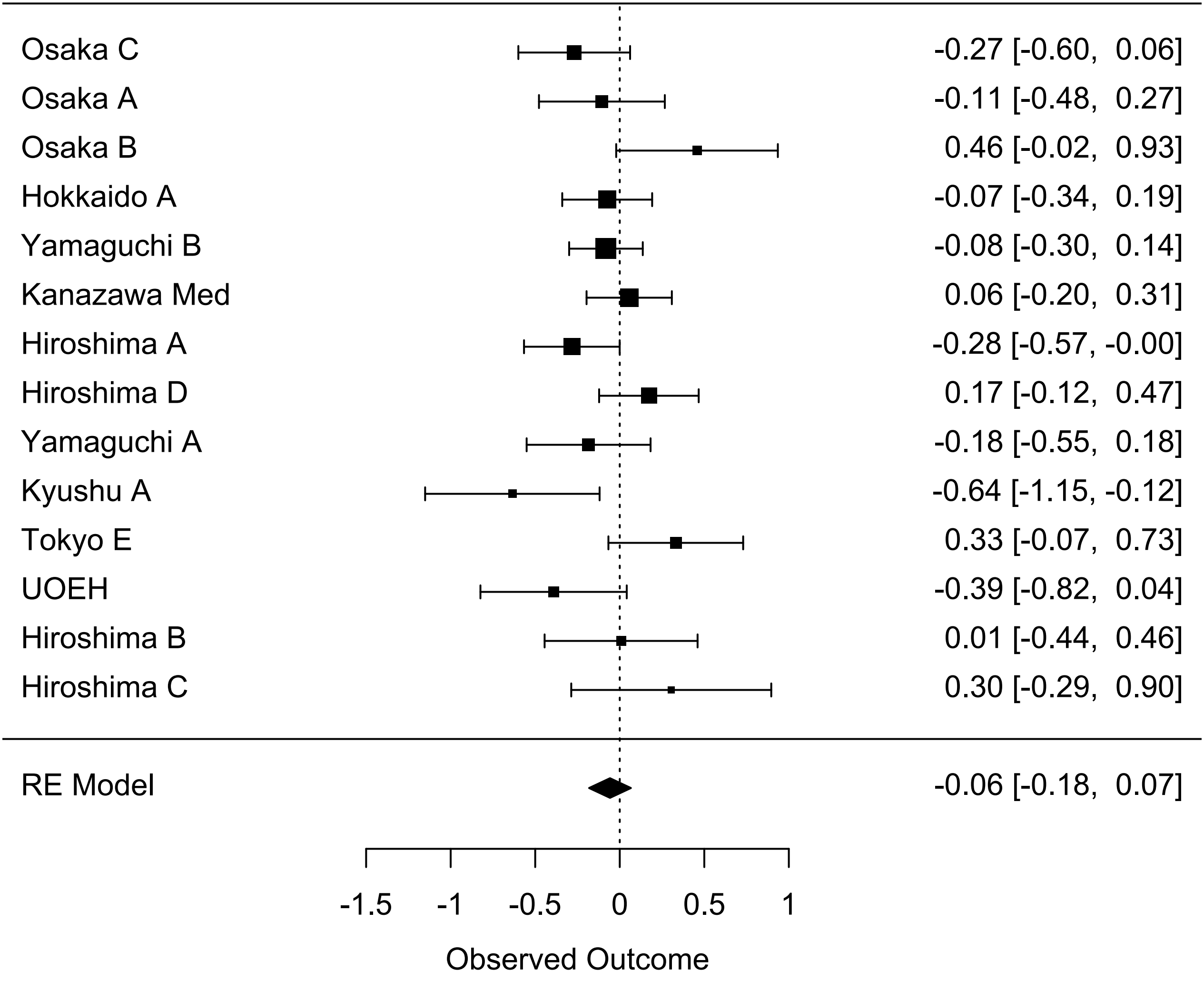
Osaka C
-0.27 [-0.60, 0.06]
Osaka A
-0.11 [-0.48, 0.27]
Osaka B
0.46 [-0.02, 0.93]
Hokkaido A
-0.07 [-0.34, 0.19]
Yamaguchi B
-0.08 [-0.30, 0.14]
Kanazawa Med
0.06 [-0.20, 0.31]
Hiroshima A
-0.28 [-0.57, -0.00]
Hiroshima D
0.17 [-0.12, 0.47]
Yamaguchi A
-0.18 [-0.55, 0.18]
Kyushu A
-0.64 [-1.15, -0.12]
Tokyo E
0.33 [-0.07, 0.73]
UOEH
-0.39 [-0.82, 0.04]
Hiroshima B
0.01 [-0.44, 0.46]
Hiroshima C
0.30 [-0.29, 0.90]
RE Model
-0.06 [-0.18, 0.07]
-1.5
-1
-0.5
0
0.5
1
Observed Outcome

L Pallidum

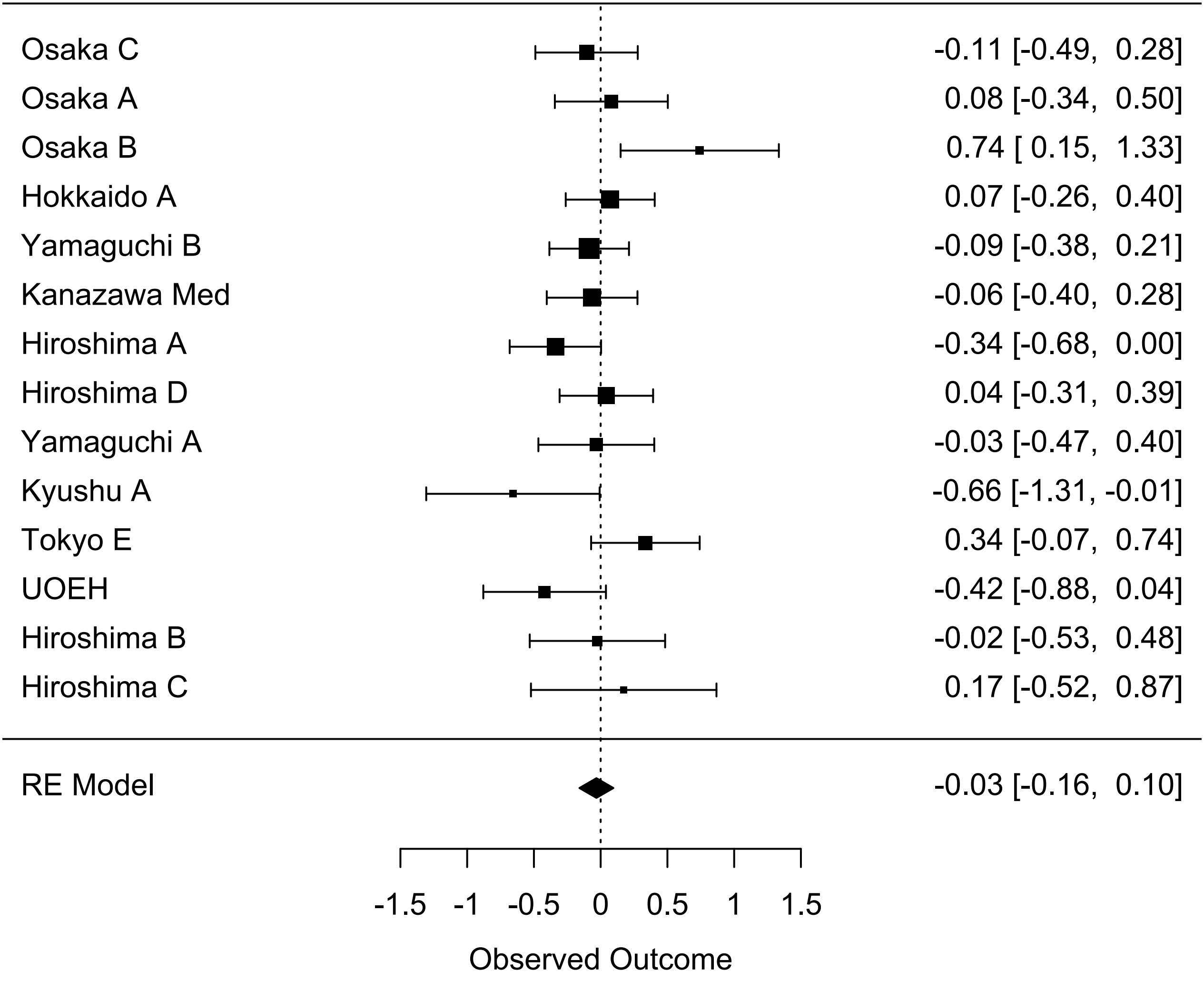

Osaka C -0.11 [-0.49, 0.28]
Osaka A 0.08 [-0.34, 0.50]
Osaka B 0.74 [0.15, 1.33]
Hokkaido A 0.07 [-0.26, 0.40]
Yamaguchi B -0.09 [-0.38, 0.21]
Kanazawa Med -0.06 [-0.40, 0.28]
Hiroshima A -0.34 [-0.68, 0.00]
Hiroshima D 0.04 [-0.31, 0.39]
Yamaguchi A -0.03 [-0.47, 0.40]
Kyushu A -0.66 [-1.31, -0.01]
Tokyo E 0.34 [-0.07, 0.74]
UOEH -0.42 [-0.88, 0.04]
Hiroshima B -0.02 [-0.53, 0.48]
Hiroshima C 0.17 [-0.52, 0.87]
RE Model -0.03 [-0.16, 0.10]
-1.5
-1
-0.5
0
0.5
1
1.5
Observed Outcome

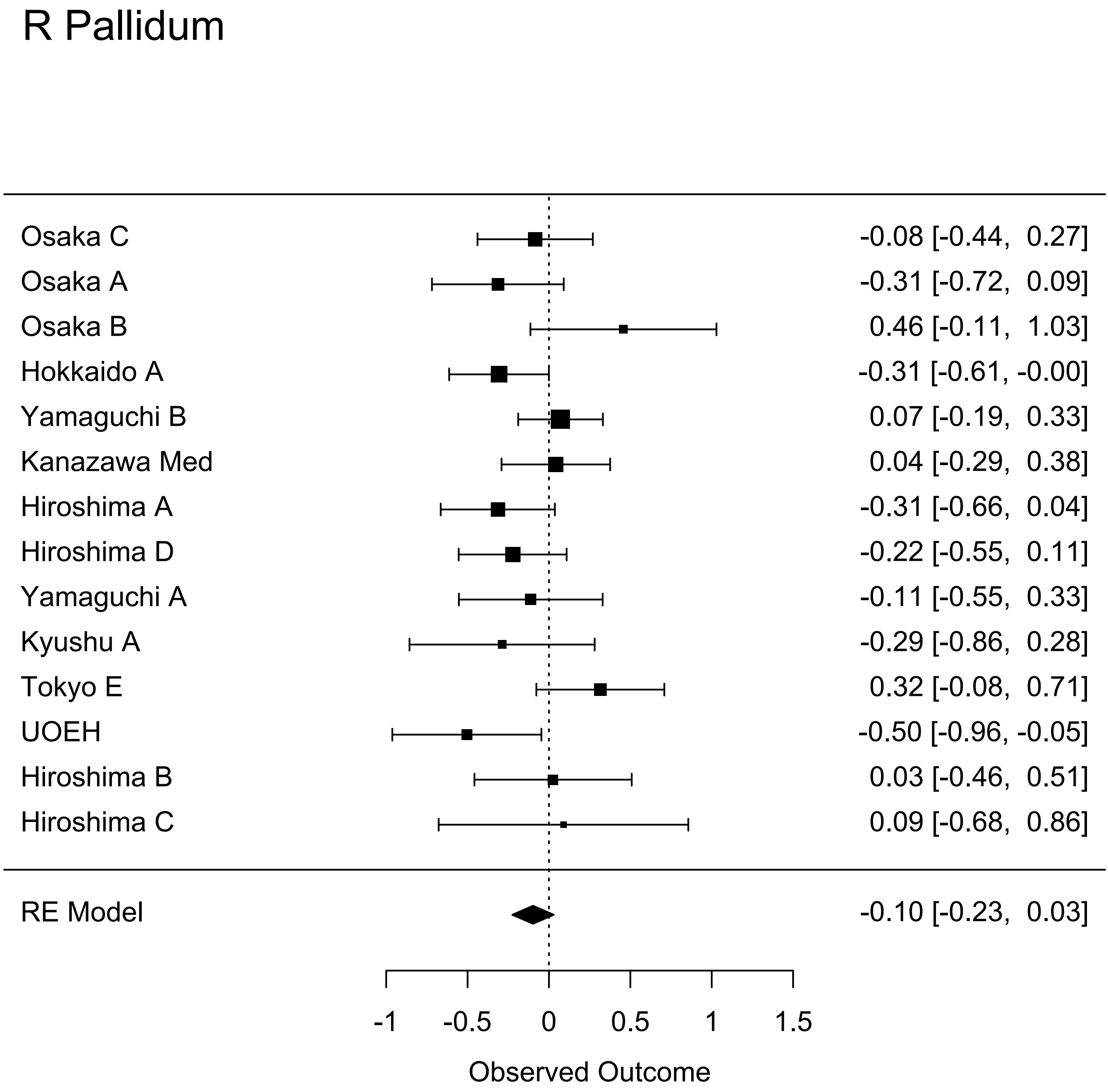
R Pallidum
Osaka C -0.08 [-0.44, 0.27]
Osaka A -0.31 [-0.72, 0.09]
Osaka B 0.46 [-0.11, 1.03]
Hokkaido A -0.31 [-0.61, -0.00]
Yamaguchi B 0.07 [-0.19, 0.33]
Kanazawa Med 0.04 [-0.29, 0.38]
Hiroshima A -0.31 [-0.66, 0.04]
Hiroshima D -0.22 [-0.55, 0.11]
Yamaguchi A -0.11 [-0.55, 0.33]
Kyushu A -0.29 [-0.86, 0.28]
Tokyo E 0.32 [-0.08, 0.71]
UOEH -0.50 [-0.96, -0.05]
Hiroshima B 0.03 [-0.46, 0.51]
Hiroshima C 0.09 [-0.68, 0.86]
RE Model -0.10 [-0.23, 0.03]
-1
-0.5
0
0.5
1
1.5
Observed Outcome

L Lateral ventricles

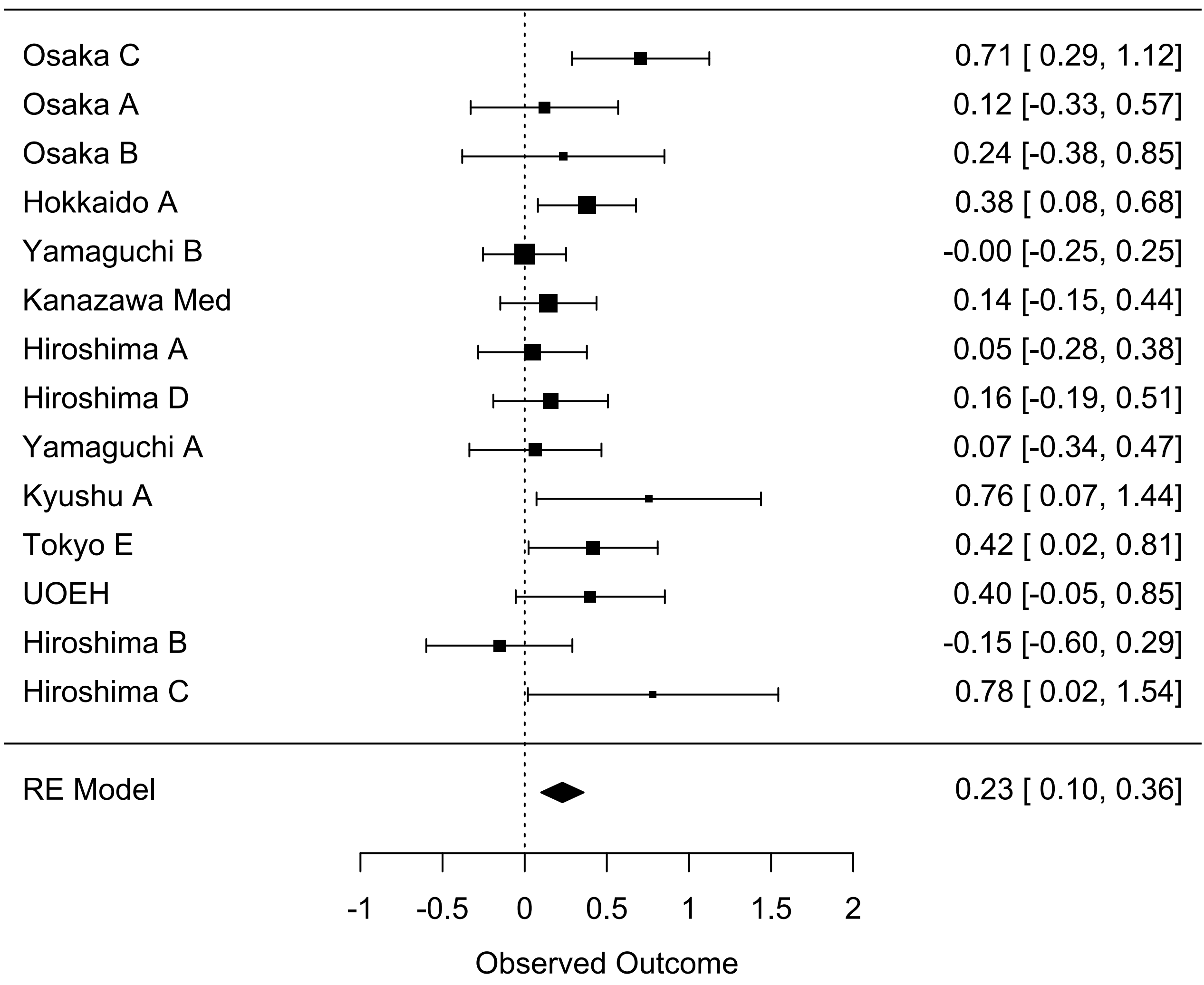

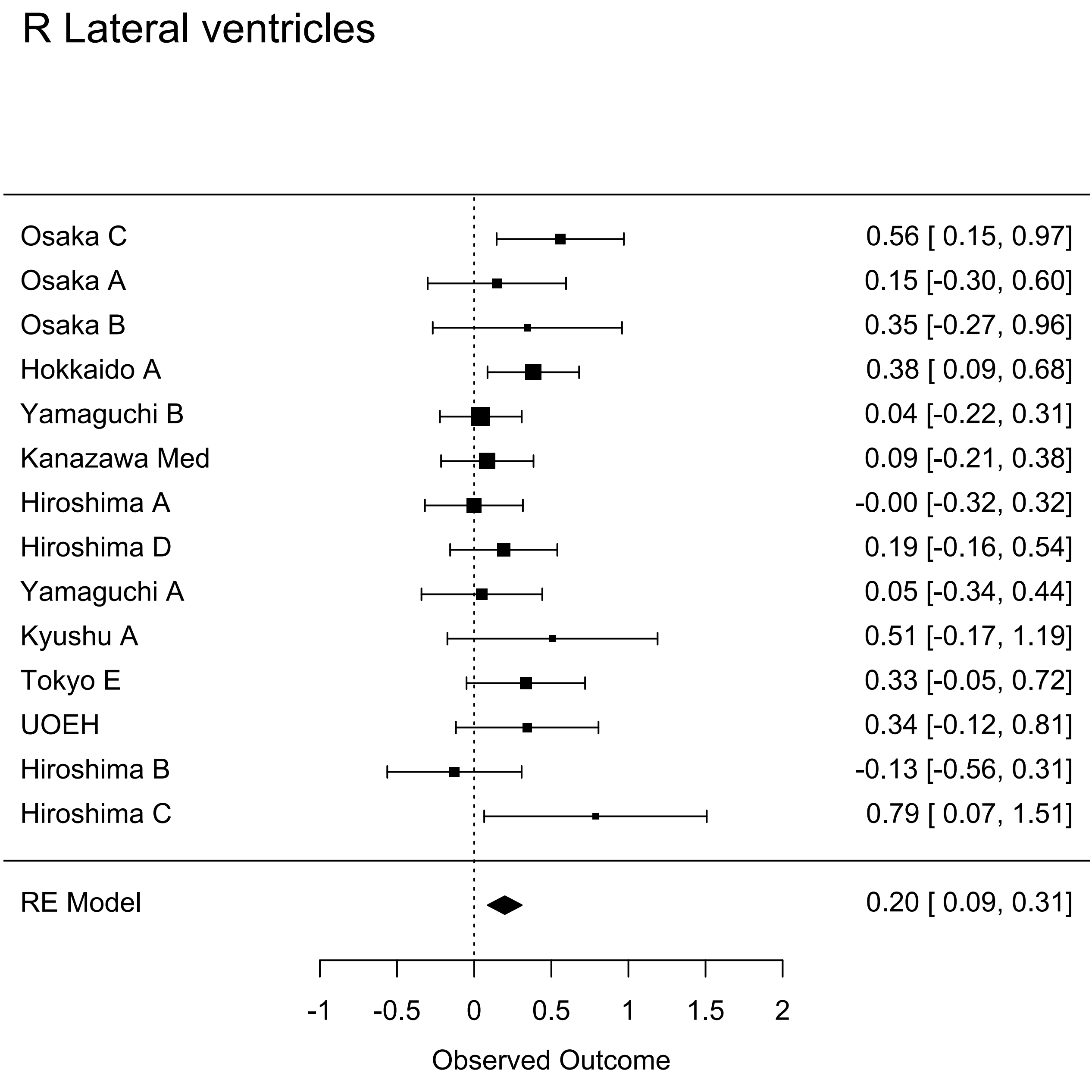
R Lateral ventricles
Osaka C 0.56 [0.15, 0.97]
Osaka A 0.15 [-0.30, 0.60]
Osaka B 0.35 [-0.27, 0.96]
Hokkaido A 0.38 [0.09, 0.68]
Yamaguchi B 0.04 [-0.22, 0.31]
Kanazawa Med 0.09 [-0.21, 0.38]
Hiroshima A -0.00 [-0.32, 0.32]
Hiroshima D 0.19 [-0.16, 0.54]
Yamaguchi A 0.05 [-0.34, 0.44]
Kyushu A 0.51 [-0.17, 1.19]
Tokyo E 0.33 [-0.05, 0.72]
UOEH 0.34 [-0.12, 0.81]
Hiroshima B -0.13 [-0.56, 0.31]
Hiroshima C 0.79 [0.07, 1.51]
RE Model 0.20 [0.09, 0.31]
-1
-0.5
0
0.5
1
1.5
2
Observed Outcome

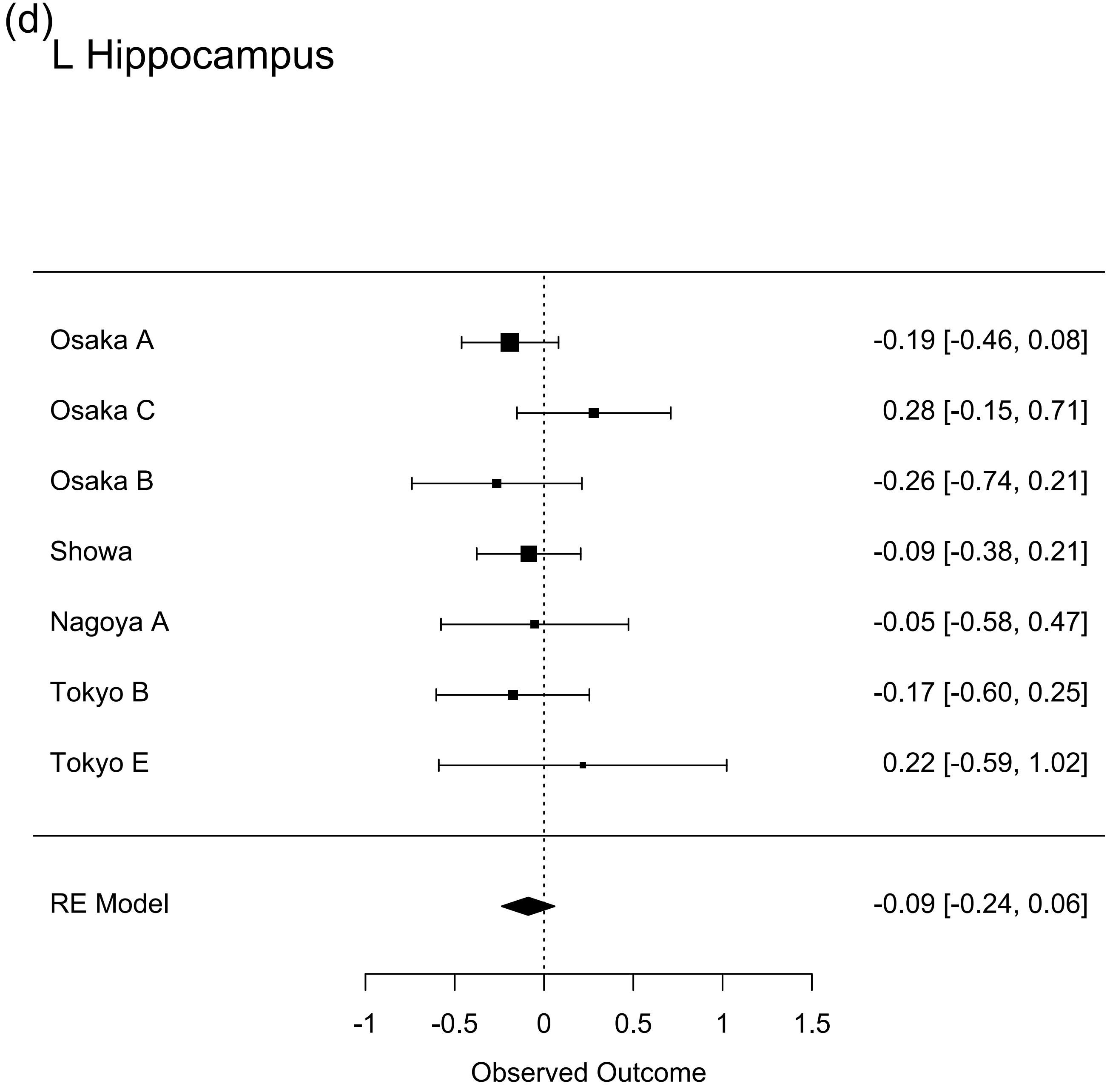
(d)
L Hippocampus
Osaka A
-0.19 [-0.46, 0.08]
Osaka C
0.28 [-0.15, 0.71]
Osaka B
-0.26 [-0.74, 0.21]
Showa
-0.09 [-0.38, 0.21]
Nagoya A
-0.05 [-0.58, 0.47]
Tokyo B
-0.17 [-0.60, 0.25]
Tokyo E
0.22 [-0.59, 1.02]
RE Model
-0.09 [-0.24, 0.06]
-1
-0.5
0
0.5
1
1.5
Observed Outcome

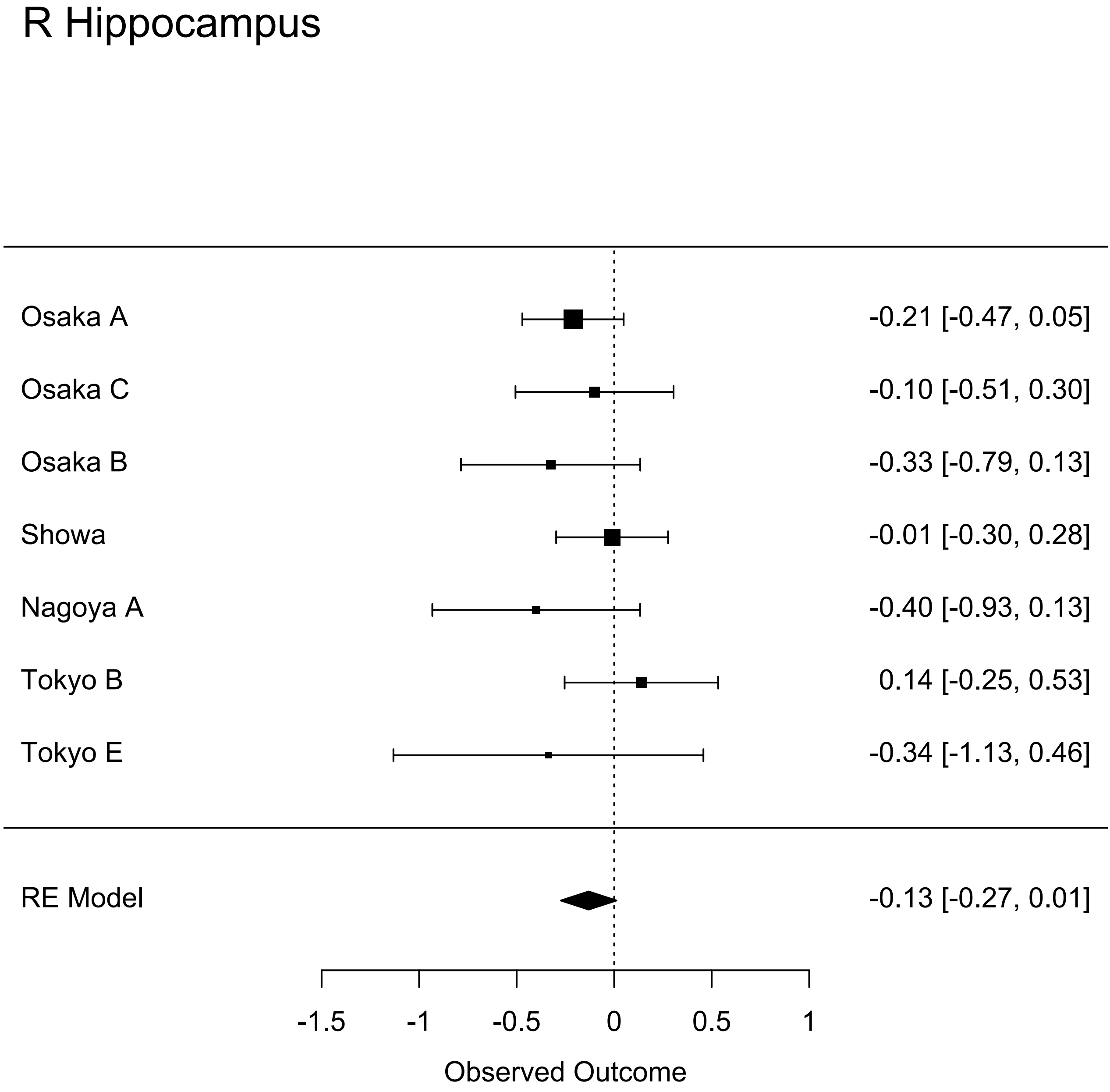
R Hippocampus
Osaka A
-0.21 [-0.47, 0.05]
Osaka C
-0.10 [-0.51, 0.30]
Osaka B
-0.33 [-0.79, 0.13]
Showa
-0.01 [-0.30, 0.28]
Nagoya A
-0.40 [-0.93, 0.13]
Tokyo B
0.14 [-0.25, 0.53]
Tokyo E
-0.34 [-1.13, 0.46]
RE Model
-0.13 [-0.27, 0.01]
-1.5
-1
-0.5
0
0.5
1
Observed Outcome

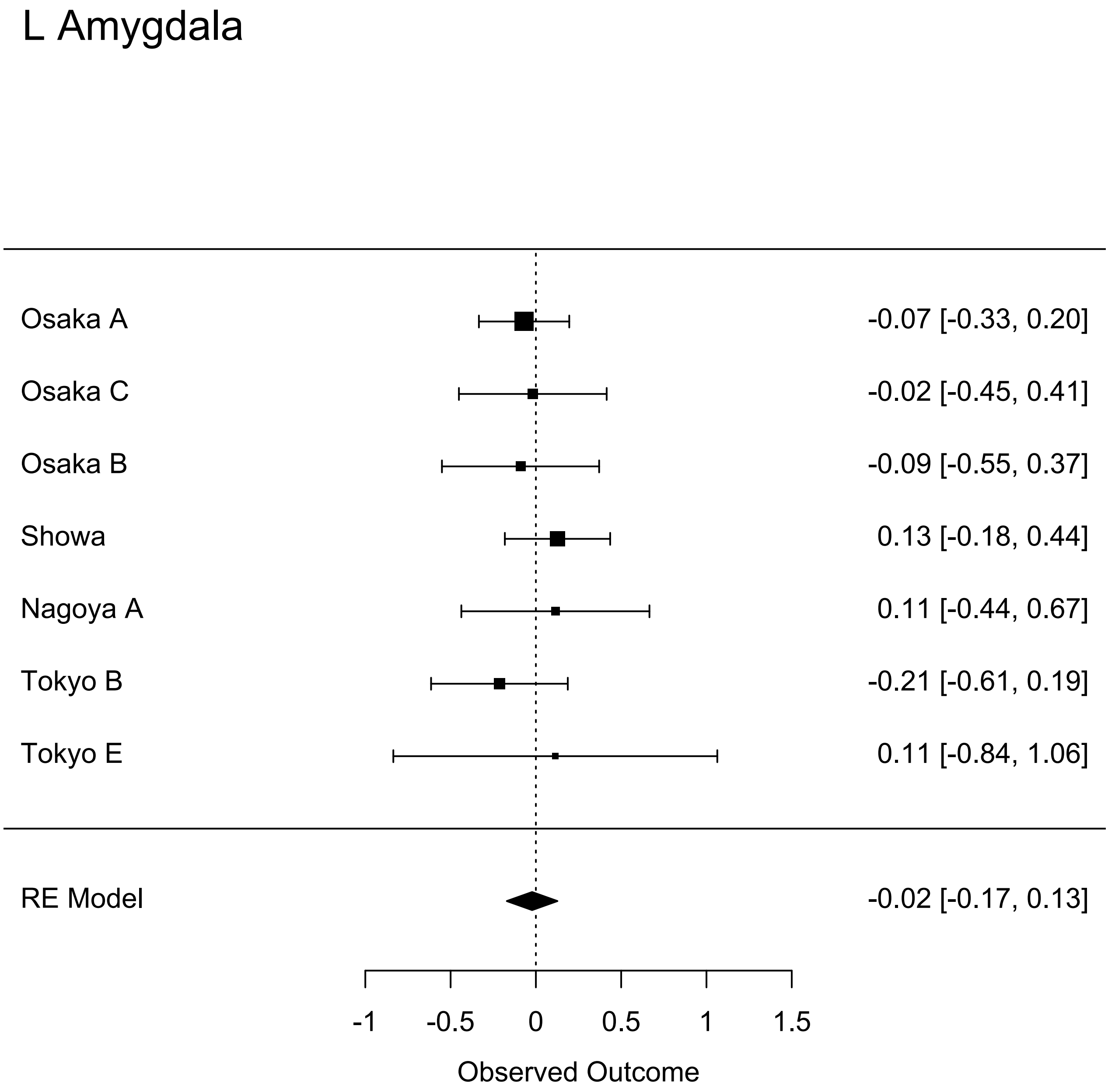

L Amygdala
Osaka A
Osaka C
Osaka B
Showa
Nagoya A
Tokyo B
Tokyo E
RE Model
-0.07 [-0.33, 0.20]
-0.02 [-0.45, 0.41]
-0.09 [-0.55, 0.37]
0.13 [-0.18, 0.44]
0.11 [-0.44, 0.67]
-0.21 [-0.61, 0.19]
0.11 [-0.84, 1.06]
-0.02 [-0.17, 0.13]
-1
-0.5
0
0.5
1
1.5
Observed Outcome

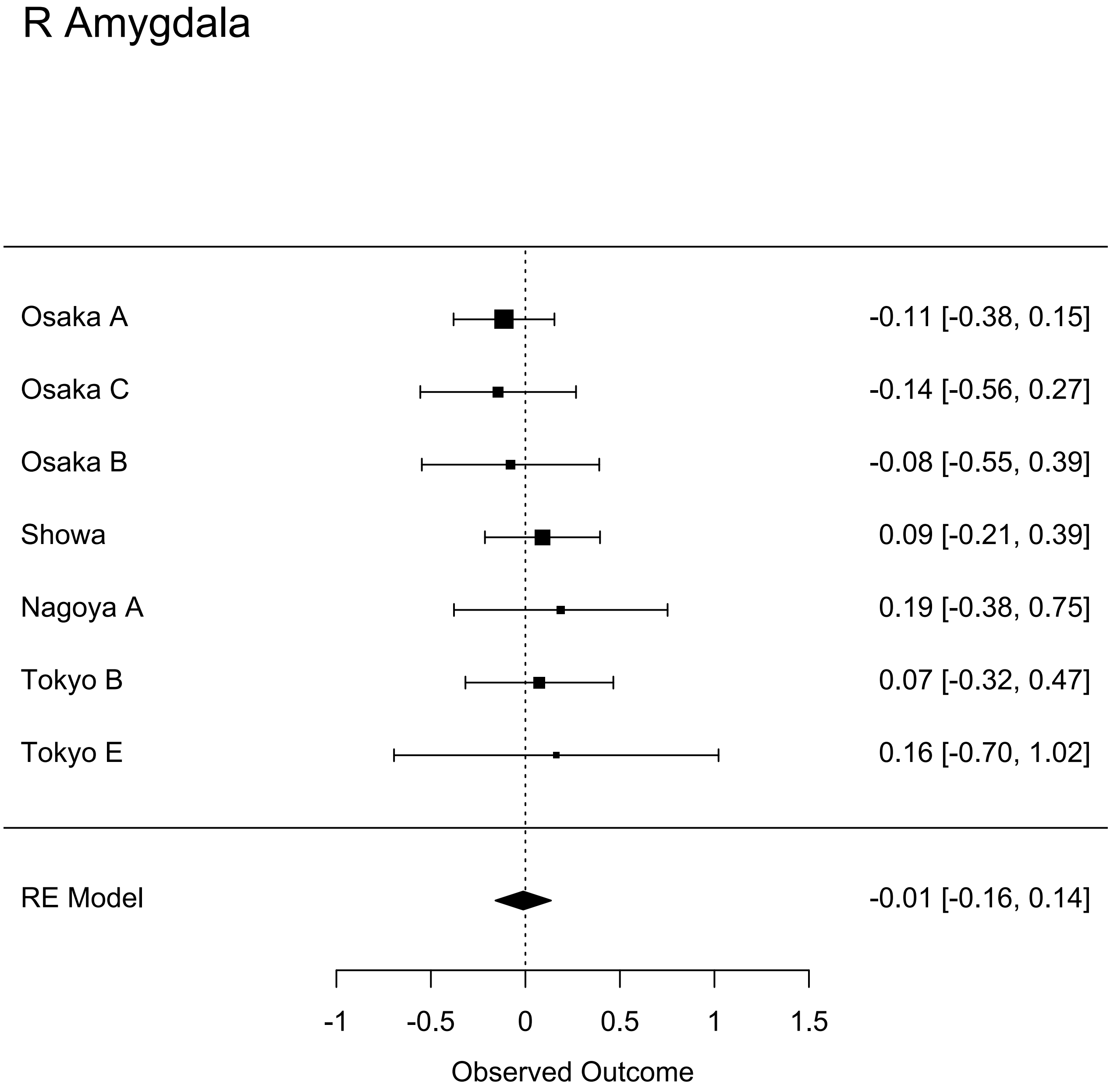
R Amygdala
Osaka A
-0.11 [-0.38, 0.15]
Osaka C
-0.14 [-0.56, 0.27]
Osaka B
-0.08 [-0.55, 0.39]
Showa
0.09 [-0.21, 0.39]
Nagoya A
0.19 [-0.38, 0.75]
Tokyo B
0.07 [-0.32, 0.47]
Tokyo E
0.16 [-0.70, 1.02]
RE Model
-0.01 [-0.16, 0.14]
-1
-0.5
0
0.5
1
1.5
Observed Outcome

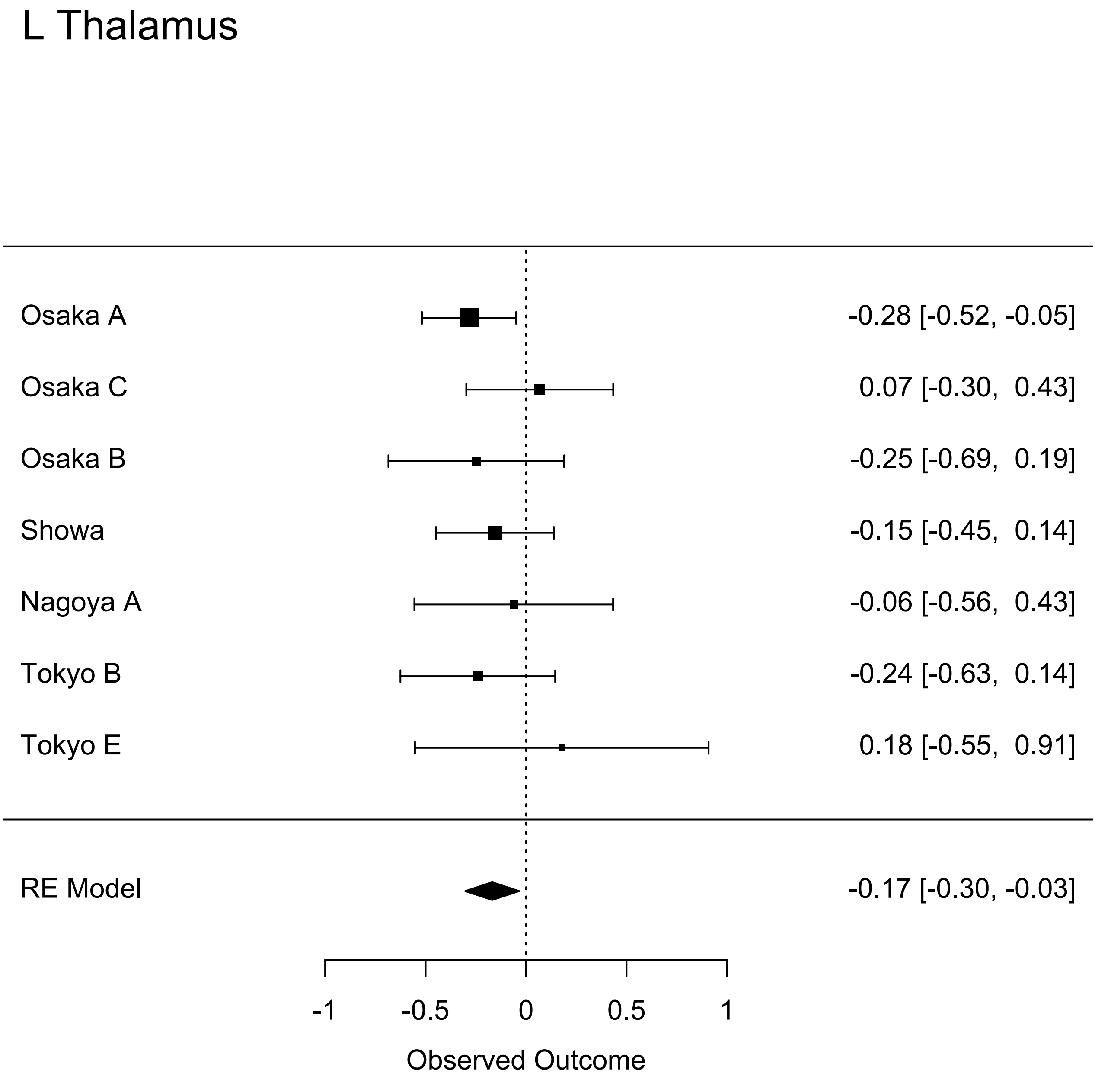
L Thalamus
Osaka A
-0.28 [-0.52, -0.05]
Osaka C
0.07 [-0.30, 0.43]
Osaka B
-0.25 [-0.69, 0.19]
Showa
-0.15 [-0.45, 0.14]
Nagoya A
-0.06 [-0.56, 0.43]
Tokyo B
-0.24 [-0.63, 0.14]
Tokyo E
0.18 [-0.55, 0.91]
RE Model
-0.17 [-0.30, -0.03]
-1
-0.5
0
0.5
1
Observed Outcome

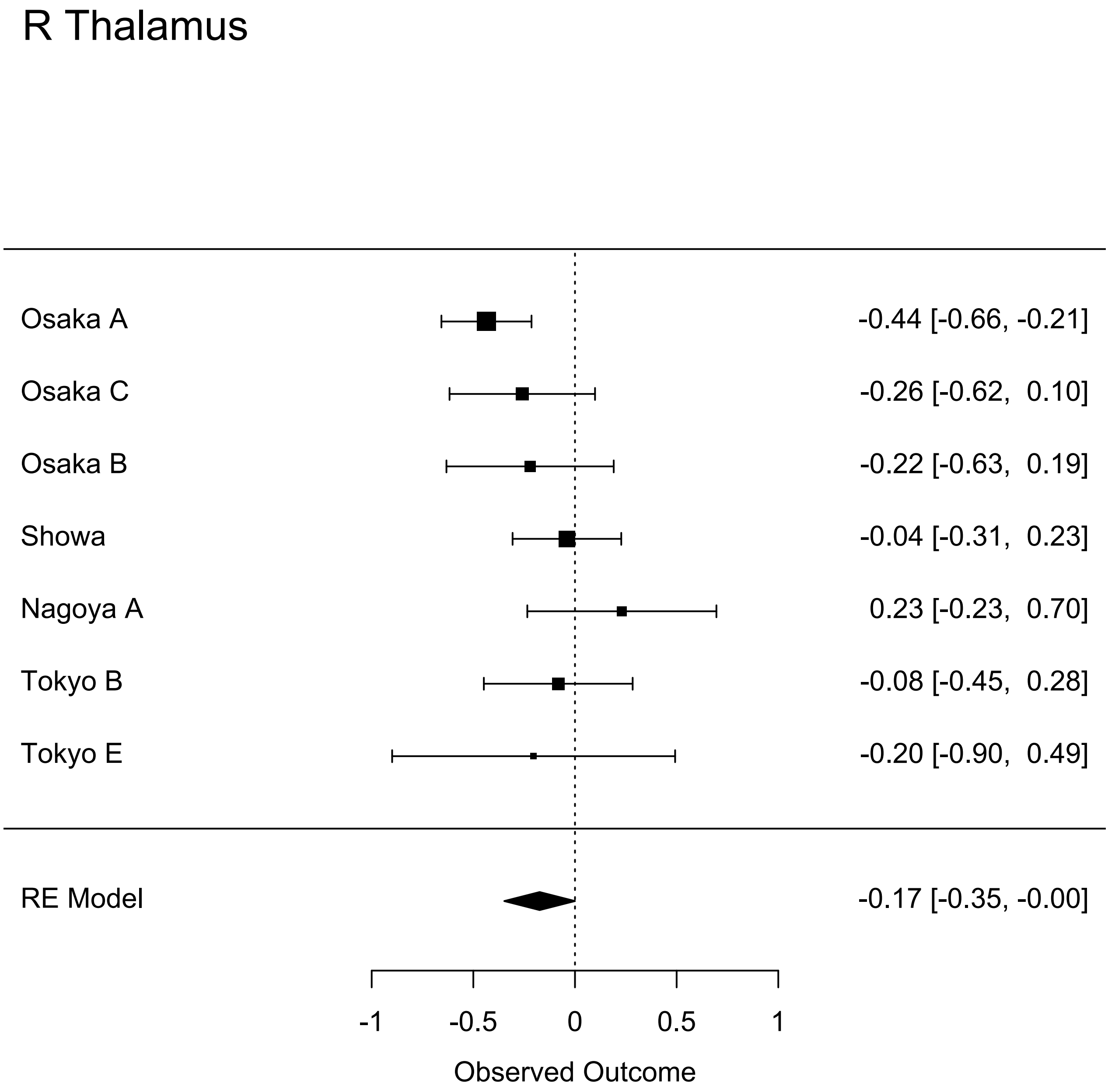
R Thalamus
Osaka A
-0.44 [-0.66, -0.21]
Osaka C
-0.26 [-0.62, 0.10]
Osaka B
-0.22 [-0.63, 0.19]
Showa
-0.04 [-0.31, 0.23]
Nagoya A
0.23 [-0.23, 0.70]
Tokyo B
-0.08 [-0.45, 0.28]
Tokyo E
-0.20 [-0.90, 0.49]
RE Model
-0.17 [-0.35, -0.00]
-1
-0.5
0
0.5
1
Observed Outcome

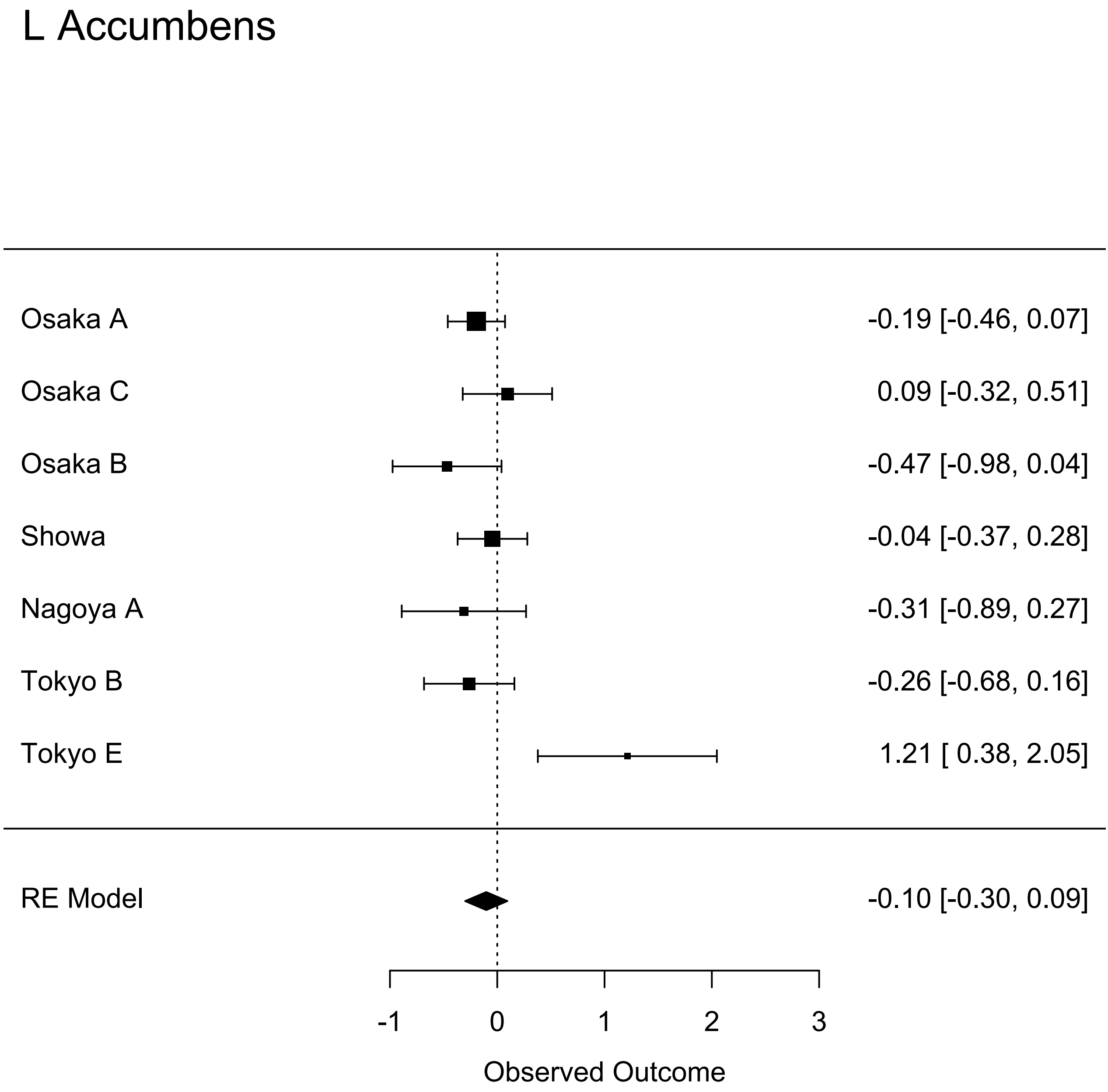
L Accumbens
Osaka A
-0.19 [-0.46, 0.07]
Osaka C
0.09 [-0.32, 0.51]
Osaka B
-0.47 [-0.98, 0.04]
Showa
-0.04 [-0.37, 0.28]
Nagoya A
-0.31 [-0.89, 0.27]
Tokyo B
-0.26 [-0.68, 0.16]
Tokyo E
1.21 [0.38, 2.05]
RE Model
-0.10 [-0.30, 0.09]
-1
0
1
2
3
Observed Outcome

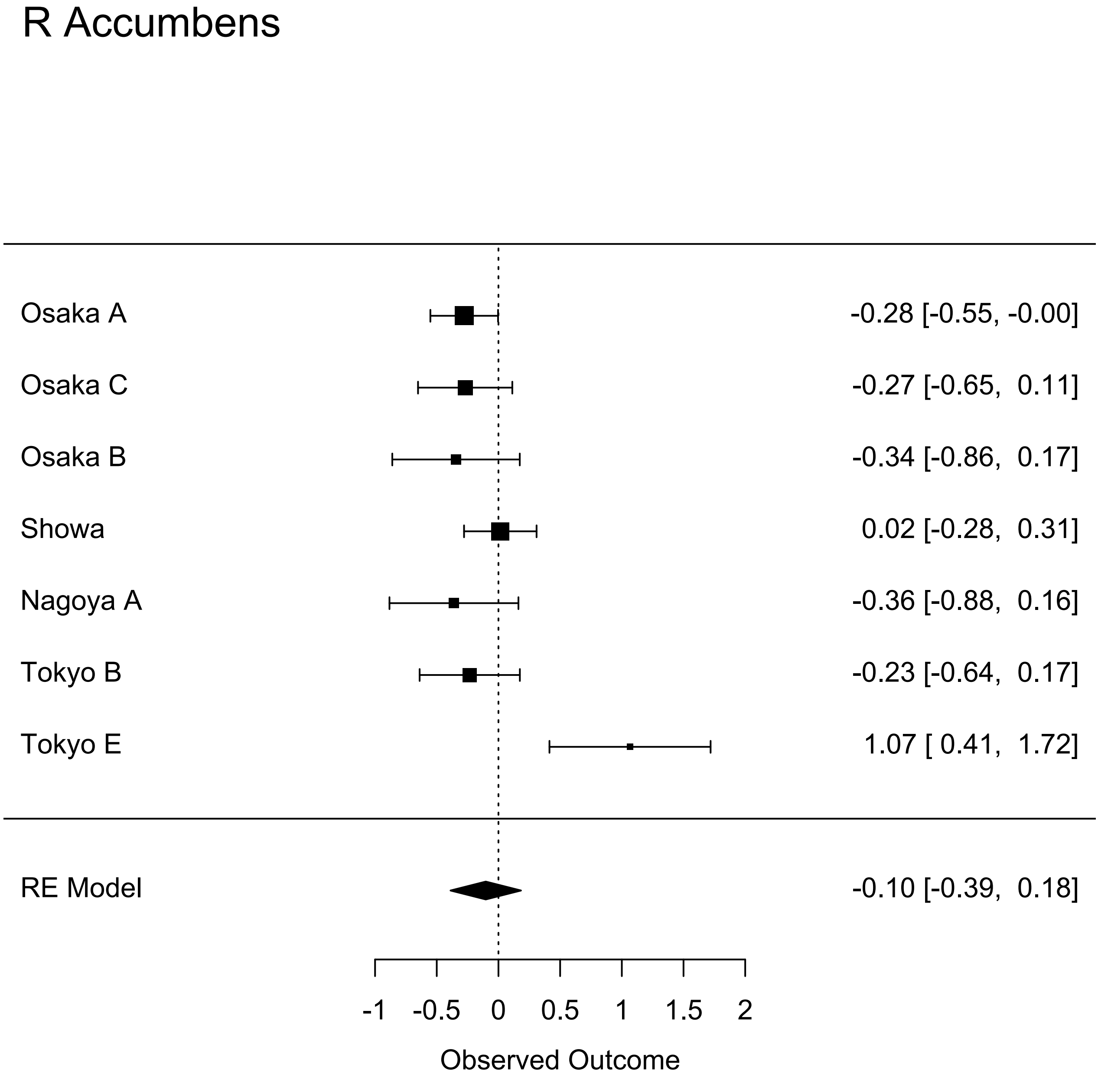
R Accumbens
Osaka A
-0.28 [-0.55, -0.00]
Osaka C
-0.27 [-0.65, 0.11]
Osaka B
-0.34 [-0.86, 0.17]
Showa
0.02 [-0.28, 0.31]
Nagoya A
-0.36 [-0.88, 0.16]
Tokyo B
-0.23 [-0.64, 0.17]
Tokyo E
1.07 [0.41, 1.72]
RE Model
-0.10 [-0.39, 0.18]
-1
-0.5
0
0.5
1
1.5
2
Observed Outcome

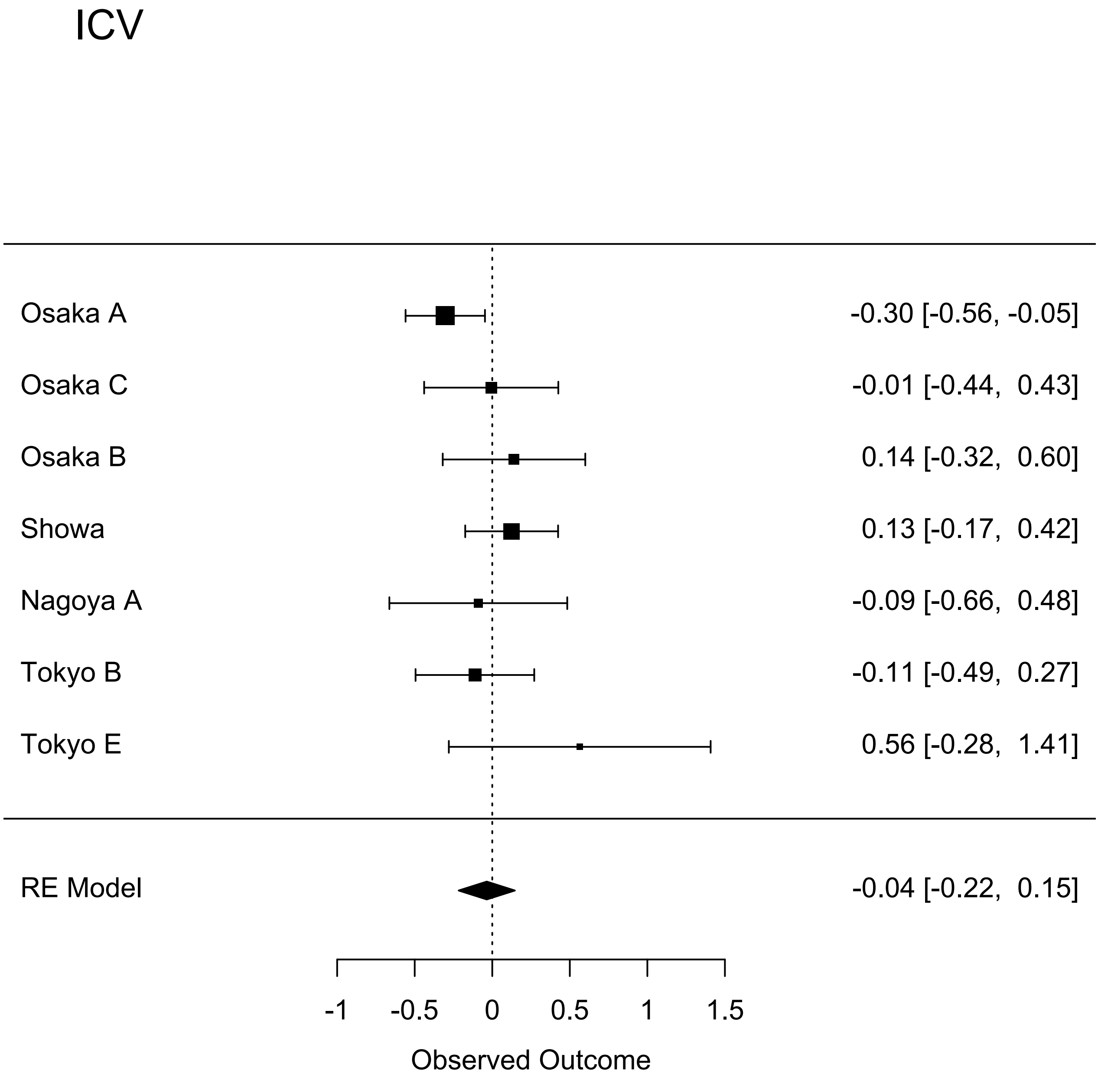
ICV
Osaka A
Osaka C
Osaka B
Showa
Nagoya A
Tokyo B
Tokyo E
RE Model
-0.30 [-0.56, -0.05]
-0.01 [-0.44, 0.43]
0.14 [-0.32, 0.60]
0.13 [-0.17, 0.42]
-0.09 [-0.66, 0.48]
-0.11 [-0.49, 0.27]
0.56 [-0.28, 1.41]
-0.04 [-0.22, 0.15]
-1
-0.5
0
0.5
1
1.5
Observed Outcome

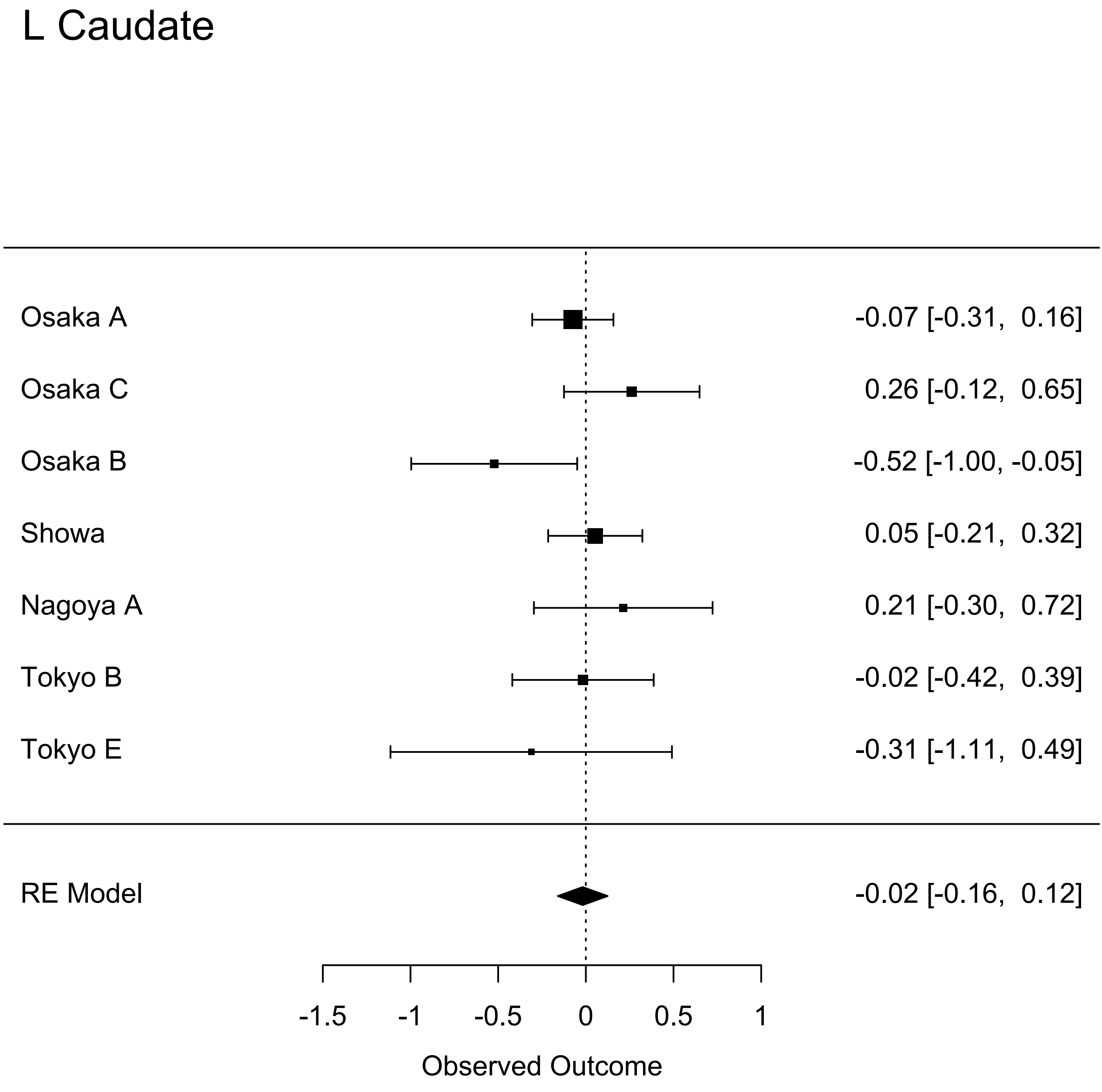
L Caudate
Osaka A
-0.07 [-0.31, 0.16]
Osaka C
0.26 [-0.12, 0.65]
Osaka B
-0.52 [-1.00, -0.05]
Showa
0.05 [-0.21, 0.32]
Nagoya A
0.21 [-0.30, 0.72]
Tokyo B
-0.02 [-0.42, 0.39]
Tokyo E
-0.31 [-1.11, 0.49]
RE Model
-0.02 [-0.16, 0.12]
-1.5
-1
-0.5
0
0.5
1
Observed Outcome

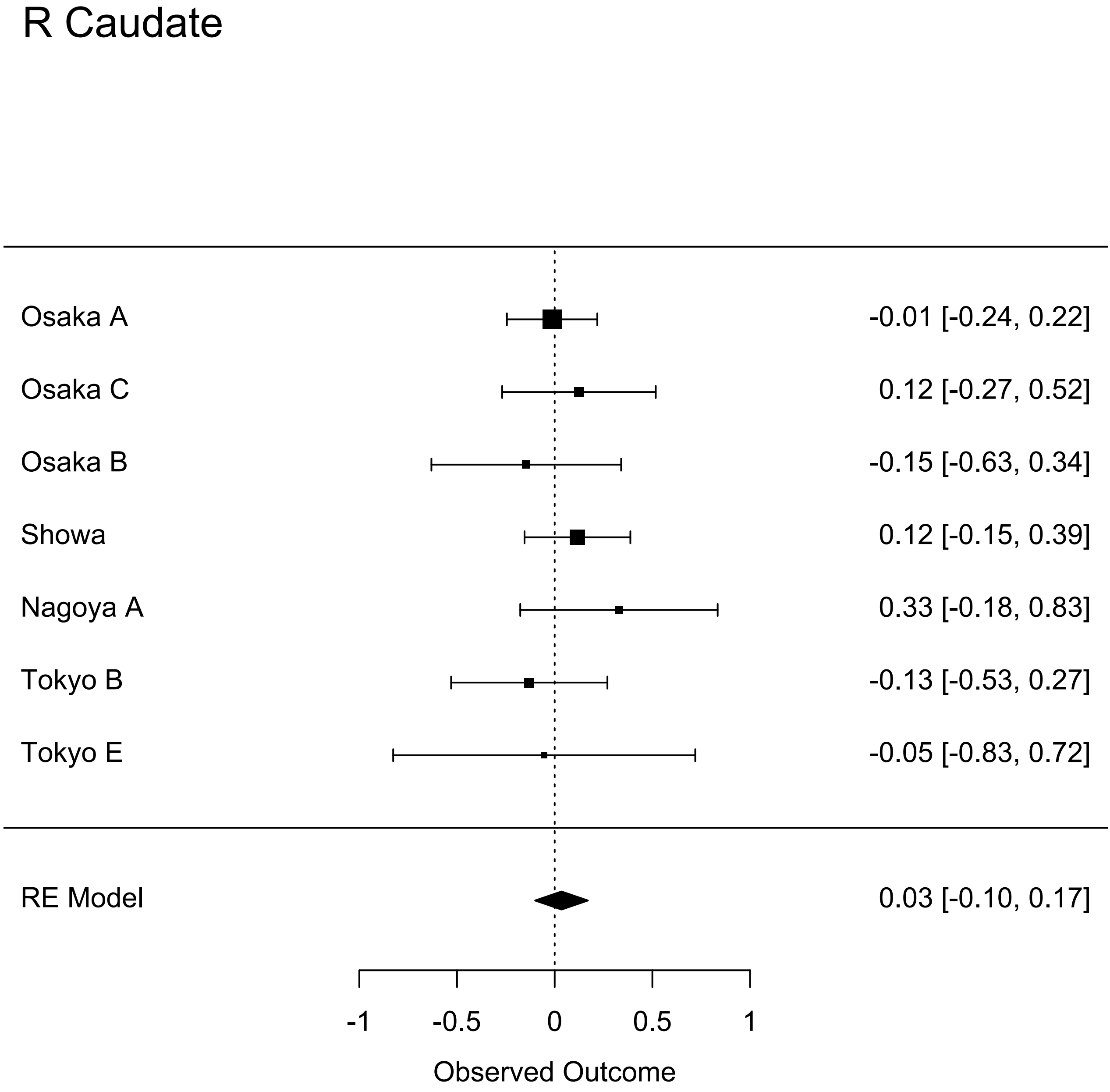
R Caudate
Osaka A
-0.01 [-0.24, 0.22]
Osaka C
0.12 [-0.27, 0.52]
Osaka B
-0.15 [-0.63, 0.34]
Showa
0.12 [-0.15, 0.39]
Nagoya A
0.33 [-0.18, 0.83]
Tokyo B
-0.13 [-0.53, 0.27]
Tokyo E
-0.05 [-0.83, 0.72]
RE Model
0.03 [-0.10, 0.17]
-1
-0.5
0
0.5
1
Observed Outcome

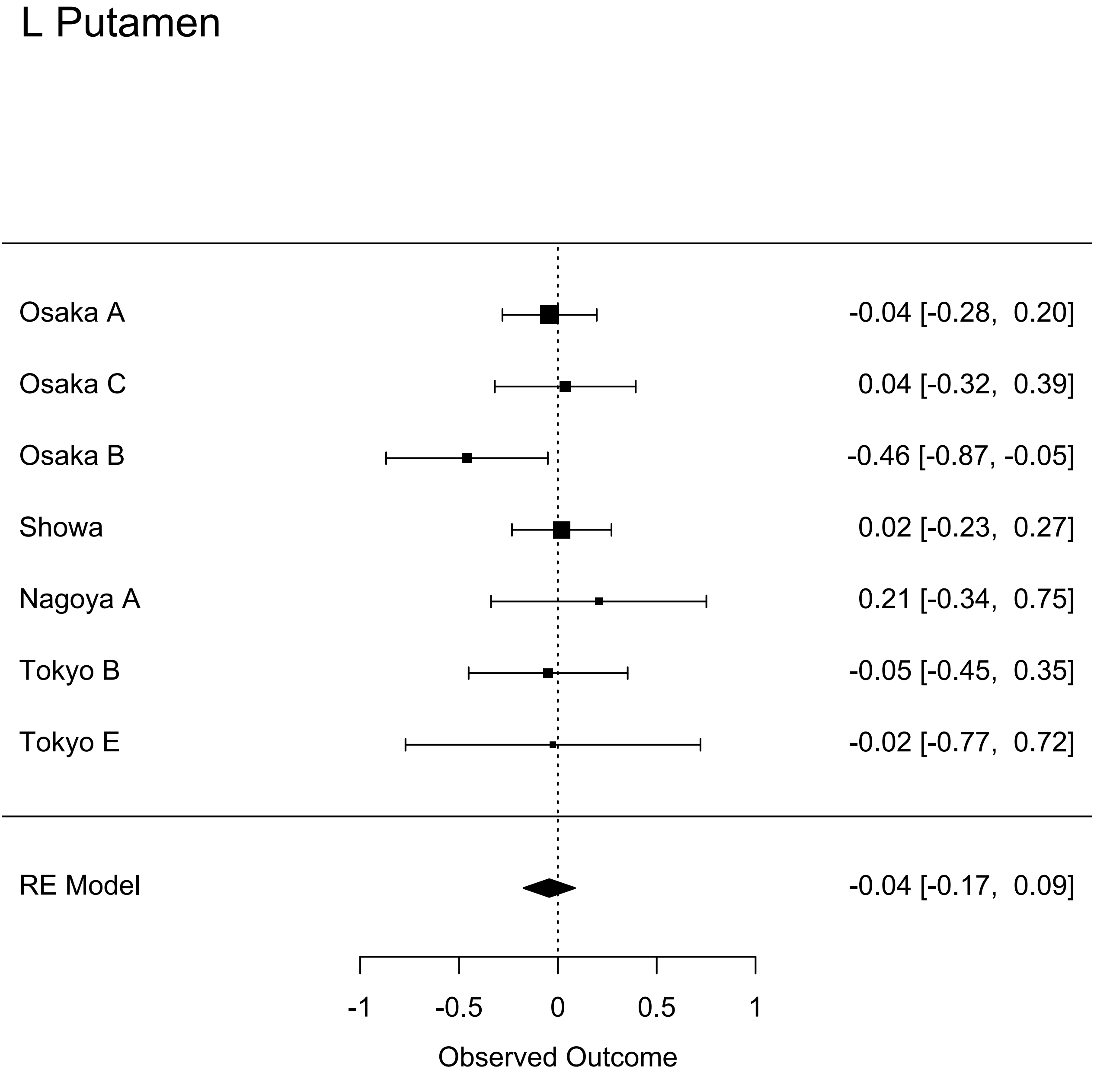
L Putamen
Osaka A
-0.04 [-0.28, 0.20]
Osaka C
0.04 [-0.32, 0.39]
Osaka B
-0.46 [-0.87, -0.05]
Showa
0.02 [-0.23, 0.27]
Nagoya A
0.21 [-0.34, 0.75]
Tokyo B
-0.05 [-0.45, 0.35]
Tokyo E
-0.02 [-0.77, 0.72]
RE Model
-0.04 [-0.17, 0.09]
-1
-0.5
0
0.5
1
Observed Outcome

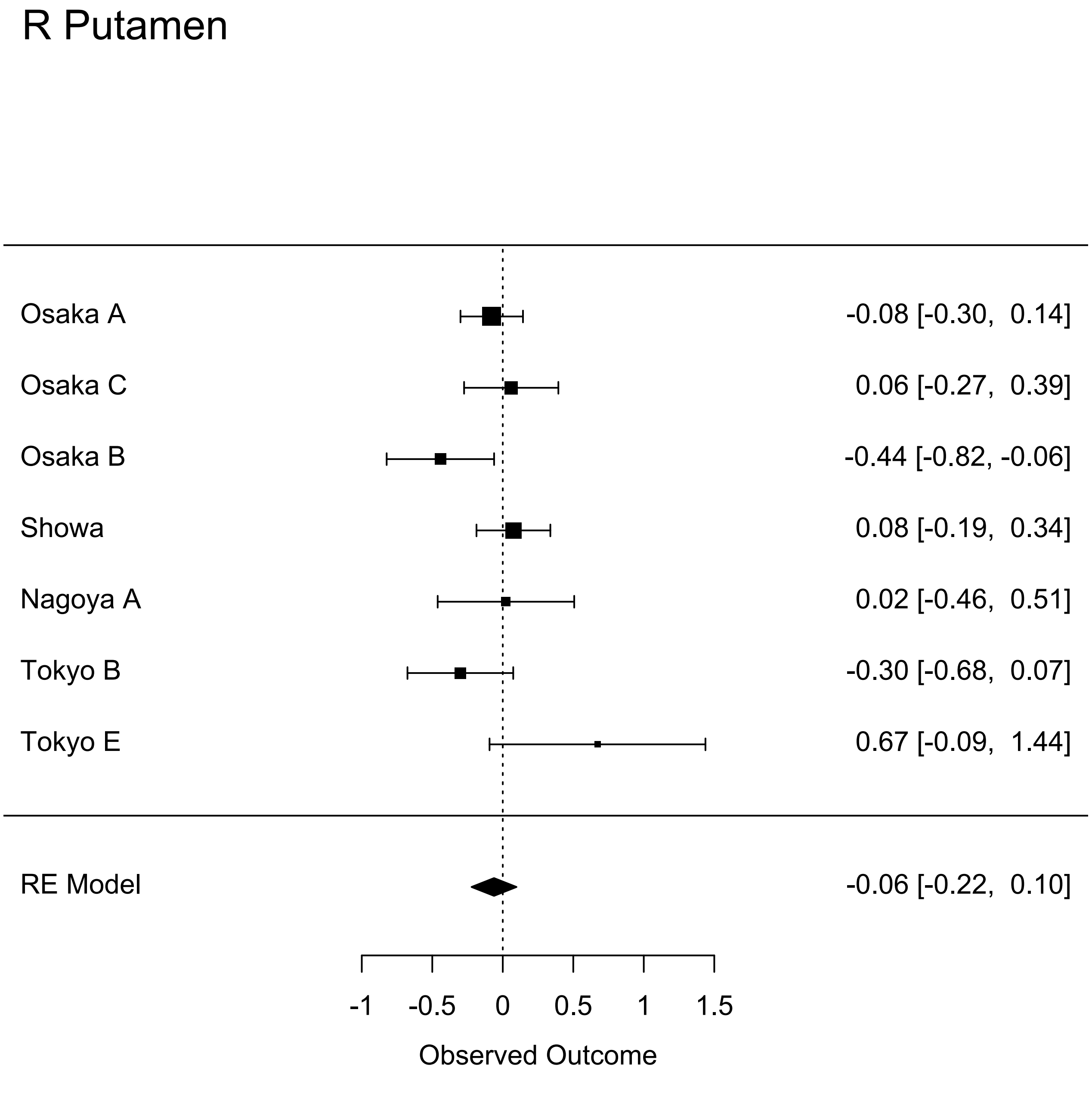
R Putamen
Osaka A
-0.08 [-0.30, 0.14]
Osaka C
0.06 [-0.27, 0.39]
Osaka B
-0.44 [-0.82, -0.06]
Showa
0.08 [-0.19, 0.34]
Nagoya A
0.02 [-0.46, 0.51]
Tokyo B
-0.30 [-0.68, 0.07]
Tokyo E
0.67 [-0.09, 1.44]
RE Model
-0.06 [-0.22, 0.10]
-1
-0.5
0
0.5
1
1.5
Observed Outcome

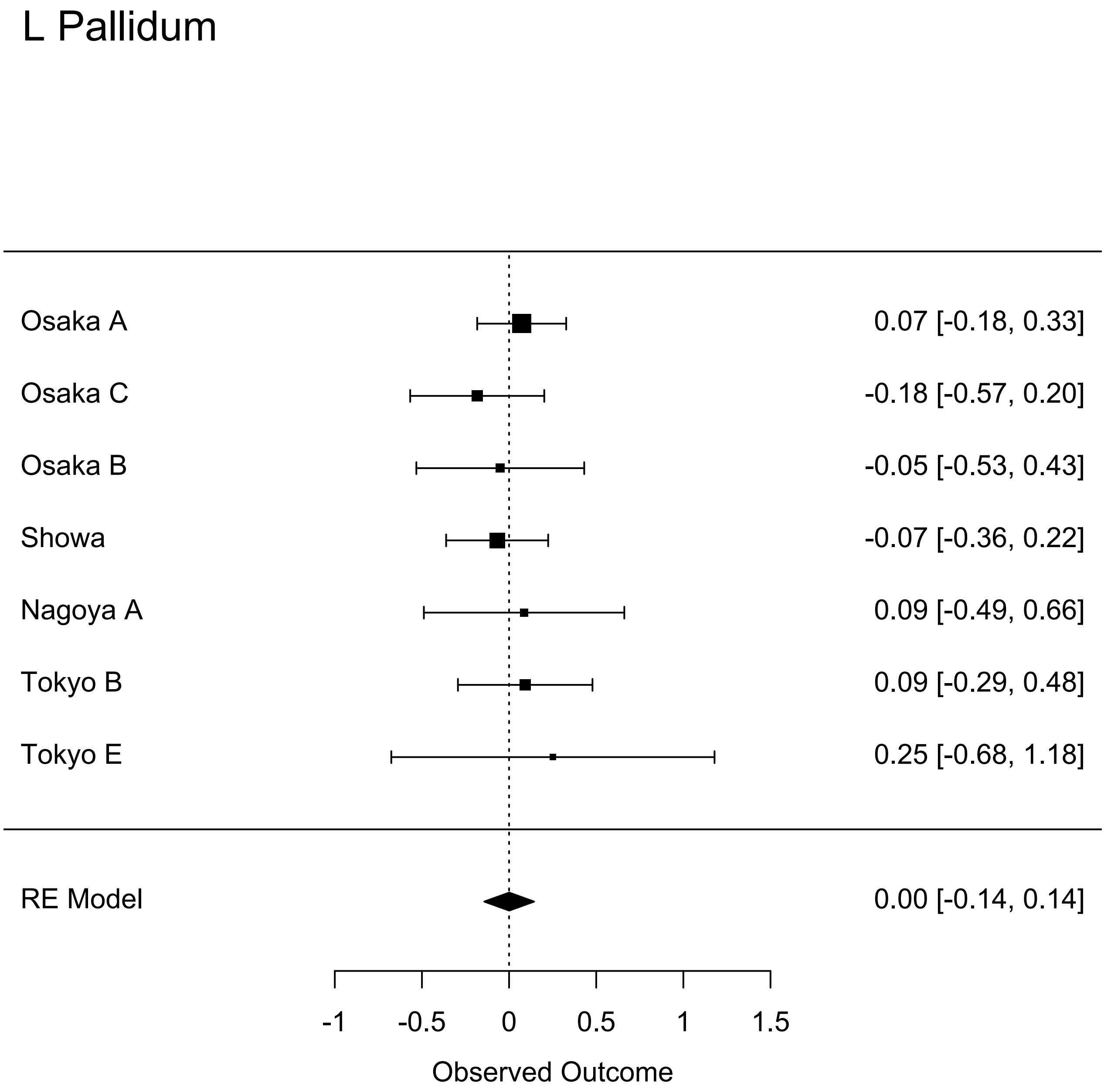
L Pallidum
Osaka A
0.07 [-0.18, 0.33]
Osaka C
-0.18 [-0.57, 0.20]
Osaka B
-0.05 [-0.53, 0.43]
Showa
-0.07 [-0.36, 0.22]
Nagoya A
0.09 [-0.49, 0.66]
Tokyo B
0.09 [-0.29, 0.48]
Tokyo E
0.25 [-0.68, 1.18]
RE Model
0.00 [-0.14, 0.14]
-1
-0.5
0
0.5
1
1.5
Observed Outcome

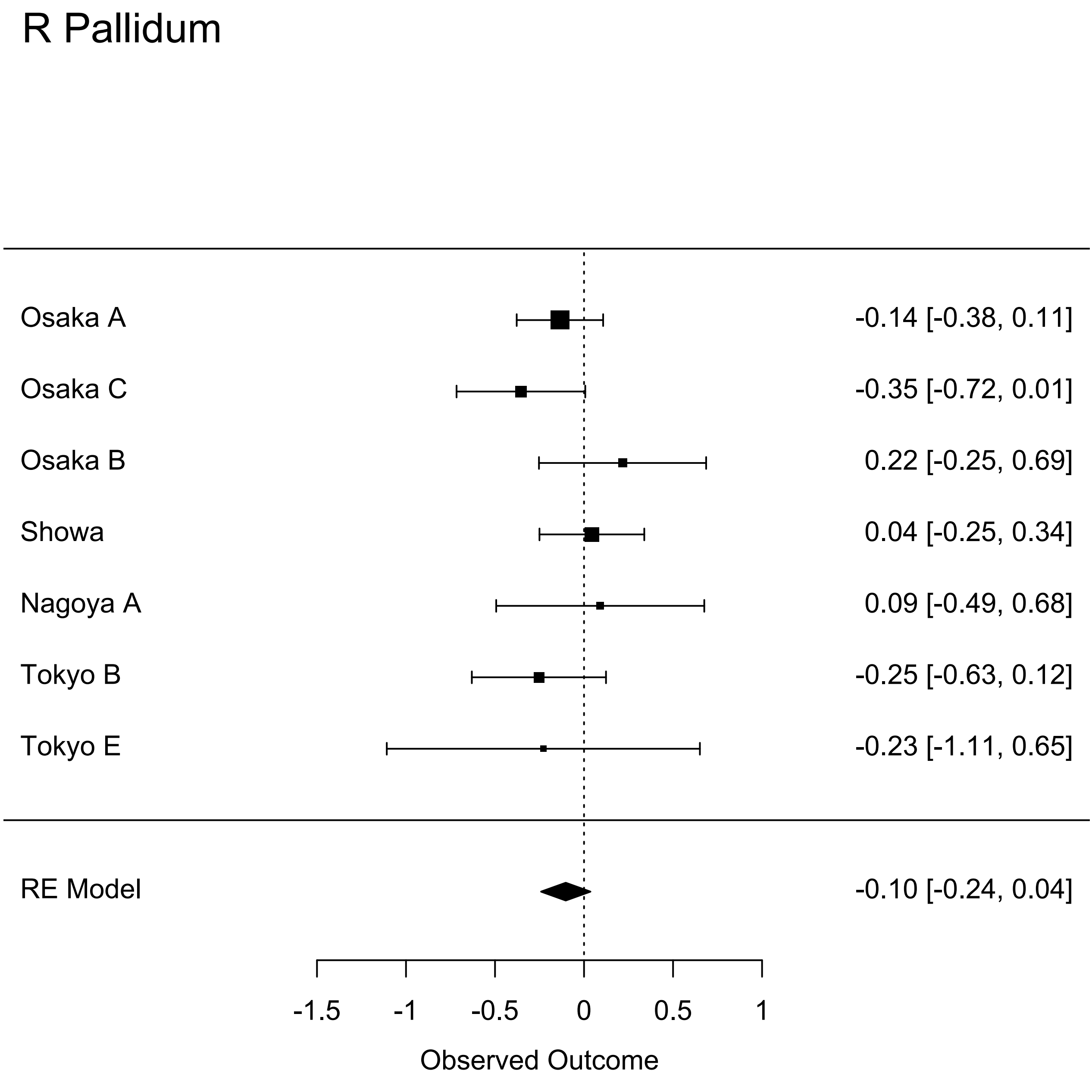
R Pallidum
Osaka A
-0.14 [-0.38, 0.11]
Osaka C
-0.35 [-0.72, 0.01]
Osaka B
0.22 [-0.25, 0.69]
Showa
0.04 [-0.25, 0.34]
Nagoya A
0.09 [-0.49, 0.68]
Tokyo B
-0.25 [-0.63, 0.12]
Tokyo E
-0.23 [-1.11, 0.65]
RE Model
-0.10 [-0.24, 0.04]
-1.5
-1
-0.5
0
0.5
1
Observed Outcome

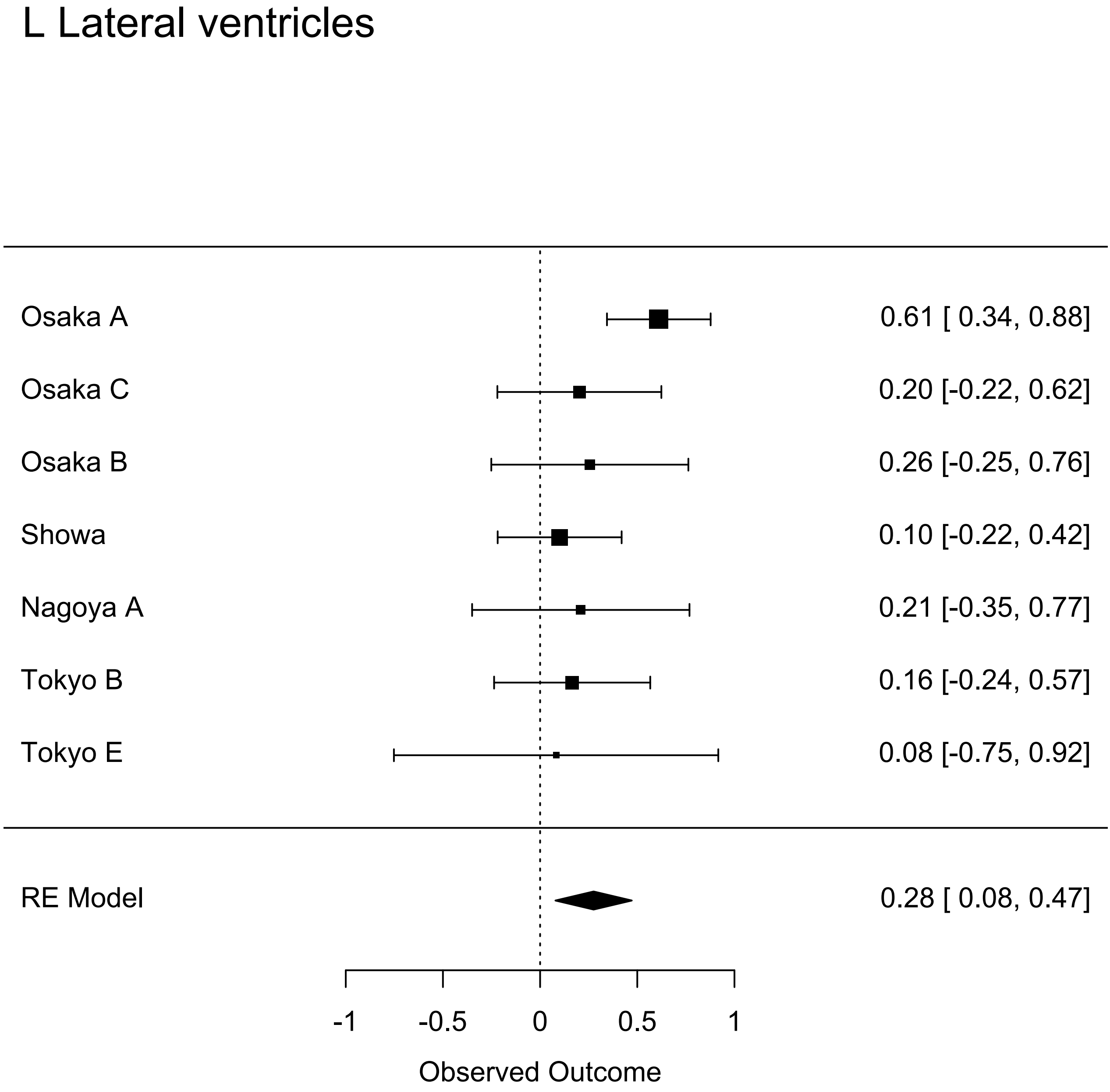
L Lateral ventricles
Osaka A
0.61 [0.34, 0.88]
Osaka C
0.20 [-0.22, 0.62]
Osaka B
0.26 [-0.25, 0.76]
Showa
0.10 [-0.22, 0.42]
Nagoya A
0.21 [-0.35, 0.77]
Tokyo B
0.16 [-0.24, 0.57]
Tokyo E
0.08 [-0.75, 0.92]
RE Model
0.28 [0.08, 0.47]
-1
-0.5
0
0.5
1
Observed Outcome

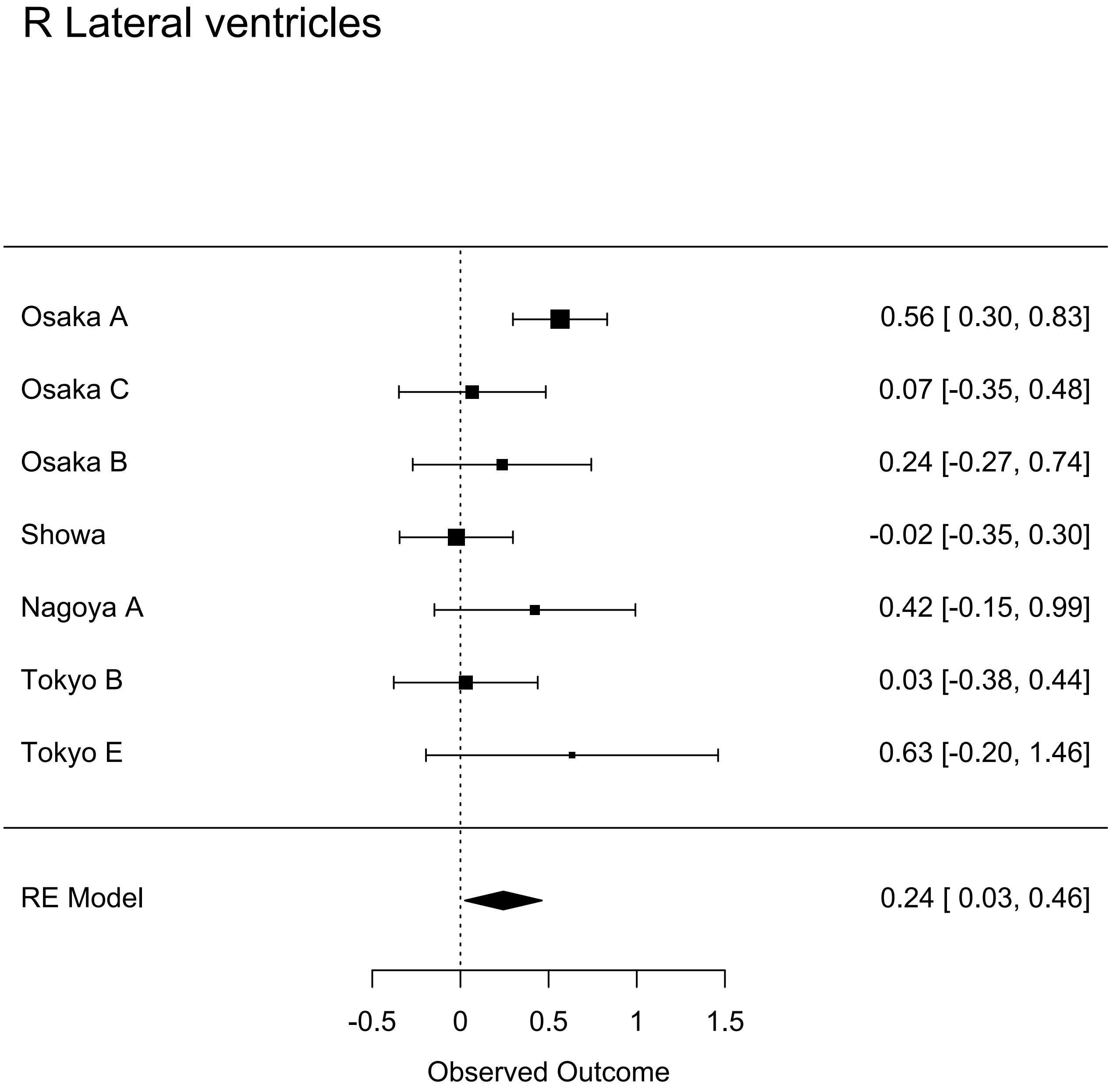
R Lateral ventricles
Osaka A
0.56 [0.30, 0.83]
Osaka C
0.07 [-0.35, 0.48]
Osaka B
0.24 [-0.27, 0.74]
Showa
-0.02 [-0.35, 0.30]
Nagoya A
0.42 [-0.15, 0.99]
Tokyo B
0.03 [-0.38, 0.44]
Tokyo E
0.63 [-0.20, 1.46]
RE Model
0.24 [0.03, 0.46]
-0.5
0
0.5
1
1.5
Observed Outcome

Supplementary Fig. 2: Mega-analytic forest plots for group differences in laterality indices of each regional volume between subjects with psychiatric disorders and healthy controls.

(a) Forest plots of all 24 included protocols evaluating effect sizes for group differences in laterality indices of each regional volume between subjects with schizophrenia and healthy controls. **(b)** Forest plots of all 10 included protocols evaluating effect sizes for group differences in laterality indices of each regional volume between subjects with bipolar disorder and healthy controls. **(c)** Forest plots of all 14 included protocols evaluating effect sizes for group differences in laterality indices of each regional volume between subjects with major depressive disorder and healthy controls. **(d)** Forest plots of all 7 included protocols evaluating effect sizes for group differences in laterality indices of each regional volume between subjects with autism spectrum disorder and healthy controls.

(a)

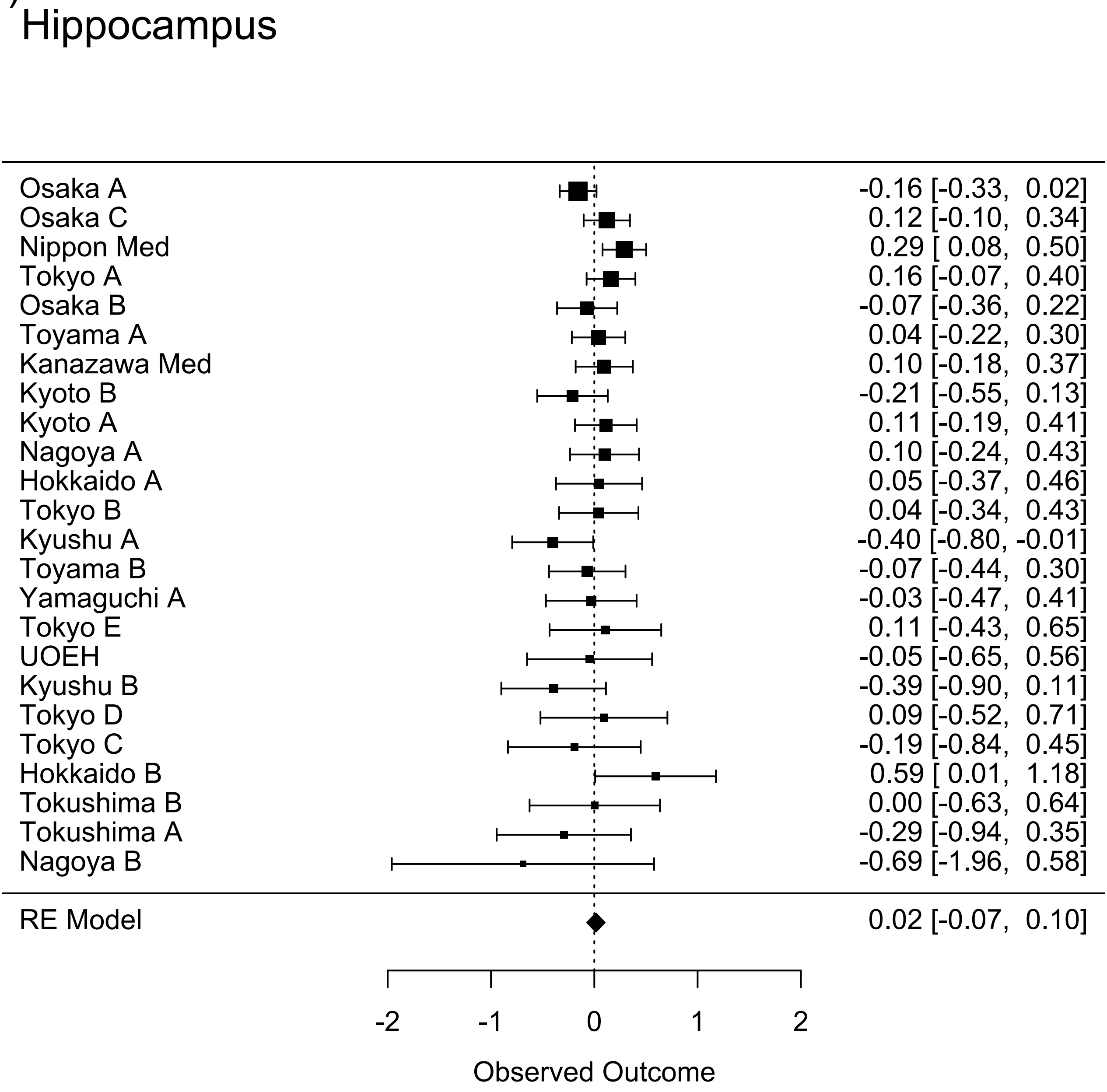
Hippocampus
Osaka A -0.16 [-0.33, 0.02]
Osaka C 0.12 [-0.10, 0.34]
Nippon Med 0.29 [0.08, 0.50]
Tokyo A 0.16 [-0.07, 0.40]
Osaka B -0.07 [-0.36, 0.22]
Toyama A 0.04 [-0.22, 0.30]
Kanazawa Med 0.10 [-0.18, 0.37]
Kyoto B -0.21 [-0.55, 0.13]
Kyoto A 0.11 [-0.19, 0.41]
Nagoya A 0.10 [-0.24, 0.43]
Hokkaido A 0.05 [-0.37, 0.46]
Tokyo B 0.04 [-0.34, 0.43]
Kyushu A -0.40 [-0.80, -0.01]
Toyama B -0.07 [-0.44, 0.30]
Yamaguchi A -0.03 [-0.47, 0.41]
Tokyo E 0.11 [-0.43, 0.65]
UOEH -0.05 [-0.65, 0.56]
Kyushu B -0.39 [-0.90, 0.11]
Tokyo D 0.09 [-0.52, 0.71]
Tokyo C -0.19 [-0.84, 0.45]
Hokkaido B 0.59 [0.01, 1.18]
Tokushima B 0.00 [-0.63, 0.64]
Tokushima A -0.29 [-0.94, 0.35]
Nagoya B -0.69 [-1.96, 0.58]
RE Model 0.02 [-0.07, 0.10]
-2
-1
0
1
2
Observed Outcome

Amygdala

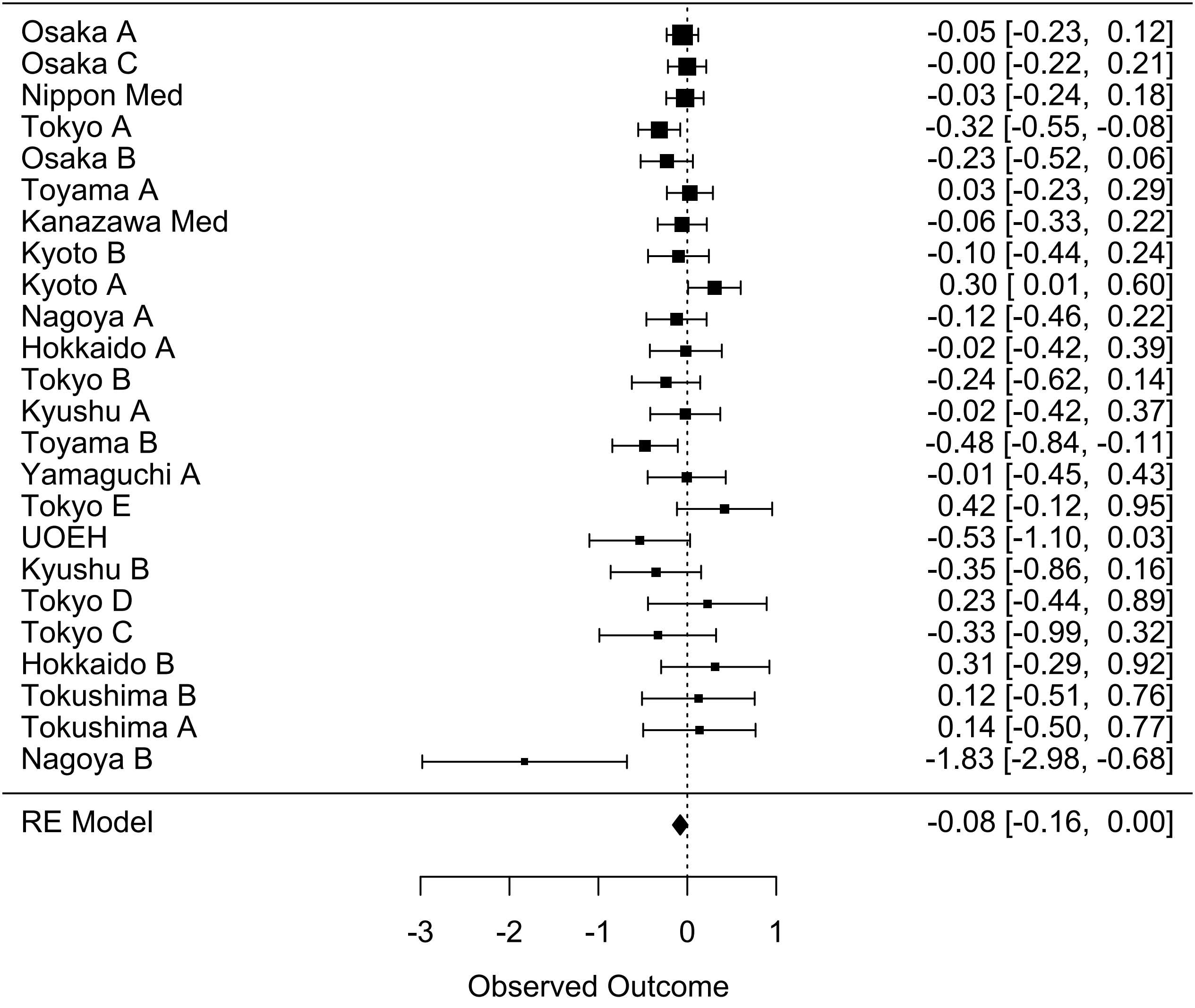

Osaka A -0.05 [-0.23, 0.12]
Osaka C -0.00 [-0.22, 0.21]
Nippon Med -0.03 [-0.24, 0.18]
Tokyo A -0.32 [-0.55, -0.08]
Osaka B -0.23 [-0.52, 0.06]
Toyama A 0.03 [-0.23, 0.29]
Kanazawa Med -0.06 [-0.33, 0.22]
Kyoto B -0.10 [-0.44, 0.24]
Kyoto A 0.30 [0.01, 0.60]
Nagoya A -0.12 [-0.46, 0.22]
Hokkaido A -0.02 [-0.42, 0.39]
Tokyo B -0.24 [-0.62, 0.14]
Kyushu A -0.02 [-0.42, 0.37]
Toyama B -0.48 [-0.84, -0.11]
Yamaguchi A -0.01 [-0.45, 0.43]
Tokyo E 0.42 [-0.12, 0.95]
UOEH -0.53 [-1.10, 0.03]
Kyushu B -0.35 [-0.86, 0.16]
Tokyo D 0.23 [-0.44, 0.89]
Tokyo C -0.33 [-0.99, 0.32]
Hokkaido B 0.31 [-0.29, 0.92]
Tokushima B 0.12 [-0.51, 0.76]
Tokushima A 0.14 [-0.50, 0.77]
Nagoya B -1.83 [-2.98, -0.68]
RE Model -0.08 [-0.16, 0.00]
-3
-2
-1
0
1
Observed Outcome

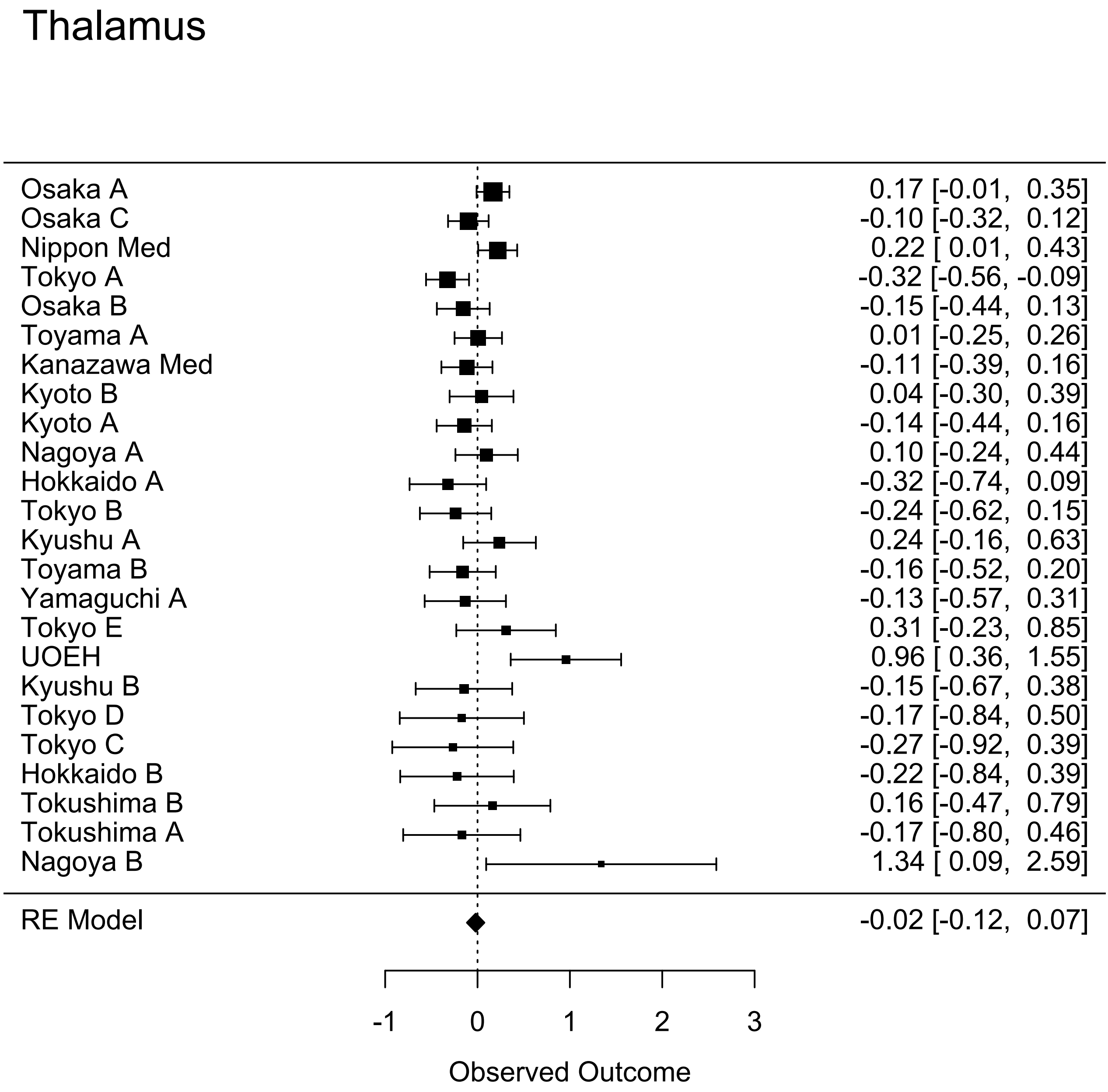
Thalamus
Osaka A 0.17 [-0.01, 0.35]
Osaka C -0.10 [-0.32, 0.12]
Nippon Med 0.22 [0.01, 0.43]
Tokyo A -0.32 [-0.56, -0.09]
Osaka B -0.15 [-0.44, 0.13]
Toyama A 0.01 [-0.25, 0.26]
Kanazawa Med -0.11 [-0.39, 0.16]
Kyoto B 0.04 [-0.30, 0.39]
Kyoto A -0.14 [-0.44, 0.16]
Nagoya A 0.10 [-0.24, 0.44]
Hokkaido A -0.32 [-0.74, 0.09]
Tokyo B -0.24 [-0.62, 0.15]
Kyushu A 0.24 [-0.16, 0.63]
Toyama B -0.16 [-0.52, 0.20]
Yamaguchi A -0.13 [-0.57, 0.31]
Tokyo E 0.31 [-0.23, 0.85]
UOEH 0.96 [0.36, 1.55]
Kyushu B -0.15 [-0.67, 0.38]
Tokyo D -0.17 [-0.84, 0.50]
Tokyo C -0.27 [-0.92, 0.39]
Hokkaido B -0.22 [-0.84, 0.39]
Tokushima B 0.16 [-0.47, 0.79]
Tokushima A -0.17 [-0.80, 0.46]
Nagoya B 1.34 [0.09, 2.59]
RE Model -0.02 [-0.12, 0.07]
-1
0
1
2
3
Observed Outcome

Accumbens

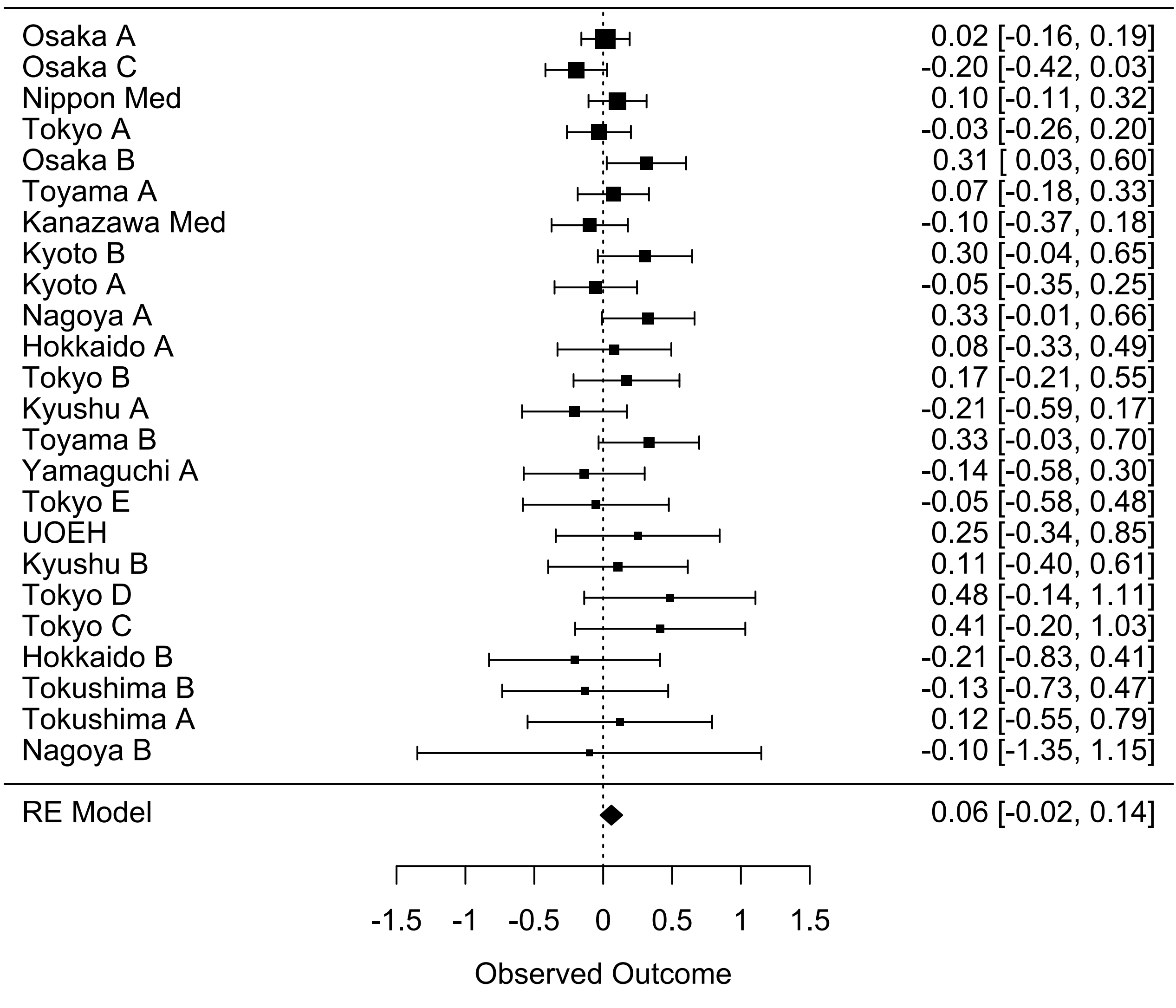
Osaka A 0.02 [-0.16, 0.19]
Osaka C -0.20 [-0.42, 0.03]
Nippon Med 0.10 [-0.11, 0.32]
Tokyo A -0.03 [-0.26, 0.20]
Osaka B 0.31 [0.03, 0.60]
Toyama A 0.07 [-0.18, 0.33]
Kanazawa Med -0.10 [-0.37, 0.18]
Kyoto B 0.30 [-0.04, 0.65]
Kyoto A -0.05 [-0.35, 0.25]
Nagoya A 0.33 [-0.01, 0.66]
Hokkaido A 0.08 [-0.33, 0.49]
Tokyo B 0.17 [-0.21, 0.55]
Kyushu A -0.21 [-0.59, 0.17]
Toyama B 0.33 [-0.03, 0.70]
Yamaguchi A -0.14 [-0.58, 0.30]
Tokyo E -0.05 [-0.58, 0.48]
UOEH 0.25 [-0.34, 0.85]
Kyushu B 0.11 [-0.40, 0.61]
Tokyo D 0.48 [-0.14, 1.11]
Tokyo C 0.41 [-0.20, 1.03]
Hokkaido B -0.21 [-0.83, 0.41]
Tokushima B -0.13 [-0.73, 0.47]
Tokushima A 0.12 [-0.55, 0.79]
Nagoya B -0.10 [-1.35, 1.15]
RE Model 0.06 [-0.02, 0.14]
-1.5
-1
-0.5
0
0.5
1
1.5
Observed Outcome

Caudate

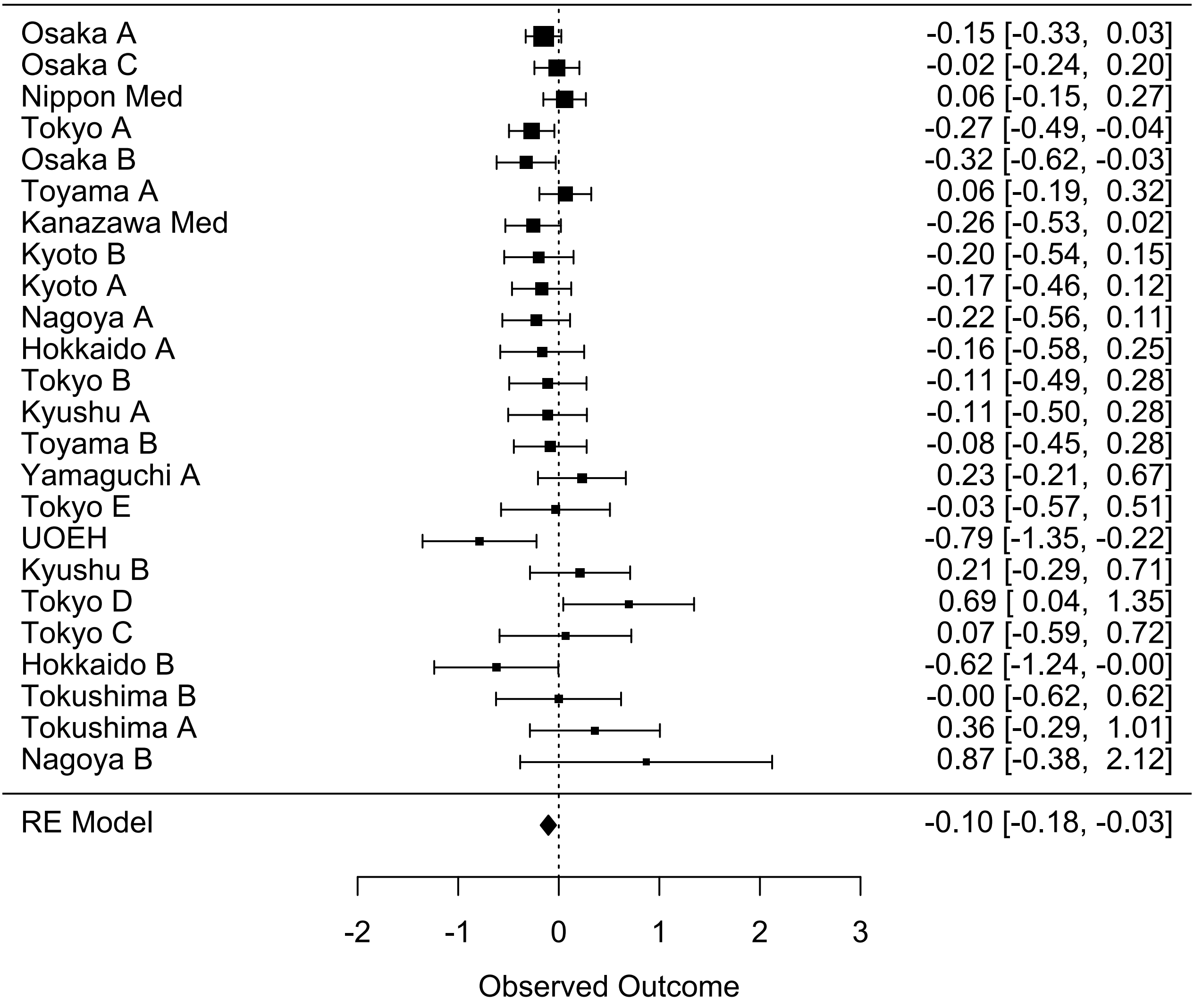

Osaka A -0.15 [-0.33, 0.03]
Osaka C -0.02 [-0.24, 0.20]
Nippon Med 0.06 [-0.15, 0.27]
Tokyo A -0.27 [-0.49, -0.04]
Osaka B -0.32 [-0.62, -0.03]
Toyama A 0.06 [-0.19, 0.32]
Kanazawa Med -0.26 [-0.53, 0.02]
Kyoto B -0.20 [-0.54, 0.15]
Kyoto A -0.17 [-0.46, 0.12]
Nagoya A -0.22 [-0.56, 0.11]
Hokkaido A -0.16 [-0.58, 0.25]
Tokyo B -0.11 [-0.49, 0.28]
Kyushu A -0.11 [-0.50, 0.28]
Toyama B -0.08 [-0.45, 0.28]
Yamaguchi A 0.23 [-0.21, 0.67]
Tokyo E -0.03 [-0.57, 0.51]
UOEH -0.79 [-1.35, -0.22]
Kyushu B 0.21 [-0.29, 0.71]
Tokyo D 0.69 [0.04, 1.35]
Tokyo C 0.07 [-0.59, 0.72]
Hokkaido B -0.62 [-1.24, -0.00]
Tokushima B -0.00 [-0.62, 0.62]
Tokushima A 0.36 [-0.29, 1.01]
Nagoya B 0.87 [-0.38, 2.12]
RE Model -0.10 [-0.18, -0.03]
-2
-1
0
1
2
3
Observed Outcome

Putamen

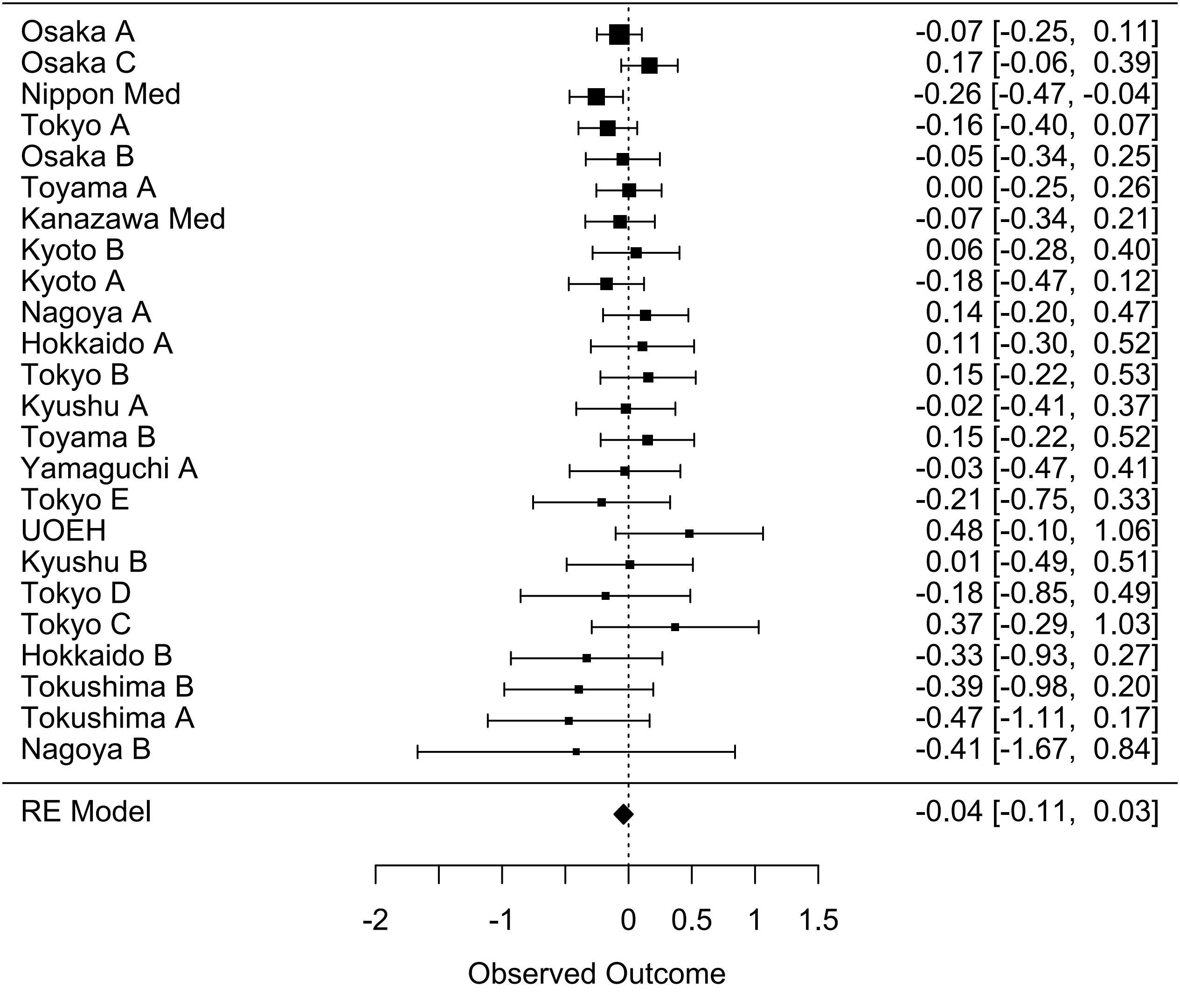

Osaka A -0.07 [-0.25, 0.11]
Osaka C 0.17 [-0.06, 0.39]
Nippon Med -0.26 [-0.47, -0.04]
Tokyo A -0.16 [-0.40, 0.07]
Osaka B -0.05 [-0.34, 0.25]
Toyama A 0.00 [-0.25, 0.26]
Kanazawa Med -0.07 [-0.34, 0.21]
Kyoto B 0.06 [-0.28, 0.40]
Kyoto A -0.18 [-0.47, 0.12]
Nagoya A 0.14 [-0.20, 0.47]
Hokkaido A 0.11 [-0.30, 0.52]
Tokyo B 0.15 [-0.22, 0.53]
Kyushu A -0.02 [-0.41, 0.37]
Toyama B 0.15 [-0.22, 0.52]
Yamaguchi A -0.03 [-0.47, 0.41]
Tokyo E -0.21 [-0.75, 0.33]
UOEH 0.48 [-0.10, 1.06]
Kyushu B 0.01 [-0.49, 0.51]
Tokyo D -0.18 [-0.85, 0.49]
Tokyo C 0.37 [-0.29, 1.03]
Hokkaido B -0.33 [-0.93, 0.27]
Tokushima B -0.39 [-0.98, 0.20]
Tokushima A -0.47 [-1.11, 0.17]
Nagoya B -0.41 [-1.67, 0.84]
RE Model -0.04 [-0.11, 0.03]
-2
-1
0
0.5
1
1.5
Observed Outcome

Pallidum

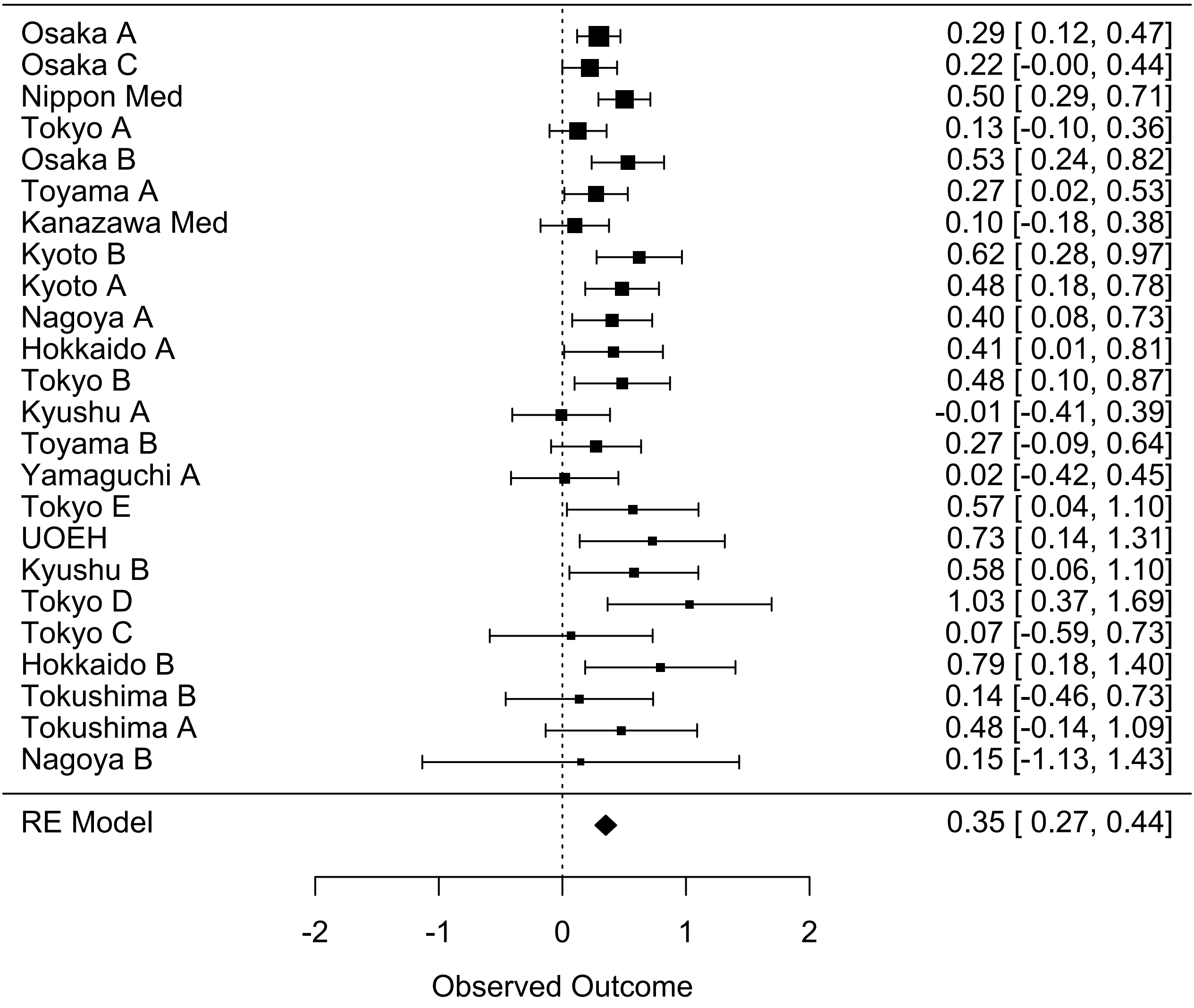

Osaka A 0.29 [0.12, 0.47]
Osaka C 0.22 [-0.00, 0.44]
Nippon Med 0.50 [0.29, 0.71]
Tokyo A 0.13 [-0.10, 0.36]
Osaka B 0.53 [0.24, 0.82]
Toyama A 0.27 [0.02, 0.53]
Kanazawa Med 0.10 [-0.18, 0.38]
Kyoto B 0.62 [0.28, 0.97]
Kyoto A 0.48 [0.18, 0.78]
Nagoya A 0.40 [0.08, 0.73]
Hokkaido A 0.41 [0.01, 0.81]
Tokyo B 0.48 [0.10, 0.87]
Kyushu A -0.01 [-0.41, 0.39]
Toyama B 0.27 [-0.09, 0.64]
Yamaguchi A 0.02 [-0.42, 0.45]
Tokyo E 0.57 [0.04, 1.10]
UOEH 0.73 [0.14, 1.31]
Kyushu B 0.58 [0.06, 1.10]
Tokyo D 1.03 [0.37, 1.69]
Tokyo C 0.07 [-0.59, 0.73]
Hokkaido B 0.79 [0.18, 1.40]
Tokushima B 0.14 [-0.46, 0.73]
Tokushima A 0.48 [-0.14, 1.09]
Nagoya B 0.15 [-1.13, 1.43]
RE Model 0.35 [0.27, 0.44]
-2
-1
0
1
2
Observed Outcome

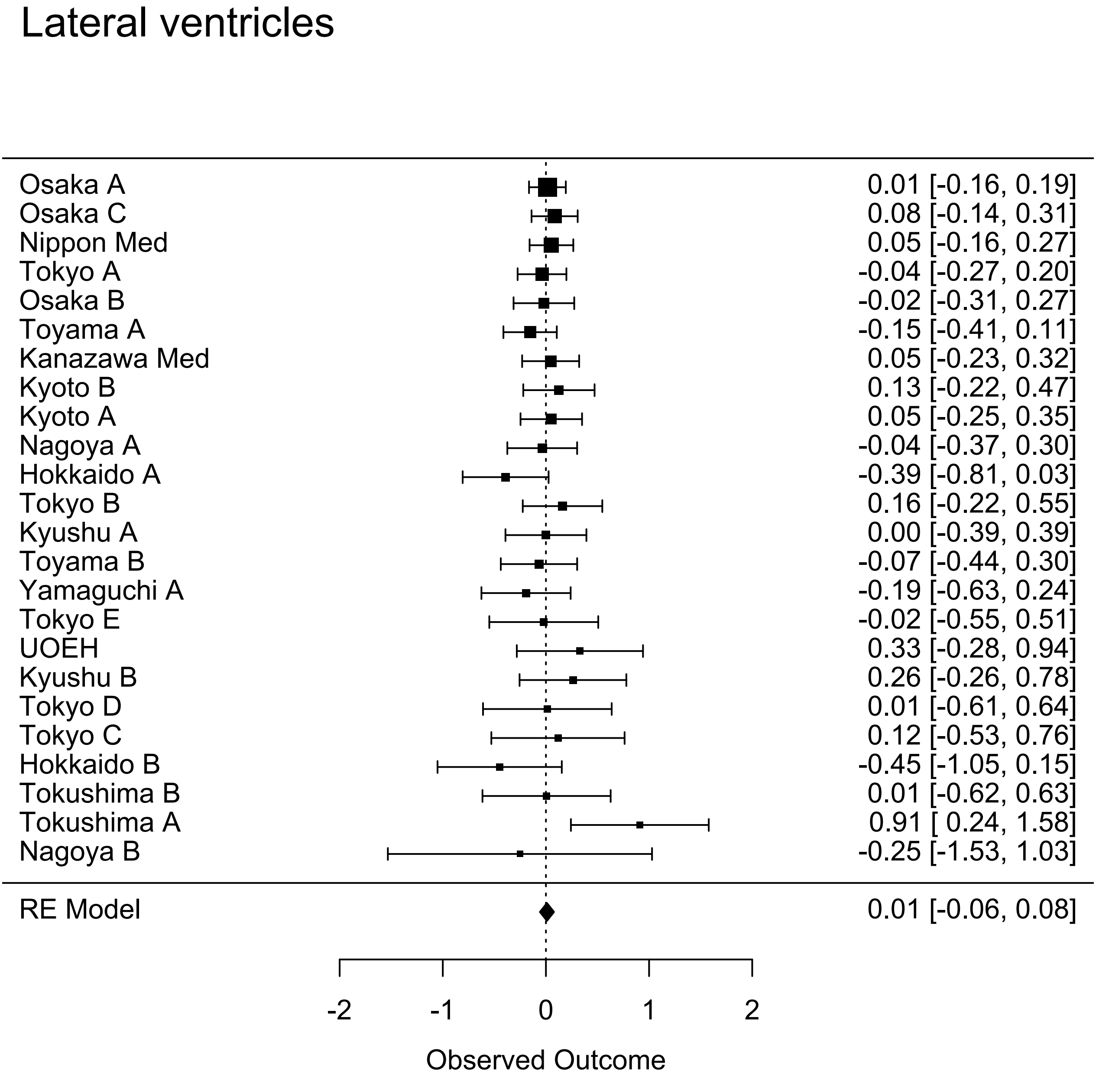
Lateral ventricles
Osaka A 0.01 [-0.16, 0.19]
Osaka C 0.08 [-0.14, 0.31]
Nippon Med 0.05 [-0.16, 0.27]
Tokyo A -0.04 [-0.27, 0.20]
Osaka B -0.02 [-0.31, 0.27]
Toyama A -0.15 [-0.41, 0.11]
Kanazawa Med 0.05 [-0.23, 0.32]
Kyoto B 0.13 [-0.22, 0.47]
Kyoto A 0.05 [-0.25, 0.35]
Nagoya A -0.04 [-0.37, 0.30]
Hokkaido A -0.39 [-0.81, 0.03]
Tokyo B 0.16 [-0.22, 0.55]
Kyushu A 0.00 [-0.39, 0.39]
Toyama B -0.07 [-0.44, 0.30]
Yamaguchi A -0.19 [-0.63, 0.24]
Tokyo E -0.02 [-0.55, 0.51]
UOEH 0.33 [-0.28, 0.94]
Kyushu B 0.26 [-0.26, 0.78]
Tokyo D 0.01 [-0.61, 0.64]
Tokyo C 0.12 [-0.53, 0.76]
Hokkaido B -0.45 [-1.05, 0.15]
Tokushima B 0.01 [-0.62, 0.63]
Tokushima A 0.91 [0.24, 1.58]
Nagoya B -0.25 [-1.53, 1.03]
RE Model 0.01 [-0.06, 0.08]
-2
-1
0
1
2
Observed Outcome

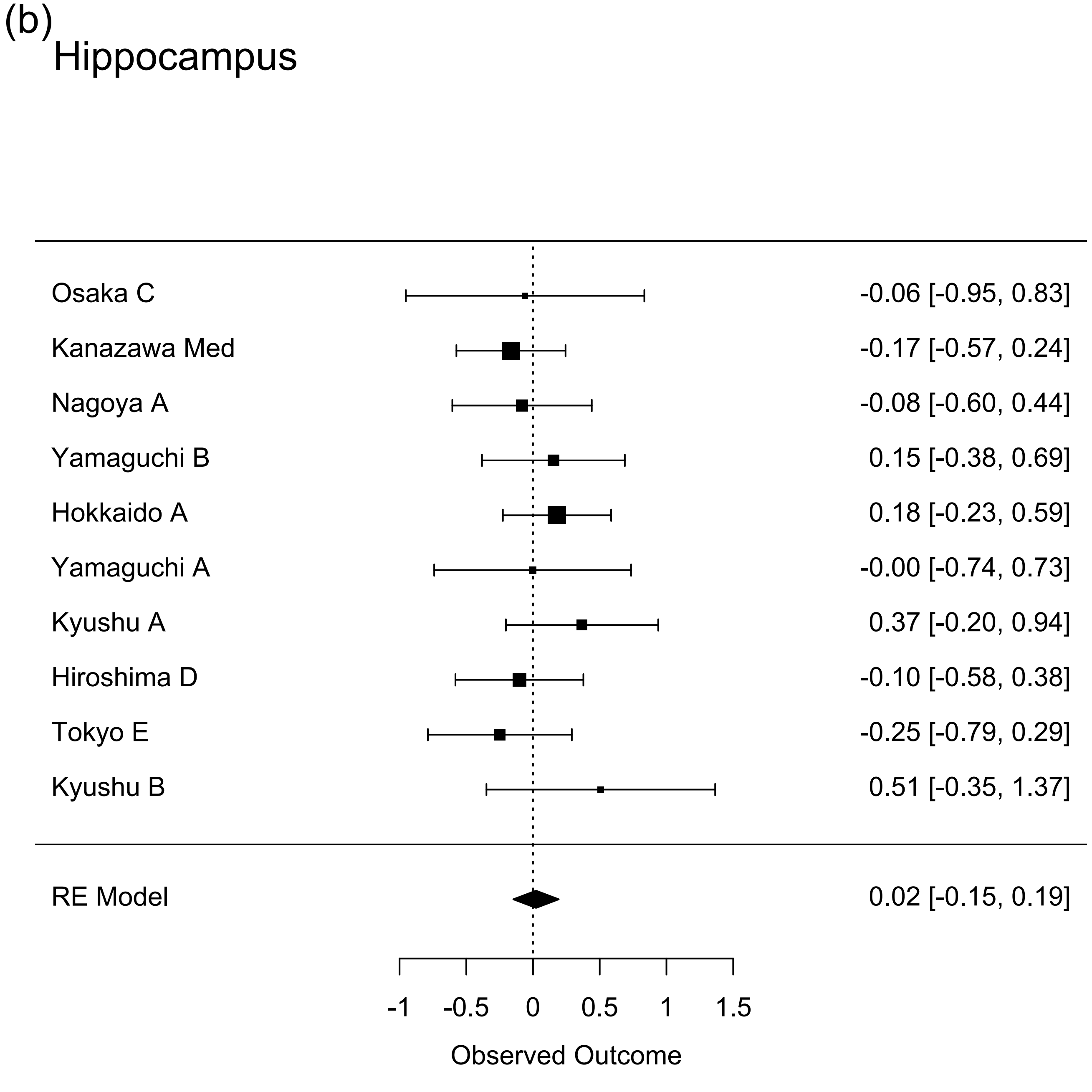
(b)
Hippocampus
Osaka C
-0.06 [-0.95, 0.83]
Kanazawa Med
-0.17 [-0.57, 0.24]
Nagoya A
-0.08 [-0.60, 0.44]
Yamaguchi B
0.15 [-0.38, 0.69]
Hokkaido A
0.18 [-0.23, 0.59]
Yamaguchi A
-0.00 [-0.74, 0.73]
Kyushu A
0.37 [-0.20, 0.94]
Hiroshima D
-0.10 [-0.58, 0.38]
Tokyo E
-0.25 [-0.79, 0.29]
Kyushu B
0.51 [-0.35, 1.37]
RE Model
0.02 [-0.15, 0.19]
-1
-0.5
0
0.5
1
1.5
Observed Outcome

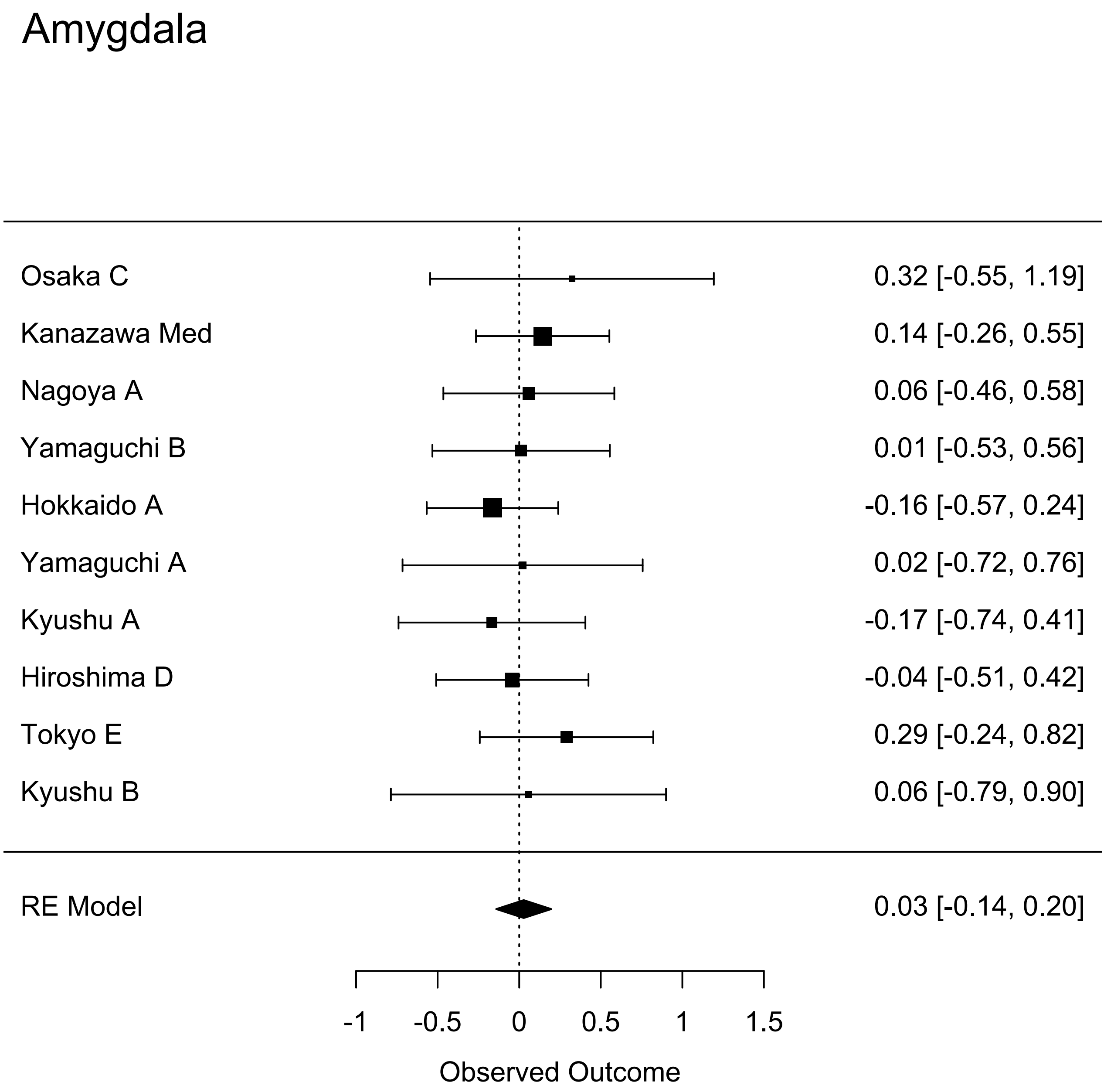
Amygdala
Osaka C
0.32 [-0.55, 1.19]
Kanazawa Med
0.14 [-0.26, 0.55]
Nagoya A
0.06 [-0.46, 0.58]
Yamaguchi B
0.01 [-0.53, 0.56]
Hokkaido A
-0.16 [-0.57, 0.24]
Yamaguchi A
0.02 [-0.72, 0.76]
Kyushu A
-0.17 [-0.74, 0.41]
Hiroshima D
-0.04 [-0.51, 0.42]
Tokyo E
0.29 [-0.24, 0.82]
Kyushu B
0.06 [-0.79, 0.90]
RE Model
0.03 [-0.14, 0.20]
-1
-0.5
0
0.5
1
1.5
Observed Outcome

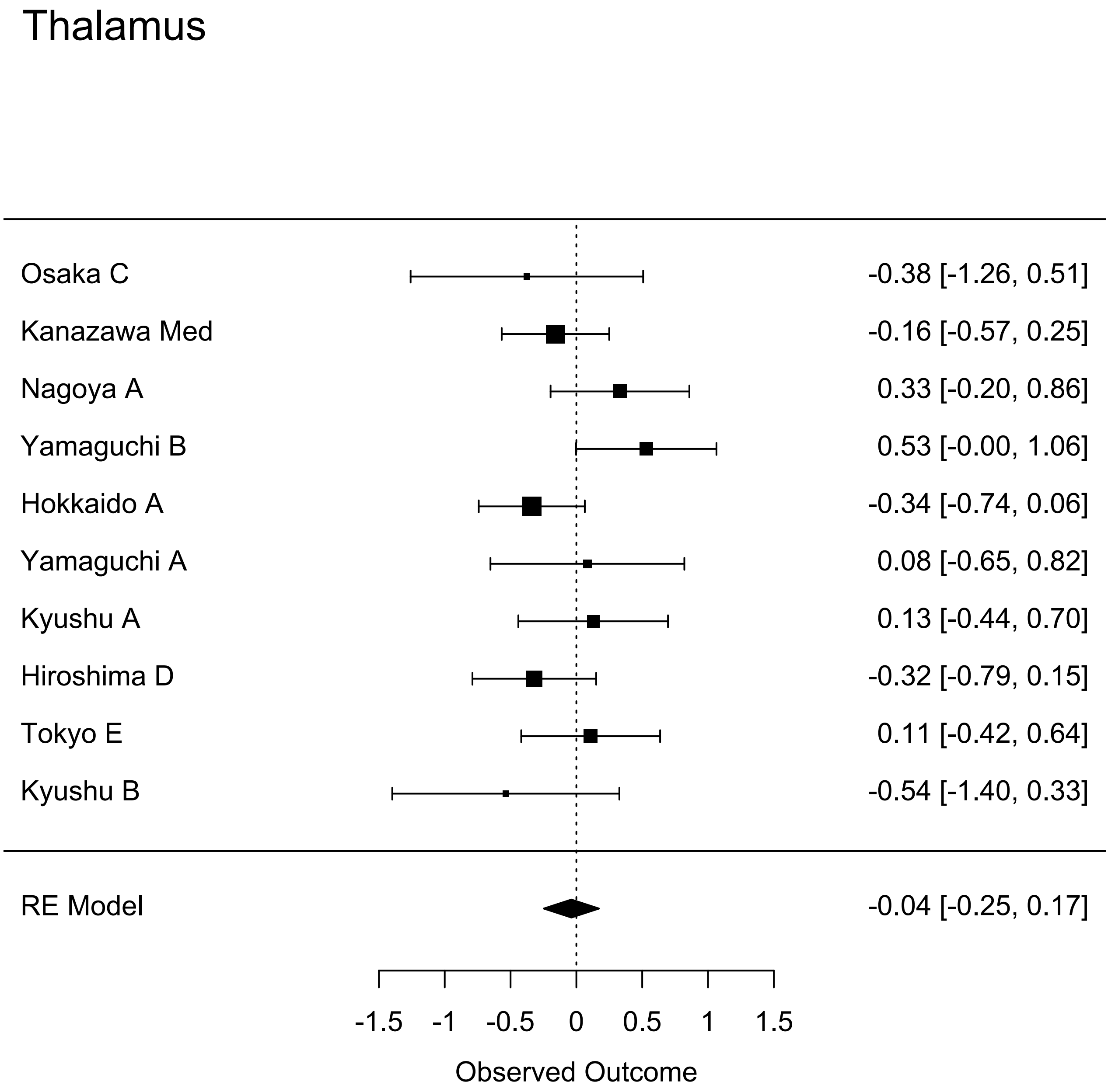
Thalamus
Osaka C
-0.38 [-1.26, 0.51]
Kanazawa Med
-0.16 [-0.57, 0.25]
Nagoya A
0.33 [-0.20, 0.86]
Yamaguchi B
0.53 [-0.00, 1.06]
Hokkaido A
-0.34 [-0.74, 0.06]
Yamaguchi A
0.08 [-0.65, 0.82]
Kyushu A
0.13 [-0.44, 0.70]
Hiroshima D
-0.32 [-0.79, 0.15]
Tokyo E
0.11 [-0.42, 0.64]
Kyushu B
-0.54 [-1.40, 0.33]
RE Model
-0.04 [-0.25, 0.17]
-1.5
-1
-0.5
0
0.5
1
1.5
Observed Outcome

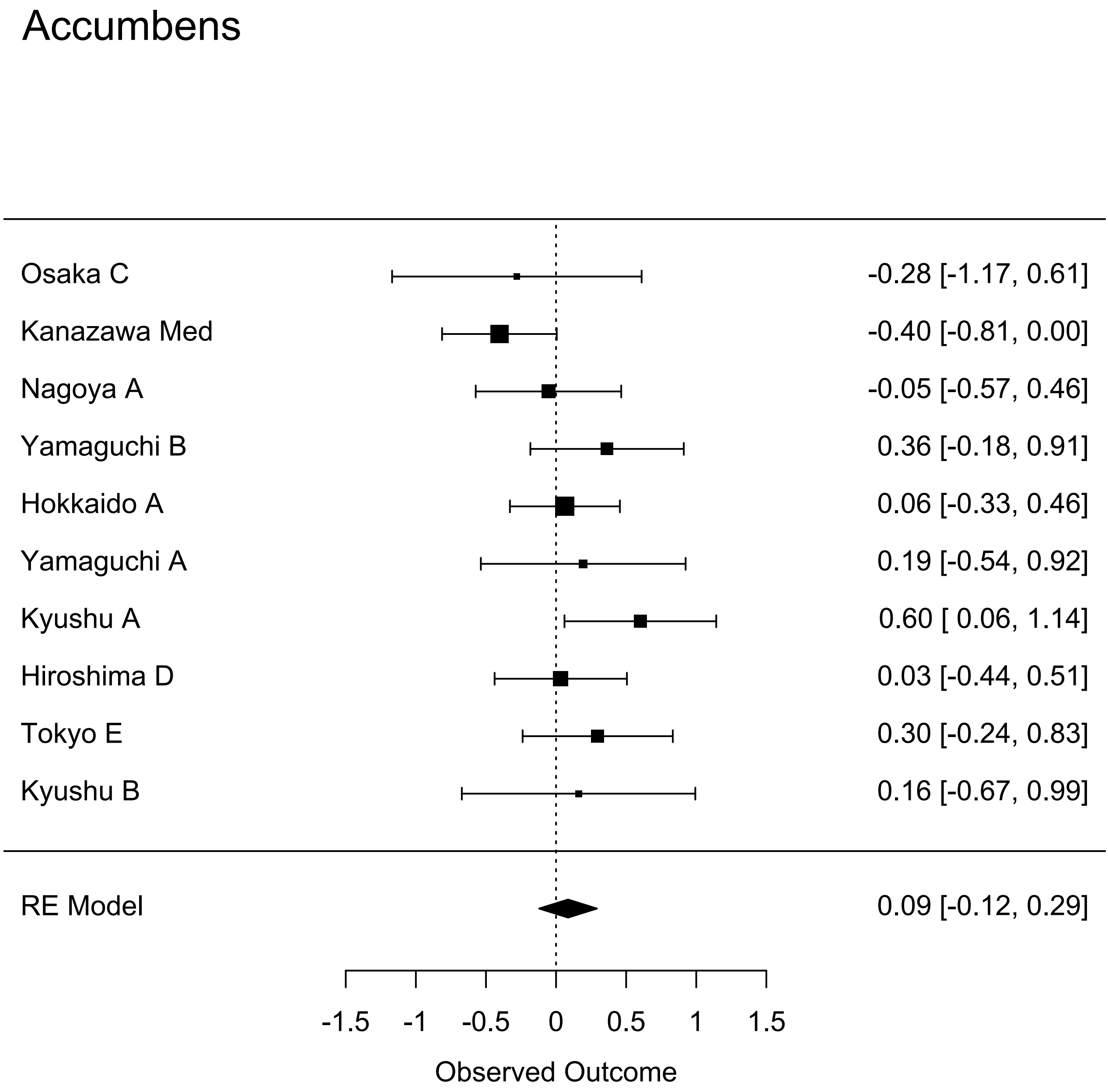
Accumbens
Osaka C
Kanazawa Med
Nagoya A
Yamaguchi B
Hokkaido A
Yamaguchi A
Kyushu A
Hiroshima D
Tokyo E
Kyushu B
RE Model
-0.28 [-1.17, 0.61]
-0.40 [-0.81, 0.00]
-0.05 [-0.57, 0.46]
0.36 [-0.18, 0.91]
0.06 [-0.33, 0.46]
0.19 [-0.54, 0.92]
0.60 [0.06, 1.14]
0.03 [-0.44, 0.51]
0.30 [-0.24, 0.83]
0.16 [-0.67, 0.99]
0.09 [-0.12, 0.29]
-1.5
-1
-0.5
0
0.5
1
1.5
Observed Outcome

Caudate

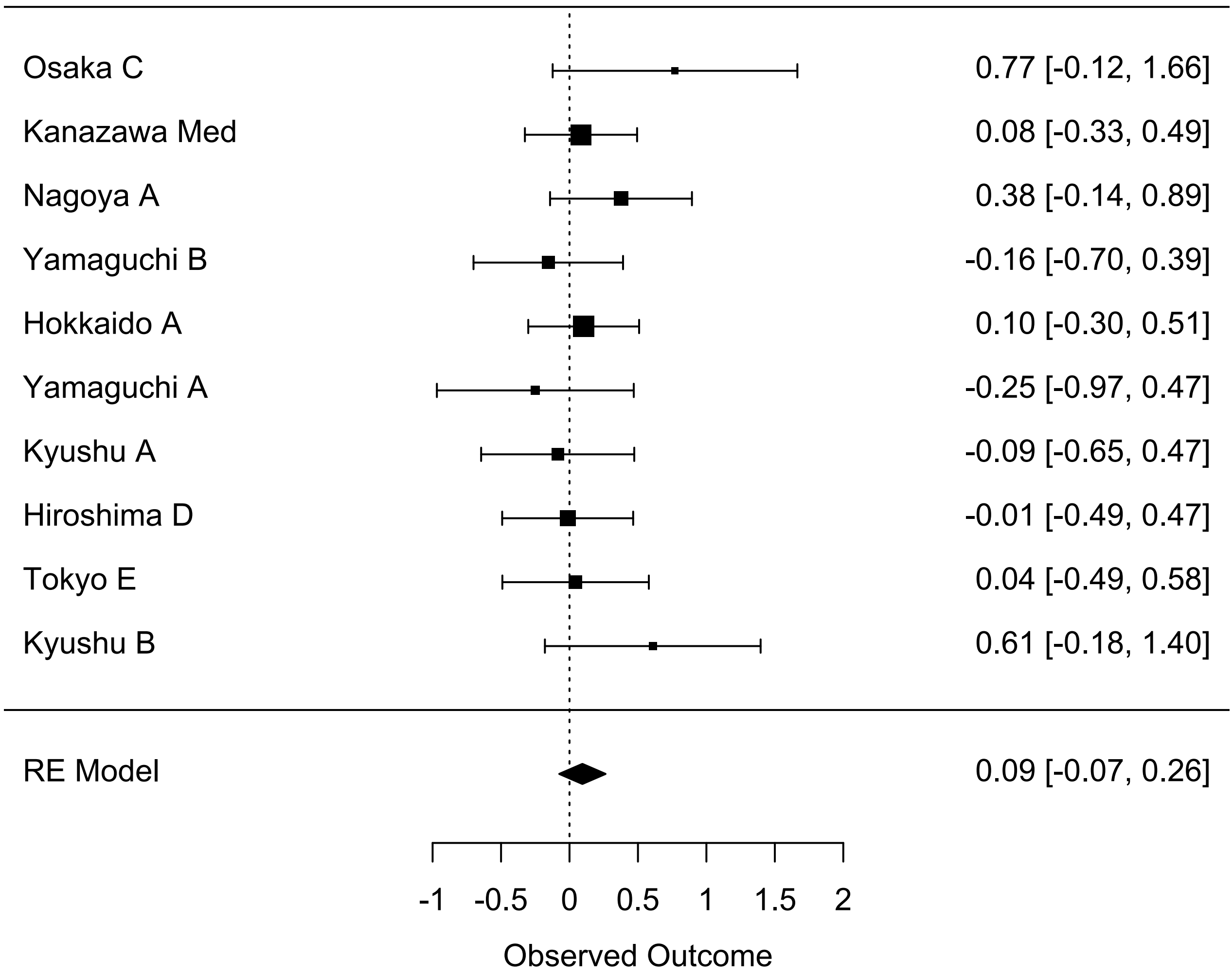
Osaka C
0.77 [-0.12, 1.66]
Kanazawa Med
0.08 [-0.33, 0.49]
Nagoya A
0.38 [-0.14, 0.89]
Yamaguchi B
-0.16 [-0.70, 0.39]
Hokkaido A
0.10 [-0.30, 0.51]
Yamaguchi A
-0.25 [-0.97, 0.47]
Kyushu A
-0.09 [-0.65, 0.47]
Hiroshima D
-0.01 [-0.49, 0.47]
Tokyo E
0.04 [-0.49, 0.58]
Kyushu B
0.61 [-0.18, 1.40]
RE Model
0.09 [-0.07, 0.26]
-1
-0.5
0
0.5
1
1.5
2
Observed Outcome

Putamen

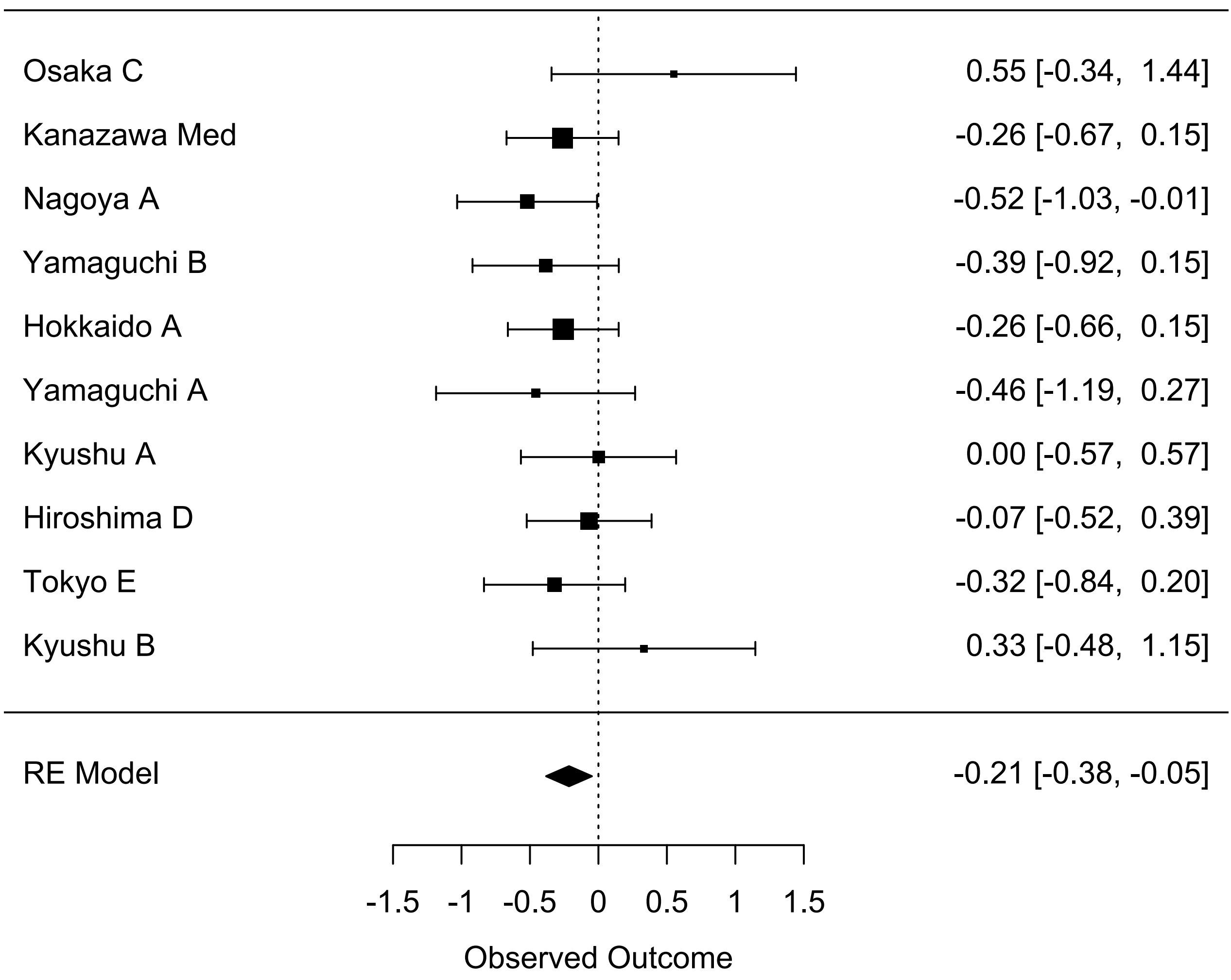
Osaka C
0.55 [-0.34, 1.44]
Kanazawa Med
-0.26 [-0.67, 0.15]
Nagoya A
-0.52 [-1.03, -0.01]
Yamaguchi B
-0.39 [-0.92, 0.15]
Hokkaido A
-0.26 [-0.66, 0.15]
Yamaguchi A
-0.46 [-1.19, 0.27]
Kyushu A
0.00 [-0.57, 0.57]
Hiroshima D
-0.07 [-0.52, 0.39]
Tokyo E
-0.32 [-0.84, 0.20]
Kyushu B
0.33 [-0.48, 1.15]
RE Model
-0.21 [-0.38, -0.05]
-1.5
-1
-0.5
0
0.5
1
1.5
Observed Outcome

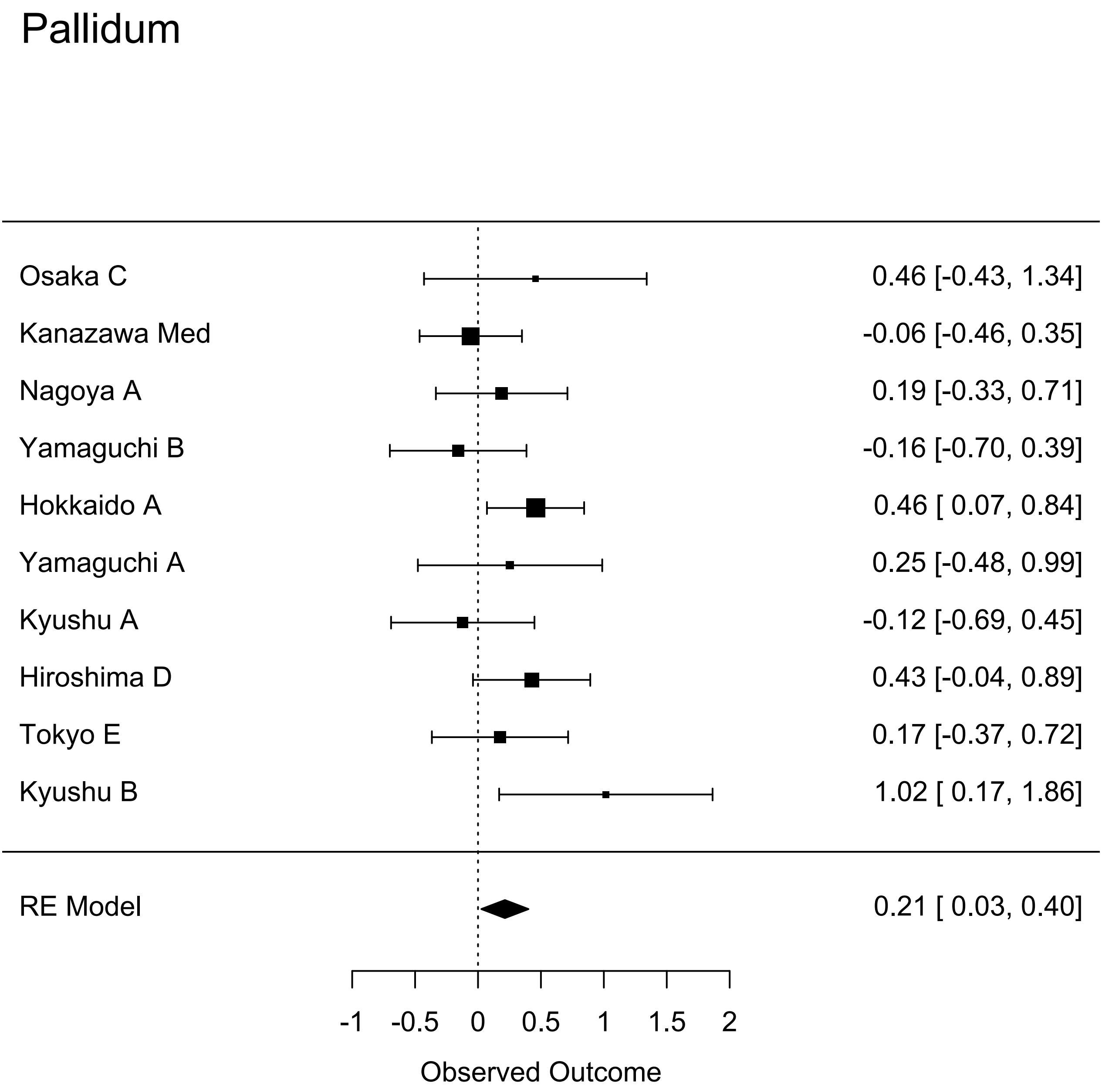

Pallidum
Osaka C
Kanazawa Med
Nagoya A
Yamaguchi B
Hokkaido A
Yamaguchi A
Kyushu A
Hiroshima D
Tokyo E
Kyushu B
RE Model
0.46 [-0.43, 1.34]
-0.06 [-0.46, 0.35]
0.19 [-0.33, 0.71]
-0.16 [-0.70, 0.39]
0.46 [0.07, 0.84]
0.25 [-0.48, 0.99]
-0.12 [-0.69, 0.45]
0.43 [-0.04, 0.89]
0.17 [-0.37, 0.72]
1.02 [0.17, 1.86]
0.21 [0.03, 0.40]
-1
-0.5
0
0.5
1
1.5
2
Observed Outcome

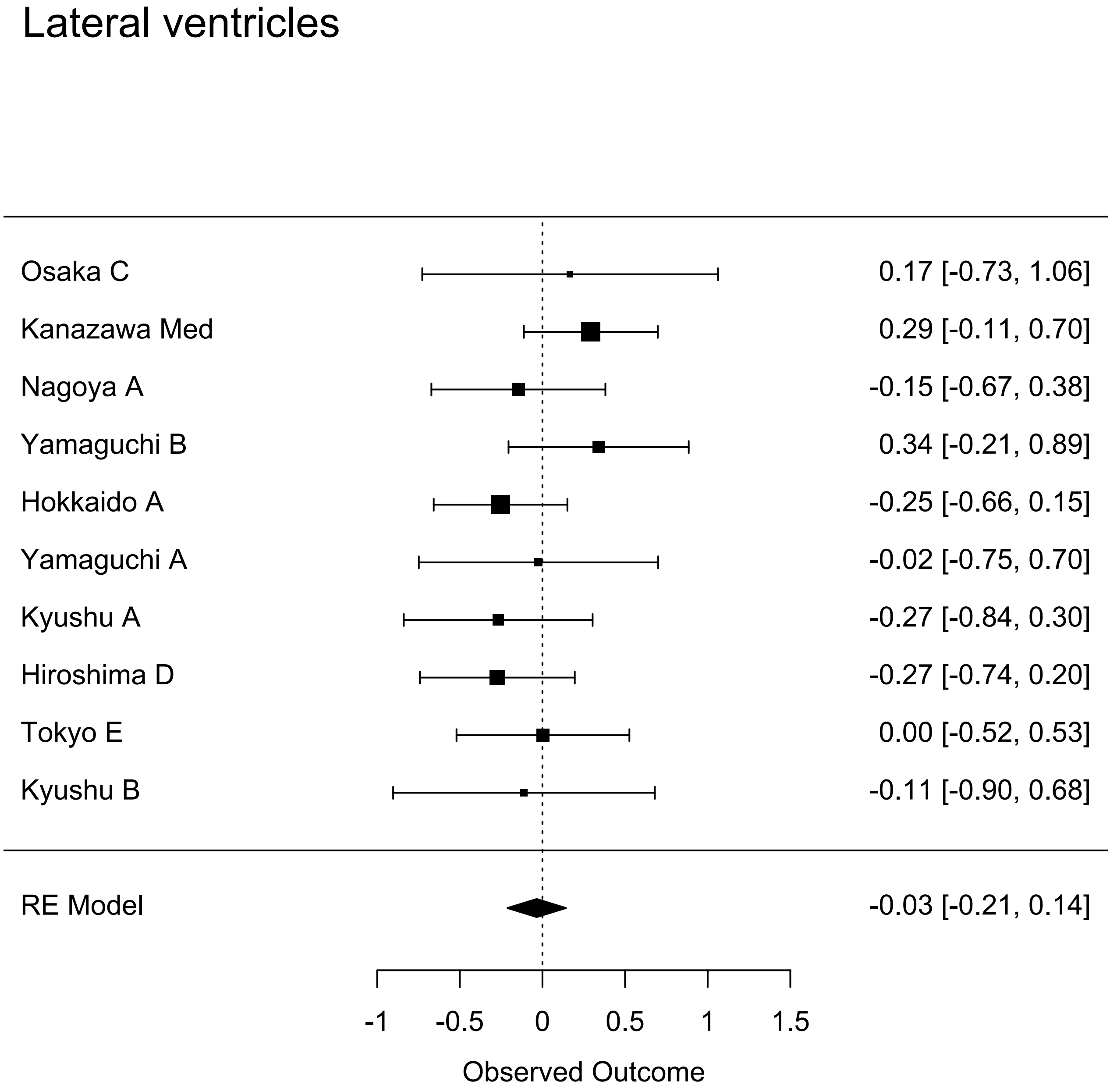
Lateral ventricles
Osaka C
0.17 [-0.73, 1.06]
Kanazawa Med
0.29 [-0.11, 0.70]
Nagoya A
-0.15 [-0.67, 0.38]
Yamaguchi B
0.34 [-0.21, 0.89]
Hokkaido A
-0.25 [-0.66, 0.15]
Yamaguchi A
-0.02 [-0.75, 0.70]
Kyushu A
-0.27 [-0.84, 0.30]
Hiroshima D
-0.27 [-0.74, 0.20]
Tokyo E
0.00 [-0.52, 0.53]
Kyushu B
-0.11 [-0.90, 0.68]
RE Model
-0.03 [-0.21, 0.14]
-1
-0.5
0
0.5
1
1.5
Observed Outcome

(c)

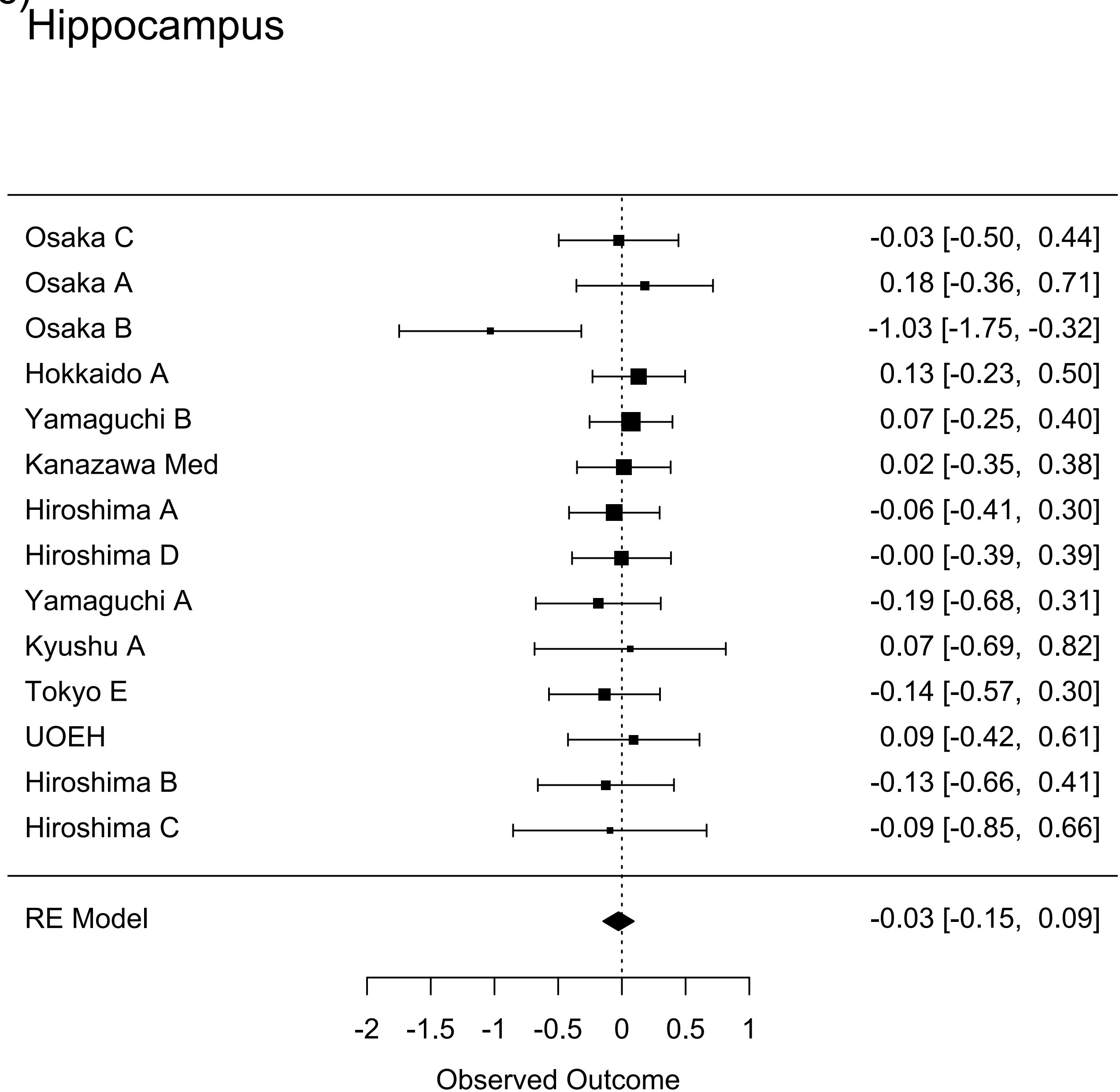
Hippocampus
Osaka C
-0.03 [-0.50, 0.44]
Osaka A
0.18 [-0.36, 0.71]
Osaka B
-1.03 [-1.75, -0.32]
Hokkaido A
0.13 [-0.23, 0.50]
Yamaguchi B
0.07 [-0.25, 0.40]
Kanazawa Med
0.02 [-0.35, 0.38]
Hiroshima A
-0.06 [-0.41, 0.30]
Hiroshima D
-0.00 [-0.39, 0.39]
Yamaguchi A
-0.19 [-0.68, 0.31]
Kyushu A
0.07 [-0.69, 0.82]
Tokyo E
-0.14 [-0.57, 0.30]
UOEH
0.09 [-0.42, 0.61]
Hiroshima B
-0.13 [-0.66, 0.41]
Hiroshima C
-0.09 [-0.85, 0.66]
RE Model
-0.03 [-0.15, 0.09]
-2
-1.5
-1
-0.5
0
0.5
1
Observed Outcome

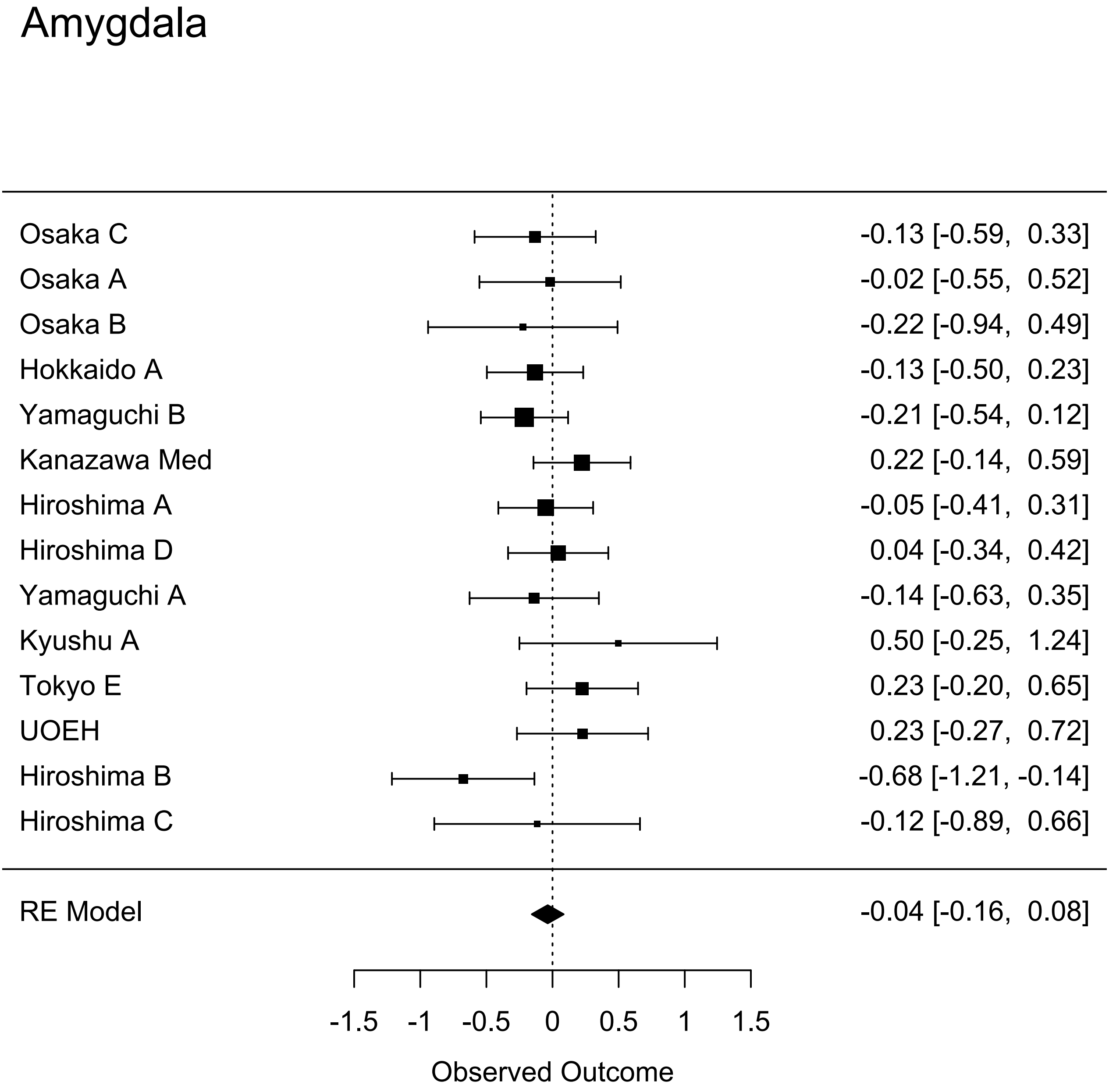

Amygdala
Osaka C -0.13 [-0.59, 0.33]
Osaka A -0.02 [-0.55, 0.52]
Osaka B -0.22 [-0.94, 0.49]
Hokkaido A -0.13 [-0.50, 0.23]
Yamaguchi B -0.21 [-0.54, 0.12]
Kanazawa Med 0.22 [-0.14, 0.59]
Hiroshima A -0.05 [-0.41, 0.31]
Hiroshima D 0.04 [-0.34, 0.42]
Yamaguchi A -0.14 [-0.63, 0.35]
Kyushu A 0.50 [-0.25, 1.24]
Tokyo E 0.23 [-0.20, 0.65]
UOEH 0.23 [-0.27, 0.72]
Hiroshima B -0.68 [-1.21, -0.14]
Hiroshima C -0.12 [-0.89, 0.66]
RE Model -0.04 [-0.16, 0.08]
-1.5
-1
-0.5
0
0.5
1
1.5
Observed Outcome

Thalamus

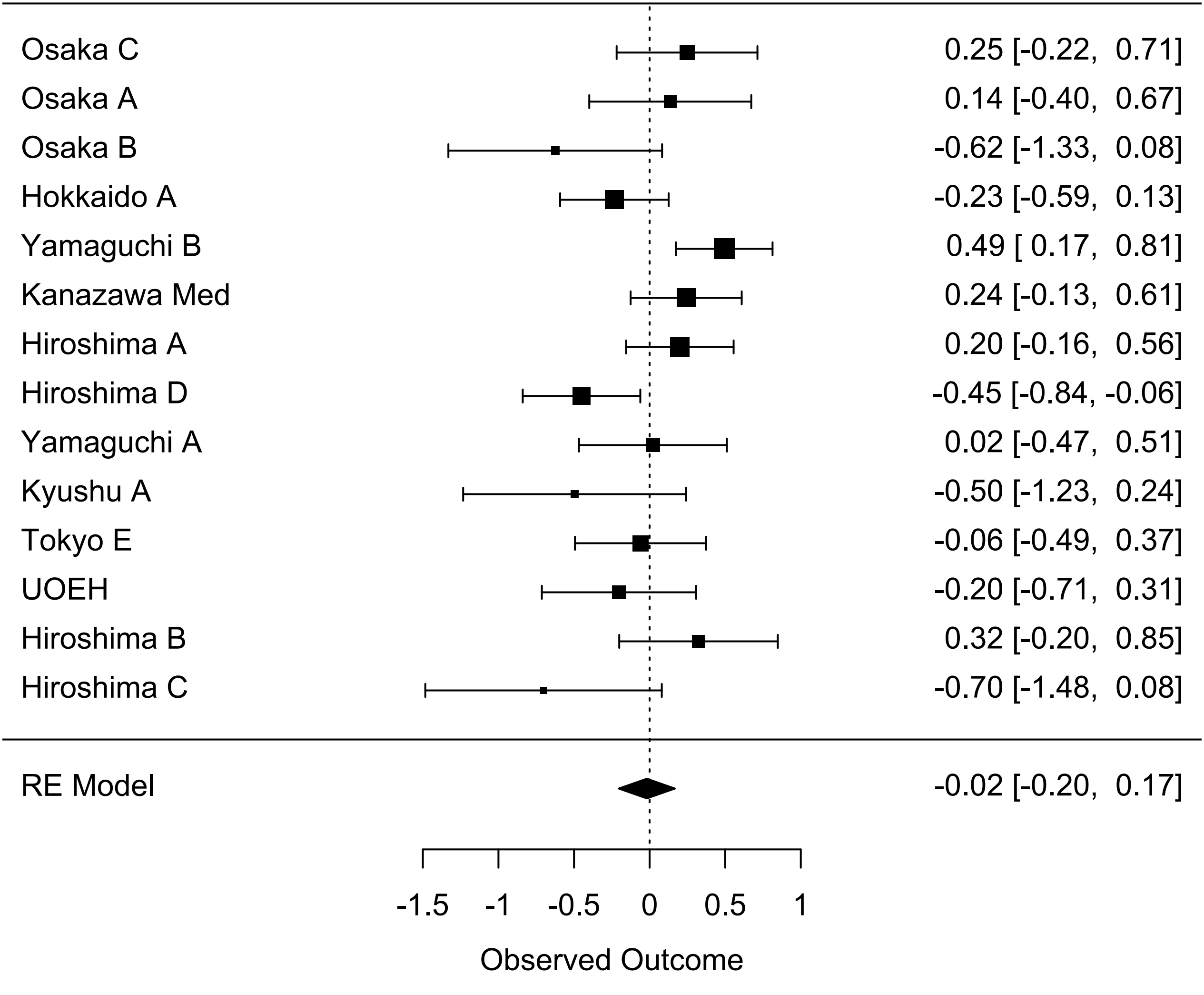

Osaka C 0.25 [-0.22, 0.71]
Osaka A 0.14 [-0.40, 0.67]
Osaka B -0.62 [-1.33, 0.08]
Hokkaido A -0.23 [-0.59, 0.13]
Yamaguchi B 0.49 [0.17, 0.81]
Kanazawa Med 0.24 [-0.13, 0.61]
Hiroshima A 0.20 [-0.16, 0.56]
Hiroshima D -0.45 [-0.84, -0.06]
Yamaguchi A 0.02 [-0.47, 0.51]
Kyushu A -0.50 [-1.23, 0.24]
Tokyo E -0.06 [-0.49, 0.37]
UOEH -0.20 [-0.71, 0.31]
Hiroshima B 0.32 [-0.20, 0.85]
Hiroshima C -0.70 [-1.48, 0.08]
RE Model -0.02 [-0.20, 0.17]
-1.5
-1
-0.5
0
0.5
1
Observed Outcome

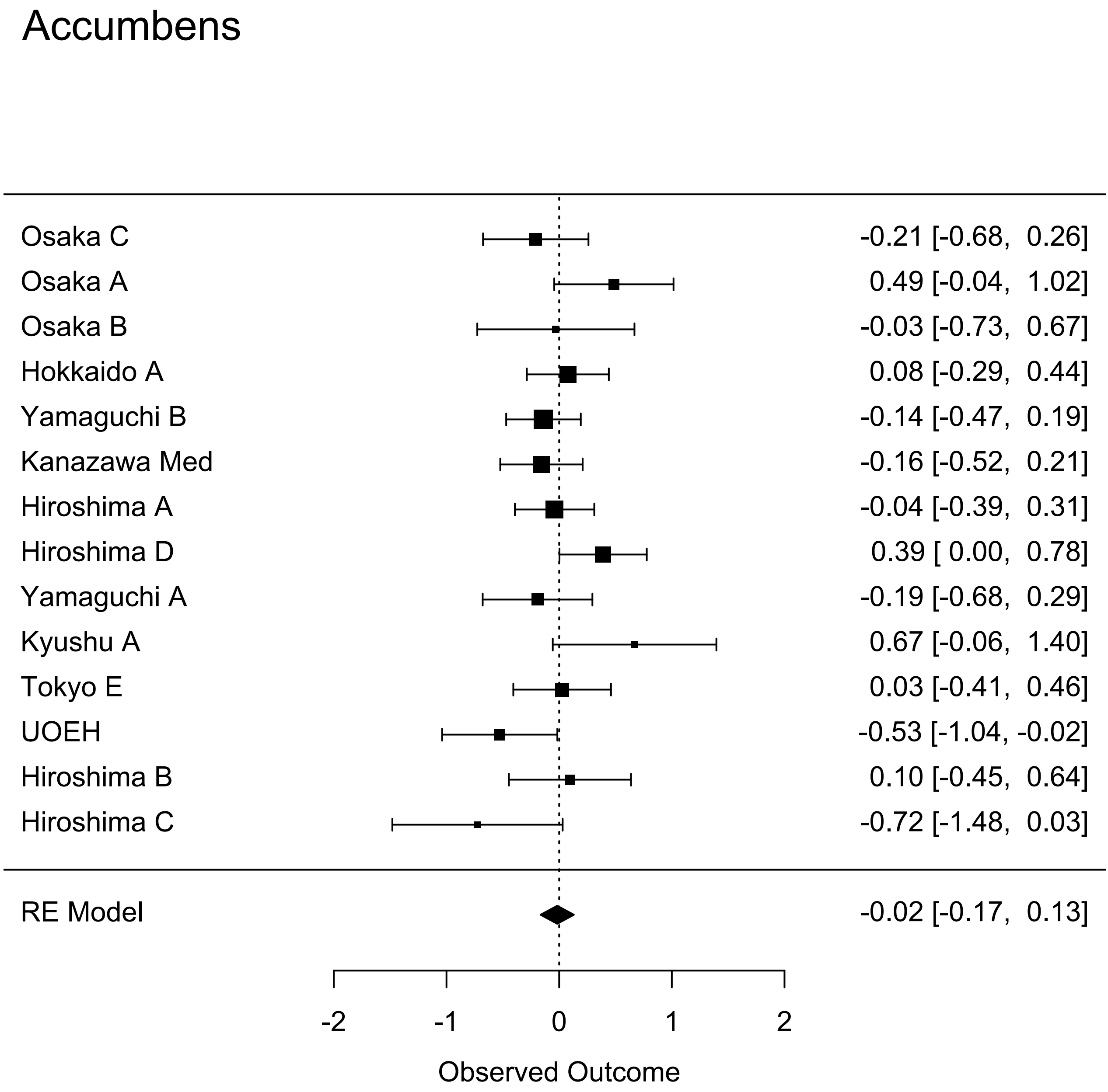

Accumbens
Osaka C
Osaka A
Osaka B
Hokkaido A
Yamaguchi B
Kanazawa Med
Hiroshima A
Hiroshima D
Yamaguchi A
Kyushu A
Tokyo E
UOEH
Hiroshima B
Hiroshima C
RE Model
-0.21 [-0.68, 0.26]
0.49 [-0.04, 1.02]
-0.03 [-0.73, 0.67]
0.08 [-0.29, 0.44]
-0.14 [-0.47, 0.19]
-0.16 [-0.52, 0.21]
-0.04 [-0.39, 0.31]
0.39 [0.00, 0.78]
-0.19 [-0.68, 0.29]
0.67 [-0.06, 1.40]
0.03 [-0.41, 0.46]
-0.53 [-1.04, -0.02]
0.10 [-0.45, 0.64]
-0.72 [-1.48, 0.03]
-0.02 [-0.17, 0.13]
-2
-1
0
1
2
Observed Outcome

Caudate

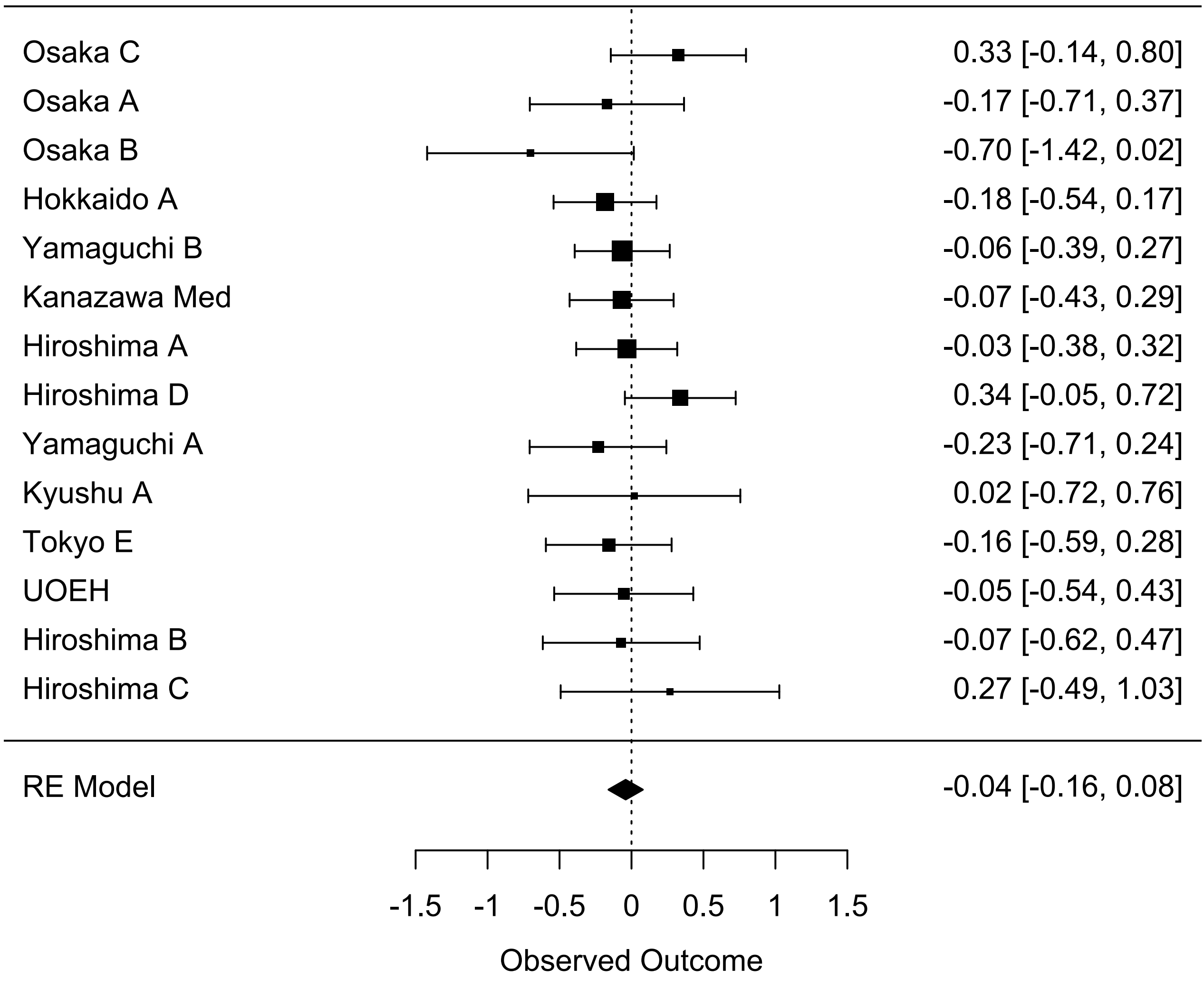

Osaka C 0.33 [-0.14, 0.80]
Osaka A -0.17 [-0.71, 0.37]
Osaka B -0.70 [-1.42, 0.02]
Hokkaido A -0.18 [-0.54, 0.17]
Yamaguchi B -0.06 [-0.39, 0.27]
Kanazawa Med -0.07 [-0.43, 0.29]
Hiroshima A -0.03 [-0.38, 0.32]
Hiroshima D 0.34 [-0.05, 0.72]
Yamaguchi A -0.23 [-0.71, 0.24]
Kyushu A 0.02 [-0.72, 0.76]
Tokyo E -0.16 [-0.59, 0.28]
UOEH -0.05 [-0.54, 0.43]
Hiroshima B -0.07 [-0.62, 0.47]
Hiroshima C 0.27 [-0.49, 1.03]
RE Model -0.04 [-0.16, 0.08]
-1.5
-1
-0.5
0
0.5
1
1.5
Observed Outcome

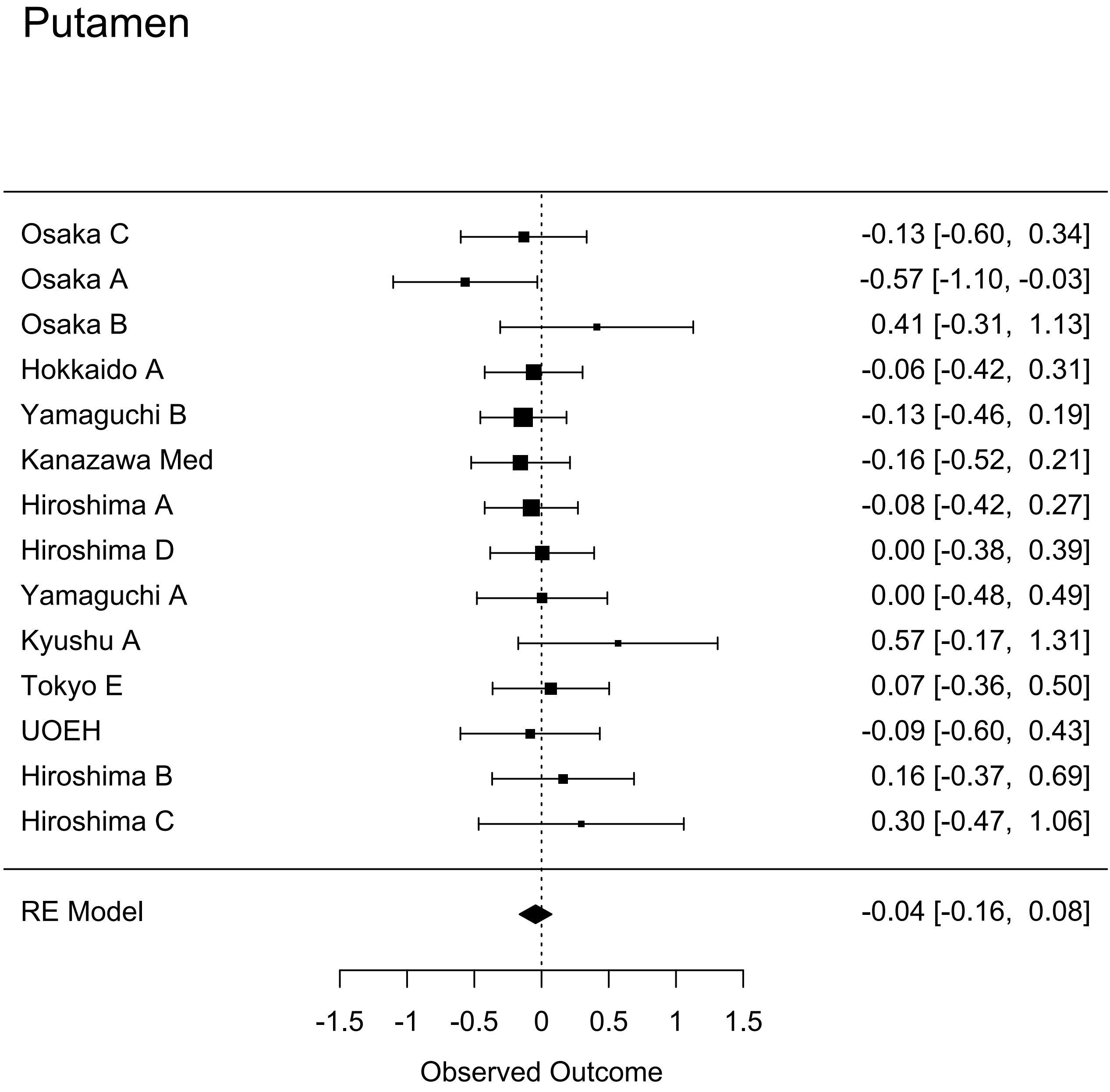
Putamen
Osaka C
Osaka A
Osaka B
Hokkaido A
Yamaguchi B
Kanazawa Med
Hiroshima A
Hiroshima D
Yamaguchi A
Kyushu A
Tokyo E
UOEH
Hiroshima B
Hiroshima C
RE Model
-0.13 [-0.60, 0.34]
-0.57 [-1.10, -0.03]
0.41 [-0.31, 1.13]
-0.06 [-0.42, 0.31]
-0.13 [-0.46, 0.19]
-0.16 [-0.52, 0.21]
-0.08 [-0.42, 0.27]
0.00 [-0.38, 0.39]
0.00 [-0.48, 0.49]
0.57 [-0.17, 1.31]
0.07 [-0.36, 0.50]
-0.09 [-0.60, 0.43]
0.16 [-0.37, 0.69]
0.30 [-0.47, 1.06]
-0.04 [-0.16, 0.08]
-1.5
-1
-0.5
0
0.5
1
1.5
Observed Outcome

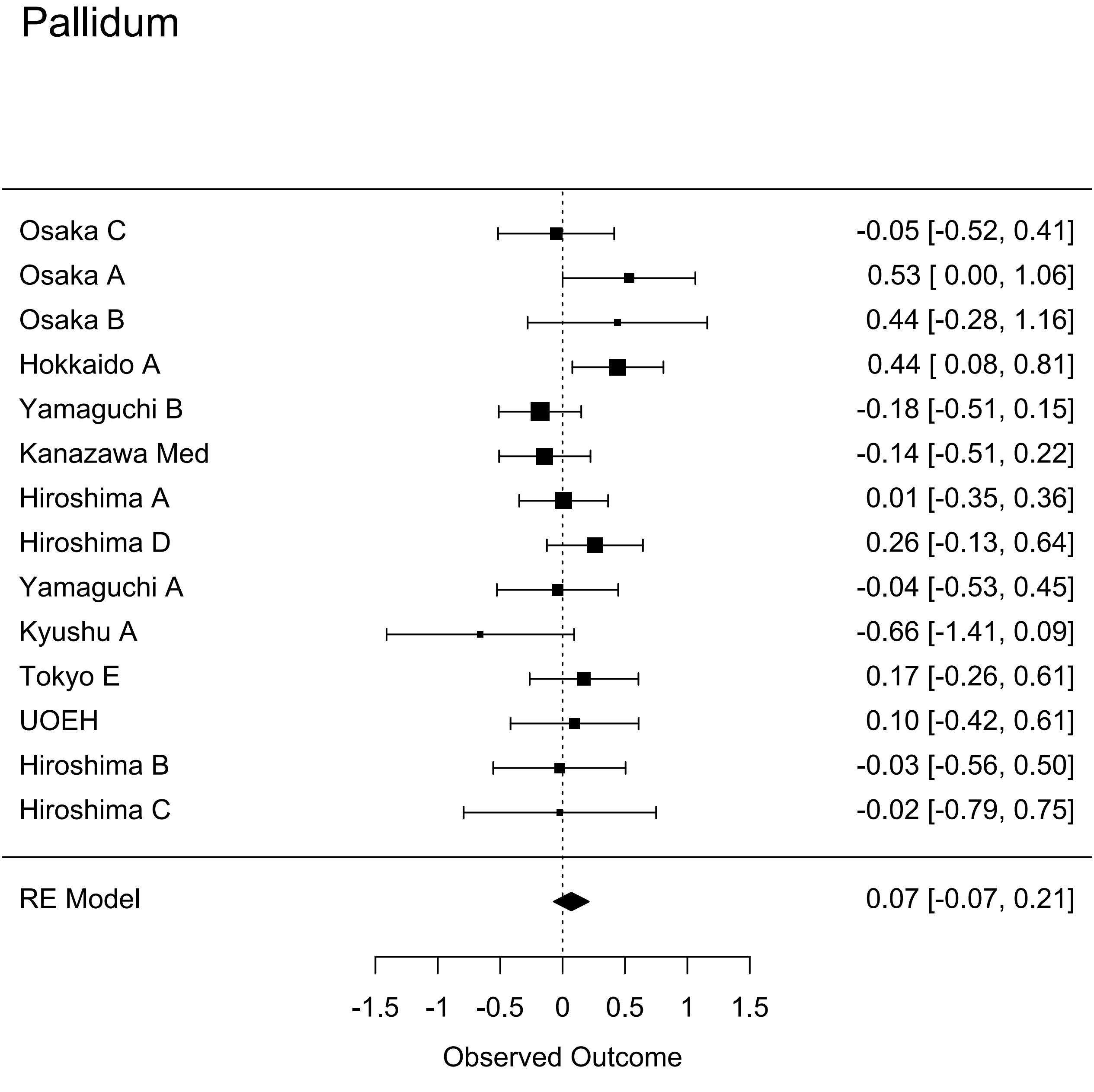
Pallidum
Osaka C
Osaka A
Osaka B
Hokkaido A
Yamaguchi B
Kanazawa Med
Hiroshima A
Hiroshima D
Yamaguchi A
Kyushu A
Tokyo E
UOEH
Hiroshima B
Hiroshima C
RE Model
-0.05 [-0.52, 0.41]
0.53 [0.00, 1.06]
0.44 [-0.28, 1.16]
0.44 [0.08, 0.81]
-0.18 [-0.51, 0.15]
-0.14 [-0.51, 0.22]
0.01 [-0.35, 0.36]
0.26 [-0.13, 0.64]
-0.04 [-0.53, 0.45]
-0.66 [-1.41, 0.09]
0.17 [-0.26, 0.61]
0.10 [-0.42, 0.61]
-0.03 [-0.56, 0.50]
-0.02 [-0.79, 0.75]
0.07 [-0.07, 0.21]
-1.5
-1
-0.5
0
0.5
1
1.5
Observed Outcome

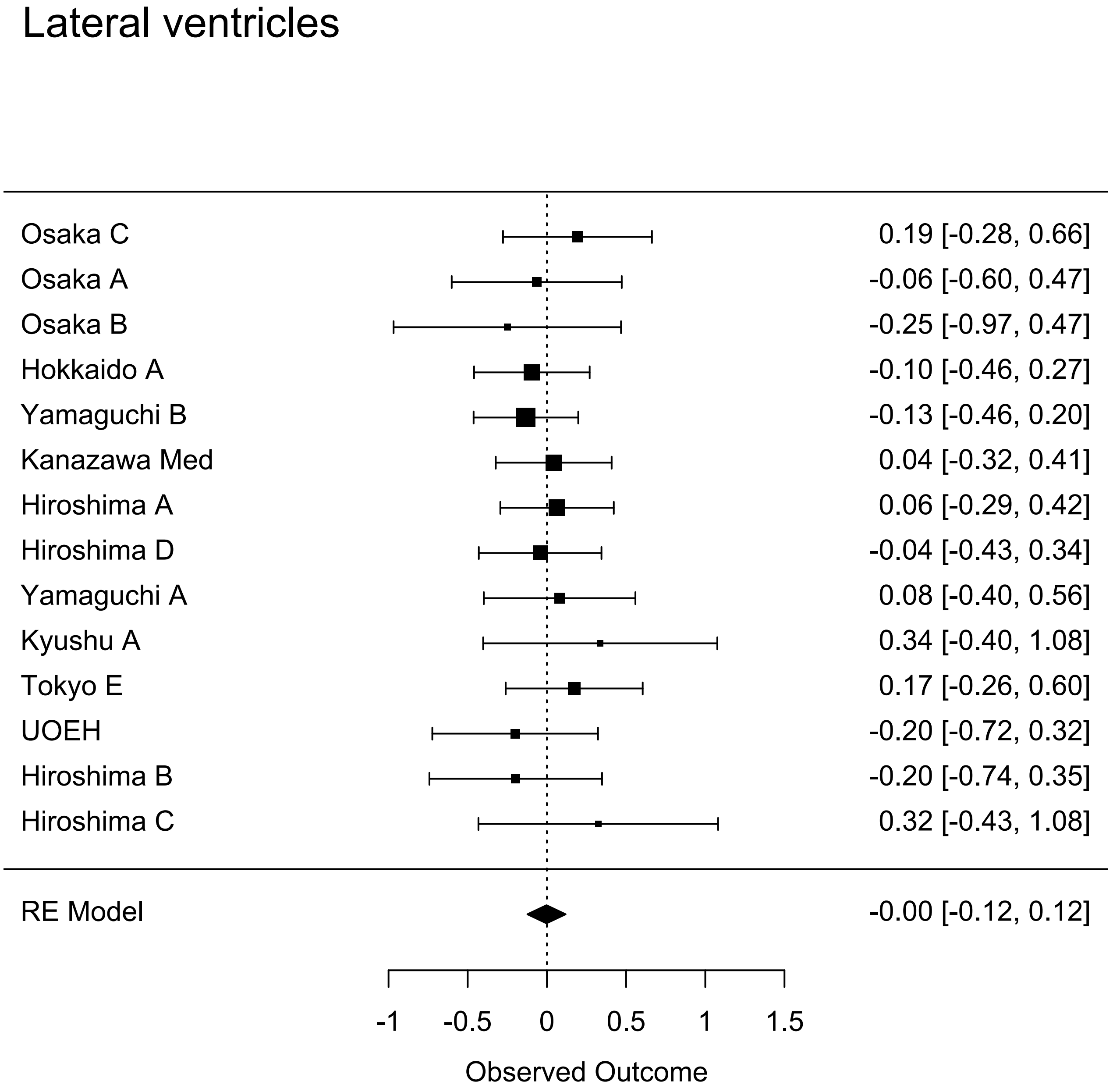
Lateral ventricles
Osaka C
0.19 [-0.28, 0.66]
Osaka A
-0.06 [-0.60, 0.47]
Osaka B
-0.25 [-0.97, 0.47]
Hokkaido A
-0.10 [-0.46, 0.27]
Yamaguchi B
-0.13 [-0.46, 0.20]
Kanazawa Med
0.04 [-0.32, 0.41]
Hiroshima A
0.06 [-0.29, 0.42]
Hiroshima D
-0.04 [-0.43, 0.34]
Yamaguchi A
0.08 [-0.40, 0.56]
Kyushu A
0.34 [-0.40, 1.08]
Tokyo E
0.17 [-0.26, 0.60]
UOEH
-0.20 [-0.72, 0.32]
Hiroshima B
-0.20 [-0.74, 0.35]
Hiroshima C
0.32 [-0.43, 1.08]
RE Model
-0.00 [-0.12, 0.12]
-1
-0.5
0
0.5
1
1.5
Observed Outcome

(d)

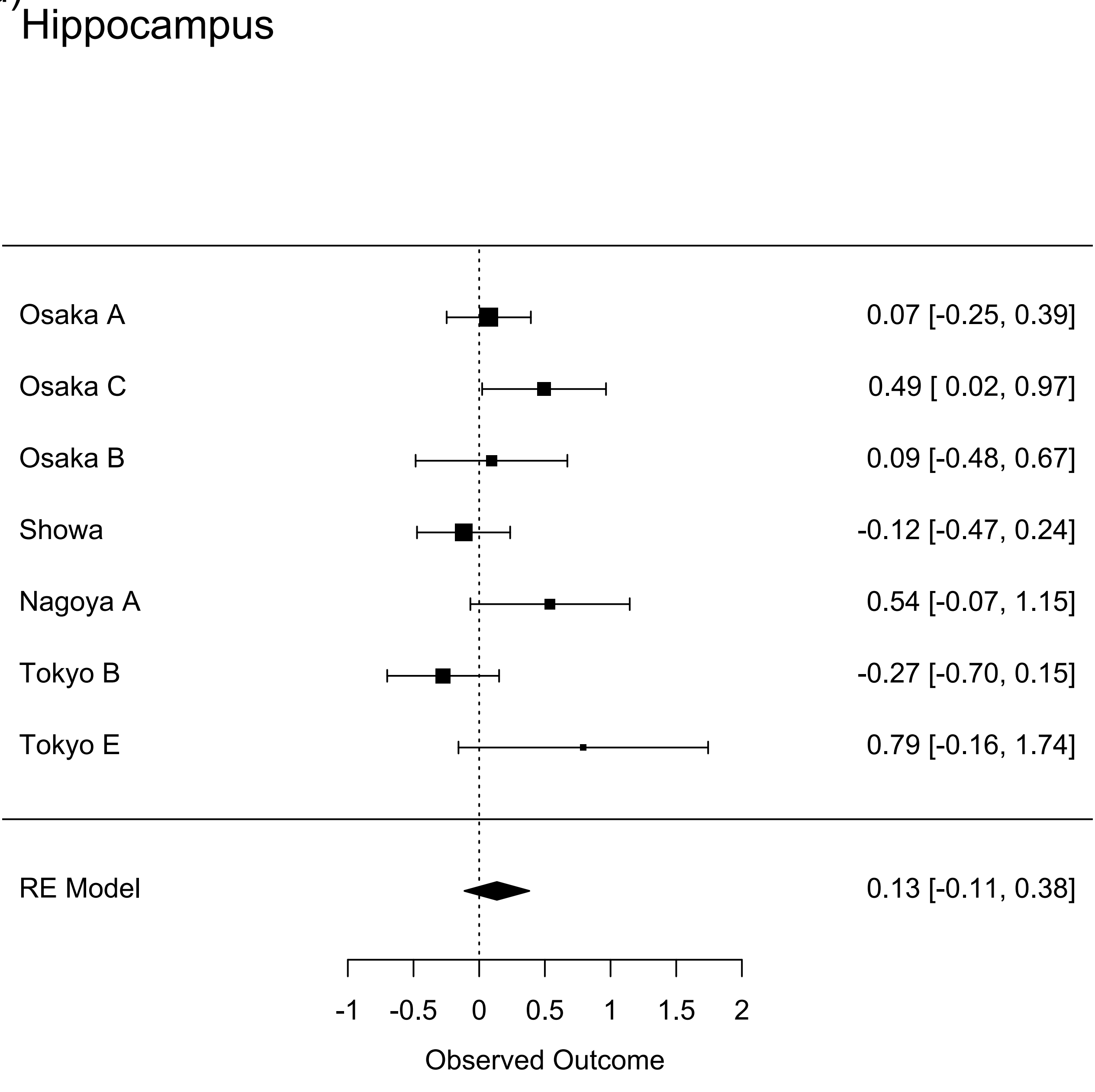
Hippocampus
Osaka A
0.07 [-0.25, 0.39]
Osaka C
0.49 [0.02, 0.97]
Osaka B
0.09 [-0.48, 0.67]
Showa
-0.12 [-0.47, 0.24]
Nagoya A
0.54 [-0.07, 1.15]
Tokyo B
-0.27 [-0.70, 0.15]
Tokyo E
0.79 [-0.16, 1.74]
RE Model
0.13 [-0.11, 0.38]
-1
-0.5
0
0.5
1
1.5
2
Observed Outcome

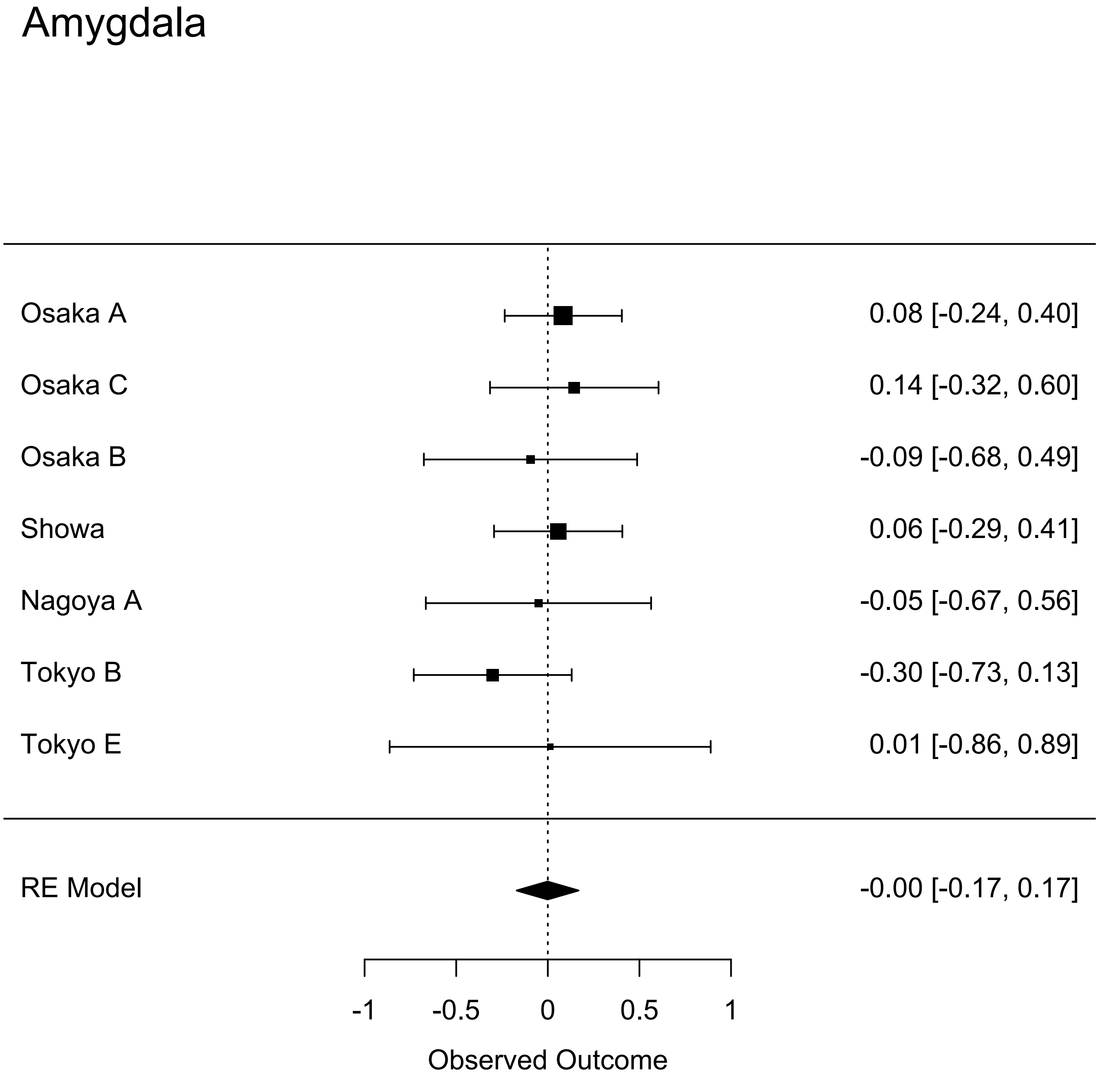
Amygdala
Osaka A
0.08 [-0.24, 0.40]
Osaka C
0.14 [-0.32, 0.60]
Osaka B
-0.09 [-0.68, 0.49]
Showa
0.06 [-0.29, 0.41]
Nagoya A
-0.05 [-0.67, 0.56]
Tokyo B
-0.30 [-0.73, 0.13]
Tokyo E
0.01 [-0.86, 0.89]
RE Model
-0.00 [-0.17, 0.17]
-1
-0.5
0
0.5
1
Observed Outcome

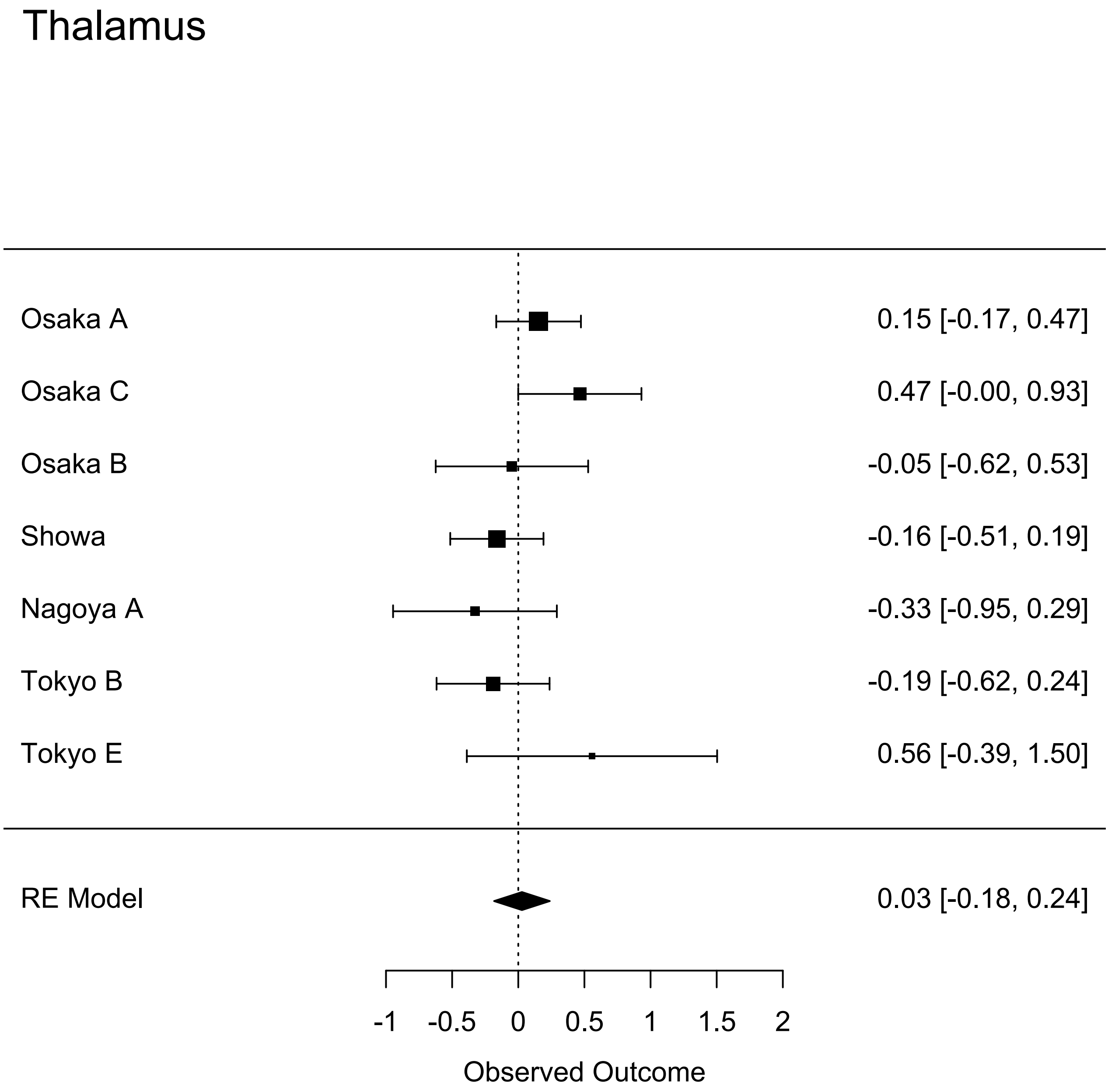
Thalamus
Osaka A
0.15 [-0.17, 0.47]
Osaka C
0.47 [-0.00, 0.93]
Osaka B
-0.05 [-0.62, 0.53]
Showa
-0.16 [-0.51, 0.19]
Nagoya A
-0.33 [-0.95, 0.29]
Tokyo B
-0.19 [-0.62, 0.24]
Tokyo E
0.56 [-0.39, 1.50]
RE Model
0.03 [-0.18, 0.24]
-1
-0.5
0
0.5
1
1.5
2
Observed Outcome

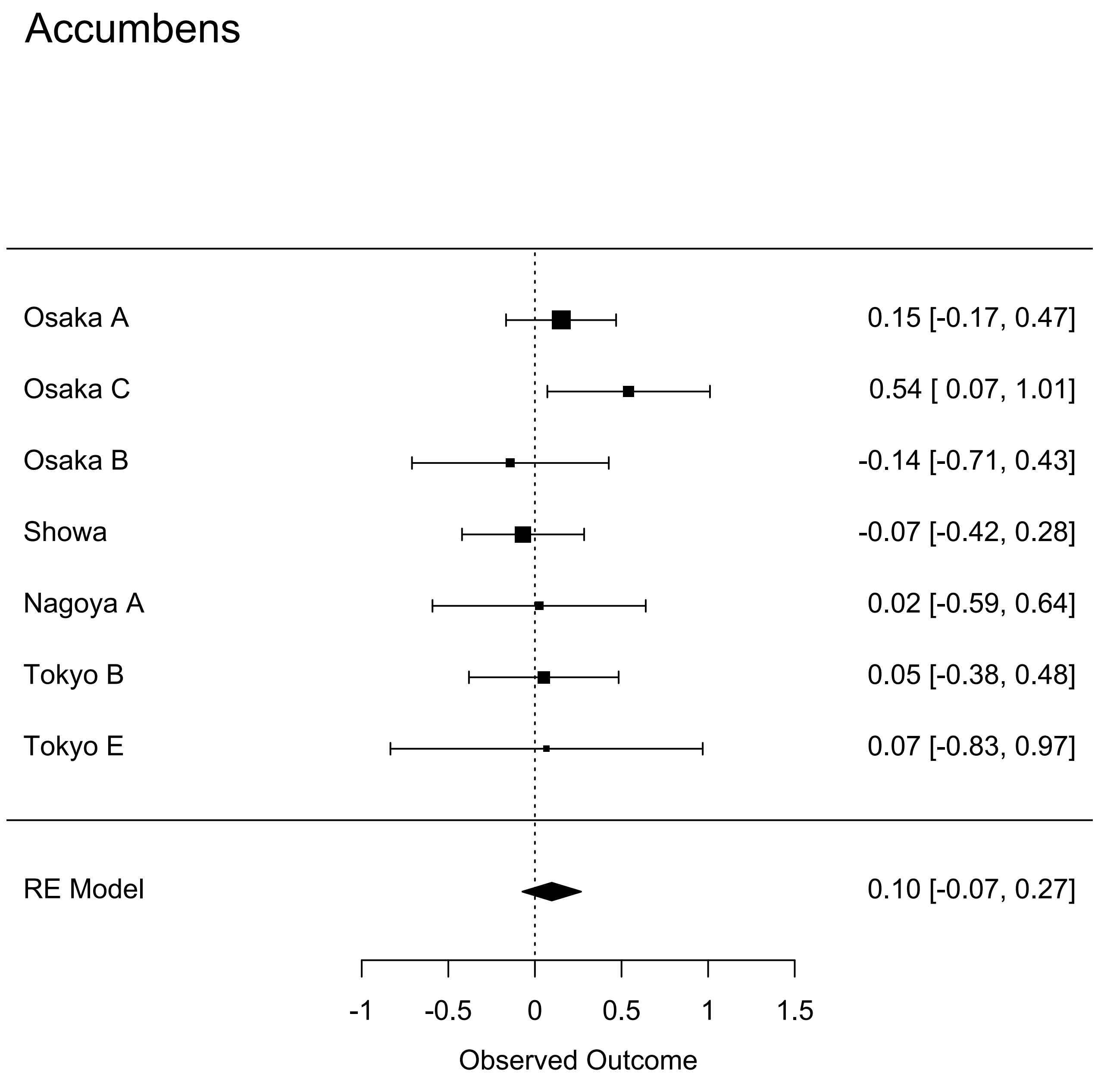
Accumbens
Osaka A
Osaka C
Osaka B
Showa
Nagoya A
Tokyo B
Tokyo E
RE Model
0.15 [-0.17, 0.47]
0.54 [0.07, 1.01]
-0.14 [-0.71, 0.43]
-0.07 [-0.42, 0.28]
0.02 [-0.59, 0.64]
0.05 [-0.38, 0.48]
0.07 [-0.83, 0.97]
0.10 [-0.07, 0.27]
-1
-0.5
0
0.5
1
1.5
Observed Outcome

Caudate

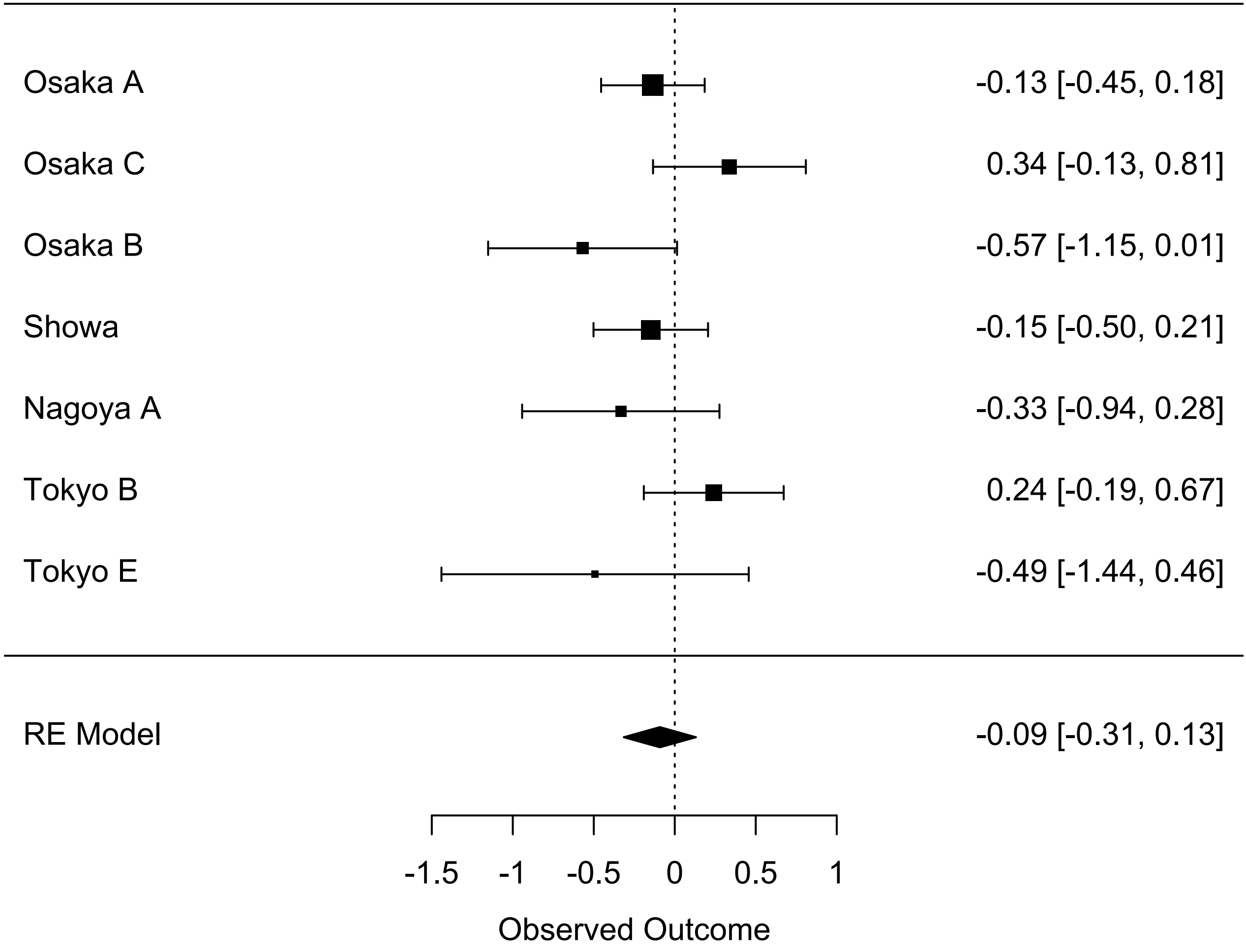
Osaka A
-0.13 [-0.45, 0.18]
Osaka C
0.34 [-0.13, 0.81]
Osaka B
-0.57 [-1.15, 0.01]
Showa
-0.15 [-0.50, 0.21]
Nagoya A
-0.33 [-0.94, 0.28]
Tokyo B
0.24 [-0.19, 0.67]
Tokyo E
-0.49 [-1.44, 0.46]
RE Model
-0.09 [-0.31, 0.13]
-1.5
-1
-0.5
0
0.5
1
Observed Outcome

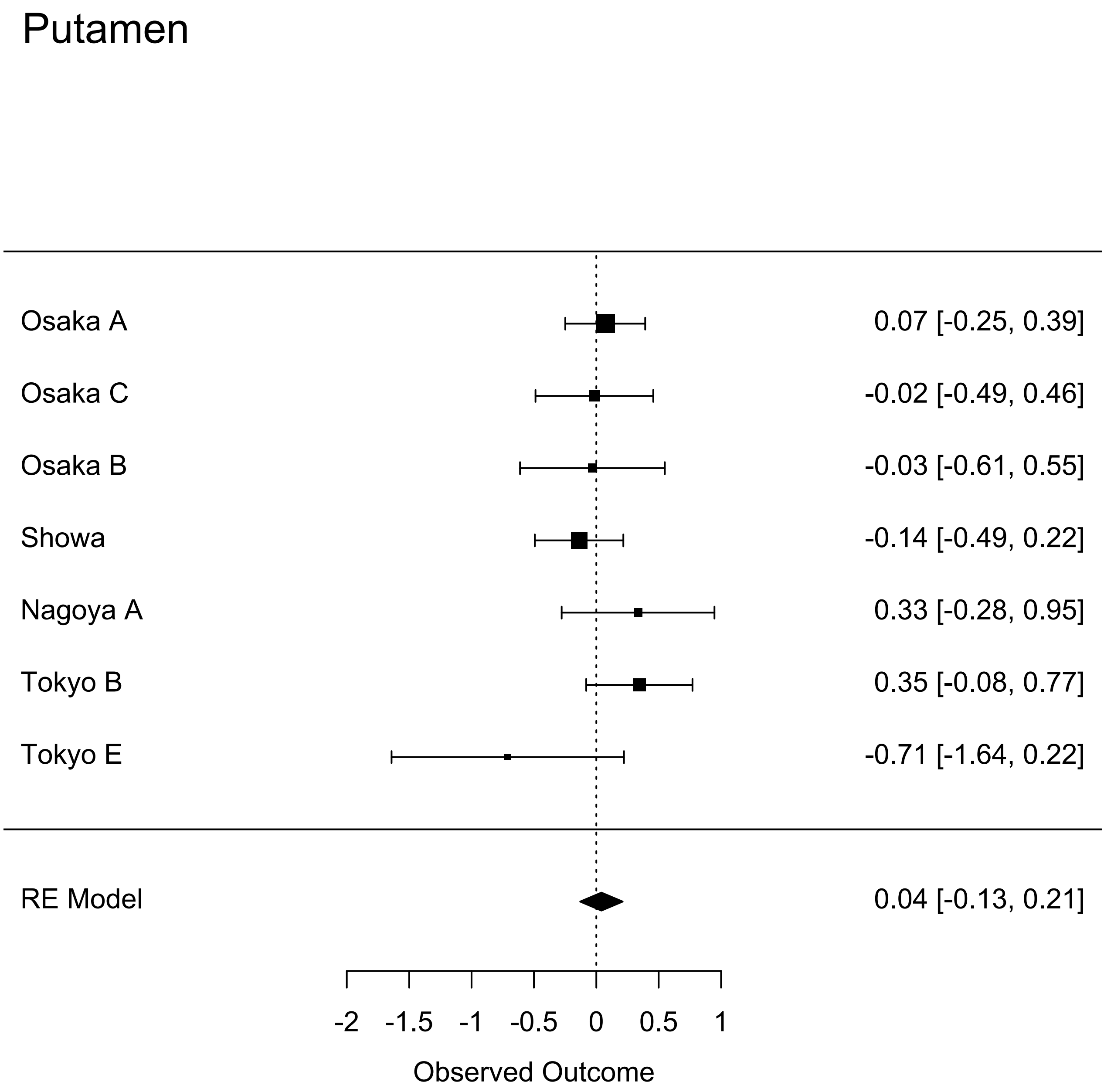
Putamen
Osaka A
0.07 [-0.25, 0.39]
Osaka C
-0.02 [-0.49, 0.46]
Osaka B
-0.03 [-0.61, 0.55]
Showa
-0.14 [-0.49, 0.22]
Nagoya A
0.33 [-0.28, 0.95]
Tokyo B
0.35 [-0.08, 0.77]
Tokyo E
-0.71 [-1.64, 0.22]
RE Model
0.04 [-0.13, 0.21]
-2
-1.5
-1
-0.5
0
0.5
1
Observed Outcome

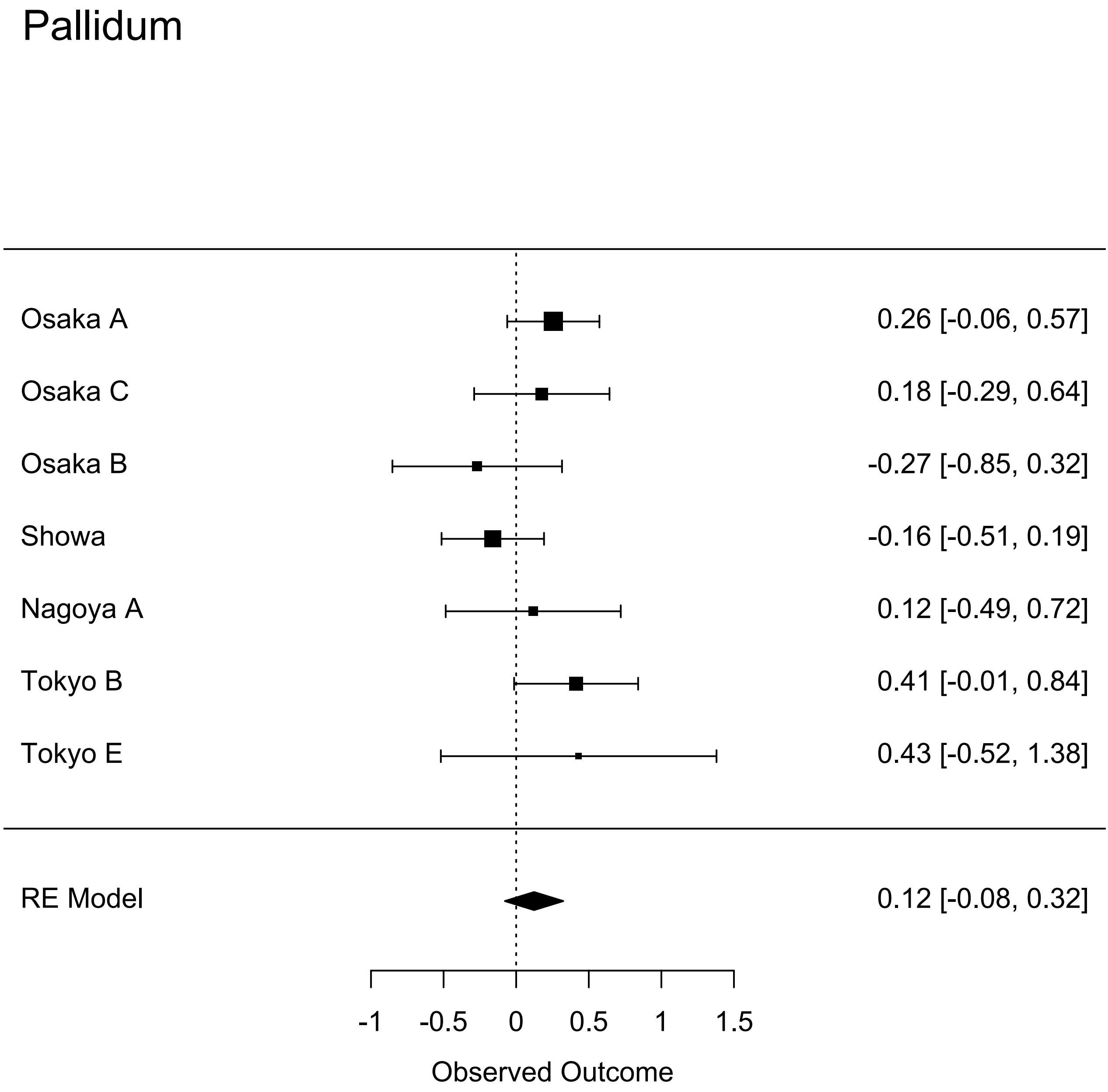
Pallidum
Osaka A
0.26 [-0.06, 0.57]
Osaka C
0.18 [-0.29, 0.64]
Osaka B
-0.27 [-0.85, 0.32]
Showa
-0.16 [-0.51, 0.19]
Nagoya A
0.12 [-0.49, 0.72]
Tokyo B
0.41 [-0.01, 0.84]
Tokyo E
0.43 [-0.52, 1.38]
RE Model
0.12 [-0.08, 0.32]
-1
-0.5
0
0.5
1
1.5
Observed Outcome

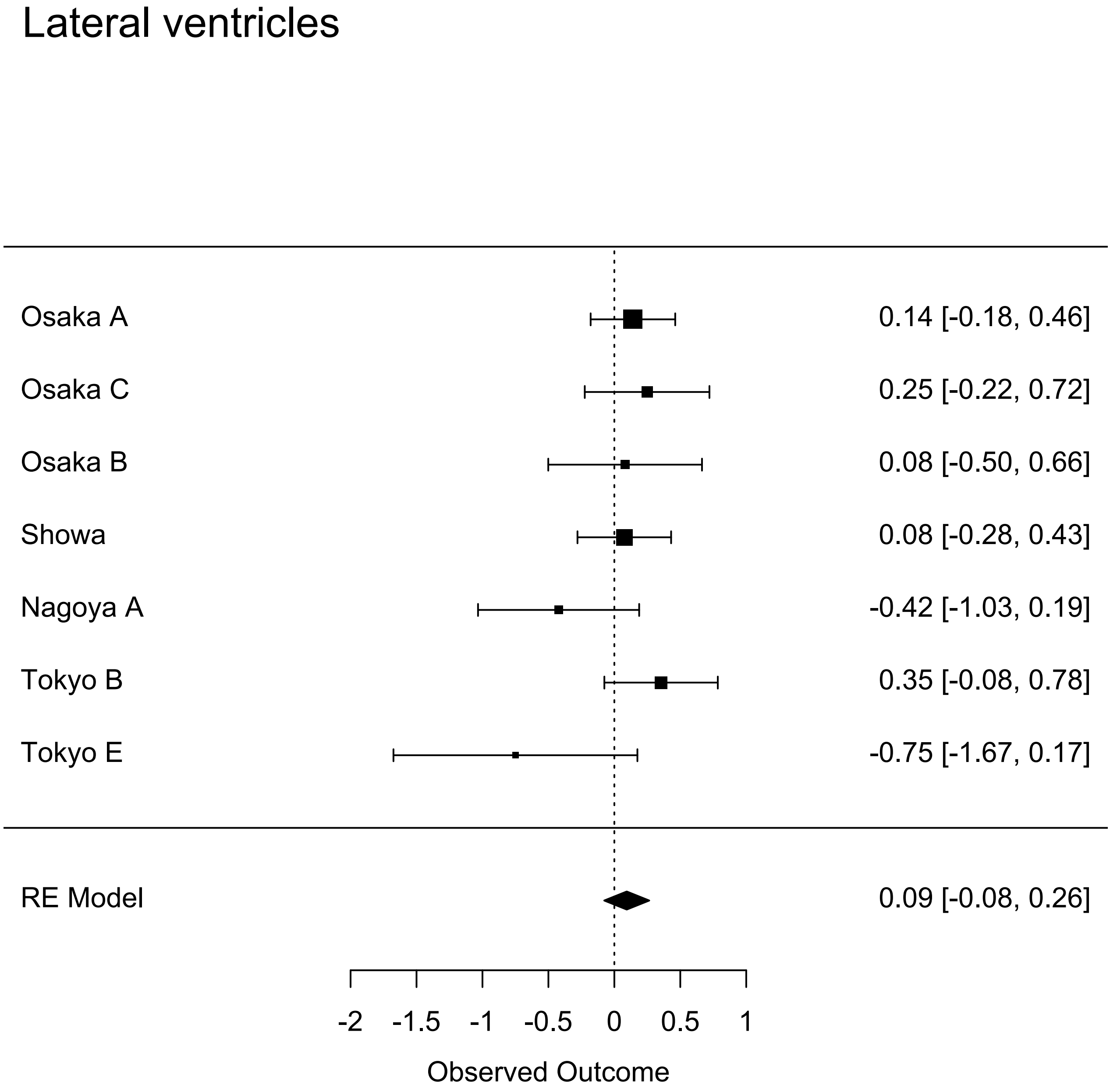
Lateral ventricles
Osaka A
0.14 [-0.18, 0.46]
Osaka C
0.25 [-0.22, 0.72]
Osaka B
0.08 [-0.50, 0.66]
Showa
0.08 [-0.28, 0.43]
Nagoya A
-0.42 [-1.03, 0.19]
Tokyo B
0.35 [-0.08, 0.78]
Tokyo E
-0.75 [-1.67, 0.17]
RE Model
0.09 [-0.08, 0.26]
-2
-1.5
-1
-0.5
0
0.5
1
Observed Outcome

Supplementary Fig. 3: The detailed association between MRI data-driven clustering results and cognitive/social function.

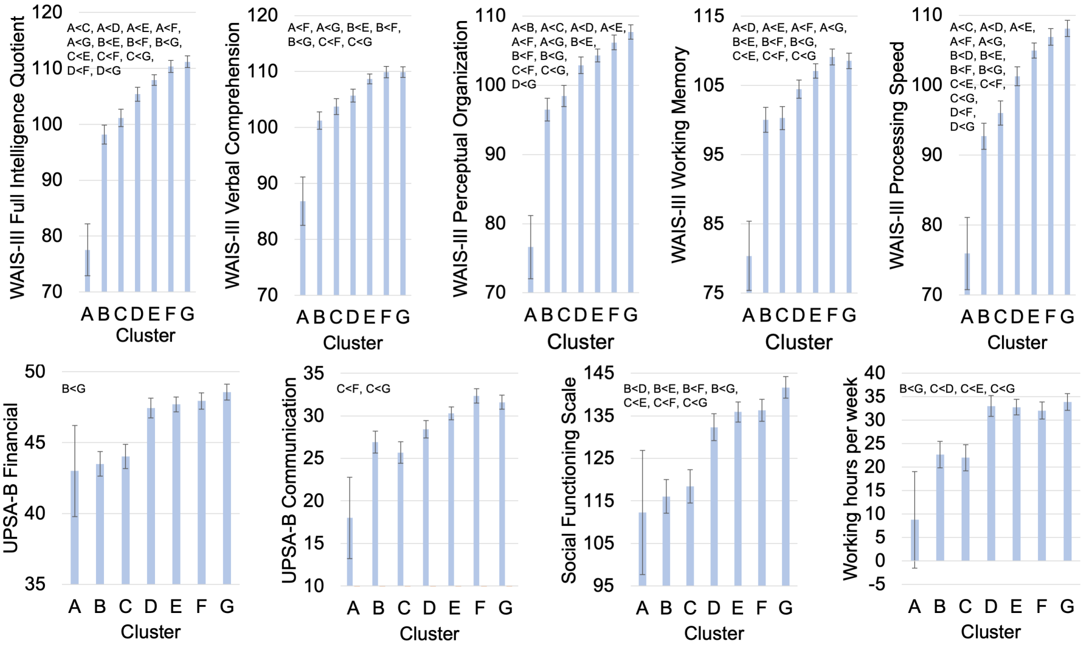

An analysis of variance (ANOVA) analysis revealed that, in subjects recruited at Osaka site, clustering results had significant effects on WAIS-III full intelligence quotient. In addition, a multiple analysis of variance (MANOVA) for WAIS-III subscales found their significant effects on Verbal Comprehension (VC), Perceptual Organization (PO), Working Memory (WM), and Processing Speed (PS) subscales. Further, a MANOVA analysis for social function found that their significant effects on UPSA-B Financial and Communication subscales, Social Functioning Scale and working hours per week. Vertical axes represent mean scores and standard errors. In addition, post hoc Games-Howell test results are shown here. Abbreviations: WAIS-III, Wechsler Adult Intelligence Scale 3rd edition; UPSA-B, the University of California San Diego Performance-Based Skills Assessment-Brief Version.

Supplementary Fig. 4: The distribution of medication daily doses in each brain biotype.

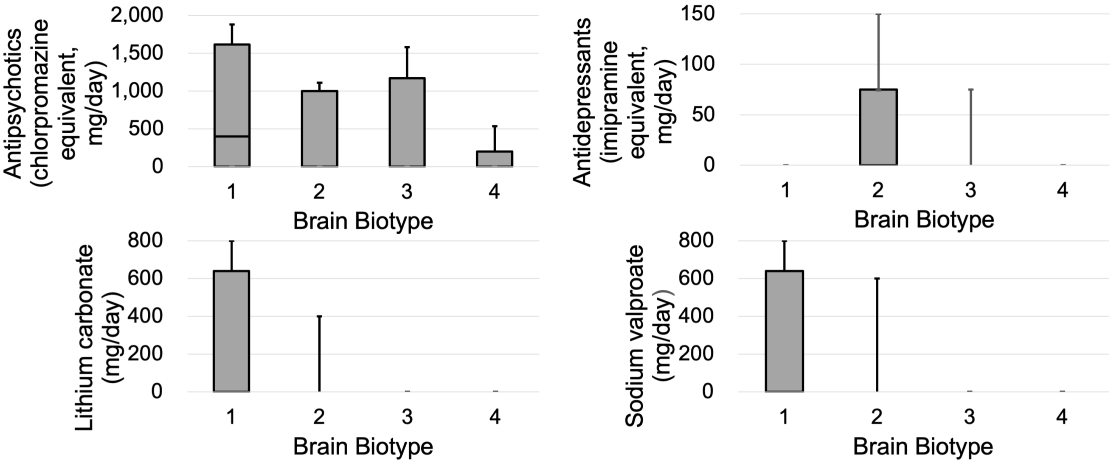

Results of post-hoc tests	Comparisons of BB1, BB2, and BB3 with BB4			Comparisons among BB1, BB2, and BB3
	BB1	BB2	BB3	
Antipsychotics	↑	↑	↑	BB1 > BB2 BB1 > BB3
Antidepressants		↑		BB2 > BB3
Lithium carbonate	↑	↑	↑	
Sodium valproate	↑	↑		BB1 > BB2 BB1 > BB3

Kruskal-Wallis tests revealed that, in subjects recruited at the Osaka site, there were significant differences among brain biotypes in daily doses of antipsychotics, antidepressants, lithium carbonate, and sodium valproate. Gray-colored box plots represent 10–90 percentile values. Error bars represent 5–95 percentile values. All 50 percentile values except of antipsychotics in Brain Biotype 1 (400 mg/day) were 0 mg/day. 95 percentile values in Brain Biotype 1 could not be calculated due to the small sample number (N = 17), and thus they are replaced with 100 percentile values in this figure. The results of post hoc pairwise comparison tests are shown in the lower part (abbreviation: BB, Brain Biotype).

Supplementary Tables

Supplementary Table 1: Participant demographics.

(a) Participant demographics of the HC vs SZ study population.

Protocol Name	HC	Sex		Age		SZ	Sex		Age	
	N	Male	Female	Mean	SD	N	Male	Female	Mean	SD
Osaka A	413	193	220	35.8	12.7	174	102	72	36.6	12.9
Osaka C	419	226	193	32.6	15.2	94	50	44	34.3	12.2
Nippon Med	194	41	153	47.9	9.0	213	121	92	44.1	13.6
Tokyo A	232	142	90	34.4	11.5	99	55	44	33.3	9.6
Osaka B	227	125	102	30.8	12.9	57	22	35	34.0	13.2
Toyama A	118	63	55	25.9	6.3	114	57	57	26.4	6.2
Kanazawa Med	114	72	42	35.3	11.5	109	41	68	40.3	12.7
Kyoto B	148	90	58	36.5	11.8	43	22	21	40.4	9.2
Kyoto A	111	63	48	31.9	10.6	77	41	36	36.4	9.1
Nagoya A	121	74	47	36.5	9.8	54	30	24	43.3	10.0
Hokkaido A	35	14	21	47.8	12.9	113	43	70	34.8	12.5
Tokyo B	80	54	26	28.6	5.6	41	27	14	31.4	9.2
Kyushu A	78	36	42	33.2	11.9	41	11	30	38.2	9.6
Toyama B	56	32	24	25.7	3.3	61	31	30	27.7	9.2
Yamaguchi A	90	18	72	49.3	16.1	27	5	22	55.4	8.2
Tokyo E	41	17	24	37.3	7.7	28	19	9	30.3	10.4
UOEH	54	36	18	36.6	12.0	15	6	9	28.0	13.4
Kyushu B	27	11	16	34.6	13.8	31	15	16	35.5	11.1
Tokyo D	47	17	30	38.8	9.0	11	6	5	38.4	4.2
Tokyo C	41	25	16	28.8	7.5	12	6	6	27.4	9.4
Hokkaido B	21	14	7	33.2	7.7	28	11	17	37.9	9.7
Tokushima B	19	10	9	41.6	10.7	21	11	10	42.9	10.3
Tokushima A	21	16	5	34.3	8.4	18	9	9	34.8	9.4
Nagoya B	13	8	5	66.8	4.3	19	9	10	40.4	11.5
Total	2720	1397	1323	35.3	13.3	1500	750	750	36.6	12.6

Abbreviations: HC, healthy control; SZ, schizophrenia; SD, standard deviation.

(b) Participant demographics of the HC vs BP study population.

Protocol Name	HC	Sex		Age		BP	Sex		Age	
	N	Male	Female	Mean	SD	N	Male	Female	Mean	SD
Osaka C	419	226	193	32.6	15.2	5	5	0	50.0	19.8
Kanazawa Med	114	72	42	35.3	11.5	34	18	16	45.6	15.0
Nagoya A	121	74	47	36.5	9.8	19	8	11	49.0	14.0
Yamaguchi B	113	46	67	44.4	19.0	15	9	6	40.9	13.6
Hokkaido A	35	14	21	47.8	12.9	78	41	37	45.5	16.3
Yamaguchi A	90	18	72	49.3	16.1	8	0	8	50.3	11.2
Kyushu A	78	36	42	33.2	11.9	18	7	11	48.4	14.7
Hiroshima D	53	28	25	56.0	14.2	26	10	16	52.7	15.7
Tokyo E	41	17	24	37.3	7.7	23	15	8	34.4	9.5
Kyushu B	27	11	16	34.6	13.8	9	5	4	48.8	7.9
Total	1091	542	549	37.8	15.8	235	118	117	45.8	15.2

Abbreviations: HC, healthy control; BP, bipolar disorder; SD, standard deviation.

(c) Participant demographics of the HC vs MDD study population.

Protocol Name	HC	Sex		Age		MDD	Sex		Age	
	N	Male	Female	Mean	SD	N	Male	Female	Mean	SD
Osaka C	419	226	193	32.6	15.2	19	9	10	48.6	13.0
Osaka A	413	193	220	35.8	12.7	14	9	5	27.6	10.2
Osaka B	227	125	102	30.8	12.9	9	4	5	58.3	19.4
Hokkaido A	35	14	21	47.8	12.9	175	85	90	47.2	17.3
Yamaguchi B	113	46	67	44.4	19.0	55	24	31	51.6	12.7
Kanazawa Med	114	72	42	35.3	11.5	43	27	16	43.2	13.8
Hiroshima A	64	29	35	34.5	12.9	71	36	35	42.5	11.5
Hiroshima D	53	28	25	56.0	14.2	61	23	38	49.2	14.2
Yamaguchi A	90	18	72	49.3	16.1	21	9	12	50.2	12.1

Kyushu A	78	36	42	33.2	11.9	9	5	4	49.0	11.6
Tokyo E	41	17	24	37.3	7.7	43	24	19	37.7	11.3
UOEH	54	36	18	36.6	12.0	23	10	13	43.3	13.7
Hiroshima B	19	8	11	42.4	9.4	47	25	22	44.5	10.5
Hiroshima C	41	11	30	42.7	11.6	8	1	7	37.5	9.4
Total	1761	859	902	36.6	15.0	598	291	307	45.7	14.8

Abbreviations: HC, healthy control; MDD, major depressive disorder; SD, standard deviation.

(d) Participant demographics of the HC vs ASD study population.

Protocol Name	HC	Sex		Age		ASD	Sex		Age	
	N	Male	Female	Mean	SD	N	Male	Female	Mean	SD
Osaka A	413	193	220	35.8	12.7	45	29	16	24.4	10.2
Osaka C	419	226	193	32.6	15.2	18	10	8	27.8	9.8
Osaka B	227	125	102	30.8	12.9	12	8	4	25.8	10.0
Showa	68	55	13	26.9	5.9	66	57	9	30.1	6.5
Nagoya A	121	74	47	36.5	9.8	12	12	0	30.7	9.5
Tokyo B	80	54	26	28.6	5.6	34	34	0	30.2	6.8
Tokyo E	41	17	24	37.3	7.7	6	6	0	36.8	8.4
Total	1369	744	625	33.2	12.9	193	156	37	28.5	8.7

Abbreviations: HC, healthy control; ASD, autism spectrum disorder; SD, standard deviation.

Supplementary Table 2: Imaging parameters for each protocol employed in the current mega-analytic study.

Protocol	Scanner type	Imaging parameters	Slice orientation
Osaka A	GE 1.5T Signa EXCITE	T1-weighted images, using a fast SPGR sequence and a head QD coil, were acquired with the following parameters; TR = 12.6 ms, TE = 4.2 ms, TI = 400 ms, flip angle = 15 degrees, matrix = 256 × 256 × 124, FOV = 240 × 240 × 172 mm, voxel size = 0.9375 × 0.9375 × 1 mm, slice thickness = 1.4 mm, number of slices = 124.	sagittal
Osaka C	GE 3.0T DISCOVERY 750	T1-weighted images, using a fast SPGR sequence and a HNS Head coil, were acquired with the following parameters; TR = 8.152 ms, TE = 3.172 ms, TI = 400 ms, flip angle = 11 degrees, matrix = 256 x 256, FOV = 260 × 260 × 187.2 mm, voxel size = 1.0156 × 1.0156 × 1.2 mm, slice thickness = 1.2 mm, number of slices = 156.	sagittal
Nippon Med	GE 1.5T Signa HDxp	T1-weighted images, using a SPGR sequence and phased-array coil, were acquired with the following parameters; TR = 7.4 ms, TE = 3.4 ms, TI = 20 ms, flip angle = 20 degrees, matrix = 256 × 256 × 50, FOV = 256 × 256 × 200 mm, voxel size = 1 × 1 × 1 mm, slice thickness = 1.5 mm, number of slices = 50.	axial
Hokkaido A	Siemens 1.5T Symphony	T1-weighted images, using a MPRAGE sequence and a quadrature head coil, were acquired with the following parameters; TR = 1900 ms, TE = 3.93 ms, TI = 1100 ms, flip angle = 15 degrees, matrix = 256 × 256 × 240, FOV = 250 × 240 × 250 mm, voxel size = 0.977 × 1 × 0.977 mm, slice thickness = 1 mm, number of slices = 240.	coronal
Tokyo A	GE 1.5T Signa Horizon	T1-weighted images, using a SPGR sequence and a circularly polarized head coil, were acquired with the following parameters; TR = 35 ms, TE = 7 ms, flip angle = 30 degrees, matrix = 256 × 256 × 124, FOV = 186 × 240 × 240 mm, voxel size = 0.9375 × 0.9375 × 1.5 mm, slice thickness = 1.5 mm, number of slices = 124.	axial
Osaka B	GE 3.0T Signa HDxt	T1-weighted images, using a fast SPGR sequence and 8HRBRAIN coil, were acquired with the following parameters; TR = 7.2 ms, TE = 2.9 ms, TI = 400 ms, flip angle = 11 degrees, matrix = 256 × 256 × 172, FOV = 240 × 240 × 172 mm, voxel size = 0.9375 × 0.9375 × 1 mm, slice thickness = 1 mm, number of slices = 172.	sagittal
Kanazawa med	Siemens 3.0T	T1-weighted images, using a MPRAGE sequence and a 32-channnel head coil, were acquired with the following parameters; TR = 1420 ms, TE = 2.06 ms, TI = 800 ms, flip angle = 9 degrees, matrix = 256 ×	sagittal

	Trio, A Tim System	256 × 192, FOV = 230 × 230 × 172 mm, voxel size = 0.9 × 0.9 × 0.9 mm, slice thickness = 0.9 mm, number of slices = 192.	
Toyama A	Siemens 1.5T Vision	T1-weighted images, using a 3D gradient echo sequence (FLASH) and a CP head coil, were acquired with the following parameters; TR = 24 ms, TE = 10 ms, flip angle = 40 degrees, matrix = 256 × 256, FOV = 256 × 256 × 179 mm, voxel size = 1 × 1 × 1 mm, slice thickness = 1 mm, number of slices = 160 - 180.	sagittal
Nagoya A	Siemens 3.0T Verio	T1-weighted images, using a MPRAGE sequence and a 32-channel head coil, were acquired with the following parameters; TR = 2500 ms, TE = 2.48 ms, TI = 900 ms, flip angle = 8 degrees, matrix = 256, FOV = 256 × 256 × 192 mm, voxel size = 1 × 1 × 1 mm, slice thickness = 1 mm, number of slices = 192.	sagittal
Kyoto B	Siemens 3.0T Trio-Tim	T1-weighted images, using a MPRAGE sequence and a 32-channel head coil, were acquired with the following parameters; TR = 2000 ms, TE = 3.4 ms, TI = 990 ms, flip angle = 8 degrees, matrix = 350 × 263 × 350, FOV = 208 × 240 × 225 mm, voxel size = 1 × 0.9375 × 0.9375 mm, slice thickness = 1 mm, number of slices = 208.	axial
Kyoto A	Siemens 3.0T Trio	T1-weighted images, using a MPRAGE sequence and a 8-channnel head coil, were acquired with the following parameters; TR = 2000 ms, TE = 4.38 ms, TI = 990 ms, flip angle = 8 degrees, matrix = 350 × 263 × 350, FOV = 208 × 240 × 225 mm, voxel size = 1 × 0.9375 × 0.9375 mm, slice thickness = 1 mm, number of slices = 208.	axial
Yamaguchi B	Siemens 3.0T Skyra	T1-weighted images, using a MPRAGE sequence and a 20-channel head neck coil, were acquired with the following parameters; TR = 2300 ms, TE = 2.95 ms, TI = 900 ms, flip angle = 9 degrees, matrix = 256 × 256, FOV = 270 × 253.1 × - mm, voxel size = 1.1 × 1.1 × 1.2 mm, slice thickness = 1.2 mm, number of slices = 176.	sagittal
Tokyo B	GE 3.0T Signa	T1-weighted images, using a fast SPGR sequence and a 8-channel coil, were acquired with the following parameters; TR = 6.8 ms, TE = 1.94 ms, flip angle = 20 degrees, matrix = 256 × 256 × 176, FOV = 176 × 256 × 256 mm, voxel size = 1 × 1 × 1 mm, slice thickness = 1 mm, number of slices = 176.	axial
Kyushu A	Philips 3.0T Achieva TX	T1-weighted images, using a 3D T1-TFE sequence and a 8ch-SENSE-Head coil, were acquired with the following parameters; TR = 8.6 ms, TE = 4 ms, TI = 1072.5 ms, flip angle = 8 degrees, matrix = 240 × 240 × 190, FOV = 240 × 240 × 190 mm, voxel size = 1 × 1 × 1 mm, slice thickness = 1 mm, number of slices = 190.	sagittal
Yamaguchi A	Siemens 1.5T	T1-weighted images, using a 3D gradient echo sequence (FLASH) and a CP head coil, were acquired with the following parameters; TR = 24 ms, TE = 5 ms, flip angle = 40 degrees, matrix = 256 × 256, FOV	sagittal

	Vision	= 256 × 256 × 256 mm, voxel size = 1 × 1 × 1 mm, slice thickness = 1 mm, number of slices = 170.	
Tokyo E	GE 3.0T Discovery MR750W	T1-weighted images, using a SPGR sequence and a 24-channel head coil, were acquired with the following parameters; TR = 7.7 ms, TE = 3.1 ms, TI = 400 ms, flip angle = 11 degrees, matrix = 256 × 256, FOV = 260 × 260 × 240 mm, voxel size = 1 × 1 × 1.2 mm, slice thickness = 1.2 mm, number of slices = 200.	sagittal
Hiroshima D	Siemens 3.0T Verio.Dot	T1-weighted images, using a MPRAGE sequence and a 12-channel head coil, were acquired with the following parameters; TR = 2300 ms, TE = 2.98 ms, TI = 900 ms, flip angle = 9 degrees, matrix = 256 × 256, FOV = 256 × 256 × 192 mm, voxel size = 1 × 1 × 1 mm, slice thickness = 1 mm, number of slices = 192.	sagittal
Hiroshima A	GE 3.0T Signa HDxt	T1-weighted images, using a SPGR sequence and an 8HR brain coil, were acquired with the following parameters; TR = 6812 ms, TE = 1896 ms, TI = 450 ms, flip angle = 20 degrees, matrix = 256 × 256, FOV = 256 × 256 × 184 mm, voxel size = 1 × 1 × 1 mm, slice thickness = 1 mm, number of slices = 184.	sagittal
Showa	Siemens 3.0T Verio	T1-weighted images, using a MPRAGE sequence and a 12-channel head coil, were acquired with the following parameters; TR = 2300 ms, TE = 2.98 ms, TI = 900 ms, flip angle = 9 degrees, matrix = 256 × 256, FOV = 256 × 256 × 240 mm, voxel size = 1 × 1 × 1 mm, slice thickness = 1 mm, number of slices = 240.	sagittal
Toyama B	Siemens 3.0T Verio	T1-weighted images, using a MPRAGE sequence, were acquired with the following parameters; TR = 2300 ms, TE = 2.9 ms, TI = 900 ms, flip angle = 9 degrees, matrix = 256 × 256, FOV = 256 × 240 × 212 mm, voxel size = 1 × 1 × 1.2 mm, slice thickness = 1.2 mm, number of slices = 176.	sagittal
UOEH	GE 3.0T Signa HDxt	T1-weighted images, using a fast SPGR sequence and 8HRBRAIN coil, were acquired with the following parameters; TR = 9.9 ms, TE = 4.0 ms, TI = 700 ms, flip angle = 10 degrees, matrix = 256 × 256 × 128, FOV = 240 × 240 × 128 mm, voxel size = 0.9375 × 0.9375 × 1.2 mm, slice thickness = 1.2 mm, number of slices = 128.	sagittal
Kyushu B	Philips 3.0T Achieva TX	T1-weighted images, using a 3D T1-TFE sequence and a 8ch-SENSE-Head coil, were acquired with the following parameters; TR = 8.6 ms, TE = 4 ms, TI = 1072.5 ms, flip angle = 8 degrees, matrix = 240 × 240 × 190, FOV = 240 × 240 × 190 mm, voxel size = 1 × 1 × 1 mm, slice thickness = 1 mm, number of slices = 190.	sagittal
Hiroshima B	Siemens 3.0T	T1-weighted images, using a MPRAGE sequence and a 12-channel head coil, were acquired with the following parameters; TR = 1900 ms, TE = 2.38 ms, TI = 900 ms, flip angle = 10 degrees, matrix = 256 ×	sagittal

	Spectra	256, FOV = 256 × 256 × 192 mm, voxel size = 0.8 × 0.8 × 0.8 mm, slice thickness = 0.8 mm, number of slices = 192.	
Tokyo D	Philips 3.0T Achieva	T1-weighted images, using a MPRAGE sequence and a SENSE-Head-8 coil, were acquired with the following parameters; TR = 6.8 ms, TE = 3.1 ms, TI = 845.9 ms, flip angle = 9 degrees, matrix = 256 × 256 (ACQ: 256 × 240), FOV = 256 × 240 × 204 mm, voxel size = 1 × 1 × 1.2 mm, slice thickness = 1.2 mm, number of slices = 170.	sagittal
Tokyo C	GE 3.0T Discovery MR750W	T1-weighted images, using a SPGR sequence and a 32-channel coil, were acquired with the following parameters; TR = 8.5 ms, TE Min Full, flip angle = 20 degrees, matrix = 256 × 256 × 176, FOV = 176 × 256 × 256 mm, voxel size = 1 × 1 × 1 mm, slice thickness = 1 mm, number of slices = 176.	axial
Hokkaido B	Philips 3.0T Achieva	T1-weighted images, using a MPRAGE sequence and a SENSE-Head-32 coil, were acquired with the following parameters; TR = 6.8 ms, TE = 3.1 ms, TI = 2500 ms, flip angle = 8 degrees, matrix = 256 × 256, FOV = 256 × 240 × 204 mm, voxel size = 1 × 1 × 1.2 mm, slice thickness = 1.2 mm, number of slices = 170.	sagittal
Hiroshima C	GE 3.0T Signa HDxt	T1-weighted images, using a SPGR sequence and an 8HR brain coil, were acquired with the following parameters; TR = 6812 ms, TE = 1896 ms, TI = 450 ms, flip angle = 20 degrees, matrix = 256 × 256, FOV = 256 × 256 × 184 mm, voxel size = 1 × 1 × 1 mm, slice thickness = 1 mm, number of slices = 184.	sagittal
Tokushima B	GE 3.0T DISCOVERY MR750	T1-weighted images, using a Fast SPGR sequence and an 32-channel coil, were acquired with the following parameters; TR = 6.9 ms, TE = 3 ms, TI = 400 ms, flip angle = 11 degrees, matrix = 512 × 512, FOV = 240 × 240 × 235.2 mm, voxel size = 0.4688 × 0.4688 × 0.6 mm, slice thickness = 1.2 mm, number of slices = 392.	sagittal
Tokushima A	GE 3.0T Signa	T1-weighted images, using a SPGR sequence and a standard quadrature head coil, were acquired with the following parameters; TR = 10 ms, TE = 4.19 ms, flip angle = 15 degrees, matrix = 512 × 512, FOV = 144 × 240 × 240 mm, voxel size = 0.8 × 0.46875 × 0.46875 mm, slice thickness = 1.6 mm, number of slices = 180.	axial
Nagoya B	Siemens 3.0T Verio	T1-weighted images, using a MPRAGE sequence and a 32-channel head coil, were acquired with the following parameters; TR = 2500 ms, TE = 2.48 ms, TI = 900 ms, flip angle = 8 degrees, matrix = 256, FOV = 256 × 256 × 192 mm, voxel size = 0.5 × 0.5 × 1 mm, slice thickness = 1 mm, number of slices = 192.	sagittal

Supplementary Table 3: Means and standard deviations (SDs) of regional volumes for each protocol for each diagnostic group.

(a) Means and SDs of regional volumes for each protocol for healthy controls (HC) in HC vs schizophrenia (SZ) analysis.

Protocol Name	Lhippo		Rhippo		Lamyg		Ramyg		Lthal		Rthal		Laccumb		Raccumb		ICV		Lcaud		Rcaud		Lput		Rput		Lpal		Rpal		LLatVent		RLatVent	
	Mean	SD	Mean	SD	Mean	SD	Mean	SD	Mean	SD	Mean	SD	Mean	SD	Mean	SD	Mean	SD	Mean	SD	Mean	SD	Mean	SD	Mean	SD	Mean	SD	Mean	SD	Mean	SD	Mean	SD
Osaka A	4055	415	4121	421	1390	228	1509	272	7268	946	6479	751	672	126	624	110	1.40E+06	1.54E+05	3550	508	3568	519	5640	834	5539	810	1461	260	1490	233	7601	3836	6435	3099
Osaka C	4213	487	4403	421	1636	210	1939	296	7532	1002	6813	825	768	147	701	118	1.45E+06	1.74E+05	3825	574	4025	599	6199	981	6147	843	1601	322	1687	260	8248	4561	7155	4070
Nippon Med	4262	449	4055	405	1409	168	1315	181	7622	868	6548	696	395	90	473	94	1.48E+06	1.44E+05	3295	440	3176	448	4711	557	4465	525	1440	176	1307	173	6290	2956	7633	3913
Tokyo A	4379	434	4503	410	1520	197	1464	182	8622	965	8102	900	485	100	451	82	1.66E+06	2.03E+05	3764	523	3434	542	5155	631	5046	681	1579	248	1370	201	7510	4336	6403	3851
Osaka B	4331	449	4515	441	1515	197	1480	194	8863	970	8067	890	449	95	472	82	1.61E+06	1.64E+05	3974	564	3510	565	5301	727	5163	688	1443	240	1473	217	8008	4022	6939	3473
Toyama A	4279	497	4270	461	1494	218	1543	218	7611	880	7086	743	567	111	587	107	1.51E+06	1.38E+05	3586	491	3699	505	5565	701	5407	627	1541	291	1549	215	6534	3080	6119	3403
Kanazawa Med	4303	447	4486	418	1539	216	1575	209	8447	949	7759	867	474	92	532	92	1.48E+06	2.01E+05	3760	496	3650	466	5551	815	5321	702	1402	231	1470	183	7980	4753	7322	4873
Kyoto B	4248	398	4483	450	1569	189	1586	195	8083	903	7492	837	396	99	475	86	1.57E+06	1.39E+05	3716	492	3552	507	5321	675	5216	619	1506	218	1515	185	7693	4475	6742	3519
Kyoto A	4463	473	4645	425	1545	202	1584	181	8325	790	7705	773	440	100	527	88	1.60E+06	1.55E+05	3911	501	3678	504	5638	658	5441	574	1647	215	1527	192	7900	4283	6854	3763
Nagoya A	4259	446	4455	434	1692	252	1727	226	7835	1063	7232	911	603	133	563	105	1.45E+06	1.99E+05	3612	487	3704	522	5753	780	5499	657	1437	279	1503	187	8168	4887	7078	4123
Hokkaido A	4264	408	4485	558	1461	210	1454	216	7951	868	7152	682	478	100	454	93	1.54E+06	1.38E+05	3521	335	3228	391	4858	505	4625	510	1315	216	1376	156	9067	5654	7775	4793
Tokyo B	4788	586	4798	683	1436	228	1482	212	8848	795	7794	649	530	99	502	86	1.57E+06	1.22E+05	3556	474	3128	430	5269	670	5077	539	1291	209	1334	177	7644	4653	6555	3841
Kyushu A	4243	381	4448	436	1652	193	1873	254	7860	914	7062	727	572	111	595	99	1.54E+06	1.58E+05	3680	493	3675	483	5742	686	5514	631	1632	229	1578	200	8118	4091	6764	3674
Toyama B	4250	368	4419	413	1785	261	1741	225	8054	836	7624	740	694	105	684	99	1.48E+06	1.16E+05	3836	425	3965	411	6229	845	6090	720	1650	337	1653	188	7785	3889	6428	2985
Yamaguchi A	3728	380	3849	397	1282	166	1309	205	7222	960	6649	829	439	95	390	91	1.38E+06	1.28E+05	3437	425	3284	427	4697	648	4397	624	1270	217	1298	223	8478	4297	7091	3605
Tokyo E	4261	362	4432	420	1815	208	2132	275	7162	912	6265	601	798	113	648	95	1.38E+06	1.14E+05	3331	417	3441	400	6102	764	5716	620	1387	205	1542	138	7478	3757	5937	3049
UOEH	4010	363	4209	389	1510	246	1394	243	7926	789	7247	670	373	110	431	87	1.57E+06	1.37E+05	4066	564	3480	663	5889	895	5427	791	1585	206	1571	188	6566	2448	6012	2801
Kyushu B	4435	313	4579	335	1702	220	1877	228	8107	953	7205	765	571	123	593	112	1.56E+06	1.52E+05	3735	449	3791	467	5932	954	5744	737	1641	261	1646	196	7728	3909	6663	2982
Tokyo D	4329	388	4564	385	1709	177	1905	199	7933	857	6940	546	571	93	582	83	1.50E+06	1.17E+05	3689	488	3695	441	5666	631	5306	568	1513	197	1550	169	9031	5071	7352	3307
Tokyo C	4385	735	4519	474	1326	241	1336	188	8439	822	7389	612	527	126	548	119	1.67E+06	1.38E+05	3536	431	3055	503	5101	554	4862	469	1329	156	1287	142	7545	3567	6251	2920
Hokkaido B	4594	385	4716	384	1800	227	1935	238	8267	916	7218	612	618	102	600	107	1.55E+06	1.30E+05	3639	490	3559	475	6039	661	5614	687	1533	203	1588	198	6305	2400	5791	1869
Tokushima B	3978	369	4037	359	1409	217	1416	294	9376	1054	8236	1048	385	85	388	94	1.46E+06	1.44E+05	5379	1024	4572	912	6671	787	5964	894	1731	222	1679	209	8474	4443	7043	3107
Tokushima A	4689	549	4585	460	1575	307	1516	196	9935	832	7792	633	447	72	459	81	1.67E+06	1.59E+05	3823	343	3398	402	5362	689	4999	673	1376	196	1324	172	7096	2494	7134	3077
Nagoya B	3940	405	4115	423	1394	179	1316	190	6972	909	6753	581	268	65	327	58	1.49E+06	4.42E+05	3565	546	3357	621	4326	741	4369	1047	1483	376	1507	352	13438	5236	11393	4189

Abbreviations: Lhippo, left hippocampus; Rhippo, right hippocampus; Lamyg, left amygdala; Ramyg, right amygdala; Lthal, left thalamus; Rthal, right thalamus; Laccumb, left accumbens; Raccumb, right accumbens; ICV, intracranial volume; Lcaud, left caudate; Rcaud, right caudate; Lput, left putamen; Rput, right putamen; Lpal, left pallidum; Rpal, right pallidum; LLatVent, left lateral ventricle; RLatVent, right lateral ventricle.

(b) Means and SDs of regional volumes for each protocol for SZ in HC vs SZ analysis.

Protocol Name	Lhippo		Rhippo		Lamyg		Ramyg		Lthal		Rthal		Laccumb		Raccumb		ICV		Lcaud		Rcaud		Lput		Rput		Lpal		Rpal		LLatVent		RLatVent	
	Mean	SD	Mean	SD	Mean	SD	Mean	SD	Mean	SD	Mean	SD	Mean	SD	Mean	SD	Mean	SD	Mean	SD	Mean	SD	Mean	SD	Mean	SD	Mean	SD	Mean	SD	Mean	SD	Mean	SD
Osaka A	3787	478	3894	426	1337	224	1462	255	7166	849	6327	749	633	115	584	114	1.42E+06	1.54E+05	3598	476	3654	494	5778	801	5712	742	1594	279	1556	226	10228	5696	8751	5050
Osaka C	3976	461	4121	492	1561	208	1857	345	7149	1010	6509	771	710	143	664	119	1.43E+06	1.56E+05	3736	521	3928	512	6254	870	6127	805	1667	295	1704	233	10833	4992	9298	4691
Nippon Med	3996	544	3745	565	1393	200	1319	200	7563	1081	6373	896	385	90	452	96	1.54E+06	1.74E+05	3633	574	3465	509	5130	714	4967	719	1676	222	1448	223	10044	6652	12009	8097
Tokyo A	4151	505	4221	457	1442	188	1433	200	8044	967	7701	1005	449	78	424	76	1.60E+06	2.40E+05	3754	529	3480	520	5292	725	5219	747	1717	276	1472	226	9244	5587	7713	4003
Osaka B	3959	550	4153	635	1414	220	1412	194	8283	1141	7587	965	433	106	436	100	1.54E+06	1.81E+05	3875	556	3497	524	5294	771	5169	744	1593	293	1505	224	10229	5914	8738	4445
Toyama A	4158	514	4132	460	1442	211	1490	243	7408	870	6892	747	552	113	564	109	1.51E+06	1.64E+05	3614	462	3707	465	5760	797	5595	738	1663	304	1607	239	7239	3415	6993	3148
Kanazawa Med	4116	438	4278	515	1455	202	1495	212	7897	1043	7297	924	433	107	490	100	1.45E+06	2.20E+05	3675	484	3608	484	5449	743	5232	675	1437	253	1481	207	9814	4942	8764	4601
Kyoto B	3936	420	4202	477	1484	194	1509	209	7593	892	6995	730	397	100	434	60	1.53E+06	1.47E+05	3597	530	3478	567	5305	645	5164	622	1681	274	1554	211	10916	5656	9397	4936
Kyoto A	4135	459	4283	485	1493	191	1503	205	7886	774	7359	676	399	103	482	97	1.55E+06	1.25E+05	3798	489	3603	505	5556	620	5446	664	1756	234	1551	175	10052	6326	8592	4930
Nagoya A	3861	473	4039	456	1576	248	1631	236	7152	897	6539	725	608	135	530	104	1.39E+06	1.33E+05	3429	450	3544	484	5633	880	5314	726	1609	360	1615	270	11457	5963	10007	5260
Hokkaido A	3923	437	4096	458	1389	165	1410	186	7997	938	7338	776	459	94	437	93	1.53E+06	1.44E+05	3670	473	3395	494	5145	691	4934	687	1476	263	1436	198	8840	4290	8233	4013
Tokyo B	4576	679	4537	669	1380	248	1479	177	8184	940	7339	850	502	98	462	95	1.56E+06	1.72E+05	3491	530	3095	447	5171	713	4922	663	1389	208	1361	215	9728	4472	8123	3344
Kyushu A	3877	448	4167	439	1534	221	1716	200	7292	691	6493	573	525	119	560	100	1.48E+06	1.50E+05	3503	392	3550	422	5731	618	5490	669	1632	222	1570	188	11270	6796	9016	4188
Toyama B	4031	393	4209	423	1723	228	1771	253	7321	947	6971	736	676	111	640	106	1.43E+06	1.53E+05	3763	511	3915	527	6231	748	6037	666	1679	350	1624	241	8403	3905	7335	4049
Yamaguchi A	3352	448	3473	499	1214	183	1230	186	6877	854	6403	718	389	89	350	69	1.39E+06	1.47E+05	3449	426	3274	515	4827	607	4516	585	1328	253	1343	191	11985	7560	10265	5686
Tokyo E	4254	416	4384	400	1854	218	2119	264	7508	876	6406	571	812	120	673	96	1.46E+06	1.60E+05	3717	466	3859	502	6686	854	6399	620	1652	206	1704	179	11494	8479	8542	5343
UOEH	3717	529	3855	395	1389	219	1441	250	8099	839	6903	659	424	76	446	63	1.51E+06	1.28E+05	4600	479	4318	344	6644	941	5975	514	1918	229	1708	157	8161	4173	6430	2593
Kyushu B	4027	458	4295	644	1585	265	1802	280	7642	877	6842	727	531	92	548	89	1.55E+06	1.56E+05	3658	500	3670	534	5784	725	5646	742	1742	209	1655	223	10734	6227	9004	5413
Tokyo D	4130	442	4346	422	1783	237	1958	287	7515	674	6693	726	563	136	541	94	1.48E+06	1.63E+05	3750	326	3667	361	6071	718	5734	683	1695	225	1578	287	10409	6552	8329	4256
Tokyo C	4029	644	4277	543	1152	294	1239	253	8066	764	7195	627	497	139	458	60	1.58E+06	1.17E+05	3513	334	3005	329	5194	407	4810	306	1368	141	1313	116	9860	6026	7917	3964
Hokkaido B	4041	416	4067	393	1733	241	1821	275	7266	1028	6485	800	598	117	598	125	1.42E+06	1.50E+05	3469	372	3499	321	5689	582	5478	506	1608	231	1541	184	8259	4588	8568	5111
Tokushima B	3744	414	3802	470	1329	194	1311	300	8948	1187	7743	1133	435	140	441	121	1.47E+06	1.10E+05	5537	1081	4690	1072	6785	946	6275	959	1863	302	1782	377	11329	4866	9950	4630
Tokushima A	4361	419	4375	332	1449	221	1414	206	9208	772	7318	563	425	82	421	71	1.65E+06	1.09E+05	3841	439	3347	400	5211	882	5001	730	1514	170	1417	221	8822	4378	7059	3397
Nagoya B	3981	585	4284	522	1388	242	1405	235	8095	948	7282	727	360	74	401	79	1.73E+06	6.68E+05	3641	596	3331	643	4674	749	4701	688	1442	248	1466	295	8470	3877	7698	3525

Abbreviations: Lhippo, left hippocampus; Rhippo, right hippocampus; Lamyg, left amygdala; Ramyg, right amygdala; Lthal, left thalamus; Rthal, right thalamus; Laccumb, left accumbens; Raccumb, right accumbens; ICV, intracranial volume; Lcaud, left caudate; Rcaud, right caudate; Lput, left putamen; Rput, right putamen; Lpal, left pallidum; Rpal, right pallidum; LLatVent, left lateral ventricle; RLatVent, right lateral ventricle.

(c) Means and SDs of regional volumes for each protocol for HC in HC vs bipolar disorder (BP) analysis.

Protocol Name	Lhippo		Rhippo		Lamyg		Ramyg		Lthal		Rthal		Laccumb		Raccumb		ICV		Lcaud		Rcaud		Lput		Rput		Lpal		Rpal		LLatVent		RLatVent	
	Mean	SD	Mean	SD	Mean	SD	Mean	SD	Mean	SD	Mean	SD	Mean	SD	Mean	SD	Mean	SD	Mean	SD	Mean	SD	Mean	SD	Mean	SD	Mean	SD	Mean	SD	Mean	SD	Mean	SD
Osaka C	4213	487	4403	421	1636	210	1939	296	7532	1002	6813	825	768	147	701	118	1.45E+06	1.74E+05	3825	574	4025	599	6199	981	6147	843	1601	322	1687	260	8248	4561	7155	4070
Kanazawa Med	4303	447	4486	418	1539	216	1575	209	8447	949	7759	867	474	92	532	92	1.48E+06	2.01E+05	3760	496	3650	466	5551	815	5321	702	1402	231	1470	183	7980	4753	7322	4873
Nagoya A	4259	446	4455	434	1692	252	1727	226	7835	1063	7232	911	603	133	563	105	1.45E+06	1.99E+05	3612	487	3704	522	5753	780	5499	657	1437	279	1503	187	8168	4887	7078	4123
Yamaguchi B	4200	380	4422	397	1711	166	1691	205	7206	960	6877	829	626	95	640	91	1.45E+06	1.28E+05	3538	425	3705	427	5786	648	5553	624	1528	217	1534	223	9490	4297	8149	3605
Hokkaido A	4264	408	4485	558	1461	210	1454	216	7951	868	7152	682	478	100	454	93	1.54E+06	1.38E+05	3521	335	3228	391	4858	505	4625	510	1315	216	1376	156	9067	5654	7775	4793

Yamaguchi A	3728	380	3849	397	1282	166	1309	205	7222	960	6649	829	439	95	390	91	1.38E+06	1.28E+05	3437	425	3284	427	4697	648	4397	624	1270	217	1298	223	8478	4297	7091	3605
Kyushu A	4243	381	4448	436	1652	193	1873	254	7860	914	7062	727	572	111	595	99	1.54E+06	1.58E+05	3680	493	3675	483	5742	686	5514	631	1632	229	1578	200	8118	4091	6764	3674
Hiroshima D	3987	493	4206	619	1597	230	1667	243	7449	987	7024	854	556	124	567	122	1.50E+06	1.43E+05	3597	433	3733	485	5696	705	5419	773	1593	296	1546	214	12574	6583	10988	6252
Tokyo E	4261	362	4432	420	1815	208	2132	275	7162	912	6265	601	798	113	648	95	1.38E+06	1.14E+05	3331	417	3441	400	6102	764	5716	620	1387	205	1542	138	7478	3757	5937	3049
Kyushu B	4435	313	4579	335	1702	220	1877	228	8107	953	7205	765	571	123	593	112	1.56E+06	1.52E+05	3735	449	3791	467	5932	954	5744	737	1641	261	1646	196	7728	3909	6663	2982

Abbreviations: Lhippo, left hippocampus; Rhippo, right hippocampus; Lamyg, left amygdala; Ramyg, right amygdala; Lthal, left thalamus; Rthal, right thalamus; Laccumb, left accumbens; Raccumb, right accumbens; ICV, intracranial volume; Lcaud, left caudate; Rcaud, right caudate; Lput, left putamen; Rput, right putamen; Lpal, left pallidum; Rpal, right pallidum; LLatVent, left lateral ventricle; RLatVent, right lateral ventricle.

(d) Means and SDs of regional volumes for each protocol for BP in HC vs BP analysis.

Protocol Name	Lhippo		Rhippo		Lamyg		Ramyg		Lthal		Rthal		Laccumb		Raccumb		ICV		Lcaud		Rcaud		Lput		Rput		Lpal		Rpal		LLatVent		RLatVent	
	Mean	SD	Mean	SD	Mean	SD	Mean	SD	Mean	SD	Mean	SD	Mean	SD	Mean	SD	Mean	SD	Mean	SD	Mean	SD	Mean	SD	Mean	SD	Mean	SD	Mean	SD	Mean	SD	Mean	SD
Osaka C	3883	440	4145	477	1667	280	1892	296	6738	856	6208	731	626	57	608	99	1.44E+06	6.27E+04	3924	340	3909	385	5726	577	5474	622	1650	157	1597	157	12966	7360	10938	5614
Kanazawa Med	4036	524	4271	568	1530	252	1553	242	7912	1264	7366	1208	407	94	483	101	1.45E+06	1.48E+05	3598	528	3492	545	5221	847	5100	694	1373	310	1432	218	11136	6140	9590	5332
Nagoya A	3889	547	4129	510	1585	275	1613	207	7630	1194	6807	840	567	78	521	94	1.40E+06	1.31E+05	3585	493	3583	440	5466	629	5338	773	1448	239	1509	185	10997	7512	9438	6352
Yamaguchi B	4377	356	4561	489	1863	227	1832	196	7627	1105	6954	829	651	95	634	93	1.50E+06	1.23E+05	3534	425	3722	409	5786	759	5734	690	1516	168	1574	197	10603	5327	8345	3896
Hokkaido A	3944	432	4091	457	1380	178	1389	170	7821	946	7204	814	442	91	414	96	1.54E+06	1.41E+05	3590	469	3261	509	4855	687	4689	656	1353	228	1324	195	10854	7907	9864	7551
Yamaguchi A	3627	183	3749	188	1173	94	1189	104	7087	712	6476	481	423	68	365	83	1.33E+06	8.10E+04	3436	496	3322	400	4402	399	4249	477	1312	249	1283	119	7701	2911	6581	2585
Kyushu A	4086	474	4214	386	1487	227	1704	251	7014	768	6342	711	516	114	513	89	1.48E+06	1.20E+05	3428	547	3477	488	5232	644	5008	524	1511	236	1450	176	11219	5183	10276	4822
Hiroshima D	3831	472	4053	496	1622	238	1687	280	7296	1241	6962	1035	558	110	562	117	1.50E+06	1.65E+05	3628	466	3756	439	5718	708	5450	673	1678	285	1559	222	13348	5361	12225	4572
Tokyo E	4282	566	4508	474	1846	210	2134	311	7390	1237	6325	680	855	158	667	92	1.42E+06	9.88E+04	3716	447	3819	420	6509	1025	6253	812	1509	279	1632	198	10263	4820	8321	4294
Kyushu B	4286	383	4360	387	1554	160	1747	217	7266	813	6616	667	497	85	523	104	1.53E+06	1.23E+05	3703	366	3682	372	5448	520	5243	552	1575	201	1440	201	13541	6782	12457	6672

Abbreviations: Lhippo, left hippocampus; Rhippo, right hippocampus; Lamyg, left amygdala; Ramyg, right amygdala; Lthal, left thalamus; Rthal, right thalamus; Laccumb, left accumbens; Raccumb, right accumbens; ICV, intracranial volume; Lcaud, left caudate; Rcaud, right caudate; Lput, left putamen; Rput, right putamen; Lpal, left pallidum; Rpal, right pallidum; LLatVent, left lateral ventricle; RLatVent, right lateral ventricle.

(e) Means and SDs of regional volumes for each protocol for HC in HC vs major depressive disorder (MDD) analysis.

Protocol Name	Lhippo		Rhippo		Lamyg		Ramyg		Lthal		Rthal		Laccumb		Raccumb		ICV		Lcaud		Rcaud		Lput		Rput		Lpal		Rpal		LLatVent		RLatVent	
	Mean	SD	Mean	SD	Mean	SD	Mean	SD	Mean	SD	Mean	SD	Mean	SD	Mean	SD	Mean	SD	Mean	SD	Mean	SD	Mean	SD	Mean	SD	Mean	SD	Mean	SD	Mean	SD	Mean	SD
Osaka C	4213	487	4403	421	1636	210	1939	296	7532	1002	6813	825	768	147	701	118	1.45E+06	1.74E+05	3825	574	4025	599	6199	981	6147	843	1601	322	1687	260	8248	4561	7155	4070
Osaka A	4055	415	4121	421	1390	228	1509	272	7268	946	6479	751	672	126	624	110	1.40E+06	1.54E+05	3550	508	3568	519	5640	834	5539	810	1461	260	1490	233	7601	3836	6435	3099
Osaka B	4331	449	4515	441	1515	197	1480	194	8863	970	8067	890	449	95	472	82	1.61E+06	1.64E+05	3974	564	3510	565	5301	727	5163	688	1443	240	1473	217	8008	4022	6939	3473
Hokkaido A	4264	408	4485	558	1461	210	1454	216	7951	868	7152	682	478	100	454	93	1.54E+06	1.38E+05	3521	335	3228	391	4858	505	4625	510	1315	216	1376	156	9067	5654	7775	4793
Yamaguchi B	4200	510	4422	480	1711	284	1691	251	7206	1150	6877	890	626	125	640	119	1.45E+06	1.71E+05	3538	460	3705	455	5786	794	5553	755	1528	287	1534	209	9490	5877	8149	5263
Kanazawa Med	4303	447	4486	418	1539	216	1575	209	8447	949	7759	867	474	92	532	92	1.48E+06	2.01E+05	3760	496	3650	466	5551	815	5321	702	1402	231	1470	183	7980	4753	7322	4873
Hiroshima A	4715	536	4700	450	1462	210	1476	162	8467	1156	7363	863	479	88	492	86	1.54E+06	1.47E+05	3556	394	3207	402	5339	611	4990	588	1241	170	1317	184	7801	4051	6954	3602
Hiroshima D	3987	493	4206	619	1597	230	1667	243	7449	987	7024	854	556	124	567	122	1.50E+06	1.43E+05	3597	433	3733	485	5696	705	5419	773	1593	296	1546	214	12574	6583	10988	6252
Yamaguchi A	3728	380	3849	397	1282	166	1309	205	7222	960	6649	829	439	95	390	91	1.38E+06	1.28E+05	3437	425	3284	427	4697	648	4397	624	1270	217	1298	223	8478	4297	7091	3605
Kyushu A	4243	381	4448	436	1652	193	1873	254	7860	914	7062	727	572	111	595	99	1.54E+06	1.58E+05	3680	493	3675	483	5742	686	5514	631	1632	229	1578	200	8118	4091	6764	3674
Tokyo E	4261	362	4432	420	1815	208	2132	275	7162	912	6265	601	798	113	648	95	1.38E+06	1.14E+05	3331	417	3441	400	6102	764	5716	620	1387	205	1542	138	7478	3757	5937	3049
UOEH	4010	363	4209	389	1510	246	1394	243	7926	789	7247	670	373	110	431	87	1.57E+06	1.37E+05	4066	564	3480	663	5889	895	5427	791	1585	206	1571	188	6566	2448	6012	2801
Hiroshima B	4269	460	4466	434	1580	162	1481	172	8116	992	7527	745	449	99	495	98	1.46E+06	1.45E+05	3548	523	3497	542	5286	716	5006	540	1356	229	1405	175	9522	4742	8541	4900
Hiroshima C	4265	668	4276	525	1320	175	1355	236	7779	893	6841	762	478	87	462	78	1.54E+06	2.04E+05	3190	401	2739	316	4433	577	4102	541	1033	163	1024	187	8315	4249	6788	2765

Abbreviations: Lhippo, left hippocampus; Rhippo, right hippocampus; Lamyg, left amygdala; Ramyg, right amygdala; Lthal, left thalamus; Rthal, right thalamus; Laccumb, left accumbens; Raccumb, right accumbens; ICV, intracranial volume; Lcaud, left caudate; Rcaud, right caudate; Lput, left putamen; Rput, right putamen; Lpal, left pallidum; Rpal, right pallidum; LLatVent, left lateral ventricle; RLatVent, right lateral ventricle.

(f) Means and SDs of regional volumes for each protocol for MDD in HC vs MDD analysis.

Protocol Name	Lhippo		Rhippo		Lamyg		Ramyg		Lthal		Rthal		Laccumb		Raccumb		ICV		Lcaud		Rcaud		Lput		Rput		Lpal		Rpal		LLatVent		RLatVent	
	Mean	SD	Mean	SD	Mean	SD	Mean	SD	Mean	SD	Mean	SD	Mean	SD	Mean	SD	Mean	SD	Mean	SD	Mean	SD	Mean	SD	Mean	SD	Mean	SD	Mean	SD	Mean	SD	Mean	SD
Osaka C	3764	494	3970	540	1472	192	1735	254	6884	1069	6113	764	651	140	606	105	1.42E+06	1.13E+05	3545	565	3628	460	5291	1046	5343	779	1428	257	1516	190	13290	6277	11150	5477
Osaka A	4125	504	4116	498	1378	234	1532	370	7271	614	6404	616	738	130	645	157	1.41E+06	1.48E+05	3656	460	3709	477	5578	676	5790	817	1567	289	1500	255	7061	2437	6124	2145
Osaka B	3828	538	4280	336	1370	156	1400	135	7459	611	7180	644	409	78	434	96	1.49E+06	1.51E+05	3619	505	3344	341	4966	463	4642	485	1474	148	1419	133	11398	5990	10032	4985
Hokkaido A	3854	535	4003	541	1360	209	1367	200	7733	1088	7079	909	434	101	406	96	1.54E+06	1.52E+05	3551	513	3287	489	4795	682	4585	667	1333	225	1314	206	11649	7155	10026	6198
Yamaguchi B	4077	483	4306	531	1650	206	1684	185	7121	863	6593	578	558	118	582	106	1.44E+06	1.51E+05	3466	506	3642	485	5511	697	5311	704	1471	291	1510	189	10943	5913	9597	4898
Kanazawa Med	4181	407	4368	449	1531	226	1538	193	8139	1170	7336	1007	420	98	484	88	1.48E+06	2.86E+05	3580	536	3512	541	5277	720	5114	637	1355	229	1449	177	10186	7631	9118	6208
Hiroshima A	4503	762	4535	676	1362	217	1407	275	8184	1112	7056	845	443	87	456	83	1.48E+06	1.45E+05	3375	362	3076	361	4928	652	4636	559	1169	179	1240	189	9148	5335	8019	4412
Hiroshima D	3933	556	4138	509	1589	252	1629	281	7271	1161	7046	1009	586	130	552	100	1.46E+06	1.55E+05	3629	511	3693	468	5833	854	5531	771	1569	297	1486	215	11641	7499	10334	8181
Yamaguchi A	3706	385	3883	371	1300	153	1352	208	7057	783	6481	704	403	78	371	86	1.39E+06	1.11E+05	3472	411	3372	396	4604	564	4325	496	1273	300	1267	154	9496	3730	7588	2682
Kyushu A	3621	366	3813	479	1496	241	1611	245	6431	766	6105	855	457	118	448	108	1.45E+06	1.16E+05	3057	398	3072	306	5006	390	4622	384	1382	163	1401	152	11666	4240	9845	4526
Tokyo E	4364	494	4564	484	1880	231	2171	284	7339	1020	6435	700	856	139	693	120	1.47E+06	2.50E+05	3548	401	3702	418	6410	701	6017	779	1485	191	1619	174	10044	5865	7751	4680
UOEH	3844	422	3975	445	1407	212	1221	223	7432	1047	6876	840	290	104	389	69	1.53E+06	1.56E+05	3761	492	3175	486	5244	629	4867	615	1433	196	1429	212	7566	3309	6932	2694
Hiroshima B	4144	398	4397	412	1549	202	1536	210	8091	923	7344	735	450	95	483	79	1.45E+06	1.58E+05	3582	385	3542	408	5330	596	4992	508	1370	239	1407	178	9225	4610	8317	4140
Hiroshima C	4329	362	4327	527	1340	329	1368	269	8022	1054	7500	1221	463	75	520	98	1.51E+06	1.75E+05	3236	317	2711	264	4779	675	4255	748	1066	228	1037	164	11171	6011	8474	4423

Abbreviations: Lhippo, left hippocampus; Rhippo, right hippocampus; Lamyg, left amygdala; Ramyg, right amygdala; Lthal, left thalamus; Rthal, right thalamus; Laccumb, left accumbens; Raccumb, right accumbens; ICV, intracranial volume; Lcaud, left caudate; Rcaud, right caudate; Lput, left putamen; Rput, right putamen; Lpal, left pallidum; Rpal, right pallidum; LLatVent, left lateral ventricle; RLatVent, right lateral ventricle.

(g) Means and SDs of regional volumes for each protocol for HC in HC vs autism spectrum disorder (ASD) analysis.

Protocol Name	Lhippo		Rhippo		Lamyg		Ramyg		Lthal		Rthal		Laccumb		Raccumb		ICV		Lcaud		Rcaud		Lput		Rput		Lpal		Rpal		LLatVent		RLatVent	
	Mean	SD	Mean	SD	Mean	SD	Mean	SD	Mean	SD	Mean	SD	Mean	SD	Mean	SD	Mean	SD	Mean	SD	Mean	SD	Mean	SD	Mean	SD	Mean	SD	Mean	SD	Mean	SD	Mean	SD
Osaka A	4055	415	4121	421	1390	228	1509	272	7268	946	6479	751	672	126	624	110	1.40E+06	1.54E+05	3550	508	3568	519	5640	834	5539	810	1461	260	1490	233	7601	3836	6435	3099
Osaka C	4213	487	4403	421	1636	210	1939	296	7532	1002	6813	825	768	147	701	118	1.45E+06	1.74E+05	3825	574	4025	599	6199	981	6147	843	1601	322	1687	260	8248	4561	7155	4070
Osaka B	4331	449	4515	441	1515	197	1480	194	8863	970	8067	890	449	95	472	82	1.61E+06	1.64E+05	3974	564	3510	565	5301	727	5163	688	1443	240	1473	217	8008	4022	6939	3473

Showa	4424	385	4723	420	1721	212	1729	203	9112	930	8675	810	519	103	578	87	1.60E+06	1.27E+05	4044	522	4015	509	6247	773	5953	769	1771	297	1641	256	7323	3714	6404	3420
Nagoya A	4259	446	4455	434	1692	252	1727	226	7835	1063	7232	911	603	133	563	105	1.45E+06	1.99E+05	3612	487	3704	522	5753	780	5499	657	1437	279	1503	187	8168	4887	7078	4123
Tokyo B	4788	586	4798	683	1436	228	1482	212	8848	795	7794	649	530	99	502	86	1.57E+06	1.22E+05	3556	474	3128	430	5269	670	5077	539	1291	209	1334	177	7644	4653	6555	3841
Tokyo E	4261	362	4432	420	1815	208	2132	275	7162	912	6265	601	798	113	648	95	1.38E+06	1.14E+05	3331	417	3441	400	6102	764	5716	620	1387	205	1542	138	7478	3757	5937	3049

Abbreviations: Lhippo, left hippocampus; Rhippo, right hippocampus; Lamyg, left amygdala; Ramyg, right amygdala; Lthal, left thalamus; Rthal, right thalamus; Laccumb, left accumbens; Raccumb, right accumbens; ICV, intracranial volume; Lcaud, left caudate; Rcaud, right caudate; Lput, left putamen; Rput, right putamen; Lpal, left pallidum; Rpal, right pallidum; LLatVent, left lateral ventricle; RLatVent, right lateral ventricle.

(h) Means and SDs of regional volumes for each protocol for ASD in HC vs ASD analysis.

Protocol Name	Lhippo		Rhippo		Lamyg		Ramyg		Lthal		Rthal		Laccumb		Raccumb		ICV		Lcaud		Rcaud		Lput		Rput		Lpal		Rpal		LLatVent		RLatVent	
	Mean	SD	Mean	SD	Mean	SD	Mean	SD	Mean	SD	Mean	SD	Mean	SD	Mean	SD	Mean	SD	Mean	SD	Mean	SD	Mean	SD	Mean	SD	Mean	SD	Mean	SD	Mean	SD	Mean	SD
Osaka A	4072	404	4101	442	1426	190	1559	275	7386	936	6468	762	699	124	638	129	1.41E+06	1.56E+05	3683	484	3729	527	6012	721	5909	681	1589	286	1565	224	8477	4426	7049	4111
Osaka C	4396	417	4403	517	1652	156	1933	218	7769	1231	6749	800	802	112	688	127	1.46E+06	1.76E+05	4049	637	4178	671	6414	773	6365	642	1583	265	1638	249	8668	5573	6944	4170
Osaka B	4323	281	4480	363	1545	221	1506	97	8913	719	8147	569	425	44	462	77	1.66E+06	1.45E+05	3824	541	3564	700	5196	465	5090	434	1484	265	1572	247	9032	9610	7811	8565
Showa	4405	429	4745	462	1767	226	1752	227	9007	923	8704	935	517	97	577	87	1.61E+06	1.59E+05	4058	543	4054	537	6241	693	5987	660	1731	305	1636	233	8182	4390	6742	2977
Nagoya A	4422	214	4461	355	1816	227	1843	207	8236	642	7866	772	599	101	568	77	1.50E+06	8.69E+04	3912	495	4075	599	6219	935	5853	779	1557	186	1560	219	8532	3383	8378	4361
Tokyo B	4731	760	5026	439	1439	259	1549	159	8866	869	7902	768	522	79	494	94	1.61E+06	1.14E+05	3605	503	3145	567	5356	675	5062	659	1354	217	1336	186	9024	5137	7494	5038
Tokyo E	4542	238	4541	328	1876	122	2288	252	7929	1088	6478	389	951	129	738	56	1.50E+06	8.16E+04	3477	445	3724	516	6210	1019	6221	690	1529	273	1588	189	9004	3486	8980	3590

Abbreviations: Lhippo, left hippocampus; Rhippo, right hippocampus; Lamyg, left amygdala; Ramyg, right amygdala; Lthal, left thalamus; Rthal, right thalamus; Laccumb, left accumbens; Raccumb, right accumbens; ICV, intracranial volume; Lcaud, left caudate; Rcaud, right caudate; Lput, left putamen; Rput, right putamen; Lpal, left pallidum; Rpal, right pallidum; LLatVent, left lateral ventricle; RLatVent, right lateral ventricle.

Supplementary Table 4: Group differences in regional volumes within each protocol.

(a) Group differences and standard errors (SEs) in regional volumes within each protocol in healthy controls (HC) vs schizophrenia (SZ) analysis.

Protocol Name	Lhippo		Rhippo		Lamyg		Ramyg		Lthal		Rthal		Laccumb		Raccumb		ICV		Lcaud		Rcaud		Lput		Rput		Lpal		Rpal		LLatVent		RLatVent	
	Beta	SE	Beta	SE	Beta	SE	Beta	SE	Beta	SE	Beta	SE	Beta	SE	Beta	SE	Beta	SE	Beta	SE	Beta	SE	Beta	SE	Beta	SE	Beta	SE	Beta	SE	Beta	SE	Beta	SE
Osaka A	-292	33	-253	32	-74	17	-73	20	-129	60	-186	47	-46	9	-41	9	-2.98E+03	1.12E+04	24	33	57	34	106	56	131	50	114	19	55	17	2374	344	2098	295
Osaka C	-212	50	-254	43	-62	22	-63	30	-304	89	-230	71	-48	15	-27	11	-1.82E+04	1.79E+04	-46	52	-53	55	138	84	54	68	87	30	37	22	2524	479	2072	427
Nippon Med	-397	41	-443	40	-72	16	-62	16	-391	66	-426	54	-24	9	-37	9	-2.00E+04	1.40E+04	284	45	250	44	268	55	339	55	191	19	90	18	4057	451	4688	567
Tokyo A	-181	47	-235	43	-52	18	-10	18	-481	83	-297	77	-30	10	-23	9	-4.22E+04	1.99E+04	50	47	108	49	185	62	226	64	153	23	111	21	2437	493	1857	410
Osaka B	-255	59	-231	58	-43	23	-13	22	-294	112	-195	96	6	13	-19	11	-2.96E+04	2.00E+04	59	66	137	67	230	75	233	68	213	31	95	25	2481	599	2062	489
Toyama A	-111	57	-128	50	-46	23	-48	24	-195	83	-183	74	-12	14	-21	13	3.85E+03	1.58E+04	32	52	15	51	211	82	205	71	129	36	61	26	666	370	841	378
Kanazawa Med	-76	55	-94	59	-21	26	-10	25	-207	112	-119	95	-17	13	-15	12	1.38E+04	2.71E+04	44	60	92	57	161	91	175	75	75	33	56	25	1332	620	968	604
Kyoto B	-262	65	-218	73	-51	29	-36	30	-302	125	-303	101	11	17	-24	13	-1.37E+04	1.98E+04	-5	67	39	74	145	93	122	78	211	35	74	28	3222	690	2663	579
Kyoto A	-210	60	-240	54	13	22	-29	23	-180	86	-101	77	-26	15	-26	12	-3.95E+04	1.73E+04	35	57	73	57	120	77	183	74	161	28	72	22	2099	692	1714	557
Nagoya A	-276	69	-315	67	-71	39	-53	35	-321	143	-353	115	35	22	2	16	-3.81E+04	2.88E+04	-27	69	17	72	137	124	71	96	245	49	130	35	2557	828	2398	711
Hokkaido A	-446	76	-485	85	-79	29	-68	32	-146	156	18	115	-47	17	-54	17	-3.19E+04	2.72E+04	88	73	130	78	130	107	93	105	92	46	28	34	1827	835	2261	778
Tokyo B	-173	113	-231	118	-46	39	-2	34	-601	135	-403	111	-19	18	-29	15	-4.24E+03	2.29E+04	-14	89	14	78	-31	124	-87	95	122	36	53	31	1777	847	1181	675
Kyushu A	-265	69	-168	67	-63	34	-82	35	-252	115	-321	88	-33	22	-18	18	1.36E+02	2.01E+04	-46	72	-19	77	196	106	206	97	53	40	53	31	3354	964	2194	696
Toyama B	-131	62	-105	64	-7	37	81	37	-538	141	-422	94	-2	20	-24	18	-4.46E+04	2.37E+04	55	70	74	71	206	121	124	104	77	62	16	37	1362	633	1520	591
Yamaguchi A	-354	75	-355	82	-59	33	-64	39	-203	141	-112	122	-34	16	-27	15	2.65E+04	2.32E+04	47	82	12	92	197	116	187	106	52	45	36	43	2465	993	2137	772
Tokyo E	-221	92	-292	90	-38	56	-130	68	-173	192	-257	122	-35	29	-29	20	4.74E+04	3.33E+04	191	101	207	98	193	196	362	153	190	53	118	41	3518	1330	2694	838
UOEH	-246	118	-266	101	-51	61	67	57	248	192	-206	151	8	28	-15	21	-9.04E+03	3.20E+04	486	153	685	165	740	244	403	184	304	57	138	52	2581	805	1436	753
Kyushu B	-401	96	-268	118	-108	49	-67	54	-416	157	-325	129	-33	26	-43	24	-2.24E+04	3.21E+04	-27	94	-61	105	-110	183	-92	149	105	51	24	38	3151	1067	2423	896
Tokyo D	-201	119	-253	110	69	59	52	69	-381	267	-197	166	5	33	-43	27	-4.14E+04	3.73E+04	100	140	6	125	421	174	420	180	159	60	19	57	1850	1555	1109	1048
Tokyo C	-360	255	-210	164	-120	85	-44	68	-93	235	61	170	-21	44	-90	38	-7.81E+04	3.68E+04	156	105	126	130	142	170	23	134	69	50	45	46	4199	1221	3014	922
Hokkaido B	-370	110	-518	105	32	65	-5	69	-296	185	-221	140	-4	36	33	36	-5.56E+04	3.37E+04	34	116	124	113	9	159	159	158	185	59	54	50	2466	1103	3428	1242
Tokushima B	-235	113	-235	113	-82	59	-104	89	-453	319	-490	326	50	38	57	32	1.21E+04	3.42E+04	95	274	113	291	78	205	308	198	131	66	109	94	2451	1109	2582	931
Tokushima A	-276	132	-157	119	-71	72	-71	59	-683	219	-450	194	-6	24	-25	22	2.63E+04	3.89E+04	67	114	-45	130	116	152	201	138	197	39	109	49	1636	1081	-47	964
Nagoya B	-145	381	-151	353	-184	135	74	153	618	681	-643	402	4	50	40	52	1.03E+06	3.37E+05	-385	396	-515	437	-291	541	-344	614	-134	215	-178	222	-2531	2721	-1289	2316

Abbreviations: Lhippo, left hippocampus; Rhippo, right hippocampus; Lamyg, left amygdala; Ramyg, right amygdala; Lthal, left thalamus; Rthal, right thalamus; Laccumb, left accumbens; Raccumb, right accumbens; ICV, intracranial volume; Lcaud, left caudate; Rcaud, right caudate; Lput, left putamen; Rput, right putamen; Lpal, left pallidum; Rpal, right pallidum; LLatVent, left lateral ventricle; RLatVent, right lateral ventricle.

(b) Group differences and SEs in regional volumes within each protocol in HC vs bipolar disorder (BP) analysis.

Protocol Name	Lhippo		Rhippo		Lamyg		Ramyg		Lthal		Rthal		Laccumb		Raccumb		ICV		Lcaud		Rcaud		Lput		Rput		Lpal		Rpal		LLatVent		RLatVent	
	Beta	SE	Beta	SE	Beta	SE	Beta	SE	Beta	SE	Beta	SE	Beta	SE	Beta	SE	Beta	SE	Beta	SE	Beta	SE	Beta	SE	Beta	SE	Beta	SE	Beta	SE	Beta	SE	Beta	SE
Osaka C	-230	202	-202	165	98	88	21	117	-411	349	-170	282	-79	59	-44	43	-4.57E+04	7.24E+04	216	213	32	227	24	334	-239	271	98	119	-4	90	2068	1872	1167	1640
Kanazawa Med	-112	84	-62	83	76	39	54	37	-73	170	29	153	-32	17	-9	17	1.65E+02	3.56E+04	54	86	42	83	104	128	183	97	26	49	26	35	1524	921	569	902
Nagoya A	-151	106	-136	102	-14	61	-36	55	431	226	150	179	8	32	20	23	1.00E+04	4.70E+04	212	106	136	111	73	180	303	137	93	70	47	47	447	1246	455	1059
Yamaguchi B	79	107	43	109	90	62	88	56	157	206	-169	132	2	26	-27	23	1.71E+04	4.04E+04	-93	102	-75	98	-169	166	3	133	-56	70	-1	46	1046	1289	140	1184
Hokkaido A	-337	67	-407	80	-87	31	-69	30	-172	135	12	112	-40	17	-41	16	-1.43E+04	2.69E+04	76	78	53	84	-42	100	31	96	22	42	-54	32	2440	1209	2634	1145
Yamaguchi A	-68	114	-70	121	-84	52	-93	65	-10	238	-63	199	0	27	-12	25	-1.44E+04	3.72E+04	102	126	137	130	-165	188	5	171	81	73	14	72	-333	1298	-242	1045
Kyushu A	-10	90	-125	92	-67	43	-58	51	-292	160	-296	130	-1	29	-50	26	4.78E+03	2.86E+04	6	109	15	112	-114	149	-120	119	-35	57	-21	42	2166	1085	2429	990
Hiroshima D	-236	90	-236	111	0	48	12	53	-325	171	-192	131	-15	25	-16	23	2.50E+04	3.18E+04	24	94	17	96	-24	135	-12	134	107	58	11	40	1978	1145	2194	1078
Tokyo E	-102	103	-54	98	-5	56	-65	74	-52	252	-155	130	43	32	3	20	1.47E+04	2.82E+04	288	104	284	96	201	182	382	163	85	62	51	41	2974	1017	2650	809
Kyushu B	-127	125	-232	127	-94	65	-113	77	-541	300	-312	239	-17	47	-32	47	-3.71E+04	3.88E+04	210	137	91	167	-86	340	-201	236	74	87	-86	55	4370	1706	4263	1411

Abbreviations: Lhippo, left hippocampus; Rhippo, right hippocampus; Lamyg, left amygdala; Ramyg, right amygdala; Lthal, left thalamus; Rthal, right thalamus; Laccumb, left accumbens; Raccumb, right accumbens; ICV, intracranial volume; Lcaud, left caudate; Rcaud, right caudate; Lput, left putamen; Rput, right putamen; Lpal, left pallidum; Rpal, right pallidum; LLatVent, left lateral ventricle; RLatVent, right lateral ventricle.

(c) Group differences and SEs in regional volumes within each protocol in HC vs major depressive disorder (MDD) analysis.

Protocol Name	Lhippo		Rhippo		Lamyg		Ramyg		Lthal		Rthal		Laccumb		Raccumb		ICV		Lcaud		Rcaud		Lput		Rput		Lpal		Rpal		LLatVent		RLatVent	
	Beta	SE	Beta	SE	Beta	SE	Beta	SE	Beta	SE	Beta	SE	Beta	SE	Beta	SE	Beta	SE	Beta	SE	Beta	SE	Beta	SE	Beta	SE	Beta	SE	Beta	SE	Beta	SE	Beta	SE
Osaka C	-274	106	-270	87	-91	46	-79	61	-57	184	-173	147	-46	31	-28	23	7.13E+03	3.79E+04	-27	113	-123	118	-278	176	-226	142	-34	63	-22	47	3280	991	2310	871
Osaka A	-11	95	-67	92	-59	52	-45	62	-293	187	-321	141	26	29	-12	26	-3.61E+04	3.38E+04	-30	97	6	100	-373	168	-86	154	21	56	-73	48	456	870	453	703
Osaka B	-142	138	85	128	-3	55	24	56	-242	258	109	226	25	30	38	27	-5.85E+04	4.70E+04	163	165	320	167	465	184	312	166	176	72	98	63	965	1289	1220	1107
Hokkaido A	-402	76	-475	79	-103	31	-86	30	-206	137	-74	106	-44	15	-48	14	-1.70E+04	2.23E+04	27	74	59	74	-74	94	-48	87	16	38	-61	31	2628	1056	2298	907
Yamaguchi B	-22	68	-36	71	-24	35	26	32	192	119	-77	78	-40	16	-29	14	-3.28E+03	2.54E+04	6	65	14	63	-126	95	-60	82	-25	44	14	27	-6	760	223	696
Kanazawa Med	-49	71	-49	69	34	33	-3	31	-37	151	-179	128	-32	16	-28	15	-1.45E+04	3.93E+04	-57	80	-39	77	-20	115	38	88	-15	40	8	31	820	849	449	805
Hiroshima A	-97	115	-33	99	-56	37	-19	38	105	164	-23	123	-9	14	-8	14	-4.23E+04	2.34E+04	-97	65	-83	66	-215	102	-162	83	-59	31	-59	33	228	805	-6	656
Hiroshima D	-69	84	-94	90	-9	43	-3	43	-203	152	6	117	17	23	-22	20	-3.20E+04	2.54E+04	90	81	17	77	166	123	133	116	13	53	-48	36	1120	1261	1408	1302
Yamaguchi A	13	78	55	84	21	34	46	43	-99	160	-89	136	-36	18	-19	17	-2.28E+04	2.52E+04	4	80	53	85	-110	125	-111	113	-8	52	-24	48	274	861	173	690
Kyushu A	-404	108	-400	116	-41	56	-98	64	-558	206	-303	176	-38	39	-90	35	-6.09E+04	3.65E+04	-271	143	-292	149	-245	199	-389	161	-147	74	-56	57	3101	1431	1911	1308
Tokyo E	22	86	30	88	24	44	-10	54	21	196	62	129	43	26	31	21	6.31E+04	4.03E+04	138	81	164	77	246	149	234	144	66	41	50	32	2063	993	1329	778
UOEH	-109	93	-143	94	-37	52	-88	55	-162	167	-76	141	-61	26	-15	19	-3.68E+03	3.01E+04	-142	126	-116	142	-347	185	-291	164	-85	48	-99	46	1090	632	953	653
Hiroshima B	-117	105	-78	103	-31	48	55	47	87	190	-123	159	6	26	-6	22	-2.18E+04	4.06E+04	64	111	80	115	56	157	4	119	-6	61	4	44	-718	1054	-557	971
Hiroshima C	50	245	135	202	39	73	35	96	210	310	629	275	-17	31	51	31	-2.34E+04	7.42E+04	16	123	-46	110	315	187	176	174	30	62	16	72	3561	1771	2414	1130

Abbreviations: Lhippo, left hippocampus; Rhippo, right hippocampus; Lamyg, left amygdala; Ramyg, right amygdala; Lthal, left thalamus; Rthal, right thalamus; Laccumb, left accumbens; Raccumb, right accumbens; ICV, intracranial volume; Lcaud, left caudate; Rcaud, right caudate; Lput, left putamen; Rput, right putamen; Lpal, left pallidum; Rpal, right pallidum; LLatVent, left lateral ventricle; RLatVent, right lateral ventricle.

(d) Group differences and SEs in regional volumes within each protocol in HC vs autism spectrum disorder (ASD) analysis.

Protocol Name	Lhippo	Rhippo	Lamyg	Ramyg	Lthal	Rthal	Laccumb	Raccumb	ICV	Lcaud	Rcaud	Lput	Rput	Lpal	Rpal	LLatVent	RLatVent

	Beta	SE	Beta	SE	Beta	SE	Beta	SE	Beta	SE	Beta	SE	Beta	SE	Beta	SE	Beta	SE	Beta	SE	Beta	SE	Beta	SE	Beta	SE	Beta	SE	Beta	SE	Beta	SE
Osaka A	-79	57	-89	56	-16	30	-31	37	-268	113	-327	85	-25	17	-31	16	-4.68E+04	2.02E+04	-38	60	-7	61	-35	100	-63	90	19	34	-31	29	2380	530	1813	438
Osaka C	135	106	-43	88	-4	46	-42	62	69	189	-214	150	14	31	-32	23	-1.15E+03	3.84E+04	151	114	75	121	36	177	50	143	-59	63	-92	48	930	991	276	865
Osaka B	-117	108	-142	103	-18	47	-15	46	-238	214	-194	184	-44	24	-28	22	2.28E+04	3.82E+04	-294	136	-83	142	-329	150	-300	132	-12	59	47	52	1136	1148	911	997
Showa	-35	60	-5	65	28	34	19	33	-143	139	-35	119	-4	17	1	13	1.79E+04	2.19E+04	29	73	61	72	14	94	54	96	-21	45	11	37	408	661	-76	526
Nagoya A	-22	116	-171	116	29	70	42	65	-63	261	208	214	-41	39	-37	27	-1.72E+04	5.61E+04	104	127	174	136	164	221	15	165	23	80	17	57	1000	1363	1750	1205
Tokyo B	-112	140	87	125	-51	49	15	39	-196	161	-56	128	-25	20	-21	18	-1.34E+04	2.34E+04	-8	99	-62	97	-33	138	-174	110	20	42	-45	34	792	984	127	881
Tokyo E	76	144	-139	167	23	97	45	119	166	348	-118	206	139	49	98	31	6.28E+04	4.79E+04	-131	172	-22	163	-20	303	422	245	54	101	-33	65	307	1587	1971	1316

Abbreviations: Lhippo, left hippocampus; Rhippo, right hippocampus; Lamyg, left amygdala; Ramyg, right amygdala; Lthal, left thalamus; Rthal, right thalamus; Laccumb, left accumbens; Raccumb, right accumbens; ICV, intracranial volume; Lcaud, left caudate; Rcaud, right caudate; Lput, left putamen; Rput, right putamen; Lpal, left pallidum; Rpal, right pallidum; LLatVent, left lateral ventricle; RLatVent, right lateral ventricle.

Supplementary Table 5: Cohen's *d* effect sizes for group differences in regional volumes within each protocol in each psychiatric disorder.

(a) Cohen's *d* effect sizes and standard errors (SEs) for group differences in regional volumes within each protocol in healthy controls (HC) vs schizophrenia (SZ) analysis.

Protocol Name	Lhippo		Rhippo		Lamyg		Ramyg		Lthal		Rthal		Laccumb		Raccumb		ICV		Lcaud		Rcaud		Lput		Rput		Lpal		Rpal		LLatVent		RLatVent	
	d	SE	*d*	SE	*d*	SE	*d*	SE	*d*	SE	*d*	SE	*d*	SE	*d*	SE	*d*	SE	*d*	SE	*d*	SE	*d*	SE	*d*	SE	*d*	SE	*d*	SE	*d*	SE	*d*	SE
Osaka A	-0.67	0.08	-0.60	0.08	-0.33	0.08	-0.27	0.08	-0.14	0.07	-0.25	0.06	-0.37	0.07	-0.37	0.08	-0.02	0.07	0.05	0.07	0.11	0.07	0.13	0.07	0.17	0.06	0.43	0.07	0.24	0.07	0.53	0.08	0.55	0.08
Osaka C	-0.44	0.10	-0.58	0.10	-0.30	0.11	-0.21	0.10	-0.30	0.09	-0.28	0.09	-0.33	0.10	-0.23	0.09	-0.11	0.10	-0.08	0.09	-0.09	0.09	0.14	0.09	0.06	0.08	0.28	0.09	0.15	0.09	0.54	0.10	0.49	0.10
Nippon Med	-0.79	0.08	-0.89	0.08	-0.39	0.09	-0.32	0.08	-0.40	0.07	-0.53	0.07	-0.26	0.10	-0.39	0.10	-0.12	0.09	0.55	0.09	0.52	0.09	0.42	0.08	0.53	0.09	0.95	0.10	0.45	0.09	0.78	0.09	0.73	0.09
Tokyo A	-0.40	0.10	-0.55	0.10	-0.27	0.09	-0.05	0.10	-0.50	0.09	-0.32	0.08	-0.32	0.10	-0.29	0.11	-0.20	0.09	0.10	0.09	0.20	0.09	0.28	0.09	0.32	0.09	0.60	0.09	0.53	0.10	0.51	0.10	0.48	0.11
Osaka B	-0.54	0.13	-0.47	0.12	-0.21	0.11	-0.07	0.12	-0.29	0.11	-0.22	0.11	0.06	0.13	-0.22	0.13	-0.18	0.12	0.10	0.12	0.25	0.12	0.31	0.10	0.33	0.10	0.85	0.12	0.43	0.12	0.56	0.13	0.56	0.13
Toyama A	-0.22	0.11	-0.28	0.11	-0.21	0.11	-0.21	0.10	-0.22	0.10	-0.25	0.10	-0.11	0.12	-0.19	0.12	0.03	0.10	0.07	0.11	0.03	0.11	0.28	0.11	0.30	0.10	0.43	0.12	0.27	0.11	0.20	0.11	0.26	0.12
Kanazawa Med	-0.17	0.13	-0.20	0.13	-0.10	0.12	-0.05	0.12	-0.21	0.11	-0.13	0.11	-0.17	0.13	-0.16	0.13	0.07	0.13	0.09	0.12	0.19	0.12	0.21	0.12	0.25	0.11	0.31	0.14	0.29	0.13	0.27	0.13	0.20	0.13
Kyoto B	-0.65	0.16	-0.48	0.16	-0.27	0.15	-0.18	0.15	-0.33	0.14	-0.37	0.12	0.11	0.17	-0.30	0.15	-0.10	0.14	-0.01	0.13	0.08	0.14	0.22	0.14	0.20	0.13	0.91	0.15	0.39	0.14	0.68	0.14	0.69	0.15
Kyoto A	-0.45	0.13	-0.53	0.12	0.06	0.11	-0.15	0.12	-0.23	0.11	-0.14	0.11	-0.26	0.14	-0.28	0.14	-0.28	0.12	0.07	0.12	0.15	0.11	0.19	0.12	0.30	0.12	0.72	0.13	0.39	0.12	0.40	0.13	0.40	0.13
Nagoya A	-0.61	0.15	-0.71	0.15	-0.28	0.15	-0.23	0.15	-0.32	0.14	-0.41	0.13	0.26	0.16	0.02	0.15	-0.21	0.16	-0.06	0.14	0.03	0.14	0.17	0.15	0.10	0.14	0.80	0.16	0.60	0.16	0.49	0.16	0.53	0.16
Hokkaido A	-1.04	0.18	-1.00	0.18	-0.45	0.17	-0.35	0.17	-0.16	0.17	0.02	0.15	-0.49	0.18	-0.58	0.18	-0.22	0.19	0.20	0.16	0.28	0.16	0.20	0.16	0.14	0.16	0.36	0.18	0.15	0.18	0.39	0.18	0.54	0.18
Tokyo B	-0.28	0.18	-0.34	0.17	-0.20	0.17	-0.01	0.17	-0.71	0.16	-0.56	0.15	-0.19	0.18	-0.32	0.17	-0.03	0.16	-0.03	0.18	0.03	0.18	-0.05	0.18	-0.15	0.16	0.59	0.17	0.28	0.16	0.39	0.18	0.32	0.18
Kyushu A	-0.65	0.17	-0.38	0.15	-0.31	0.17	-0.35	0.15	-0.30	0.14	-0.47	0.13	-0.29	0.19	-0.18	0.18	0.00	0.13	-0.10	0.16	-0.04	0.17	0.29	0.16	0.32	0.15	0.23	0.18	0.27	0.16	0.65	0.19	0.57	0.18
Toyama B	-0.34	0.16	-0.25	0.15	-0.03	0.15	0.34	0.16	-0.60	0.16	-0.57	0.13	-0.02	0.18	-0.24	0.17	-0.33	0.17	0.12	0.15	0.16	0.15	0.26	0.15	0.18	0.15	0.22	0.18	0.08	0.17	0.35	0.16	0.42	0.17
Yamaguchi A	-0.89	0.19	-0.84	0.19	-0.35	0.19	-0.32	0.20	-0.22	0.15	-0.14	0.15	-0.37	0.17	-0.31	0.17	0.20	0.18	0.11	0.19	0.03	0.20	0.31	0.18	0.30	0.17	0.23	0.20	0.17	0.20	0.47	0.19	0.51	0.19
Tokyo E	-0.57	0.24	-0.71	0.22	-0.18	0.26	-0.48	0.25	-0.19	0.21	-0.44	0.21	-0.31	0.25	-0.31	0.21	0.35	0.25	0.44	0.23	0.47	0.22	0.24	0.24	0.58	0.25	0.93	0.26	0.76	0.26	0.58	0.22	0.65	0.20
UOEH	-0.61	0.29	-0.68	0.26	-0.21	0.25	0.27	0.23	0.31	0.24	-0.31	0.23	0.08	0.27	-0.18	0.26	-0.07	0.24	0.89	0.28	1.12	0.27	0.82	0.27	0.54	0.25	1.44	0.27	0.76	0.28	0.89	0.28	0.52	0.27
Kyushu B	-1.01	0.24	-0.51	0.23	-0.44	0.20	-0.26	0.21	-0.46	0.17	-0.44	0.17	-0.31	0.24	-0.43	0.24	-0.15	0.21	-0.06	0.20	-0.12	0.21	-0.13	0.22	-0.12	0.20	0.45	0.22	0.11	0.18	0.60	0.20	0.54	0.20
Tokyo D	-0.50	0.30	-0.65	0.28	0.36	0.31	0.24	0.32	-0.46	0.32	-0.34	0.28	0.05	0.32	-0.50	0.31	-0.33	0.30	0.22	0.30	0.01	0.29	0.65	0.27	0.71	0.31	0.79	0.30	0.10	0.29	0.34	0.29	0.32	0.30
Tokyo C	-0.50	0.36	-0.43	0.34	-0.47	0.34	-0.22	0.33	-0.12	0.29	0.10	0.28	-0.16	0.34	-0.83	0.35	-0.58	0.27	0.38	0.26	0.27	0.28	0.27	0.32	0.05	0.31	0.45	0.33	0.33	0.33	0.99	0.29	0.95	0.29
Hokkaido B	-0.92	0.27	-1.33	0.27	0.14	0.28	-0.02	0.26	-0.30	0.19	-0.30	0.19	-0.03	0.33	0.28	0.31	-0.39	0.24	0.08	0.27	0.31	0.29	0.02	0.26	0.27	0.27	0.84	0.27	0.28	0.27	0.65	0.29	0.84	0.31
Tokushima B	-0.60	0.29	-0.56	0.27	-0.40	0.29	-0.35	0.30	-0.40	0.28	-0.45	0.30	0.43	0.33	0.52	0.29	0.09	0.27	0.09	0.26	0.11	0.29	0.09	0.23	0.33	0.21	0.49	0.25	0.35	0.31	0.52	0.24	0.65	0.23
Tokushima A	-0.56	0.27	-0.39	0.29	-0.26	0.26	-0.35	0.29	-0.85	0.27	-0.75	0.32	-0.08	0.32	-0.32	0.28	0.19	0.28	0.17	0.29	-0.11	0.32	0.15	0.19	0.29	0.20	1.07	0.21	0.56	0.25	0.47	0.31	-0.01	0.30
Nagoya B	-0.28	0.73	-0.31	0.73	-0.84	0.61	0.34	0.70	0.66	0.73	-0.96	0.60	0.05	0.71	0.55	0.73	1.75	0.57	-0.67	0.69	-0.81	0.69	-0.39	0.73	-0.40	0.72	-0.44	0.70	-0.56	0.70	-0.57	0.61	-0.34	0.61

Abbreviations: Lhippo, left hippocampus; Rhippo, right hippocampus; Lamyg, left amygdala; Ramyg, right amygdala; Lthal, left thalamus; Rthal, right thalamus; Laccumb, left accumbens; Raccumb, right accumbens; ICV, intracranial volume; Lcaud, left caudate; Rcaud, right caudate; Lput, left putamen; Rput, right putamen; Lpal, left pallidum; Rpal, right pallidum; LLatVent, left lateral ventricle; RLatVent, right lateral ventricle.

(b) Cohen's *d* effect sizes and SEs for group differences in regional volumes within each protocol in HC vs bipolar disorder (BP) analysis.

Protocol Name	Lhippo		Rhippo		Lamyg		Ramyg		Lthal		Rthal		Laccumb		Raccumb		ICV		Lcaud		Rcaud		Lput		Rput		Lpal		Rpal		LLatVent		RLatVent	
	d	SE	*d*	SE	*d*	SE	*d*	SE	*d*	SE	*d*	SE	*d*	SE	*d*	SE	*d*	SE	*d*	SE	*d*	SE	*d*	SE	*d*	SE	*d*	SE	*d*	SE	*d*	SE	*d*	SE
Osaka C	-0.47	0.42	-0.48	0.39	0.46	0.42	0.07	0.40	-0.41	0.35	-0.21	0.34	-0.54	0.40	-0.38	0.37	-0.26	0.42	0.38	0.37	0.05	0.38	0.02	0.34	-0.28	0.32	0.31	0.37	-0.01	0.35	0.45	0.41	0.29	0.40
Kanazawa Med	-0.24	0.18	-0.14	0.18	0.34	0.17	0.25	0.17	-0.07	0.17	0.03	0.16	-0.34	0.19	-0.10	0.18	0.00	0.19	0.11	0.17	0.09	0.17	0.13	0.16	0.26	0.14	0.10	0.19	0.14	0.18	0.30	0.18	0.11	0.18
Nagoya A	-0.33	0.23	-0.31	0.23	-0.06	0.24	-0.16	0.25	0.40	0.21	0.17	0.20	0.07	0.25	0.19	0.23	0.05	0.25	0.43	0.22	0.27	0.22	0.10	0.24	0.45	0.20	0.34	0.26	0.25	0.25	0.08	0.24	0.10	0.24
Yamaguchi B	0.16	0.22	0.09	0.23	0.32	0.22	0.36	0.23	0.14	0.18	-0.19	0.15	0.02	0.21	-0.23	0.20	0.10	0.24	-0.20	0.22	-0.17	0.22	-0.21	0.21	0.00	0.18	-0.20	0.25	-0.01	0.22	0.18	0.22	0.03	0.23
Hokkaido A	-0.79	0.16	-0.83	0.16	-0.46	0.16	-0.37	0.16	-0.19	0.15	0.02	0.14	-0.42	0.18	-0.43	0.17	-0.10	0.19	0.18	0.18	0.11	0.18	-0.07	0.16	0.05	0.16	0.10	0.19	-0.29	0.17	0.33	0.17	0.39	0.17
Yamaguchi A	-0.19	0.31	-0.18	0.31	-0.52	0.32	-0.46	0.33	-0.01	0.25	-0.08	0.25	0.00	0.29	-0.13	0.27	-0.12	0.30	0.24	0.29	0.32	0.31	-0.26	0.30	0.01	0.28	0.37	0.33	0.06	0.33	-0.08	0.31	-0.07	0.30
Kyushu A	-0.02	0.23	-0.29	0.21	-0.34	0.22	-0.23	0.20	-0.33	0.18	-0.41	0.18	-0.01	0.26	-0.51	0.27	0.03	0.19	0.01	0.22	0.03	0.23	-0.17	0.22	-0.20	0.19	-0.15	0.25	-0.11	0.22	0.50	0.25	0.62	0.25
Hiroshima D	-0.49	0.19	-0.41	0.19	0.00	0.21	0.05	0.21	-0.30	0.16	-0.21	0.14	-0.13	0.20	-0.13	0.19	0.17	0.21	0.05	0.21	0.04	0.20	-0.03	0.19	-0.02	0.18	0.36	0.20	0.05	0.18	0.32	0.18	0.38	0.19
Tokyo E	-0.23	0.23	-0.12	0.22	-0.03	0.27	-0.22	0.26	-0.05	0.24	-0.25	0.21	0.33	0.24	0.04	0.21	0.13	0.26	0.67	0.24	0.70	0.24	0.23	0.21	0.55	0.23	0.37	0.27	0.32	0.25	0.71	0.24	0.75	0.23
Kyushu B	-0.39	0.38	-0.67	0.37	-0.45	0.31	-0.50	0.34	-0.59	0.32	-0.42	0.32	-0.15	0.41	-0.29	0.42	-0.26	0.27	0.49	0.32	0.20	0.37	-0.10	0.39	-0.29	0.34	0.30	0.35	-0.44	0.28	0.92	0.36	1.03	0.34

Abbreviations: Lhippo, left hippocampus; Rhippo, right hippocampus; Lamyg, left amygdala; Ramyg, right amygdala; Lthal, left thalamus; Rthal, right thalamus; Laccumb, left accumbens; Raccumb, right accumbens; ICV, intracranial volume; Lcaud, left caudate; Rcaud, right caudate; Lput, left putamen; Rput, right putamen; Lpal, left pallidum; Rpal, right pallidum; LLatVent, left lateral ventricle; RLatVent, right lateral ventricle.

(c) Cohen's *d* effect sizes and SEs for group differences in regional volumes within each protocol in HC vs major depressive disorder (MDD) analysis.

Protocol Name	Lhippo		Rhippo		Lamyg		Ramyg		Lthal		Rthal		Laccumb		Raccumb		ICV		Lcaud		Rcaud		Lput		Rput		Lpal		Rpal		LLatVent		RLatVent	
	d	SE	*d*	SE	*d*	SE	*d*	SE	*d*	SE	*d*	SE	*d*	SE	*d*	SE	*d*	SE	*d*	SE	*d*	SE	*d*	SE	*d*	SE	*d*	SE	*d*	SE	*d*	SE	*d*	SE
Osaka C	-0.56	0.22	-0.63	0.20	-0.44	0.22	-0.27	0.21	-0.06	0.18	-0.21	0.18	-0.31	0.21	-0.24	0.19	0.04	0.22	-0.05	0.20	-0.21	0.20	-0.28	0.18	-0.27	0.17	-0.11	0.20	-0.08	0.18	0.71	0.21	0.56	0.21
Osaka A	-0.03	0.23	-0.16	0.22	-0.26	0.23	-0.17	0.22	-0.31	0.20	-0.43	0.19	0.20	0.23	-0.11	0.23	-0.23	0.22	-0.06	0.19	0.01	0.19	-0.45	0.20	-0.11	0.19	0.08	0.22	-0.31	0.21	0.12	0.23	0.15	0.23
Osaka B	-0.31	0.31	0.19	0.29	-0.01	0.28	0.13	0.29	-0.25	0.27	0.12	0.26	0.26	0.32	0.46	0.32	-0.36	0.29	0.29	0.29	0.57	0.30	0.65	0.26	0.46	0.24	0.74	0.30	0.46	0.29	0.24	0.31	0.35	0.31
Hokkaido A	-0.78	0.15	-0.87	0.15	-0.49	0.15	-0.42	0.15	-0.20	0.13	-0.09	0.12	-0.44	0.15	-0.50	0.15	-0.11	0.15	0.06	0.15	0.13	0.16	-0.11	0.14	-0.07	0.14	0.07	0.17	-0.31	0.16	0.38	0.15	0.38	0.15
Yamaguchi B	-0.04	0.14	-0.07	0.14	-0.09	0.14	0.11	0.14	0.18	0.11	-0.10	0.10	-0.33	0.13	-0.25	0.12	-0.02	0.15	0.01	0.14	0.03	0.14	-0.17	0.13	-0.08	0.11	-0.09	0.15	0.07	0.13	0.00	0.13	0.04	0.14
Kanazawa Med	-0.11	0.16	-0.11	0.16	0.15	0.15	-0.01	0.15	-0.04	0.15	-0.20	0.14	-0.34	0.17	-0.31	0.16	-0.06	0.17	-0.11	0.16	-0.08	0.16	-0.03	0.14	0.06	0.13	-0.06	0.17	0.04	0.17	0.14	0.15	0.09	0.15

Hiroshima A	-0.15	0.17	-0.06	0.17	-0.26	0.17	-0.08	0.17	0.09	0.14	-0.03	0.14	-0.10	0.16	-0.09	0.16	-0.29	0.16	-0.26	0.17	-0.22	0.17	-0.34	0.16	-0.28	0.14	-0.34	0.17	-0.31	0.18	0.05	0.17	0.00	0.16
Hiroshima D	-0.13	0.16	-0.17	0.16	-0.04	0.18	-0.01	0.16	-0.19	0.14	0.01	0.12	0.13	0.18	-0.20	0.18	-0.21	0.17	0.19	0.17	0.04	0.16	0.21	0.16	0.17	0.15	0.04	0.18	-0.22	0.17	0.16	0.18	0.19	0.18
Yamaguchi A	0.03	0.20	0.14	0.21	0.13	0.21	0.22	0.21	-0.11	0.17	-0.11	0.17	-0.39	0.19	-0.21	0.19	-0.18	0.20	0.01	0.19	0.13	0.20	-0.17	0.20	-0.18	0.19	-0.03	0.22	-0.11	0.23	0.07	0.21	0.05	0.20
Kyushu A	-1.06	0.29	-0.91	0.26	-0.21	0.28	-0.39	0.25	-0.62	0.23	-0.41	0.24	-0.34	0.35	-0.90	0.35	-0.40	0.24	-0.56	0.30	-0.62	0.32	-0.37	0.30	-0.64	0.26	-0.66	0.33	-0.29	0.29	0.76	0.35	0.51	0.35
Tokyo E	0.05	0.20	0.07	0.19	0.11	0.20	-0.03	0.19	0.02	0.20	0.10	0.20	0.34	0.20	0.29	0.19	0.32	0.21	0.34	0.20	0.40	0.19	0.34	0.20	0.33	0.20	0.34	0.21	0.32	0.20	0.42	0.20	0.33	0.20
UOEH	-0.29	0.24	-0.35	0.23	-0.16	0.22	-0.37	0.23	-0.19	0.19	-0.10	0.20	-0.56	0.24	-0.19	0.23	-0.03	0.21	-0.26	0.23	-0.19	0.23	-0.42	0.22	-0.39	0.22	-0.42	0.23	-0.50	0.23	0.40	0.23	0.34	0.24
Hiroshima B	-0.28	0.25	-0.19	0.25	-0.16	0.25	0.28	0.24	0.09	0.20	-0.17	0.22	0.06	0.27	-0.07	0.26	-0.14	0.26	0.15	0.26	0.18	0.26	0.09	0.25	0.01	0.23	-0.02	0.26	0.03	0.25	-0.15	0.23	-0.13	0.22
Hiroshima C	0.08	0.39	0.26	0.38	0.19	0.36	0.14	0.40	0.23	0.34	0.74	0.33	-0.20	0.36	0.62	0.38	-0.12	0.37	0.04	0.31	-0.15	0.36	0.53	0.32	0.30	0.30	0.17	0.35	0.09	0.39	0.78	0.39	0.79	0.37

Abbreviations: Lhippo, left hippocampus; Rhippo, right hippocampus; Lamyg, left amygdala; Ramyg, right amygdala; Lthal, left thalamus; Rthal, right thalamus; Laccumb, left accumbens; Raccumb, right accumbens; ICV, intracranial volume; Lcaud, left caudate; Rcaud, right caudate; Lput, left putamen; Rput, right putamen; Lpal, left pallidum; Rpal, right pallidum; LLatVent, left lateral ventricle; RLatVent, right lateral ventricle.

(d) Cohen's *d* effect sizes and SEs for group differences in regional volumes within each protocol in HC vs autism spectrum disorder (ASD) analysis.

Protocol Name	Lhippo		Rhippo		Lamyg		Ramyg		Lthal		Rthal		Laccumb		Raccumb		ICV		Lcaud		Rcaud		Lput		Rput		Lpal		Rpal		LLatVent		RLatVent	
	d	SE	*d*	SE	*d*	SE	*d*	SE	*d*	SE	*d*	SE	*d*	SE	*d*	SE	*d*	SE	*d*	SE	*d*	SE	*d*	SE	*d*	SE	*d*	SE	*d*	SE	*d*	SE	*d*	SE
Osaka A	-0.19	0.14	-0.21	0.13	-0.07	0.14	-0.11	0.14	-0.28	0.12	-0.44	0.11	-0.19	0.14	-0.28	0.14	-0.30	0.13	-0.07	0.12	-0.01	0.12	-0.04	0.12	-0.08	0.11	0.07	0.13	-0.14	0.12	0.61	0.14	0.56	0.14
Osaka C	0.28	0.22	-0.10	0.21	-0.02	0.22	-0.14	0.21	0.07	0.19	-0.26	0.18	0.09	0.21	-0.27	0.19	-0.01	0.22	0.26	0.20	0.12	0.20	0.04	0.18	0.06	0.17	-0.18	0.20	-0.35	0.18	0.20	0.22	0.07	0.21
Osaka B	-0.26	0.24	-0.33	0.23	-0.09	0.24	-0.08	0.24	-0.25	0.22	-0.22	0.21	-0.47	0.26	-0.34	0.26	0.14	0.23	-0.52	0.24	-0.15	0.25	-0.46	0.21	-0.44	0.19	-0.05	0.25	0.22	0.24	0.26	0.26	0.24	0.26
Showa	-0.09	0.15	-0.01	0.15	0.13	0.16	0.09	0.16	-0.15	0.15	-0.04	0.14	-0.04	0.17	0.02	0.15	0.13	0.15	0.05	0.14	0.12	0.14	0.02	0.13	0.08	0.13	-0.07	0.15	0.04	0.15	0.10	0.16	-0.02	0.16
Nagoya A	-0.05	0.27	-0.40	0.27	0.11	0.28	0.19	0.29	-0.06	0.25	0.23	0.24	-0.31	0.30	-0.36	0.27	-0.09	0.29	0.21	0.26	0.33	0.26	0.21	0.28	0.02	0.25	0.09	0.29	0.09	0.30	0.21	0.29	0.42	0.29
Tokyo B	-0.17	0.22	0.14	0.20	-0.21	0.20	0.07	0.20	-0.24	0.20	-0.08	0.19	-0.26	0.21	-0.23	0.21	-0.11	0.20	-0.02	0.21	-0.13	0.20	-0.05	0.21	-0.30	0.19	0.09	0.20	-0.25	0.19	0.16	0.20	0.03	0.21
Tokyo E	0.22	0.41	-0.34	0.41	0.11	0.48	0.16	0.44	0.18	0.37	-0.20	0.36	1.21	0.43	1.07	0.33	0.56	0.43	-0.31	0.41	-0.05	0.39	-0.02	0.38	0.67	0.39	0.25	0.47	-0.23	0.45	0.08	0.43	0.63	0.42

Abbreviations: Lhippo, left hippocampus; Rhippo, right hippocampus; Lamyg, left amygdala; Ramyg, right amygdala; Lthal, left thalamus; Rthal, right thalamus; Laccumb, left accumbens; Raccumb, right accumbens; ICV, intracranial volume; Lcaud, left caudate; Rcaud, right caudate; Lput, left putamen; Rput, right putamen; Lpal, left pallidum; Rpal, right pallidum; LLatVent, left lateral ventricle; RLatVent, right lateral ventricle.

Supplementary Table 6: Meta-analytic results for differences in each regional volume between healthy controls (HC) vs schizophrenia (SZ).

(a) Meta-analytic results for differences in left hippocampus volume between HC vs SZ.

```
Random-Effects Model (k = 24; tau^2 estimator: REML)

tau^2 (estimated amount of total heterogeneity): 0.0316 (SE = 0.0174)
tau (square root of estimated tau^2 value):      0.1777
I^2 (total heterogeneity / total variability):   58.86%
H^2 (total variability / sampling variability):  2.43

Test for Heterogeneity:
Q(df = 23) = 54.3419, p-val = 0.0002

Model Results:

estimate      se      zval    pval    ci.lb    ci.ub
 -0.5607  0.0515  -10.8887  <.0001  -0.6616  -0.4598  ***

---
Signif. codes:  0 '***' 0.001 '**' 0.01 '*' 0.05 '.' 0.1 ' ' 1
```

(b) Meta-analytic results for differences in right hippocampus volume between HC vs SZ.

```
Random-Effects Model (k = 24; tau^2 estimator: REML)

tau^2 (estimated amount of total heterogeneity): 0.0326 (SE = 0.0174)
tau (square root of estimated tau^2 value):      0.1806
I^2 (total heterogeneity / total variability):   61.06%
H^2 (total variability / sampling variability):  2.57

Test for Heterogeneity:
Q(df = 23) = 58.9574, p-val < .0001

Model Results:

estimate      se      zval    pval    ci.lb    ci.ub
 -0.5645  0.0513  -11.0137  <.0001  -0.6649  -0.4640  ***

---
Signif. codes:  0 '***' 0.001 '**' 0.01 '*' 0.05 '.' 0.1 ' ' 1
```

(c) Meta-analytic results for differences in left amygdala volume between HC vs SZ.

```
Random-Effects Model (k = 24; tau^2 estimator: REML)

tau^2 (estimated amount of total heterogeneity): 0.0044 (SE = 0.0067)
tau (square root of estimated tau^2 value):      0.0660
I^2 (total heterogeneity / total variability):   17.44%
H^2 (total variability / sampling variability):  1.21

Test for Heterogeneity:
```

```
Q(df = 23) = 25.9901, p-val = 0.3013

Model Results:

estimate      se     zval    pval    ci.lb    ci.ub
 -0.2413  0.0335  -7.2058  <.0001  -0.3069  -0.1757  ***

---
Signif. codes:  0 '***' 0.001 '**' 0.01 '*' 0.05 '.' 0.1 ' ' 1
```

(d) Meta-analytic results for differences in right amygdala volume between HC vs SZ.

```
Random-Effects Model (k = 24; tau^2 estimator: REML)

tau^2 (estimated amount of total heterogeneity): 0.0082 (SE = 0.0082)
tau (square root of estimated tau^2 value):      0.0904
I^2 (total heterogeneity / total variability):   28.70%
H^2 (total variability / sampling variability):  1.40

Test for Heterogeneity:
Q(df = 23) = 32.2605, p-val = 0.0948

Model Results:

estimate      se     zval    pval    ci.lb    ci.ub
 -0.1661  0.0366  -4.5399  <.0001  -0.2378  -0.0944  ***

---
Signif. codes:  0 '***' 0.001 '**' 0.01 '*' 0.05 '.' 0.1 ' ' 1
```

(e) Meta-analytic results for differences in left thalamus volume between HC vs SZ.

```
Random-Effects Model (k = 24; tau^2 estimator: REML)

tau^2 (estimated amount of total heterogeneity): 0.0096 (SE = 0.0078)
tau (square root of estimated tau^2 value):      0.0980
I^2 (total heterogeneity / total variability):   37.11%
H^2 (total variability / sampling variability):  1.59

Test for Heterogeneity:
Q(df = 23) = 40.4228, p-val = 0.0138

Model Results:

estimate      se     zval    pval    ci.lb    ci.ub
 -0.3122  0.0355  -8.7907  <.0001  -0.3818  -0.2426  ***

---
Signif. codes:  0 '***' 0.001 '**' 0.01 '*' 0.05 '.' 0.1 ' ' 1
```

(f) Meta-analytic results for differences in right thalamus volume between HC vs SZ.

```
Random-Effects Model (k = 24; tau^2 estimator: REML)
```

tau^2 (estimated amount of total heterogeneity): 0.0112 (SE = 0.0081)
tau (square root of estimated tau^2 value): 0.1059
I^2 (total heterogeneity / total variability): 42.72%
H^2 (total variability / sampling variability): 1.75

Test for Heterogeneity:
Q(df = 23) = 40.1387, p-val = 0.0148

Model Results:

```
estimate    se     zval     pval    ci.lb    ci.ub
-0.3192  0.0359  -8.8846  <.0001  -0.3896  -0.2488  ***

---
Signif. codes:  0 ‘***’ 0.001 ‘**’ 0.01 ‘*’ 0.05 ‘.’ 0.1 ‘ ’ 1
```

(g) Meta-analytic results for differences in left accumbens volume between HC vs SZ.

Random-Effects Model (k = 24; tau^2 estimator: REML)

tau^2 (estimated amount of total heterogeneity): 0.0161 (SE = 0.0124)
tau (square root of estimated tau^2 value): 0.1267
I^2 (total heterogeneity / total variability): 39.68%
H^2 (total variability / sampling variability): 1.66

Test for Heterogeneity:
Q(df = 23) = 35.6635, p-val = 0.0446

Model Results:

```
estimate    se     zval     pval    ci.lb    ci.ub
-0.1776  0.0444  -4.0003  <.0001  -0.2646  -0.0906  ***

---
Signif. codes:  0 ‘***’ 0.001 ‘**’ 0.01 ‘*’ 0.05 ‘.’ 0.1 ‘ ’ 1
```

(h) Meta-analytic results for differences in right accumbens volume between HC vs SZ.

Random-Effects Model (k = 24; tau^2 estimator: REML)

tau^2 (estimated amount of total heterogeneity): 0.0000 (SE = 0.0052)
tau (square root of estimated tau^2 value): 0.0007
I^2 (total heterogeneity / total variability): 0.00%
H^2 (total variability / sampling variability): 1.00

Test for Heterogeneity:
Q(df = 23) = 27.3518, p-val = 0.2413

Model Results:

```
estimate    se     zval     pval    ci.lb    ci.ub
```

```
 -0.2731  0.0305  -8.9579  <.0001  -0.3329  -0.2134  ***

---
Signif. codes:  0 '***' 0.001 '**' 0.01 '*' 0.05 '.' 0.1 ' ' 1
```

(i) Meta-analytic results for differences in intracranial volume between HC vs SZ.

```
Random-Effects Model (k = 24; tau^2 estimator: REML)

tau^2 (estimated amount of total heterogeneity): 0.0003 (SE = 0.0047)
tau (square root of estimated tau^2 value):      0.0170
I^2 (total heterogeneity / total variability):   1.42%
H^2 (total variability / sampling variability):  1.01

Test for Heterogeneity:
Q(df = 23) = 34.8770, p-val = 0.0535

Model Results:

estimate      se     zval    pval    ci.lb    ci.ub
 -0.0903  0.0289  -3.1260  0.0018  -0.1470  -0.0337  **

---
Signif. codes:  0 '***' 0.001 '**' 0.01 '*' 0.05 '.' 0.1 ' ' 1
```

(j) Meta-analytic results for differences in left caudate volume between HC vs SZ.

```
Random-Effects Model (k = 24; tau^2 estimator: REML)

tau^2 (estimated amount of total heterogeneity): 0.0190 (SE = 0.0121)
tau (square root of estimated tau^2 value):      0.1378
I^2 (total heterogeneity / total variability):   50.01%
H^2 (total variability / sampling variability):  2.00

Test for Heterogeneity:
Q(df = 23) = 48.9380, p-val = 0.0013

Model Results:

estimate      se    zval    pval   ci.lb   ci.ub
  0.1147  0.0434  2.6449  0.0082  0.0297  0.1997  **

---
Signif. codes:  0 '***' 0.001 '**' 0.01 '*' 0.05 '.' 0.1 ' ' 1
```

(k) Meta-analytic results for differences in right caudate volume between HC vs SZ.

```
Random-Effects Model (k = 24; tau^2 estimator: REML)

tau^2 (estimated amount of total heterogeneity): 0.0196 (SE = 0.0125)
tau (square root of estimated tau^2 value):      0.1401
I^2 (total heterogeneity / total variability):   50.15%
H^2 (total variability / sampling variability):  2.01
```

Test for Heterogeneity:
Q(df = 23) = 49.4124, p-val = 0.0011

Model Results:

```
estimate     se    zval    pval   ci.lb   ci.ub
  0.1553  0.0441  3.5190  0.0004  0.0688  0.2418  ***

---
Signif. codes:  0 ‘***’ 0.001 ‘**’ 0.01 ‘*’ 0.05 ‘.’ 0.1 ‘ ’ 1
```

(l) Meta-analytic results for differences in left putamen volume between HC vs SZ.

```
Random-Effects Model (k = 24; tau^2 estimator: REML)

tau^2 (estimated amount of total heterogeneity): 0.0021 (SE = 0.0052)
tau (square root of estimated tau^2 value):      0.0463
I^2 (total heterogeneity / total variability):   10.29%
H^2 (total variability / sampling variability):  1.11

Test for Heterogeneity:
Q(df = 23) = 24.1315, p-val = 0.3966

Model Results:

estimate     se    zval    pval   ci.lb   ci.ub
  0.2286  0.0300  7.6113  <.0001  0.1697  0.2875  ***

---
Signif. codes:  0 ‘***’ 0.001 ‘**’ 0.01 ‘*’ 0.05 ‘.’ 0.1 ‘ ’ 1
```

(m) Meta-analytic results for differences in right putamen volume between HC vs SZ.

```
Random-Effects Model (k = 24; tau^2 estimator: REML)

tau^2 (estimated amount of total heterogeneity): 0.0111 (SE = 0.0086)
tau (square root of estimated tau^2 value):      0.1052
I^2 (total heterogeneity / total variability):   39.24%
H^2 (total variability / sampling variability):  1.65

Test for Heterogeneity:
Q(df = 23) = 38.0811, p-val = 0.0250

Model Results:

estimate     se    zval    pval   ci.lb   ci.ub
  0.2468  0.0370  6.6655  <.0001  0.1742  0.3194  ***

---
Signif. codes:  0 ‘***’ 0.001 ‘**’ 0.01 ‘*’ 0.05 ‘.’ 0.1 ‘ ’ 1
```

(n) Meta-analytic results for differences in left pallidum volume between HC vs SZ.

```
Random-Effects Model (k = 24; tau^2 estimator: REML)

tau^2 (estimated amount of total heterogeneity): 0.0580 (SE = 0.0262)
tau (square root of estimated tau^2 value):      0.2409
I^2 (total heterogeneity / total variability):   72.67%
H^2 (total variability / sampling variability):  3.66

Test for Heterogeneity:
Q(df = 23) = 81.0112, p-val < .0001

Model Results:

estimate      se     zval     pval    ci.lb    ci.ub
  0.5984  0.0621  9.6385  <.0001  0.4767  0.7201  ***

---
Signif. codes:  0 '***' 0.001 '**' 0.01 '*' 0.05 '.' 0.1 ' ' 1
```

(o) Meta-analytic results for differences in right pallidum volume between HC vs SZ.

```
Random-Effects Model (k = 24; tau^2 estimator: REML)

tau^2 (estimated amount of total heterogeneity): 0.0069 (SE = 0.0078)
tau (square root of estimated tau^2 value):      0.0830
I^2 (total heterogeneity / total variability):   24.88%
H^2 (total variability / sampling variability):  1.33

Test for Heterogeneity:
Q(df = 23) = 29.6168, p-val = 0.1607

Model Results:

estimate      se     zval     pval    ci.lb    ci.ub
  0.3264  0.0359  9.0816  <.0001  0.2559  0.3968  ***

---
Signif. codes:  0 '***' 0.001 '**' 0.01 '*' 0.05 '.' 0.1 ' ' 1
```

(p) Meta-analytic results for differences in left lateral ventricle volume between HC vs SZ.

```
Random-Effects Model (k = 24; tau^2 estimator: REML)

tau^2 (estimated amount of total heterogeneity): 0.0110 (SE = 0.0099)
tau (square root of estimated tau^2 value):      0.1047
I^2 (total heterogeneity / total variability):   32.82%
H^2 (total variability / sampling variability):  1.49

Test for Heterogeneity:
Q(df = 23) = 33.4026, p-val = 0.0743

Model Results:
```

estimate se zval pval ci.lb ci.ub
0.5100 0.0397 12.8444 <.0001 0.4322 0.5878 ***

Signif. codes: 0 ‘***’ 0.001 ‘**’ 0.01 ‘*’ 0.05 ‘.’ 0.1 ‘ ’ 1

(q) Meta-analytic results for differences in right lateral ventricle volume between HC vs SZ.

Random-Effects Model (k = 24; tau^2 estimator: REML)

tau^2 (estimated amount of total heterogeneity): 0.0083 (SE = 0.0088)
tau (square root of estimated tau^2 value): 0.0910
I^2 (total heterogeneity / total variability): 26.92%
H^2 (total variability / sampling variability): 1.37

Test for Heterogeneity:
Q(df = 23) = 30.6497, p-val = 0.1316

Model Results:

estimate se zval pval ci.lb ci.ub
0.5007 0.0378 13.2602 <.0001 0.4267 0.5747 ***

Signif. codes: 0 ‘***’ 0.001 ‘**’ 0.01 ‘*’ 0.05 ‘.’ 0.1 ‘ ’ 1

Supplementary Table 7: Meta-analytic results for differences in each regional volume between healthy controls (HC) vs bipolar disorder (BP).

(a) Meta-analytic results for differences in left hippocampus volume between HC vs BP.

```
Random-Effects Model (k = 10; tau^2 estimator: REML)

tau^2 (estimated amount of total heterogeneity): 0.0478 (SE = 0.0478)
tau (square root of estimated tau^2 value):      0.2185
I^2 (total heterogeneity / total variability):   48.31%
H^2 (total variability / sampling variability):  1.93

Test for Heterogeneity:
Q(df = 9) = 17.1328, p-val = 0.0467

Model Results:

estimate      se     zval    pval    ci.lb    ci.ub
 -0.3065  0.1023  -2.9976  0.0027  -0.5070  -0.1061  **

---
Signif. codes:  0 '***' 0.001 '**' 0.01 '*' 0.05 '.' 0.1 ' ' 1
```

(b) Meta-analytic results for differences in right hippocampus volume between HC vs BP.

```
Random-Effects Model (k = 10; tau^2 estimator: REML)

tau^2 (estimated amount of total heterogeneity): 0.0425 (SE = 0.0450)
tau (square root of estimated tau^2 value):      0.2061
I^2 (total heterogeneity / total variability):   45.28%
H^2 (total variability / sampling variability):  1.83

Test for Heterogeneity:
Q(df = 9) = 16.1784, p-val = 0.0632

Model Results:

estimate      se     zval    pval    ci.lb    ci.ub
 -0.3283  0.0992  -3.3099  0.0009  -0.5227  -0.1339  ***

---
Signif. codes:  0 '***' 0.001 '**' 0.01 '*' 0.05 '.' 0.1 ' ' 1
```

(c) Meta-analytic results for differences in left amygdala volume between HC vs BP.

```
andom-Effects Model (k = 10; tau^2 estimator: REML)

tau^2 (estimated amount of total heterogeneity): 0.0664 (SE = 0.0583)
tau (square root of estimated tau^2 value):      0.2577
I^2 (total heterogeneity / total variability):   55.49%
H^2 (total variability / sampling variability):  2.25

Test for Heterogeneity:
Q(df = 9) = 21.2951, p-val = 0.0114
```

Model Results:

```
estimate     se    zval    pval    ci.lb   ci.ub
 -0.0779  0.1124  -0.6932  0.4882  -0.2981  0.1424

---
Signif. codes:  0 ‘***’ 0.001 ‘**’ 0.01 ‘*’ 0.05 ‘.’ 0.1 ‘ ’ 1
```

(d) Meta-analytic results for differences in right amygdala volume between HC vs BP.

```
Random-Effects Model (k = 10; tau^2 estimator: REML)

tau^2 (estimated amount of total heterogeneity): 0.0375 (SE = 0.0433)
tau (square root of estimated tau^2 value):      0.1936
I^2 (total heterogeneity / total variability):   41.42%
H^2 (total variability / sampling variability):  1.71

Test for Heterogeneity:
Q(df = 9) = 15.0210, p-val = 0.0904

Model Results:

estimate     se    zval    pval    ci.lb   ci.ub
 -0.0973  0.0975  -0.9982  0.3182  -0.2883  0.0937

---
Signif. codes:  0 ‘***’ 0.001 ‘**’ 0.01 ‘*’ 0.05 ‘.’ 0.1 ‘ ’ 1
```

(e) Meta-analytic results for differences in left thalamus volume between HC vs BP.

```
Random-Effects Model (k = 10; tau^2 estimator: REML)

tau^2 (estimated amount of total heterogeneity): 0.0217 (SE = 0.0290)
tau (square root of estimated tau^2 value):      0.1474
I^2 (total heterogeneity / total variability):   35.28%
H^2 (total variability / sampling variability):  1.55

Test for Heterogeneity:
Q(df = 9) = 14.1796, p-val = 0.1161

Model Results:

estimate     se    zval    pval    ci.lb   ci.ub
 -0.1143  0.0801  -1.4280  0.1533  -0.2713  0.0426

---
Signif. codes:  0 ‘***’ 0.001 ‘**’ 0.01 ‘*’ 0.05 ‘.’ 0.1 ‘ ’ 1
```

(f) Meta-analytic results for differences in right thalamus volume between HC vs BP.

```
Random-Effects Model (k = 10; tau^2 estimator: REML)

tau^2 (estimated amount of total heterogeneity): 0.0001 (SE = 0.0148)
tau (square root of estimated tau^2 value):      0.0075
I^2 (total heterogeneity / total variability):   0.16%
H^2 (total variability / sampling variability):  1.00
```

Test for Heterogeneity:
Q(df = 9) = 8.3826, p-val = 0.4961

Model Results:

```
estimate     se    zval    pval    ci.lb    ci.ub
 -0.1299  0.0583  -2.2300  0.0257  -0.2442  -0.0157  *

---
Signif. codes:  0 ‘***’ 0.001 ‘**’ 0.01 ‘*’ 0.05 ‘.’ 0.1 ‘ ’ 1
```

(g) Meta-analytic results for differences in left accumbens volume between HC vs BP.

Random-Effects Model (k = 10; tau^2 estimator: REML)

tau^2 (estimated amount of total heterogeneity): 0.0146 (SE = 0.0329)
tau (square root of estimated tau^2 value): 0.1209
I^2 (total heterogeneity / total variability): 20.30%
H^2 (total variability / sampling variability): 1.25

Test for Heterogeneity:
Q(df = 9) = 10.1711, p-val = 0.3368

Model Results:

```
estimate     se    zval    pval    ci.lb   ci.ub
 -0.1240  0.0856  -1.4480  0.1476  -0.2918  0.0438

---
Signif. codes:  0 ‘***’ 0.001 ‘**’ 0.01 ‘*’ 0.05 ‘.’ 0.1 ‘ ’ 1
```

(h) Meta-analytic results for differences in right accumbens volume between HC vs BP.

Random-Effects Model (k = 10; tau^2 estimator: REML)

tau^2 (estimated amount of total heterogeneity): 0.0021 (SE = 0.0232)
tau (square root of estimated tau^2 value): 0.0459
I^2 (total heterogeneity / total variability): 3.95%
H^2 (total variability / sampling variability): 1.04

Test for Heterogeneity:
Q(df = 9) = 8.1944, p-val = 0.5147

Model Results:

```
estimate     se    zval    pval    ci.lb    ci.ub
 -0.1811  0.0725  -2.4965  0.0125  -0.3232  -0.0389  *

---
Signif. codes:  0 ‘***’ 0.001 ‘**’ 0.01 ‘*’ 0.05 ‘.’ 0.1 ‘ ’ 1
```

(i) Meta-analytic results for differences in intracranial volume between HC vs BP.

andom-Effects Model (k = 10; tau^2 estimator: REML)

tau^2 (estimated amount of total heterogeneity): 0 (SE = 0.0242)
tau (square root of estimated tau^2 value): 0
I^2 (total heterogeneity / total variability): 0.00%
H^2 (total variability / sampling variability): 1.00

Test for Heterogeneity:
Q(df = 9) = 2.8900, p-val = 0.9685

Model Results:

estimate	se	zval	pval	ci.lb	ci.ub
0.0004	0.0734	0.0055	0.9956	-0.1435	0.1443

Signif. codes: 0 ‘***’ 0.001 ‘**’ 0.01 ‘*’ 0.05 ‘.’ 0.1 ‘ ’ 1

(j) Meta-analytic results for differences in left caudate volume between HC vs BP.

Random-Effects Model (k = 10; tau^2 estimator: REML)

tau^2 (estimated amount of total heterogeneity): 0.0090 (SE = 0.0278)
tau (square root of estimated tau^2 value): 0.0946
I^2 (total heterogeneity / total variability): 14.63%
H^2 (total variability / sampling variability): 1.17

Test for Heterogeneity:
Q(df = 9) = 10.7556, p-val = 0.2928

Model Results:

estimate	se	zval	pval	ci.lb	ci.ub	
0.1957	0.0784	2.4959	0.0126	0.0420	0.3494	*

Signif. codes: 0 ‘***’ 0.001 ‘**’ 0.01 ‘*’ 0.05 ‘.’ 0.1 ‘ ’ 1

(k) Meta-analytic results for differences in right caudate volume between HC vs BP.

Random-Effects Model (k = 10; tau^2 estimator: REML)

tau^2 (estimated amount of total heterogeneity): 0 (SE = 0.0228)
tau (square root of estimated tau^2 value): 0
I^2 (total heterogeneity / total variability): 0.00%
H^2 (total variability / sampling variability): 1.00

Test for Heterogeneity:
Q(df = 9) = 8.9388, p-val = 0.4429

Model Results:

estimate	se	zval	pval	ci.lb	ci.ub	
0.1437	0.0719	1.9983	0.0457	0.0028	0.2847	*

Signif. codes: 0 ‘***’ 0.001 ‘**’ 0.01 ‘*’ 0.05 ‘.’ 0.1 ‘ ’ 1

(l) Meta-analytic results for differences in left putamen volume between HC vs BP.

```
Random-Effects Model (k = 10; tau^2 estimator: REML)

tau^2 (estimated amount of total heterogeneity): 0 (SE = 0.0199)
tau (square root of estimated tau^2 value):      0
I^2 (total heterogeneity / total variability):   0.00%
H^2 (total variability / sampling variability):  1.00

Test for Heterogeneity:
Q(df = 9) = 4.6824, p-val = 0.8611

Model Results:

estimate      se     zval    pval    ci.lb   ci.ub
 -0.0143  0.0675  -0.2116  0.8324  -0.1467  0.1181

---
Signif. codes:  0 '***' 0.001 '**' 0.01 '*' 0.05 '.' 0.1 ' ' 1
```

(m) Meta-analytic results for differences in right putamen volume between HC vs BP.

```
Random-Effects Model (k = 10; tau^2 estimator: REML)

tau^2 (estimated amount of total heterogeneity): 0.0199 (SE = 0.0283)
tau (square root of estimated tau^2 value):      0.1411
I^2 (total heterogeneity / total variability):   33.07%
H^2 (total variability / sampling variability):  1.49

Test for Heterogeneity:
Q(df = 9) = 13.9817, p-val = 0.1230

Model Results:

estimate      se    zval    pval    ci.lb   ci.ub
  0.0913  0.0792  1.1523  0.2492  -0.0640  0.2465

---
Signif. codes:  0 '***' 0.001 '**' 0.01 '*' 0.05 '.' 0.1 ' ' 1
```

(n) Meta-analytic results for differences in left pallidum volume between HC vs BP.

```
Random-Effects Model (k = 10; tau^2 estimator: REML)

tau^2 (estimated amount of total heterogeneity): 0 (SE = 0.0267)
tau (square root of estimated tau^2 value):      0
I^2 (total heterogeneity / total variability):   0.00%
H^2 (total variability / sampling variability):  1.00

Test for Heterogeneity:
Q(df = 9) = 6.6541, p-val = 0.6731

Model Results:

estimate      se    zval    pval   ci.lb   ci.ub
```

0.1646 0.0776 2.1223 0.0338 0.0126 0.3167 *

Signif. codes: 0 ‘***’ 0.001 ‘**’ 0.01 ‘*’ 0.05 ‘.’ 0.1 ‘ ’ 1

(o) Meta-analytic results for differences in right pallidum volume between HC vs BP.

Random-Effects Model (k = 10; tau^2 estimator: REML)

tau^2 (estimated amount of total heterogeneity): 0.0037 (SE = 0.0247)
tau (square root of estimated tau^2 value):   0.0610
I^2 (total heterogeneity / total variability):   6.70%
H^2 (total variability / sampling variability): 1.07

Test for Heterogeneity:
Q(df = 9) = 8.7912, p-val = 0.4568

Model Results:

estimate   se   zval   pval   ci.lb   ci.ub
-0.0136 0.0742 -0.1827 0.8550 -0.1591 0.1319

Signif. codes: 0 ‘***’ 0.001 ‘**’ 0.01 ‘*’ 0.05 ‘.’ 0.1 ‘ ’ 1

(p) Meta-analytic results for differences in left lateral ventricle volume between HC vs BP.

Random-Effects Model (k = 10; tau^2 estimator: REML)

tau^2 (estimated amount of total heterogeneity): 0.0000 (SE = 0.0228)
tau (square root of estimated tau^2 value):   0.0009
I^2 (total heterogeneity / total variability):   0.00%
H^2 (total variability / sampling variability): 1.00

Test for Heterogeneity:
Q(df = 9) = 9.0660, p-val = 0.4312

Model Results:

estimate   se   zval   pval   ci.lb   ci.ub
0.3379 0.0722 4.6794 <.0001 0.1964 0.4795 ***

Signif. codes: 0 ‘***’ 0.001 ‘**’ 0.01 ‘*’ 0.05 ‘.’ 0.1 ‘ ’ 1

(q) Meta-analytic results for differences in right lateral ventricle volume between HC vs BP.

Random-Effects Model (k = 10; tau^2 estimator: REML)

tau^2 (estimated amount of total heterogeneity): 0.0311 (SE = 0.0400)
tau (square root of estimated tau^2 value):   0.1764
I^2 (total heterogeneity / total variability):   36.85%
H^2 (total variability / sampling variability): 1.58

Test for Heterogeneity:
Q(df = 9) = 14.9574, p-val = 0.0921

```
Model Results:

estimate      se     zval     pval    ci.lb    ci.ub
  0.3419  0.0937  3.6500  0.0003  0.1583  0.5255  ***

---
Signif. codes:  0 ‘***’ 0.001 ‘**’ 0.01 ‘*’ 0.05 ‘.’ 0.1 ‘ ’ 1
```

Supplementary Table 8: Meta-analytic results for differences in each regional volume between healthy controls (HC) vs major depressive disorder (MDD).

(a) Meta-analytic results for differences in left hippocampus volume between HC vs MDD.

```
Random-Effects Model (k = 14; tau^2 estimator: REML)

tau^2 (estimated amount of total heterogeneity): 0.0578 (SE = 0.0396)
tau (square root of estimated tau^2 value):      0.2403
I^2 (total heterogeneity / total variability):   59.47%
H^2 (total variability / sampling variability):  2.47

Test for Heterogeneity:
Q(df = 13) = 32.8609, p-val = 0.0018

Model Results:

estimate     se    zval    pval    ci.lb    ci.ub
 -0.2493  0.0859  -2.9018  0.0037  -0.4178  -0.0809  **

---
Signif. codes:  0 ‘***’ 0.001 ‘**’ 0.01 ‘*’ 0.05 ‘.’ 0.1 ‘ ’ 1
```

(b) Meta-analytic results for differences in right hippocampus volume between HC vs MDD.

```
Random-Effects Model (k = 14; tau^2 estimator: REML)

tau^2 (estimated amount of total heterogeneity): 0.0827 (SE = 0.0493)
tau (square root of estimated tau^2 value):      0.2876
I^2 (total heterogeneity / total variability):   68.27%
H^2 (total variability / sampling variability):  3.15

Test for Heterogeneity:
Q(df = 13) = 42.3923, p-val < .0001

Model Results:

estimate     se    zval    pval    ci.lb    ci.ub
 -0.2228  0.0954  -2.3346  0.0196  -0.4099  -0.0358  *

---
Signif. codes:  0 ‘***’ 0.001 ‘**’ 0.01 ‘*’ 0.05 ‘.’ 0.1 ‘ ’ 1
```

(c) Meta-analytic results for differences in left amygdala volume between HC vs MDD.

```
Random-Effects Model (k = 14; tau^2 estimator: REML)

tau^2 (estimated amount of total heterogeneity): 0.0167 (SE = 0.0217)
tau (square root of estimated tau^2 value):      0.1292
I^2 (total heterogeneity / total variability):   30.03%
H^2 (total variability / sampling variability):  1.43

Test for Heterogeneity:
Q(df = 13) = 16.7836, p-val = 0.2094
```

Model Results:

estimate se zval pval ci.lb ci.ub
-0.1224 0.0643 -1.9036 0.0570 -0.2484 0.0036 .

Signif. codes: 0 ‘***’ 0.001 ‘**’ 0.01 ‘*’ 0.05 ‘.’ 0.1 ‘ ’ 1

(d) Meta-analytic results for differences in right amygdala volume between HC vs MDD.

Random-Effects Model (k = 14; tau^2 estimator: REML)

tau^2 (estimated amount of total heterogeneity): 0.0133 (SE = 0.0196)
tau (square root of estimated tau^2 value): 0.1155
I^2 (total heterogeneity / total variability): 26.20%
H^2 (total variability / sampling variability): 1.35

Test for Heterogeneity:
Q(df = 13) = 16.9051, p-val = 0.2037

Model Results:

estimate se zval pval ci.lb ci.ub
-0.0740 0.0613 -1.2074 0.2273 -0.1942 0.0461

Signif. codes: 0 ‘***’ 0.001 ‘**’ 0.01 ‘*’ 0.05 ‘.’ 0.1 ‘ ’ 1

(e) Meta-analytic results for differences in left thalamus volume between HC vs MDD.

Random-Effects Model (k = 14; tau^2 estimator: REML)

tau^2 (estimated amount of total heterogeneity): 0.0111 (SE = 0.0156)
tau (square root of estimated tau^2 value): 0.1054
I^2 (total heterogeneity / total variability): 27.55%
H^2 (total variability / sampling variability): 1.38

Test for Heterogeneity:
Q(df = 13) = 17.7768, p-val = 0.1662

Model Results:

estimate se zval pval ci.lb ci.ub
-0.0787 0.0547 -1.4385 0.1503 -0.1859 0.0285

Signif. codes: 0 ‘***’ 0.001 ‘**’ 0.01 ‘*’ 0.05 ‘.’ 0.1 ‘ ’ 1

(f) Meta-analytic results for differences in right thalamus volume between HC vs MDD.

Random-Effects Model (k = 14; tau^2 estimator: REML)

tau^2 (estimated amount of total heterogeneity): 0.0000 (SE = 0.0089)
tau (square root of estimated tau^2 value): 0.0026
I^2 (total heterogeneity / total variability): 0.03%

```
H^2 (total variability / sampling variability):  1.00

Test for Heterogeneity:
Q(df = 13) = 15.1505, p-val = 0.2981

Model Results:

estimate     se    zval    pval    ci.lb    ci.ub
 -0.0941  0.0428  -2.1992  0.0279  -0.1780  -0.0102  *

---
Signif. codes:  0 ‘***’ 0.001 ‘**’ 0.01 ‘*’ 0.05 ‘.’ 0.1 ‘ ’ 1
```

(g) Meta-analytic results for differences in left accumbens volume between HC vs MDD.

```
Random-Effects Model (k = 14; tau^2 estimator: REML)

tau^2 (estimated amount of total heterogeneity): 0.0388 (SE = 0.0319)
tau (square root of estimated tau^2 value):      0.1970
I^2 (total heterogeneity / total variability):   49.13%
H^2 (total variability / sampling variability):  1.97

Test for Heterogeneity:
Q(df = 13) = 24.7166, p-val = 0.0251

Model Results:

estimate     se    zval    pval    ci.lb    ci.ub
 -0.1619  0.0776  -2.0862  0.0370  -0.3141  -0.0098  *

---
Signif. codes:  0 ‘***’ 0.001 ‘**’ 0.01 ‘*’ 0.05 ‘.’ 0.1 ‘ ’ 1
```

(h) Meta-analytic results for differences in right accumbens volume between HC vs MDD.

```
Random-Effects Model (k = 14; tau^2 estimator: REML)

tau^2 (estimated amount of total heterogeneity): 0.0251 (SE = 0.0252)
tau (square root of estimated tau^2 value):      0.1584
I^2 (total heterogeneity / total variability):   39.60%
H^2 (total variability / sampling variability):  1.66

Test for Heterogeneity:
Q(df = 13) = 24.7563, p-val = 0.0248

Model Results:

estimate     se    zval    pval    ci.lb    ci.ub
 -0.1647  0.0694  -2.3740  0.0176  -0.3007  -0.0287  *

---
Signif. codes:  0 ‘***’ 0.001 ‘**’ 0.01 ‘*’ 0.05 ‘.’ 0.1 ‘ ’ 1
```

(i) Meta-analytic results for differences in intracranial volume between HC vs MDD.

```
Random-Effects Model (k = 14; tau^2 estimator: REML)
```

```
tau^2 (estimated amount of total heterogeneity): 0 (SE = 0.0143)
tau (square root of estimated tau^2 value):      0
I^2 (total heterogeneity / total variability):   0.00%
H^2 (total variability / sampling variability):  1.00

Test for Heterogeneity:
Q(df = 13) = 9.7072, p-val = 0.7177

Model Results:

estimate     se     zval    pval    ci.lb    ci.ub
 -0.1188  0.0529  -2.2459  0.0247  -0.2224  -0.0151  *

---
Signif. codes:  0 ‘***’ 0.001 ‘**’ 0.01 ‘*’ 0.05 ‘.’ 0.1 ‘ ’ 1
```

(j) Meta-analytic results for differences in left caudate volume between HC vs MDD.

```
Random-Effects Model (k = 14; tau^2 estimator: REML)

tau^2 (estimated amount of total heterogeneity): 0.0000 (SE = 0.0134)
tau (square root of estimated tau^2 value):      0.0026
I^2 (total heterogeneity / total variability):   0.02%
H^2 (total variability / sampling variability):  1.00

Test for Heterogeneity:
Q(df = 13) = 13.3689, p-val = 0.4197

Model Results:

estimate     se     zval    pval    ci.lb    ci.ub
 -0.0078  0.0514  -0.1519  0.8792  -0.1085  0.0929

---
Signif. codes:  0 ‘***’ 0.001 ‘**’ 0.01 ‘*’ 0.05 ‘.’ 0.1 ‘ ’ 1
```

(k) Meta-analytic results for differences in right caudate volume between HC vs MDD.

```
Random-Effects Model (k = 14; tau^2 estimator: REML)

tau^2 (estimated amount of total heterogeneity): 0.0021 (SE = 0.0144)
tau (square root of estimated tau^2 value):      0.0458
I^2 (total heterogeneity / total variability):   5.25%
H^2 (total variability / sampling variability):  1.06

Test for Heterogeneity:
Q(df = 13) = 17.3549, p-val = 0.1836

Model Results:

estimate     se    zval    pval    ci.lb    ci.ub
  0.0141  0.0533  0.2635  0.7921  -0.0904  0.1185

---
```

Signif. codes: 0 ‘***’ 0.001 ‘**’ 0.01 ‘*’ 0.05 ‘.’ 0.1 ‘ ’ 1

(l) Meta-analytic results for differences in left putamen volume between HC vs MDD.

```
Random-Effects Model (k = 14; tau^2 estimator: REML)

tau^2 (estimated amount of total heterogeneity): 0.0526 (SE = 0.0354)
tau (square root of estimated tau^2 value):      0.2294
I^2 (total heterogeneity / total variability):   60.63%
H^2 (total variability / sampling variability):  2.54

Test for Heterogeneity:
Q(df = 13) = 31.1923, p-val = 0.0032

Model Results:

estimate     se    zval    pval    ci.lb   ci.ub
 -0.0614  0.0811  -0.7572  0.4489  -0.2203  0.0975

---
Signif. codes:  0 ‘***’ 0.001 ‘**’ 0.01 ‘*’ 0.05 ‘.’ 0.1 ‘ ’ 1
```

(m) Meta-analytic results for differences in right putamen volume between HC vs MDD.

```
Random-Effects Model (k = 14; tau^2 estimator: REML)

tau^2 (estimated amount of total heterogeneity): 0.0229 (SE = 0.0211)
tau (square root of estimated tau^2 value):      0.1512
I^2 (total heterogeneity / total variability):   43.60%
H^2 (total variability / sampling variability):  1.77

Test for Heterogeneity:
Q(df = 13) = 24.4599, p-val = 0.0272

Model Results:

estimate     se    zval    pval    ci.lb   ci.ub
 -0.0577  0.0631  -0.9141  0.3606  -0.1814  0.0660

---
Signif. codes:  0 ‘***’ 0.001 ‘**’ 0.01 ‘*’ 0.05 ‘.’ 0.1 ‘ ’ 1
```

(n) Meta-analytic results for differences in left pallidum volume between HC vs MDD.

```
Random-Effects Model (k = 14; tau^2 estimator: REML)

tau^2 (estimated amount of total heterogeneity): 0.0140 (SE = 0.0221)
tau (square root of estimated tau^2 value):      0.1184
I^2 (total heterogeneity / total variability):   24.42%
H^2 (total variability / sampling variability):  1.32

Test for Heterogeneity:
Q(df = 13) = 20.5208, p-val = 0.0830

Model Results:
```

```
estimate     se    zval    pval    ci.lb   ci.ub
 -0.0318  0.0649  -0.4901  0.6241  -0.1589  0.0953

---
Signif. codes:  0 '***' 0.001 '**' 0.01 '*' 0.05 '.' 0.1 ' ' 1
```

(o) Meta-analytic results for differences in right pallidum volume between HC vs MDD.

```
Random-Effects Model (k = 14; tau^2 estimator: REML)

tau^2 (estimated amount of total heterogeneity): 0.0172 (SE = 0.0220)
tau (square root of estimated tau^2 value):      0.1312
I^2 (total heterogeneity / total variability):   30.38%
H^2 (total variability / sampling variability):  1.44

Test for Heterogeneity:
Q(df = 13) = 19.0181, p-val = 0.1225

Model Results:

estimate     se    zval    pval    ci.lb   ci.ub
 -0.0980  0.0649  -1.5099  0.1311  -0.2251  0.0292

---
Signif. codes:  0 '***' 0.001 '**' 0.01 '*' 0.05 '.' 0.1 ' ' 1
```

(p) Meta-analytic results for differences in left lateral ventricle volume between HC vs MDD.

```
Random-Effects Model (k = 14; tau^2 estimator: REML)

tau^2 (estimated amount of total heterogeneity): 0.0187 (SE = 0.0226)
tau (square root of estimated tau^2 value):      0.1368
I^2 (total heterogeneity / total variability):   32.39%
H^2 (total variability / sampling variability):  1.48

Test for Heterogeneity:
Q(df = 13) = 20.0964, p-val = 0.0928

Model Results:

estimate     se   zval    pval   ci.lb   ci.ub
  0.2295  0.0659  3.4811  0.0005  0.1003  0.3586  ***

---
Signif. codes:  0 '***' 0.001 '**' 0.01 '*' 0.05 '.' 0.1 ' ' 1
```

(q) Meta-analytic results for differences in right lateral ventricle volume between HC vs MDD.

```
Random-Effects Model (k = 14; tau^2 estimator: REML)

tau^2 (estimated amount of total heterogeneity): 0.0049 (SE = 0.0160)
tau (square root of estimated tau^2 value):      0.0697
I^2 (total heterogeneity / total variability):   11.12%
H^2 (total variability / sampling variability):  1.13

Test for Heterogeneity:
```

```
Q(df = 13) = 14.9845, p-val = 0.3083

Model Results:

estimate      se    zval    pval   ci.lb   ci.ub
  0.1987  0.0561  3.5398  0.0004  0.0887  0.3087  ***

---
Signif. codes:  0 ‘***’ 0.001 ‘**’ 0.01 ‘*’ 0.05 ‘.’ 0.1 ‘ ’ 1
```

Supplementary Table 9: Meta-analytic results for differences in each regional volume between healthy controls (HC) vs autism spectrum disorder (ASD).

(a) Meta-analytic results for differences in left hippocampus volume between HC vs ASD.

```
Random-Effects Model (k = 7; tau^2 estimator: REML)

tau^2 (estimated amount of total heterogeneity): 0 (SE = 0.0213)
tau (square root of estimated tau^2 value):     0
I^2 (total heterogeneity / total variability):  0.00%
H^2 (total variability / sampling variability):  1.00

Test for Heterogeneity:
Q(df = 6) = 4.5868, p-val = 0.5978

Model Results:

estimate     se    zval    pval    ci.lb   ci.ub
 -0.0886  0.0754  -1.1747  0.2401  -0.2364  0.0592

---
Signif. codes:  0 '***' 0.001 '**' 0.01 '*' 0.05 '.' 0.1 ' ' 1
```

(b) Meta-analytic results for differences in right hippocampus volume between HC vs ASD.

```
Random-Effects Model (k = 7; tau^2 estimator: REML)

tau^2 (estimated amount of total heterogeneity): 0 (SE = 0.0197)
tau (square root of estimated tau^2 value):     0
I^2 (total heterogeneity / total variability):  0.00%
H^2 (total variability / sampling variability):  1.00

Test for Heterogeneity:
Q(df = 6) = 4.8063, p-val = 0.5689

Model Results:

estimate     se    zval    pval    ci.lb   ci.ub
 -0.1308  0.0726  -1.8015  0.0716  -0.2731  0.0115  .

---
Signif. codes:  0 '***' 0.001 '**' 0.01 '*' 0.05 '.' 0.1 ' ' 1
```

(c) Meta-analytic results for differences in left amygdala volume between HC vs ASD.

```
Random-Effects Model (k = 7; tau^2 estimator: REML)

tau^2 (estimated amount of total heterogeneity): 0 (SE = 0.0214)
tau (square root of estimated tau^2 value):     0
I^2 (total heterogeneity / total variability):  0.00%
H^2 (total variability / sampling variability):  1.00

Test for Heterogeneity:
Q(df = 6) = 2.2910, p-val = 0.8911
```

Model Results:

```
estimate     se     zval    pval    ci.lb   ci.ub
 -0.0221  0.0757  -0.2922  0.7701  -0.1705  0.1263

---
Signif. codes:  0 '***' 0.001 '**' 0.01 '*' 0.05 '.' 0.1 ' ' 1
```

(d) Meta-analytic results for differences in right amygdala volume between HC vs ASD.

```
Random-Effects Model (k = 7; tau^2 estimator: REML)

tau^2 (estimated amount of total heterogeneity): 0 (SE = 0.0210)
tau (square root of estimated tau^2 value):      0
I^2 (total heterogeneity / total variability):   0.00%
H^2 (total variability / sampling variability):  1.00

Test for Heterogeneity:
Q(df = 6) = 2.2828, p-val = 0.8919

Model Results:

estimate     se     zval    pval    ci.lb   ci.ub
 -0.0115  0.0750  -0.1533  0.8781  -0.1585  0.1355

---
Signif. codes:  0 '***' 0.001 '**' 0.01 '*' 0.05 '.' 0.1 ' ' 1
```

(e) Meta-analytic results for differences in left thalamus volume between HC vs ASD.

```
Random-Effects Model (k = 7; tau^2 estimator: REML)

tau^2 (estimated amount of total heterogeneity): 0 (SE = 0.0177)
tau (square root of estimated tau^2 value):      0
I^2 (total heterogeneity / total variability):   0.00%
H^2 (total variability / sampling variability):  1.00

Test for Heterogeneity:
Q(df = 6) = 3.8501, p-val = 0.6970

Model Results:

estimate     se     zval    pval    ci.lb    ci.ub
 -0.1683  0.0686  -2.4527  0.0142  -0.3028  -0.0338  *

---
Signif. codes:  0 '***' 0.001 '**' 0.01 '*' 0.05 '.' 0.1 ' ' 1
```

(f) Meta-analytic results for differences in right thalamus volume between HC vs ASD.

```
Random-Effects Model (k = 7; tau^2 estimator: REML)

tau^2 (estimated amount of total heterogeneity): 0.0207 (SE = 0.0306)
tau (square root of estimated tau^2 value):      0.1438
I^2 (total heterogeneity / total variability):   39.62%
```

```
H^2 (total variability / sampling variability):  1.66

Test for Heterogeneity:
Q(df = 6) = 9.5195, p-val = 0.1464

Model Results:

estimate     se    zval    pval    ci.lb    ci.ub
 -0.1739  0.0887  -1.9610  0.0499  -0.3477  -0.0001  *

---
Signif. codes:  0 ‘***’ 0.001 ‘**’ 0.01 ‘*’ 0.05 ‘.’ 0.1 ‘ ’ 1
```

(g) Meta-analytic results for differences in left accumbens volume between HC vs ASD.

```
Random-Effects Model (k = 7; tau^2 estimator: REML)

tau^2 (estimated amount of total heterogeneity): 0.0224 (SE = 0.0390)
tau (square root of estimated tau^2 value):      0.1496
I^2 (total heterogeneity / total variability):   33.08%
H^2 (total variability / sampling variability):  1.49

Test for Heterogeneity:
Q(df = 6) = 13.9806, p-val = 0.0299

Model Results:

estimate     se    zval    pval    ci.lb    ci.ub
 -0.1024  0.1005  -1.0198  0.3078  -0.2993  0.0944

---
Signif. codes:  0 ‘***’ 0.001 ‘**’ 0.01 ‘*’ 0.05 ‘.’ 0.1 ‘ ’ 1
```

(h) Meta-analytic results for differences in right accumbens volume between HC vs ASD.

```
Random-Effects Model (k = 7; tau^2 estimator: REML)

tau^2 (estimated amount of total heterogeneity): 0.1005 (SE = 0.0851)
tau (square root of estimated tau^2 value):      0.3170
I^2 (total heterogeneity / total variability):   71.26%
H^2 (total variability / sampling variability):  3.48

Test for Heterogeneity:
Q(df = 6) = 17.0675, p-val = 0.0090

Model Results:

estimate     se    zval    pval    ci.lb    ci.ub
 -0.1027  0.1457  -0.7052  0.4807  -0.3883  0.1828

---
Signif. codes:  0 ‘***’ 0.001 ‘**’ 0.01 ‘*’ 0.05 ‘.’ 0.1 ‘ ’ 1
```

(i) Meta-analytic results for differences in intracranial volume between HC vs ASD.

```
Random-Effects Model (k = 7; tau^2 estimator: REML)
```

tau^2 (estimated amount of total heterogeneity): 0.0166 (SE = 0.0327)
tau (square root of estimated tau^2 value): 0.1288
I^2 (total heterogeneity / total variability): 28.78%
H^2 (total variability / sampling variability): 1.40

Test for Heterogeneity:
Q(df = 6) = 7.8980, p-val = 0.2457

Model Results:

estimate se zval pval ci.lb ci.ub
-0.0354 0.0924 -0.3832 0.7016 -0.2166 0.1457

Signif. codes: 0 '***' 0.001 '**' 0.01 '*' 0.05 '.' 0.1 ' ' 1

(j) Meta-analytic results for differences in left caudate volume between HC vs ASD.

Random-Effects Model (k = 7; tau^2 estimator: REML)

tau^2 (estimated amount of total heterogeneity): 0.0025 (SE = 0.0195)
tau (square root of estimated tau^2 value): 0.0499
I^2 (total heterogeneity / total variability): 6.46%
H^2 (total variability / sampling variability): 1.07

Test for Heterogeneity:
Q(df = 6) = 8.1892, p-val = 0.2246

Model Results:

estimate se zval pval ci.lb ci.ub
-0.0178 0.0724 -0.2463 0.8055 -0.1597 0.1240

Signif. codes: 0 '***' 0.001 '**' 0.01 '*' 0.05 '.' 0.1 ' ' 1

(k) Meta-analytic results for differences in right caudate volume between HC vs ASD.

Random-Effects Model (k = 7; tau^2 estimator: REML)

tau^2 (estimated amount of total heterogeneity): 0 (SE = 0.0176)
tau (square root of estimated tau^2 value): 0
I^2 (total heterogeneity / total variability): 0.00%
H^2 (total variability / sampling variability): 1.00

Test for Heterogeneity:
Q(df = 6) = 3.2487, p-val = 0.7770

Model Results:

estimate se zval pval ci.lb ci.ub
0.0349 0.0689 0.5070 0.6121 -0.1001 0.1699

Signif. codes: 0 ‘***’ 0.001 ‘**’ 0.01 ‘*’ 0.05 ‘.’ 0.1 ‘ ’ 1

(l) Meta-analytic results for differences in left putamen volume between HC vs ASD.

```
Random-Effects Model (k = 7; tau^2 estimator: REML)

tau^2 (estimated amount of total heterogeneity): 0 (SE = 0.0164)
tau (square root of estimated tau^2 value):      0
I^2 (total heterogeneity / total variability):   0.00%
H^2 (total variability / sampling variability):  1.00

Test for Heterogeneity:
Q(df = 6) = 5.2312, p-val = 0.5145

Model Results:

estimate     se     zval    pval    ci.lb   ci.ub
 -0.0433  0.0667  -0.6499  0.5158  -0.1740  0.0873

---
Signif. codes:  0 ‘***’ 0.001 ‘**’ 0.01 ‘*’ 0.05 ‘.’ 0.1 ‘ ’ 1
```

(m) Meta-analytic results for differences in right putamen volume between HC vs ASD.

```
Random-Effects Model (k = 7; tau^2 estimator: REML)

tau^2 (estimated amount of total heterogeneity): 0.0137 (SE = 0.0254)
tau (square root of estimated tau^2 value):      0.1172
I^2 (total heterogeneity / total variability):   30.94%
H^2 (total variability / sampling variability):  1.45

Test for Heterogeneity:
Q(df = 6) = 10.6270, p-val = 0.1006

Model Results:

estimate     se     zval    pval    ci.lb   ci.ub
 -0.0621  0.0813  -0.7635  0.4452  -0.2214  0.0973

---
Signif. codes:  0 ‘***’ 0.001 ‘**’ 0.01 ‘*’ 0.05 ‘.’ 0.1 ‘ ’ 1
```

(n) Meta-analytic results for differences in left pallidum volume between HC vs ASD.

```
andom-Effects Model (k = 7; tau^2 estimator: REML)

tau^2 (estimated amount of total heterogeneity): 0 (SE = 0.0197)
tau (square root of estimated tau^2 value):      0
I^2 (total heterogeneity / total variability):   0.00%
H^2 (total variability / sampling variability):  1.00

Test for Heterogeneity:
Q(df = 6) = 2.0182, p-val = 0.9180

Model Results:
```

```
estimate     se    zval    pval    ci.lb   ci.ub
  0.0006  0.0729  0.0089  0.9929  -0.1422  0.1435

---
Signif. codes:  0 ‘***’ 0.001 ‘**’ 0.01 ‘*’ 0.05 ‘.’ 0.1 ‘ ’ 1
```

(o) Meta-analytic results for differences in right pallidum volume between HC vs ASD.

```
Random-Effects Model (k = 7; tau^2 estimator: REML)

tau^2 (estimated amount of total heterogeneity): 0 (SE = 0.0186)
tau (square root of estimated tau^2 value):      0
I^2 (total heterogeneity / total variability):   0.00%
H^2 (total variability / sampling variability):  1.00

Test for Heterogeneity:
Q(df = 6) = 5.7792, p-val = 0.4484

Model Results:

estimate     se     zval    pval    ci.lb   ci.ub
 -0.1027  0.0708  -1.4504  0.1469  -0.2414  0.0361

---
Signif. codes:  0 ‘***’ 0.001 ‘**’ 0.01 ‘*’ 0.05 ‘.’ 0.1 ‘ ’ 1
```

(p) Meta-analytic results for differences in left lateral ventricle volume between HC vs ASD.

```
Random-Effects Model (k = 7; tau^2 estimator: REML)

tau^2 (estimated amount of total heterogeneity): 0.0231 (SE = 0.0389)
tau (square root of estimated tau^2 value):      0.1519
I^2 (total heterogeneity / total variability):   34.33%
H^2 (total variability / sampling variability):  1.52

Test for Heterogeneity:
Q(df = 6) = 7.7452, p-val = 0.2574

Model Results:

estimate     se    zval    pval    ci.lb   ci.ub
  0.2753  0.1002  2.7467  0.0060  0.0788  0.4717  **

---
Signif. codes:  0 ‘***’ 0.001 ‘**’ 0.01 ‘*’ 0.05 ‘.’ 0.1 ‘ ’ 1
```

(q) Meta-analytic results for differences in right lateral ventricle volume between HC vs ASD.

```
Random-Effects Model (k = 7; tau^2 estimator: REML)

tau^2 (estimated amount of total heterogeneity): 0.0372 (SE = 0.0485)
tau (square root of estimated tau^2 value):      0.1929
I^2 (total heterogeneity / total variability):   45.54%
H^2 (total variability / sampling variability):  1.84

Test for Heterogeneity:
```

```
Q(df = 6) = 11.1315, p-val = 0.0844

Model Results:

estimate      se    zval    pval   ci.lb   ci.ub
  0.2436  0.1114  2.1864  0.0288  0.0252  0.4620  *

---
Signif. codes:  0 '***' 0.001 '**' 0.01 '*' 0.05 '.' 0.1 ' ' 1
```

Supplementary Table 10: Means and standard deviations (SDs) of laterality indices (LIs) of regional volumes for each protocol for each diagnostic group.

(a) Means and SDs of LIs of regional volumes for each protocol for healthy controls (HC) in HC vs schizophrenia (SZ) analysis.

Protocol Name	hippo		amyg		thal		accumb		caud		put		pal		LatVent	
	Mean	SD	Mean	SD	Mean	SD	Mean	SD	Mean	SD	Mean	SD	Mean	SD	Mean	SD
Osaka A	-0.008	0.045	-0.040	0.069	0.056	0.039	0.036	0.072	-0.002	0.029	0.009	0.042	-0.012	0.061	0.082	0.129
Osaka C	-0.023	0.047	-0.083	0.061	0.049	0.038	0.044	0.068	-0.025	0.030	0.002	0.047	-0.030	0.074	0.074	0.126
Nippon Med	0.025	0.032	0.035	0.056	0.075	0.031	-0.092	0.086	0.019	0.033	0.027	0.029	0.049	0.061	-0.087	0.130
Tokyo A	-0.014	0.037	0.018	0.046	0.031	0.029	0.033	0.080	0.047	0.041	0.011	0.029	0.070	0.060	0.084	0.133
Osaka B	-0.021	0.027	0.012	0.049	0.047	0.038	-0.030	0.092	0.063	0.041	0.013	0.036	-0.011	0.075	0.069	0.122
Toyama A	0.000	0.056	-0.017	0.077	0.035	0.045	-0.019	0.096	-0.015	0.043	0.014	0.034	-0.007	0.078	0.037	0.156
Kanazawa Med	-0.021	0.035	-0.012	0.051	0.042	0.039	-0.060	0.070	0.015	0.024	0.020	0.034	-0.026	0.066	0.053	0.117
Kyoto B	-0.027	0.029	-0.005	0.046	0.038	0.045	-0.097	0.124	0.023	0.032	0.009	0.036	-0.004	0.063	0.058	0.113
Kyoto A	-0.021	0.027	-0.013	0.040	0.039	0.025	-0.095	0.083	0.031	0.031	0.017	0.031	0.037	0.048	0.068	0.113
Nagoya A	-0.023	0.031	-0.011	0.054	0.039	0.050	0.030	0.086	-0.012	0.023	0.022	0.037	-0.028	0.086	0.074	0.111
Hokkaido A	-0.024	0.032	0.003	0.043	0.052	0.040	0.026	0.095	0.045	0.035	0.025	0.034	-0.026	0.061	0.078	0.100
Tokyo B	0.001	0.089	-0.017	0.084	0.063	0.034	0.025	0.088	0.064	0.043	0.017	0.045	-0.019	0.061	0.065	0.143
Kyushu A	-0.023	0.032	-0.061	0.042	0.053	0.033	-0.022	0.077	0.000	0.033	0.020	0.030	0.016	0.045	0.095	0.111
Toyama B	-0.019	0.028	0.012	0.050	0.027	0.042	0.007	0.063	-0.017	0.022	0.010	0.041	-0.009	0.086	0.088	0.138
Yamaguchi A	-0.016	0.041	-0.008	0.065	0.041	0.041	0.060	0.087	0.023	0.030	0.033	0.038	-0.012	0.083	0.091	0.094
Tokyo E	-0.019	0.028	-0.079	0.048	0.065	0.044	0.104	0.064	-0.017	0.035	0.031	0.047	-0.057	0.061	0.112	0.134
UOEH	-0.024	0.041	0.041	0.079	0.044	0.031	-0.086	0.138	0.081	0.048	0.041	0.036	0.004	0.062	0.055	0.099
Kyushu B	-0.016	0.021	-0.049	0.038	0.058	0.030	-0.021	0.089	-0.007	0.030	0.014	0.035	-0.004	0.054	0.059	0.090
Tokyo D	-0.027	0.029	-0.054	0.036	0.065	0.038	-0.011	0.064	-0.002	0.021	0.033	0.033	-0.013	0.045	0.082	0.127
Tokyo C	-0.020	0.091	-0.008	0.094	0.066	0.032	-0.020	0.121	0.075	0.053	0.023	0.040	0.016	0.044	0.088	0.136
Hokkaido B	-0.013	0.021	-0.036	0.035	0.067	0.034	0.015	0.082	0.011	0.039	0.037	0.031	-0.019	0.050	0.032	0.094
Tokushima B	-0.008	0.035	0.002	0.083	0.065	0.044	-0.001	0.111	0.082	0.084	0.058	0.061	0.015	0.061	0.073	0.095
Tokushima A	0.010	0.034	0.014	0.077	0.121	0.028	-0.012	0.107	0.060	0.036	0.035	0.025	0.018	0.057	0.011	0.099
Nagoya B	-0.022	0.046	0.030	0.058	0.014	0.065	-0.104	0.142	0.032	0.036	-0.001	0.067	-0.012	0.105	0.079	0.088

Abbreviations: hippo, hippocampus; amyg, amygdala; thal, thalamus; accumb, accumbens; caud, caudate; put, putamen; pal, pallidum; LLatVent, lateral ventricle.

(b) Means and SDs of LIs of regional volumes for each protocol for SZ in HC vs SZ analysis.

Protocol Name	hippo		amyg		thal		accumb		caud		put		pal		LatVent	
	Mean	SD	Mean	SD	Mean	SD	Mean	SD	Mean	SD	Mean	SD	Mean	SD	Mean	SD
Osaka A	-0.015	0.043	-0.044	0.073	0.062	0.039	0.041	0.070	-0.008	0.028	0.005	0.041	0.010	0.063	0.083	0.107
Osaka C	-0.017	0.056	-0.082	0.072	0.045	0.041	0.030	0.074	-0.026	0.026	0.010	0.045	-0.014	0.070	0.084	0.108
Nippon Med	0.034	0.043	0.028	0.061	0.085	0.037	-0.081	0.115	0.023	0.047	0.017	0.035	0.074	0.063	-0.083	0.111
Tokyo A	-0.009	0.030	0.004	0.044	0.022	0.030	0.029	0.078	0.038	0.037	0.007	0.024	0.076	0.049	0.079	0.114
Osaka B	-0.023	0.031	-0.001	0.048	0.043	0.034	-0.006	0.102	0.051	0.040	0.012	0.031	0.025	0.064	0.070	0.112
Toyama A	0.002	0.039	-0.015	0.062	0.036	0.045	-0.012	0.099	-0.013	0.024	0.014	0.030	0.014	0.080	0.014	0.143
Kanazawa Med	-0.019	0.034	-0.013	0.052	0.039	0.040	-0.068	0.091	0.009	0.027	0.020	0.034	-0.017	0.070	0.059	0.110
Kyoto B	-0.032	0.033	-0.008	0.053	0.040	0.040	-0.056	0.126	0.018	0.032	0.013	0.040	0.037	0.073	0.074	0.086
Kyoto A	-0.017	0.032	-0.003	0.049	0.034	0.032	-0.100	0.102	0.027	0.027	0.011	0.029	0.060	0.056	0.076	0.117
Nagoya A	-0.023	0.035	-0.018	0.055	0.044	0.035	0.066	0.091	-0.016	0.026	0.027	0.034	-0.008	0.109	0.072	0.121
Hokkaido A	-0.021	0.028	-0.006	0.041	0.042	0.030	0.025	0.078	0.040	0.035	0.021	0.033	0.011	0.056	0.037	0.115
Tokyo B	0.004	0.081	-0.040	0.075	0.055	0.032	0.042	0.088	0.060	0.044	0.024	0.038	0.011	0.054	0.084	0.102
Kyushu A	-0.037	0.043	-0.059	0.073	0.058	0.029	-0.036	0.076	-0.006	0.022	0.022	0.027	0.018	0.052	0.085	0.116
Toyama B	-0.021	0.030	-0.013	0.048	0.023	0.037	0.027	0.048	-0.020	0.018	0.015	0.035	0.011	0.086	0.079	0.126
Yamaguchi A	-0.017	0.049	-0.006	0.070	0.035	0.037	0.050	0.093	0.029	0.040	0.033	0.037	-0.010	0.092	0.067	0.098
Tokyo E	-0.015	0.026	-0.066	0.058	0.078	0.041	0.093	0.060	-0.018	0.024	0.020	0.047	-0.017	0.038	0.127	0.101
UOEH	-0.021	0.056	-0.017	0.052	0.079	0.030	-0.027	0.087	0.031	0.038	0.050	0.062	0.057	0.040	0.098	0.119
Kyushu B	-0.031	0.042	-0.066	0.051	0.055	0.026	-0.016	0.069	-0.001	0.022	0.012	0.030	0.026	0.052	0.085	0.089
Tokyo D	-0.026	0.020	-0.045	0.036	0.059	0.022	0.012	0.090	0.012	0.014	0.029	0.020	0.041	0.067	0.078	0.133
Tokyo C	-0.032	0.094	-0.043	0.166	0.057	0.042	0.027	0.129	0.078	0.044	0.038	0.031	0.020	0.067	0.104	0.111
Hokkaido B	-0.004	0.033	-0.024	0.042	0.056	0.031	0.001	0.074	-0.005	0.021	0.018	0.038	0.020	0.061	-0.006	0.142
Tokushima B	-0.007	0.033	0.015	0.107	0.073	0.039	-0.013	0.140	0.084	0.096	0.040	0.043	0.026	0.086	0.072	0.071
Tokushima A	-0.002	0.034	0.012	0.048	0.114	0.023	0.004	0.088	0.069	0.043	0.018	0.033	0.036	0.066	0.104	0.091
Nagoya B	-0.038	0.063	-0.007	0.059	0.052	0.050	-0.054	0.075	0.047	0.037	-0.003	0.065	-0.005	0.092	0.044	0.099

Abbreviations: hippo, hippocampus; amyg, amygdala; thal, thalamus; accumb, accumbens; caud, caudate; put, putamen; pal, pallidum; LLatVent, lateral ventricle.

(c) Means and SDs of LIs of regional volumes for each protocol for HC in HC vs bipolar disorder (BP) analysis.

Protocol Name	hippo		amyg		thal		accumb		caud		put		pal		LatVent	
	Mean	SD	Mean	SD	Mean	SD	Mean	SD	Mean	SD	Mean	SD	Mean	SD	Mean	SD

Osaka C	-0.023	0.047	-0.083	0.061	0.049	0.038	0.044	0.068	-0.025	0.030	0.002	0.047	-0.030	0.074	0.074	0.126
Kanazawa Med	-0.021	0.035	-0.012	0.051	0.042	0.039	-0.060	0.070	0.015	0.024	0.020	0.034	-0.026	0.066	0.053	0.117
Nagoya A	-0.023	0.031	-0.011	0.054	0.039	0.050	0.030	0.086	-0.012	0.023	0.022	0.037	-0.028	0.086	0.074	0.111
Yamaguchi B	-0.027	0.041	0.004	0.065	0.021	0.041	-0.013	0.087	-0.023	0.030	0.020	0.038	-0.006	0.083	0.076	0.094
Hokkaido A	-0.024	0.032	0.003	0.043	0.052	0.040	0.026	0.095	0.045	0.035	0.025	0.034	-0.026	0.061	0.078	0.100
Yamaguchi A	-0.016	0.041	-0.008	0.065	0.041	0.041	0.060	0.087	0.023	0.030	0.033	0.038	-0.012	0.083	0.091	0.094
Kyushu A	-0.023	0.032	-0.061	0.042	0.053	0.033	-0.022	0.077	0.000	0.033	0.020	0.030	0.016	0.045	0.095	0.111
Hiroshima D	-0.025	0.042	-0.021	0.062	0.029	0.029	-0.010	0.085	-0.018	0.026	0.026	0.035	0.011	0.079	0.074	0.104
Tokyo E	-0.019	0.028	-0.079	0.048	0.065	0.044	0.104	0.064	-0.017	0.035	0.031	0.047	-0.057	0.061	0.112	0.134
Kyushu B	-0.016	0.021	-0.049	0.038	0.058	0.030	-0.021	0.089	-0.007	0.030	0.014	0.035	-0.004	0.054	0.059	0.090

Abbreviations: hippo, hippocampus; amyg, amygdala; thal, thalamus; accumb, accumbens; caud, caudate; put, putamen; pal, pallidum; LLatVent, lateral ventricle.

(d) Means and SDs of LIs of regional volumes for each protocol for BP in HC vs BP analysis.

Protocol Name	hippo		amyg		thal		accumb		caud		put		pal		LatVent	
	Mean	SD	Mean	SD	Mean	SD	Mean	SD	Mean	SD	Mean	SD	Mean	SD	Mean	SD
Osaka C	-0.032	0.004	-0.064	0.055	0.041	0.021	0.018	0.112	0.002	0.048	0.023	0.027	0.016	0.045	0.082	0.060
Kanazawa Med	-0.028	0.028	-0.008	0.044	0.036	0.038	-0.087	0.079	0.016	0.026	0.010	0.040	-0.027	0.081	0.071	0.093
Nagoya A	-0.031	0.034	-0.012	0.057	0.055	0.037	0.046	0.092	-0.001	0.036	0.014	0.041	-0.023	0.113	0.068	0.096
Yamaguchi B	-0.019	0.029	0.008	0.063	0.044	0.061	0.013	0.060	-0.027	0.020	0.004	0.053	-0.018	0.077	0.110	0.094
Hokkaido A	-0.018	0.028	-0.004	0.044	0.041	0.031	0.035	0.075	0.050	0.040	0.017	0.031	0.009	0.070	0.054	0.096
Yamaguchi A	-0.017	0.010	-0.007	0.060	0.044	0.022	0.076	0.113	0.016	0.020	0.019	0.051	0.005	0.062	0.084	0.103
Kyushu A	-0.017	0.031	-0.068	0.049	0.050	0.024	-0.002	0.055	-0.009	0.028	0.021	0.020	0.018	0.046	0.050	0.104
Hiroshima D	-0.028	0.027	-0.018	0.052	0.022	0.027	-0.002	0.094	-0.018	0.023	0.024	0.032	0.034	0.052	0.044	0.105
Tokyo E	-0.027	0.034	-0.070	0.063	0.073	0.067	0.120	0.063	-0.014	0.020	0.018	0.058	-0.044	0.070	0.116	0.095
Kyushu B	-0.009	0.020	-0.057	0.050	0.046	0.020	-0.022	0.077	0.003	0.021	0.020	0.034	0.046	0.083	0.047	0.087

(e) Means and SDs of LIs of regional volumes for each protocol for HC in HC vs major depressive disorder (MDD) analysis.

Protocol Name	hippo		amyg		thal		accumb		caud		put		pal		LatVent	
	Mean	SD	Mean	SD	Mean	SD	Mean	SD	Mean	SD	Mean	SD	Mean	SD	Mean	SD
Osaka C	-0.023	0.047	-0.083	0.061	0.049	0.038	0.044	0.068	-0.025	0.030	0.002	0.047	-0.030	0.074	0.074	0.126
Osaka A	-0.008	0.045	-0.040	0.069	0.056	0.039	0.036	0.072	-0.002	0.029	0.009	0.042	-0.012	0.061	0.082	0.129
Osaka B	-0.021	0.027	0.012	0.049	0.047	0.038	-0.030	0.092	0.063	0.041	0.013	0.036	-0.011	0.075	0.069	0.122

Hokkaido A	-0.024	0.032	0.003	0.043	0.052	0.040	0.026	0.095	0.045	0.035	0.025	0.034	-0.026	0.061	0.078	0.100
Yamaguchi B	-0.027	0.040	0.004	0.073	0.021	0.040	-0.013	0.070	-0.023	0.022	0.020	0.036	-0.006	0.080	0.076	0.113
Kanazawa Med	-0.021	0.035	-0.012	0.051	0.042	0.039	-0.060	0.070	0.015	0.024	0.020	0.034	-0.026	0.066	0.053	0.117
Hiroshima A	0.000	0.070	-0.007	0.065	0.068	0.036	-0.014	0.072	0.052	0.039	0.034	0.035	-0.029	0.079	0.052	0.123
Hiroshima D	-0.025	0.042	-0.021	0.062	0.029	0.029	-0.010	0.085	-0.018	0.026	0.026	0.035	0.011	0.079	0.074	0.104
Yamaguchi A	-0.016	0.041	-0.008	0.065	0.041	0.041	0.060	0.087	0.023	0.030	0.033	0.038	-0.012	0.083	0.091	0.094
Kyushu A	-0.023	0.032	-0.061	0.042	0.053	0.033	-0.022	0.077	0.000	0.033	0.020	0.030	0.016	0.045	0.095	0.111
Tokyo E	-0.019	0.028	-0.079	0.048	0.065	0.044	0.104	0.064	-0.017	0.035	0.031	0.047	-0.057	0.061	0.112	0.134
UOEH	-0.024	0.041	0.041	0.079	0.044	0.031	-0.086	0.138	0.081	0.048	0.041	0.036	0.004	0.062	0.055	0.099
Hiroshima B	-0.023	0.034	0.033	0.032	0.036	0.042	-0.051	0.071	0.008	0.017	0.026	0.030	-0.020	0.069	0.068	0.121
Hiroshima C	-0.005	0.112	-0.010	0.089	0.064	0.044	0.016	0.091	0.075	0.042	0.039	0.052	0.007	0.078	0.087	0.118

Abbreviations: hippo, hippocampus; amyg, amygdala; thal, thalamus; accumb, accumbens; caud, caudate; put, putamen; pal, pallidum; LLatVent, lateral ventricle.

(f) Means and SDs of LIs of regional volumes for each protocol for MDD in HC vs MDD analysis.

Protocol Name	hippo		amyg		thal		accumb		caud		put		pal		LatVent	
	Mean	SD	Mean	SD	Mean	SD	Mean	SD	Mean	SD	Mean	SD	Mean	SD	Mean	SD
Osaka C	-0.026	0.022	-0.081	0.054	0.057	0.035	0.030	0.056	-0.014	0.031	-0.010	0.049	-0.033	0.078	0.089	0.073
Osaka A	0.001	0.029	-0.047	0.085	0.064	0.041	0.074	0.058	-0.007	0.026	-0.018	0.057	0.022	0.065	0.074	0.066
Osaka B	-0.059	0.073	-0.011	0.052	0.019	0.018	-0.028	0.065	0.037	0.042	0.034	0.027	0.019	0.041	0.055	0.073
Hokkaido A	-0.019	0.034	-0.003	0.051	0.043	0.034	0.033	0.085	0.039	0.033	0.023	0.032	0.006	0.075	0.070	0.091
Yamaguchi B	-0.027	0.032	-0.011	0.040	0.037	0.042	-0.023	0.072	-0.026	0.029	0.019	0.038	-0.019	0.086	0.063	0.116
Kanazawa Med	-0.022	0.035	-0.003	0.047	0.051	0.043	-0.077	0.096	0.010	0.026	0.015	0.040	-0.037	0.072	0.050	0.090
Hiroshima A	-0.006	0.115	-0.012	0.131	0.073	0.033	-0.014	0.087	0.047	0.045	0.030	0.046	-0.029	0.071	0.060	0.115
Hiroshima D	-0.027	0.033	-0.011	0.057	0.014	0.036	0.026	0.083	-0.010	0.028	0.026	0.040	0.024	0.076	0.076	0.111
Yamaguchi A	-0.024	0.030	-0.017	0.052	0.042	0.038	0.046	0.084	0.015	0.032	0.031	0.036	-0.009	0.096	0.106	0.092
Kyushu A	-0.025	0.036	-0.038	0.032	0.027	0.018	0.006	0.095	-0.004	0.025	0.040	0.020	-0.008	0.043	0.103	0.108
Tokyo E	-0.023	0.026	-0.071	0.054	0.064	0.045	0.106	0.049	-0.021	0.020	0.033	0.042	-0.044	0.061	0.126	0.081
UOEH	-0.017	0.045	0.073	0.111	0.038	0.034	-0.166	0.135	0.086	0.051	0.038	0.048	0.003	0.057	0.034	0.079
Hiroshima B	-0.030	0.038	0.005	0.044	0.048	0.042	-0.039	0.099	0.006	0.034	0.032	0.033	-0.017	0.083	0.049	0.099
Hiroshima C	0.002	0.064	-0.017	0.095	0.035	0.020	-0.056	0.063	0.088	0.030	0.060	0.042	0.009	0.094	0.134	0.099

Abbreviations: hippo, hippocampus; amyg, amygdala; thal, thalamus; accumb, accumbens; caud, caudate; put, putamen; pal, pallidum; LLatVent, lateral ventricle.

(g) Means and SDs of LIs of regional volumes for each protocol for HC in HC vs autism spectrum disorder (ASD) analysis.

Protocol Name	hippo		amyg		thal		accumb		caud		put		pal		LatVent	
	Mean	SD	Mean	SD	Mean	SD	Mean	SD	Mean	SD	Mean	SD	Mean	SD	Mean	SD
Osaka A	-0.008	0.045	-0.040	0.069	0.056	0.039	0.036	0.072	-0.002	0.029	0.009	0.042	-0.012	0.061	0.082	0.129
Osaka C	-0.023	0.047	-0.083	0.061	0.049	0.038	0.044	0.068	-0.025	0.030	0.002	0.047	-0.030	0.074	0.074	0.126
Osaka B	-0.021	0.027	0.012	0.049	0.047	0.038	-0.030	0.092	0.063	0.041	0.013	0.036	-0.011	0.075	0.069	0.122
Showa	-0.033	0.026	-0.002	0.054	0.024	0.032	-0.057	0.079	0.004	0.028	0.025	0.033	0.037	0.061	0.069	0.115
Nagoya A	-0.023	0.031	-0.011	0.054	0.039	0.050	0.030	0.086	-0.012	0.023	0.022	0.037	-0.028	0.086	0.074	0.111
Tokyo B	0.001	0.089	-0.017	0.084	0.063	0.034	0.025	0.088	0.064	0.043	0.017	0.045	-0.019	0.061	0.065	0.143
Tokyo E	-0.019	0.028	-0.079	0.048	0.065	0.044	0.104	0.064	-0.017	0.035	0.031	0.047	-0.057	0.061	0.112	0.134

Abbreviations: hippo, hippocampus; amyg, amygdala; thal, thalamus; accumb, accumbens; caud, caudate; put, putamen; pal, pallidum; LLatVent, lateral ventricle.

(h) Means and SDs of LIs of regional volumes for each protocol for ASD in HC vs ASD analysis.

Protocol Name	hippo		amyg		thal		accumb		caud		put		pal		LatVent	
	Mean	SD	Mean	SD	Mean	SD	Mean	SD	Mean	SD	Mean	SD	Mean	SD	Mean	SD
Osaka A	-0.003	0.036	-0.041	0.069	0.066	0.034	0.048	0.061	-0.006	0.025	0.008	0.038	0.004	0.068	0.102	0.099
Osaka C	0.000	0.029	-0.077	0.058	0.067	0.045	0.080	0.053	-0.016	0.023	0.003	0.041	-0.018	0.103	0.108	0.118
Osaka B	-0.017	0.024	0.009	0.054	0.044	0.028	-0.038	0.071	0.039	0.035	0.010	0.034	-0.029	0.076	0.075	0.114
Showa	-0.037	0.035	0.004	0.063	0.017	0.035	-0.058	0.069	0.000	0.025	0.021	0.029	0.025	0.069	0.079	0.146
Nagoya A	-0.003	0.025	-0.008	0.049	0.024	0.034	0.024	0.076	-0.019	0.018	0.029	0.041	0.000	0.085	0.021	0.077
Tokyo B	-0.036	0.088	-0.043	0.097	0.057	0.037	0.029	0.106	0.071	0.045	0.028	0.039	0.005	0.057	0.108	0.120
Tokyo E	0.001	0.021	-0.097	0.048	0.097	0.055	0.123	0.063	-0.034	0.011	-0.004	0.042	-0.023	0.084	0.002	0.070

Abbreviations: hippo, hippocampus; amyg, amygdala; thal, thalamus; accumb, accumbens; caud, caudate; put, putamen; pal, pallidum; LLatVent, lateral ventricle.

Supplementary Table 11: Group differences in laterality indices (LIs) of regional volumes within each protocol.

(a) Group differences and standard errors (SEs) in LIs of regional volumes within each protocol in healthy controls (HC) vs schizophrenia (SZ) analysis.

Protocol Name	hippo		amyg		thal		accumb		caud		put		pal		LatVent	
	Beta	SE	Beta	SE	Beta	SE	Beta	SE	Beta	SE	Beta	SE	Beta	SE	Beta	SE
Osaka A	-0.007	0.004	-0.004	0.006	0.006	0.004	0.001	0.006	-0.004	0.003	-0.003	0.004	0.018	0.006	0.002	0.011
Osaka C	0.006	0.006	0.000	0.007	-0.004	0.004	-0.014	0.008	-0.001	0.003	0.008	0.005	0.016	0.008	0.010	0.014
Nippon Med	0.011	0.004	-0.002	0.006	0.007	0.004	0.011	0.011	0.002	0.004	-0.008	0.004	0.031	0.007	0.006	0.013
Tokyo A	0.006	0.004	-0.014	0.005	-0.009	0.003	-0.002	0.009	-0.011	0.005	-0.004	0.003	0.007	0.007	-0.005	0.015
Osaka B	-0.002	0.004	-0.011	0.007	-0.006	0.005	0.030	0.014	-0.013	0.006	-0.002	0.005	0.038	0.011	-0.002	0.018
Toyama A	0.002	0.006	0.002	0.009	0.000	0.006	0.007	0.013	0.002	0.005	0.000	0.004	0.022	0.010	-0.023	0.020
Kanazawa Med	0.003	0.005	-0.003	0.007	-0.005	0.006	-0.008	0.011	-0.007	0.004	-0.002	0.005	0.007	0.010	0.005	0.016
Kyoto B	-0.006	0.005	-0.005	0.008	0.002	0.008	0.038	0.022	-0.006	0.006	0.002	0.007	0.041	0.012	0.014	0.019
Kyoto A	0.003	0.004	0.013	0.007	-0.004	0.004	-0.005	0.014	-0.005	0.004	-0.005	0.005	0.025	0.008	0.006	0.017
Nagoya A	0.003	0.006	-0.007	0.009	0.005	0.008	0.029	0.015	-0.005	0.004	0.005	0.006	0.038	0.015	-0.004	0.020
Hokkaido A	0.001	0.006	-0.001	0.009	-0.010	0.007	0.007	0.017	-0.006	0.007	0.004	0.007	0.024	0.012	-0.044	0.024
Tokyo B	0.004	0.017	-0.019	0.016	-0.008	0.007	0.015	0.017	-0.005	0.009	0.007	0.008	0.028	0.012	0.021	0.026
Kyushu A	-0.014	0.007	-0.001	0.011	0.008	0.006	-0.016	0.015	-0.003	0.006	-0.001	0.006	0.000	0.010	0.000	0.023
Toyama B	-0.002	0.005	-0.023	0.009	-0.006	0.007	0.018	0.010	-0.002	0.004	0.006	0.007	0.023	0.016	-0.009	0.025
Yamaguchi A	-0.001	0.010	0.000	0.015	-0.005	0.009	-0.012	0.020	0.008	0.007	-0.001	0.008	0.002	0.019	-0.018	0.021
Tokyo E	0.003	0.008	0.022	0.014	0.013	0.012	-0.003	0.017	-0.001	0.009	-0.010	0.013	0.030	0.014	-0.003	0.033
UOEH	-0.002	0.014	-0.039	0.021	0.029	0.009	0.032	0.039	-0.037	0.013	0.021	0.013	0.043	0.018	0.034	0.032
Kyushu B	-0.013	0.009	-0.016	0.012	-0.004	0.007	0.009	0.020	0.006	0.007	0.000	0.008	0.031	0.014	0.023	0.024
Tokyo D	0.003	0.009	0.008	0.012	-0.006	0.012	0.034	0.022	0.014	0.007	-0.006	0.010	0.051	0.017	0.002	0.041
Tokyo C	-0.018	0.030	-0.038	0.038	-0.009	0.011	0.051	0.039	0.003	0.017	0.014	0.013	0.004	0.017	0.015	0.043
Hokkaido B	0.017	0.008	0.012	0.012	-0.007	0.010	-0.016	0.025	-0.019	0.009	-0.012	0.011	0.045	0.018	-0.055	0.038
Tokushima B	0.000	0.011	0.012	0.031	0.007	0.013	-0.017	0.039	0.000	0.029	-0.021	0.016	0.010	0.023	0.001	0.026
Tokushima A	-0.010	0.011	0.009	0.021	-0.004	0.008	0.012	0.034	0.014	0.013	-0.014	0.009	0.029	0.019	0.087	0.032
Nagoya B	-0.039	0.037	-0.108	0.035	0.076	0.036	-0.011	0.068	0.032	0.023	-0.027	0.042	0.014	0.064	-0.024	0.062

Abbreviations: hippo, hippocampus; amyg, amygdala; thal, thalamus; accumb, accumbens; caud, caudate; put, putamen; pal, pallidum; LLatVent, lateral ventricle.

(b) Group differences and SEs in LIs of regional volumes within each protocol in HC vs bipolar disorder (BP) analysis.

Protocol Name	hippo		amyg		thal		accumb		caud		put		pal		LatVent	
	Beta	SE	Beta	SE	Beta	SE	Beta	SE	Beta	SE	Beta	SE	Beta	SE	Beta	SE
Osaka C	-0.003	0.021	0.020	0.027	-0.014	0.017	-0.019	0.031	0.023	0.014	0.026	0.022	0.033	0.033	0.021	0.058
Kanazawa Med	-0.006	0.007	0.007	0.010	-0.006	0.008	-0.029	0.015	0.002	0.005	-0.009	0.007	-0.004	0.015	0.033	0.023

Nagoya A	-0.003	0.008	0.003	0.015	0.016	0.013	-0.005	0.023	0.010	0.007	-0.020	0.010	0.017	0.024	-0.016	0.029
Yamaguchi B	0.006	0.011	0.001	0.020	0.023	0.012	0.025	0.019	-0.003	0.006	-0.015	0.010	-0.013	0.022	0.038	0.031
Hokkaido A	0.005	0.006	-0.007	0.009	-0.011	0.007	0.005	0.016	0.004	0.008	-0.008	0.007	0.031	0.013	-0.025	0.020
Yamaguchi A	0.000	0.015	0.001	0.024	0.003	0.015	0.017	0.033	-0.007	0.011	-0.018	0.015	0.021	0.031	-0.002	0.035
Kyushu A	0.012	0.009	-0.007	0.013	0.004	0.009	0.044	0.020	-0.003	0.009	0.000	0.008	-0.006	0.013	-0.029	0.032
Hiroshima D	-0.004	0.009	-0.002	0.014	-0.009	0.007	0.003	0.021	0.000	0.006	-0.002	0.008	0.030	0.017	-0.029	0.025
Tokyo E	-0.008	0.008	0.016	0.015	0.006	0.014	0.019	0.017	0.001	0.008	-0.016	0.013	0.011	0.018	0.000	0.032
Kyushu B	0.011	0.009	0.002	0.018	-0.015	0.012	0.014	0.037	0.017	0.011	0.012	0.014	0.063	0.027	-0.010	0.036

Abbreviations: hippo, hippocampus; amyg, amygdala; thal, thalamus; accumb, accumbens; caud, caudate; put, putamen; pal, pallidum; LLatVent, lateral ventricle.

(c) Group differences and SEs in LIs of regional volumes within each protocol in HC vs major depressive disorder (MDD) analysis.

Protocol Name	hippo		amyg		thal		accumb		caud		put		pal		LatVent	
	Beta	SE	Beta	SE	Beta	SE	Beta	SE	Beta	SE	Beta	SE	Beta	SE	Beta	SE
Osaka C	-0.001	0.011	-0.008	0.014	0.009	0.009	-0.014	0.016	0.010	0.007	-0.006	0.011	-0.004	0.018	0.024	0.030
Osaka A	0.008	0.012	-0.001	0.019	0.005	0.011	0.035	0.019	-0.005	0.008	-0.024	0.012	0.033	0.017	-0.008	0.035
Osaka B	-0.031	0.011	-0.011	0.018	-0.023	0.013	-0.003	0.033	-0.029	0.015	0.015	0.013	0.032	0.027	-0.030	0.044
Hokkaido A	0.004	0.006	-0.007	0.009	-0.008	0.006	0.007	0.016	-0.006	0.006	-0.002	0.006	0.032	0.014	-0.009	0.017
Yamaguchi B	0.003	0.006	-0.014	0.011	0.020	0.007	-0.010	0.012	-0.002	0.004	-0.005	0.006	-0.015	0.014	-0.015	0.019
Kanazawa Med	0.001	0.007	0.011	0.009	0.010	0.008	-0.012	0.015	-0.002	0.005	-0.006	0.007	-0.010	0.013	0.005	0.021
Hiroshima A	-0.006	0.018	-0.005	0.019	0.007	0.006	-0.003	0.014	-0.001	0.008	-0.003	0.007	0.001	0.014	0.008	0.022
Hiroshima D	0.000	0.007	0.003	0.011	-0.015	0.007	0.033	0.017	0.009	0.005	0.000	0.007	0.020	0.015	-0.005	0.021
Yamaguchi A	-0.007	0.010	-0.009	0.016	0.001	0.010	-0.017	0.021	-0.007	0.007	0.000	0.009	-0.003	0.021	0.008	0.023
Kyushu A	0.002	0.012	0.021	0.016	-0.016	0.012	0.053	0.029	0.001	0.012	0.016	0.011	-0.030	0.017	0.037	0.042
Tokyo E	-0.004	0.006	0.011	0.011	-0.003	0.010	0.002	0.013	-0.004	0.006	0.003	0.010	0.010	0.014	0.019	0.024
UOEH	0.004	0.011	0.020	0.023	-0.006	0.008	-0.072	0.036	-0.003	0.012	-0.003	0.011	0.006	0.016	-0.019	0.025
Hiroshima B	-0.005	0.010	-0.028	0.011	0.014	0.011	0.009	0.025	-0.002	0.008	0.005	0.009	-0.002	0.021	-0.021	0.029
Hiroshima C	-0.010	0.041	-0.010	0.036	-0.029	0.016	-0.063	0.034	0.011	0.016	0.015	0.020	-0.002	0.032	0.037	0.045

Abbreviations: hippo, hippocampus; amyg, amygdala; thal, thalamus; accumb, accumbens; caud, caudate; put, putamen; pal, pallidum; LLatVent, lateral ventricle.

(d) Group differences and SEs in LIs of regional volumes within each protocol in HC vs autism spectrum disorder (ASD) analysis.

Protocol Name	hippo		amyg		thal		accumb		caud		put		pal		LatVent	
	Beta	SE	Beta	SE	Beta	SE	Beta	SE	Beta	SE	Beta	SE	Beta	SE	Beta	SE
Osaka A	0.003	0.007	0.006	0.011	0.006	0.006	0.011	0.012	-0.004	0.005	0.003	0.007	0.016	0.010	0.018	0.021
Osaka C	0.023	0.011	0.009	0.014	0.018	0.009	0.037	0.016	0.010	0.007	-0.001	0.011	0.013	0.018	0.031	0.030
Osaka B	0.003	0.008	-0.005	0.015	-0.002	0.011	-0.013	0.026	-0.023	0.012	-0.001	0.011	-0.020	0.022	0.010	0.036
Showa	-0.004	0.006	0.003	0.010	-0.005	0.006	-0.005	0.013	-0.004	0.005	-0.004	0.006	-0.010	0.012	0.010	0.024

Nagoya A	0.017	0.010	-0.003	0.017	-0.016	0.015	0.002	0.027	-0.008	0.007	0.013	0.012	0.010	0.026	-0.046	0.034
Tokyo B	-0.024	0.019	-0.026	0.019	-0.007	0.008	0.005	0.021	0.011	0.010	0.015	0.009	0.025	0.013	0.048	0.030
Tokyo E	0.022	0.013	0.001	0.021	0.025	0.022	0.004	0.029	-0.016	0.016	-0.033	0.022	0.027	0.031	-0.096	0.060

Abbreviations: hippo, hippocampus; amyg, amygdala; thal, thalamus; accumb, accumbens; caud, caudate; put, putamen; pal, pallidum; LLatVent, lateral ventricle.

Supplementary Table 12: Cohen's *d* effect sizes for group differences in laterality indices (LIs) of regional volumes within each protocol in each psychiatric disorder.

(a) Cohen's *d* effect sizes and standard errors (SEs) for group differences in LIs of regional volumes within each protocol in healthy controls (HC) vs schizophrenia (SZ) analysis.

Protocol Name	hippo		amyg		thal		accumb		caud		put		pal		LatVent	
	d	SE	*d*	SE	*d*	SE	*d*	SE	*d*	SE	*d*	SE	*d*	SE	*d*	SE
Osaka A	-0.16	0.09	-0.05	0.09	0.17	0.09	0.02	0.09	-0.15	0.09	-0.07	0.09	0.29	0.09	0.01	0.09
Osaka C	0.12	0.11	0.00	0.11	-0.10	0.11	-0.20	0.11	-0.02	0.11	0.17	0.11	0.22	0.11	0.08	0.11
Nippon Med	0.29	0.11	-0.03	0.11	0.22	0.11	0.10	0.11	0.06	0.11	-0.26	0.11	0.50	0.11	0.05	0.11
Tokyo A	0.16	0.12	-0.32	0.12	-0.32	0.12	-0.03	0.12	-0.27	0.12	-0.16	0.12	0.13	0.12	-0.04	0.12
Osaka B	-0.07	0.15	-0.23	0.15	-0.15	0.15	0.31	0.15	-0.32	0.15	-0.05	0.15	0.53	0.15	-0.02	0.15
Toyama A	0.04	0.13	0.03	0.13	0.01	0.13	0.07	0.13	0.06	0.13	0.00	0.13	0.27	0.13	-0.15	0.13
Kanazawa Med	0.10	0.14	-0.06	0.14	-0.11	0.14	-0.10	0.14	-0.26	0.14	-0.07	0.14	0.10	0.14	0.05	0.14
Kyoto B	-0.21	0.17	-0.10	0.17	0.04	0.18	0.30	0.17	-0.20	0.18	0.06	0.18	0.62	0.18	0.13	0.18
Kyoto A	0.11	0.15	0.30	0.15	-0.14	0.15	-0.05	0.15	-0.17	0.15	-0.18	0.15	0.48	0.15	0.05	0.15
Nagoya A	0.10	0.17	-0.12	0.17	0.10	0.17	0.33	0.17	-0.22	0.17	0.14	0.17	0.40	0.17	-0.04	0.17
Hokkaido A	0.05	0.21	-0.02	0.21	-0.32	0.21	0.08	0.21	-0.16	0.21	0.11	0.21	0.41	0.20	-0.39	0.21
Tokyo B	0.04	0.20	-0.24	0.20	-0.24	0.20	0.17	0.20	-0.11	0.20	0.15	0.19	0.48	0.20	0.16	0.20
Kyushu A	-0.40	0.20	-0.02	0.20	0.24	0.20	-0.21	0.19	-0.11	0.20	-0.02	0.20	-0.01	0.20	0.00	0.20
Toyama B	-0.07	0.19	-0.48	0.19	-0.16	0.18	0.33	0.19	-0.08	0.18	0.15	0.19	0.27	0.19	-0.07	0.19
Yamaguchi A	-0.03	0.22	-0.01	0.22	-0.13	0.22	-0.14	0.22	0.23	0.22	-0.03	0.22	0.02	0.22	-0.19	0.22
Tokyo E	0.11	0.28	0.42	0.27	0.31	0.27	-0.05	0.27	-0.03	0.28	-0.21	0.28	0.57	0.27	-0.02	0.27
UOEH	-0.05	0.31	-0.53	0.29	0.96	0.30	0.25	0.30	-0.79	0.29	0.48	0.30	0.73	0.30	0.33	0.31
Kyushu B	-0.39	0.26	-0.35	0.26	-0.15	0.27	0.11	0.26	0.21	0.25	0.01	0.25	0.58	0.27	0.26	0.26
Tokyo D	0.09	0.31	0.23	0.34	-0.17	0.34	0.48	0.32	0.69	0.33	-0.18	0.34	1.03	0.34	0.01	0.32
Tokyo C	-0.19	0.33	-0.33	0.33	-0.27	0.33	0.41	0.32	0.07	0.33	0.37	0.34	0.07	0.34	0.12	0.33
Hokkaido B	0.59	0.30	0.31	0.31	-0.22	0.31	-0.21	0.32	-0.62	0.31	-0.33	0.31	0.79	0.31	-0.45	0.31
Tokushima B	0.00	0.32	0.12	0.32	0.16	0.32	-0.13	0.31	0.00	0.32	-0.39	0.30	0.14	0.30	0.01	0.32
Tokushima A	-0.29	0.33	0.14	0.32	-0.17	0.32	0.12	0.34	0.36	0.33	-0.47	0.33	0.48	0.31	0.91	0.34

Nagoya B	-0.69	0.65	-1.83	0.59	1.34	0.64	-0.10	0.64	0.87	0.64	-0.41	0.64	0.15	0.65	-0.25	0.65

Abbreviations: hippo, hippocampus; amyg, amygdala; thal, thalamus; accumb, accumbens; caud, caudate; put, putamen; pal, pallidum; LLatVent, lateral ventricle.

(b) Cohen's *d* effect sizes and SEs for group differences in LIs of regional volumes within each protocol in HC vs bipolar disorder (BP) analysis.

Protocol Name	hippo		amyg		thal		accumb		caud		put		pal		LatVent	
	d	SE	*d*	SE	*d*	SE	*d*	SE	*d*	SE	*d*	SE	*d*	SE	*d*	SE
Osaka C	-0.06	0.46	0.32	0.44	-0.38	0.45	-0.28	0.45	0.77	0.46	0.55	0.46	0.46	0.45	0.17	0.46
Kanazawa Med	-0.17	0.21	0.14	0.21	-0.16	0.21	-0.40	0.21	0.08	0.21	-0.26	0.21	-0.06	0.21	0.29	0.21
Nagoya A	-0.08	0.27	0.06	0.27	0.33	0.27	-0.05	0.26	0.38	0.26	-0.52	0.26	0.19	0.27	-0.15	0.27
Yamaguchi B	0.15	0.27	0.01	0.28	0.53	0.27	0.36	0.28	-0.16	0.28	-0.39	0.27	-0.16	0.28	0.34	0.28
Hokkaido A	0.18	0.21	-0.16	0.21	-0.34	0.21	0.06	0.20	0.10	0.21	-0.26	0.21	0.46	0.20	-0.25	0.21
Yamaguchi A	0.00	0.38	0.02	0.38	0.08	0.38	0.19	0.37	-0.25	0.37	-0.46	0.37	0.25	0.37	-0.02	0.37
Kyushu A	0.37	0.29	-0.17	0.29	0.13	0.29	0.60	0.28	-0.09	0.29	0.00	0.29	-0.12	0.29	-0.27	0.29
Hiroshima D	-0.10	0.24	-0.04	0.24	-0.32	0.24	0.03	0.24	-0.01	0.24	-0.07	0.23	0.43	0.24	-0.27	0.24
Tokyo E	-0.25	0.28	0.29	0.27	0.11	0.27	0.30	0.27	0.04	0.27	-0.32	0.26	0.17	0.28	0.00	0.27
Kyushu B	0.51	0.44	0.06	0.43	-0.54	0.44	0.16	0.42	0.61	0.40	0.33	0.41	1.02	0.43	-0.11	0.40

Abbreviations: hippo, hippocampus; amyg, amygdala; thal, thalamus; accumb, accumbens; caud, caudate; put, putamen; pal, pallidum; LLatVent, lateral ventricle.

(c) Cohen's *d* effect sizes and SEs for group differences in LIs of regional volumes within each protocol in HC vs major depressive disorder (MDD) analysis.

Protocol Name	hippo		amyg		thal		accumb		caud		put		pal		LatVent	
	d	SE	*d*	SE	*d*	SE	*d*	SE	*d*	SE	*d*	SE	*d*	SE	*d*	SE
Osaka C	-0.03	0.24	-0.13	0.23	0.25	0.24	-0.21	0.24	0.33	0.24	-0.13	0.24	-0.05	0.24	0.19	0.24
Osaka A	0.18	0.27	-0.02	0.27	0.14	0.27	0.49	0.27	-0.17	0.27	-0.57	0.27	0.53	0.27	-0.06	0.27
Osaka B	-1.03	0.36	-0.22	0.37	-0.62	0.36	-0.03	0.36	-0.70	0.37	0.41	0.37	0.44	0.37	-0.25	0.37
Hokkaido A	0.13	0.19	-0.13	0.19	-0.23	0.18	0.08	0.19	-0.18	0.18	-0.06	0.19	0.44	0.19	-0.10	0.19
Yamaguchi B	0.07	0.17	-0.21	0.17	0.49	0.16	-0.14	0.17	-0.06	0.17	-0.13	0.16	-0.18	0.17	-0.13	0.17
Kanazawa Med	0.02	0.19	0.22	0.19	0.24	0.19	-0.16	0.19	-0.07	0.18	-0.16	0.19	-0.14	0.19	0.04	0.19

Hiroshima A	-0.06	0.18	-0.05	0.18	0.20	0.18	-0.04	0.18	-0.03	0.18	-0.08	0.18	0.01	0.18	0.06	0.18
Hiroshima D	0.00	0.20	0.04	0.19	-0.45	0.20	0.39	0.20	0.34	0.20	0.00	0.20	0.26	0.20	-0.04	0.20
Yamaguchi A	-0.19	0.25	-0.14	0.25	0.02	0.25	-0.19	0.25	-0.23	0.24	0.00	0.25	-0.04	0.25	0.08	0.24
Kyushu A	0.07	0.38	0.50	0.38	-0.50	0.38	0.67	0.37	0.02	0.38	0.57	0.38	-0.66	0.38	0.34	0.38
Tokyo E	-0.14	0.22	0.23	0.22	-0.06	0.22	0.03	0.22	-0.16	0.22	0.07	0.22	0.17	0.22	0.17	0.22
UOEH	0.09	0.26	0.23	0.25	-0.20	0.26	-0.53	0.26	-0.05	0.25	-0.09	0.26	0.10	0.26	-0.20	0.27
Hiroshima B	-0.13	0.27	-0.68	0.27	0.32	0.27	0.10	0.28	-0.07	0.28	0.16	0.27	-0.03	0.27	-0.20	0.28
Hiroshima C	-0.09	0.39	-0.12	0.40	-0.70	0.40	-0.72	0.39	0.27	0.39	0.30	0.39	-0.02	0.39	0.32	0.39

Abbreviations: hippo, hippocampus; amyg, amygdala; thal, thalamus; accumb, accumbens; caud, caudate; put, putamen; pal, pallidum; LLatVent, lateral ventricle.

(d) Cohen's *d* effect sizes and SEs for group differences in LIs of regional volumes within each protocol in HC vs autism spectrum disorder (ASD) analysis.

Protocol Name	hippo		amyg		thal		accumb		caud		put		pal		LatVent	
	d	SE	*d*	SE	*d*	SE	*d*	SE	*d*	SE	*d*	SE	*d*	SE	*d*	SE
Osaka A	0.07	0.16	0.08	0.16	0.15	0.16	0.15	0.16	-0.13	0.16	0.07	0.16	0.26	0.16	0.14	0.16
Osaka C	0.49	0.24	0.14	0.23	0.47	0.24	0.54	0.24	0.34	0.24	-0.02	0.24	0.18	0.24	0.25	0.24
Osaka B	0.09	0.29	-0.09	0.30	-0.05	0.29	-0.14	0.29	-0.57	0.30	-0.03	0.30	-0.27	0.30	0.08	0.30
Showa	-0.12	0.18	0.06	0.18	-0.16	0.18	-0.07	0.18	-0.15	0.18	-0.14	0.18	-0.16	0.18	0.08	0.18
Nagoya A	0.54	0.31	-0.05	0.31	-0.33	0.32	0.02	0.31	-0.33	0.31	0.33	0.31	0.12	0.31	-0.42	0.31
Tokyo B	-0.27	0.22	-0.30	0.22	-0.19	0.22	0.05	0.22	0.24	0.22	0.35	0.22	0.41	0.22	0.35	0.22
Tokyo E	0.79	0.48	0.01	0.45	0.56	0.48	0.07	0.46	-0.49	0.48	-0.71	0.48	0.43	0.48	-0.75	0.47

Abbreviations: hippo, hippocampus; amyg, amygdala; thal, thalamus; accumb, accumbens; caud, caudate; put, putamen; pal, pallidum; LLatVent, lateral ventricle.

Supplementary Table 13: Meta-analytic results for differences in laterality indices (LIs) of each regional volume between healthy controls (HC) vs schizophrenia (SZ).

(a) Meta-analytic results for differences in LIs for hippocampus volume between HC vs SZ.

```
Random-Effects Model (k = 24; tau^2 estimator: REML)

tau^2 (estimated amount of total heterogeneity): 0.0102 (SE = 0.0114)
tau (square root of estimated tau^2 value):      0.1010
I^2 (total heterogeneity / total variability):   25.44%
H^2 (total variability / sampling variability):  1.34

Test for Heterogeneity:
Q(df = 23) = 29.0123, p-val = 0.1799

Model Results:

estimate     se    zval    pval    ci.lb   ci.ub
  0.0154  0.0429  0.3596  0.7191  -0.0687  0.0996

---
Signif. codes:  0 '***' 0.001 '**' 0.01 '*' 0.05 '.' 0.1 ' ' 1
```

(b) Meta-analytic results for differences in LIs for amygdala volume between HC vs SZ.

```
Random-Effects Model (k = 24; tau^2 estimator: REML)

tau^2 (estimated amount of total heterogeneity): 0.0091 (SE = 0.0109)
tau (square root of estimated tau^2 value):      0.0956
I^2 (total heterogeneity / total variability):   23.57%
H^2 (total variability / sampling variability):  1.31

Test for Heterogeneity:
Q(df = 23) = 37.9306, p-val = 0.0259

Model Results:

estimate     se    zval    pval    ci.lb   ci.ub
 -0.0799  0.0421  -1.8956  0.0580  -0.1624  0.0027  .

---
Signif. codes:  0 '***' 0.001 '**' 0.01 '*' 0.05 '.' 0.1 ' ' 1
```

(c) Meta-analytic results for differences in LIs for thalamus volume between HC vs SZ.

```
Random-Effects Model (k = 24; tau^2 estimator: REML)

tau^2 (estimated amount of total heterogeneity): 0.0193 (SE = 0.0149)
tau (square root of estimated tau^2 value):      0.1390
I^2 (total heterogeneity / total variability):   39.39%
H^2 (total variability / sampling variability):  1.65

Test for Heterogeneity:
Q(df = 23) = 42.7581, p-val = 0.0074
```

Model Results:

```
estimate     se    zval    pval    ci.lb   ci.ub
 -0.0204  0.0484  -0.4205  0.6741  -0.1152  0.0745

---
Signif. codes:  0 ‘***’ 0.001 ‘**’ 0.01 ‘*’ 0.05 ‘.’ 0.1 ‘ ’ 1
```

(d) Meta-analytic results for differences in LIs for accumbens volume between HC vs SZ.

```
Random-Effects Model (k = 24; tau^2 estimator: REML)

tau^2 (estimated amount of total heterogeneity): 0.0064 (SE = 0.0097)
tau (square root of estimated tau^2 value):      0.0799
I^2 (total heterogeneity / total variability):   17.75%
H^2 (total variability / sampling variability):  1.22

Test for Heterogeneity:
Q(df = 23) = 25.1473, p-val = 0.3427

Model Results:

estimate     se    zval    pval    ci.lb   ci.ub
  0.0599  0.0401  1.4931  0.1354  -0.0187  0.1385

---
Signif. codes:  0 ‘***’ 0.001 ‘**’ 0.01 ‘*’ 0.05 ‘.’ 0.1 ‘ ’ 1
```

(e) Meta-analytic results for differences in LIs for caudate volume between HC vs SZ.

```
Random-Effects Model (k = 24; tau^2 estimator: REML)

tau^2 (estimated amount of total heterogeneity): 0.0050 (SE = 0.0091)
tau (square root of estimated tau^2 value):      0.0706
I^2 (total heterogeneity / total variability):   14.41%
H^2 (total variability / sampling variability):  1.17

Test for Heterogeneity:
Q(df = 23) = 33.7929, p-val = 0.0682

Model Results:

estimate     se    zval    pval    ci.lb   ci.ub
 -0.1035  0.0390  -2.6504  0.0080  -0.1800  -0.0270  **

---
Signif. codes:  0 ‘***’ 0.001 ‘**’ 0.01 ‘*’ 0.05 ‘.’ 0.1 ‘ ’ 1
```

(f) Meta-analytic results for differences in LIs for putamen volume between HC vs SZ.

```
Random-Effects Model (k = 24; tau^2 estimator: REML)

tau^2 (estimated amount of total heterogeneity): 0.0029 (SE = 0.0082)
tau (square root of estimated tau^2 value):      0.0539
I^2 (total heterogeneity / total variability):   8.91%
```

H^2 (total variability / sampling variability): 1.10

Test for Heterogeneity:
Q(df = 23) = 22.8470, p-val = 0.4697

Model Results:

```
estimate     se    zval    pval    ci.lb   ci.ub
 -0.0396  0.0374  -1.0591  0.2895  -0.1129  0.0337

---
Signif. codes:  0 ‘***’ 0.001 ‘**’ 0.01 ‘*’ 0.05 ‘.’ 0.1 ‘ ’ 1
```

(g) Meta-analytic results for differences in LIs for pallidum volume between HC vs SZ.

Random-Effects Model (k = 24; tau^2 estimator: REML)

tau^2 (estimated amount of total heterogeneity): 0.0109 (SE = 0.0115)
tau (square root of estimated tau^2 value):   0.1042
I^2 (total heterogeneity / total variability):   26.95%
H^2 (total variability / sampling variability): 1.37

Test for Heterogeneity:
Q(df = 23) = 32.0135, p-val = 0.0999

Model Results:

```
estimate     se    zval    pval    ci.lb   ci.ub
  0.3510  0.0432  8.1268  <.0001  0.2663  0.4356  ***

---
Signif. codes:  0 ‘***’ 0.001 ‘**’ 0.01 ‘*’ 0.05 ‘.’ 0.1 ‘ ’ 1
```

(h) Meta-analytic results for differences in LIs for lateral ventricle volume between HC vs SZ.

Random-Effects Model (k = 24; tau^2 estimator: REML)

tau^2 (estimated amount of total heterogeneity): 0.0000 (SE = 0.0069)
tau (square root of estimated tau^2 value):   0.0017
I^2 (total heterogeneity / total variability):   0.01%
H^2 (total variability / sampling variability): 1.00

Test for Heterogeneity:
Q(df = 23) = 19.5571, p-val = 0.6685

Model Results:

```
estimate     se    zval    pval    ci.lb   ci.ub
  0.0098  0.0349  0.2824  0.7776  -0.0585  0.0782

---
Signif. codes:  0 ‘***’ 0.001 ‘**’ 0.01 ‘*’ 0.05 ‘.’ 0.1 ‘ ’ 1
```

Supplementary Table 14: Meta-analytic results for differences in laterality indices (LIs) of each regional volume between healthy controls (HC) vs bipolar disorder (BP).

(a) Meta-analytic results for differences in LIs for hippocampus volume between HC vs BP.

```
Random-Effects Model (k = 10; tau^2 estimator: REML)

tau^2 (estimated amount of total heterogeneity): 0 (SE = 0.0332)
tau (square root of estimated tau^2 value):     0
I^2 (total heterogeneity / total variability):   0.00%
H^2 (total variability / sampling variability):  1.00

Test for Heterogeneity:
Q(df = 9) = 5.6702, p-val = 0.7724

Model Results:

estimate      se    zval    pval    ci.lb   ci.ub
  0.0227  0.0868  0.2616  0.7936  -0.1475  0.1929

---
Signif. codes:  0 ‘***’ 0.001 ‘**’ 0.01 ‘*’ 0.05 ‘.’ 0.1 ‘ ’ 1
```

(b) Meta-analytic results for differences in LIs for amygdala volume between HC vs BP.

```
Random-Effects Model (k = 10; tau^2 estimator: REML)

tau^2 (estimated amount of total heterogeneity): 0 (SE = 0.0329)
tau (square root of estimated tau^2 value):     0
I^2 (total heterogeneity / total variability):   0.00%
H^2 (total variability / sampling variability):  1.00

Test for Heterogeneity:
Q(df = 9) = 3.1122, p-val = 0.9597

Model Results:

estimate      se    zval    pval    ci.lb   ci.ub
  0.0279  0.0863  0.3227  0.7469  -0.1414  0.1971

---
Signif. codes:  0 ‘***’ 0.001 ‘**’ 0.01 ‘*’ 0.05 ‘.’ 0.1 ‘ ’ 1
```

(c) Meta-analytic results for differences in LIs for thalamus volume between HC vs BP.

```
Random-Effects Model (k = 10; tau^2 estimator: REML)

tau^2 (estimated amount of total heterogeneity): 0.0346 (SE = 0.0519)
tau (square root of estimated tau^2 value):     0.1859
I^2 (total heterogeneity / total variability):   31.17%
H^2 (total variability / sampling variability):  1.45

Test for Heterogeneity:
Q(df = 9) = 12.6705, p-val = 0.1781
```

Model Results:

```
estimate     se    zval    pval    ci.lb   ci.ub
 -0.0378  0.1070  -0.3530  0.7241  -0.2475  0.1720

---
Signif. codes:  0 '***' 0.001 '**' 0.01 '*' 0.05 '.' 0.1 ' ' 1
```

(d) Meta-analytic results for differences in LIs for accumbens volume between HC vs BP.

```
andom-Effects Model (k = 10; tau^2 estimator: REML)

tau^2 (estimated amount of total heterogeneity): 0.0319 (SE = 0.0500)
tau (square root of estimated tau^2 value):      0.1786
I^2 (total heterogeneity / total variability):   29.78%
H^2 (total variability / sampling variability):  1.42

Test for Heterogeneity:
Q(df = 9) = 11.6563, p-val = 0.2334

Model Results:

estimate     se    zval    pval    ci.lb   ci.ub
  0.0855  0.1051  0.8137  0.4158  -0.1204  0.2914

---
Signif. codes:  0 '***' 0.001 '**' 0.01 '*' 0.05 '.' 0.1 ' ' 1
```

(e) Meta-analytic results for differences in LIs for caudate volume between HC vs BP.

```
Random-Effects Model (k = 10; tau^2 estimator: REML)

tau^2 (estimated amount of total heterogeneity): 0.0000 (SE = 0.0329)
tau (square root of estimated tau^2 value):      0.0003
I^2 (total heterogeneity / total variability):   0.00%
H^2 (total variability / sampling variability):  1.00

Test for Heterogeneity:
Q(df = 9) = 7.2838, p-val = 0.6076

Model Results:

estimate     se    zval    pval    ci.lb   ci.ub
  0.0942  0.0863  1.0926  0.2746  -0.0748  0.2633

---
Signif. codes:  0 '***' 0.001 '**' 0.01 '*' 0.05 '.' 0.1 ' ' 1
```

(f) Meta-analytic results for differences in LIs for putamen volume between HC vs BP.

```
Random-Effects Model (k = 10; tau^2 estimator: REML)

tau^2 (estimated amount of total heterogeneity): 0.0000 (SE = 0.0321)
tau (square root of estimated tau^2 value):      0.0006
I^2 (total heterogeneity / total variability):   0.00%
```

```
H^2 (total variability / sampling variability):  1.00

Test for Heterogeneity:
Q(df = 9) = 7.9826, p-val = 0.5359

Model Results:

estimate      se     zval    pval    ci.lb    ci.ub
 -0.2147  0.0853  -2.5177  0.0118  -0.3819  -0.0476  *

---
Signif. codes:  0 ‘***’ 0.001 ‘**’ 0.01 ‘*’ 0.05 ‘.’ 0.1 ‘ ’ 1
```

(g) Meta-analytic results for differences in LIs for pallidum volume between HC vs BP.

```
Random-Effects Model (k = 10; tau^2 estimator: REML)

tau^2 (estimated amount of total heterogeneity): 0.0147 (SE = 0.0407)
tau (square root of estimated tau^2 value):      0.1214
I^2 (total heterogeneity / total variability):   16.33%
H^2 (total variability / sampling variability):  1.20

Test for Heterogeneity:
Q(df = 9) = 10.9229, p-val = 0.2810

Model Results:

estimate      se    zval    pval   ci.lb   ci.ub
  0.2138  0.0955  2.2390  0.0252  0.0266  0.4010  *

---
Signif. codes:  0 ‘***’ 0.001 ‘**’ 0.01 ‘*’ 0.05 ‘.’ 0.1 ‘ ’ 1
```

(h) Meta-analytic results for differences in LIs for lateral ventricle volume between HC vs BP

```
Random-Effects Model (k = 10; tau^2 estimator: REML)

tau^2 (estimated amount of total heterogeneity): 0.0065 (SE = 0.0364)
tau (square root of estimated tau^2 value):      0.0807
I^2 (total heterogeneity / total variability):   7.91%
H^2 (total variability / sampling variability):  1.09

Test for Heterogeneity:
Q(df = 9) = 7.5055, p-val = 0.5846

Model Results:

estimate      se     zval    pval    ci.lb   ci.ub
 -0.0346  0.0904  -0.3822  0.7023  -0.2118  0.1427

---
Signif. codes:  0 ‘***’ 0.001 ‘**’ 0.01 ‘*’ 0.05 ‘.’ 0.1 ‘ ’ 1
```

Supplementary Table 15: Meta-analytic results for differences in laterality indices (LIs) of each regional volume between healthy controls (HC) vs major depressive disorder (MDD).

(a) Meta-analytic results for differences in LIs for hippocampus volume between HC vs MDD.

```
Random-Effects Model (k = 14; tau^2 estimator: REML)

tau^2 (estimated amount of total heterogeneity): 0.0000 (SE = 0.0190)
tau (square root of estimated tau^2 value):      0.0009
I^2 (total heterogeneity / total variability):   0.00%
H^2 (total variability / sampling variability):  1.00

Test for Heterogeneity:
Q(df = 13) = 10.4424, p-val = 0.6574

Model Results:

estimate     se     zval    pval    ci.lb   ci.ub
 -0.0265  0.0614  -0.4326  0.6653  -0.1468  0.0937

---
Signif. codes:  0 '***' 0.001 '**' 0.01 '*' 0.05 '.' 0.1 ' ' 1
```

(b) Meta-analytic results for differences in LIs for amygdala volume between HC vs MDD.

```
Random-Effects Model (k = 14; tau^2 estimator: REML)

tau^2 (estimated amount of total heterogeneity): 0.0005 (SE = 0.0191)
tau (square root of estimated tau^2 value):      0.0228
I^2 (total heterogeneity / total variability):   0.97%
H^2 (total variability / sampling variability):  1.01

Test for Heterogeneity:
Q(df = 13) = 14.0188, p-val = 0.3725

Model Results:

estimate     se     zval    pval    ci.lb   ci.ub
 -0.0362  0.0614  -0.5900  0.5552  -0.1566  0.0841

---
Signif. codes:  0 '***' 0.001 '**' 0.01 '*' 0.05 '.' 0.1 ' ' 1
```

(c) Meta-analytic results for differences in LIs for thalamus volume between HC vs MDD.

```
Random-Effects Model (k = 14; tau^2 estimator: REML)

tau^2 (estimated amount of total heterogeneity): 0.0649 (SE = 0.0478)
tau (square root of estimated tau^2 value):      0.2548
I^2 (total heterogeneity / total variability):   55.05%
H^2 (total variability / sampling variability):  2.22

Test for Heterogeneity:
Q(df = 13) = 29.9185, p-val = 0.0048
```

Model Results:

```
estimate     se     zval    pval    ci.lb   ci.ub
 -0.0184  0.0944  -0.1948  0.8456  -0.2034  0.1666

---
Signif. codes:  0 '***' 0.001 '**' 0.01 '*' 0.05 '.' 0.1 ' ' 1
```

(d) Meta-analytic results for differences in LIs for accumbens volume between HC vs MDD.

```
Random-Effects Model (k = 14; tau^2 estimator: REML)

tau^2 (estimated amount of total heterogeneity): 0.0234 (SE = 0.0299)
tau (square root of estimated tau^2 value):      0.1530
I^2 (total heterogeneity / total variability):   30.48%
H^2 (total variability / sampling variability):  1.44

Test for Heterogeneity:
Q(df = 13) = 21.0377, p-val = 0.0722

Model Results:

estimate     se     zval    pval    ci.lb   ci.ub
 -0.0172  0.0754  -0.2274  0.8201  -0.1650  0.1307

---
Signif. codes:  0 '***' 0.001 '**' 0.01 '*' 0.05 '.' 0.1 ' ' 1
```

(e) Meta-analytic results for differences in LIs for caudate volume between HC vs MDD.

```
Random-Effects Model (k = 14; tau^2 estimator: REML)

tau^2 (estimated amount of total heterogeneity): 0.0000 (SE = 0.0187)
tau (square root of estimated tau^2 value):      0.0015
I^2 (total heterogeneity / total variability):   0.00%
H^2 (total variability / sampling variability):  1.00

Test for Heterogeneity:
Q(df = 13) = 11.8025, p-val = 0.5439

Model Results:

estimate     se     zval    pval    ci.lb   ci.ub
 -0.0404  0.0608  -0.6651  0.5060  -0.1597  0.0788

---
Signif. codes:  0 '***' 0.001 '**' 0.01 '*' 0.05 '.' 0.1 ' ' 1
```

(f) Meta-analytic results for differences in LIs for putamen volume between HC vs MDD.

```
Random-Effects Model (k = 14; tau^2 estimator: REML)

tau^2 (estimated amount of total heterogeneity): 0.0000 (SE = 0.0187)
tau (square root of estimated tau^2 value):      0.0021
I^2 (total heterogeneity / total variability):   0.01%
```

H^2 (total variability / sampling variability): 1.00

Test for Heterogeneity:
Q(df = 13) = 10.3804, p-val = 0.6626

Model Results:

estimate	se	zval	pval	ci.lb	ci.ub
-0.0443	0.0609	-0.7265	0.4675	-0.1637	0.0752

Signif. codes: 0 '***' 0.001 '**' 0.01 '*' 0.05 '.' 0.1 ' ' 1

(g) Meta-analytic results for differences in LIs for pallidum volume between HC vs MDD.

Random-Effects Model (k = 14; tau^2 estimator: REML)

tau^2 (estimated amount of total heterogeneity): 0.0157 (SE = 0.0265)
tau (square root of estimated tau^2 value):   0.1253
I^2 (total heterogeneity / total variability):   22.64%
H^2 (total variability / sampling variability): 1.29

Test for Heterogeneity:
Q(df = 13) = 16.9510, p-val = 0.2016

Model Results:

estimate	se	zval	pval	ci.lb	ci.ub
0.0701	0.0714	0.9823	0.3260	-0.0698	0.2100

Signif. codes: 0 '***' 0.001 '**' 0.01 '*' 0.05 '.' 0.1 ' ' 1

(h) Meta-analytic results for differences in LIs for lateral ventricle volume between HC vs MDD.

Random-Effects Model (k = 14; tau^2 estimator: REML)

tau^2 (estimated amount of total heterogeneity): 0 (SE = 0.0191)
tau (square root of estimated tau^2 value):   0
I^2 (total heterogeneity / total variability):   0.00%
H^2 (total variability / sampling variability): 1.00

Test for Heterogeneity:
Q(df = 13) = 5.5535, p-val = 0.9609

Model Results:

estimate	se	zval	pval	ci.lb	ci.ub
-0.0025	0.0615	-0.0404	0.9678	-0.1230	0.1180

Signif. codes: 0 '***' 0.001 '**' 0.01 '*' 0.05 '.' 0.1 ' ' 1

Supplementary Table 16: Meta-analytic results for differences in laterality indices (LIs) of each regional volume between healthy controls (HC) vs autism spectrum disorder (ASD).

(a) Meta-analytic results for differences in LIs for hippocampus volume between HC vs ASD.

```
Random-Effects Model (k = 7; tau^2 estimator: REML)

tau^2 (estimated amount of total heterogeneity): 0.0491 (SE = 0.0622)
tau (square root of estimated tau^2 value):     0.2215
I^2 (total heterogeneity / total variability):  46.77%
H^2 (total variability / sampling variability):  1.88

Test for Heterogeneity:
Q(df = 6) = 11.1843, p-val = 0.0828

Model Results:

estimate      se    zval    pval    ci.lb   ci.ub
  0.1348  0.1260  1.0692  0.2850  -0.1123  0.3818

---
Signif. codes:  0 '***' 0.001 '**' 0.01 '*' 0.05 '.' 0.1 ' ' 1
```

(b) Meta-analytic results for differences in LIs for amygdala volume between HC vs ASD.

```
Random-Effects Model (k = 7; tau^2 estimator: REML)

tau^2 (estimated amount of total heterogeneity): 0 (SE = 0.0280)
tau (square root of estimated tau^2 value):     0
I^2 (total heterogeneity / total variability):  0.00%
H^2 (total variability / sampling variability):  1.00

Test for Heterogeneity:
Q(df = 6) = 2.7456, p-val = 0.8400

Model Results:

estimate      se     zval    pval    ci.lb   ci.ub
 -0.0013  0.0863  -0.0148  0.9882  -0.1704  0.1679

---
Signif. codes:  0 '***' 0.001 '**' 0.01 '*' 0.05 '.' 0.1 ' ' 1
```

(c) Meta-analytic results for differences in LIs for thalamus volume between HC vs ASD.

```
Random-Effects Model (k = 7; tau^2 estimator: REML)

tau^2 (estimated amount of total heterogeneity): 0.0226 (SE = 0.0445)
tau (square root of estimated tau^2 value):     0.1503
I^2 (total heterogeneity / total variability):  28.85%
H^2 (total variability / sampling variability):  1.41

Test for Heterogeneity:
Q(df = 6) = 8.6345, p-val = 0.1952
```

Model Results:

```
estimate     se    zval    pval    ci.lb   ci.ub
  0.0277  0.1074  0.2580  0.7964  -0.1828  0.2382

---
Signif. codes:  0 ‘***’ 0.001 ‘**’ 0.01 ‘*’ 0.05 ‘.’ 0.1 ‘ ’ 1
```

(d) Meta-analytic results for differences in LIs for accumbens volume between HC vs ASD.

```
Random-Effects Model (k = 7; tau^2 estimator: REML)

tau^2 (estimated amount of total heterogeneity): 0 (SE = 0.0281)
tau (square root of estimated tau^2 value):      0
I^2 (total heterogeneity / total variability):   0.00%
H^2 (total variability / sampling variability):  1.00

Test for Heterogeneity:
Q(df = 6) = 5.1766, p-val = 0.5214

Model Results:

estimate     se    zval    pval    ci.lb   ci.ub
  0.0972  0.0865  1.1239  0.2610  -0.0723  0.2667

---
Signif. codes:  0 ‘***’ 0.001 ‘**’ 0.01 ‘*’ 0.05 ‘.’ 0.1 ‘ ’ 1
```

(e) Meta-analytic results for differences in LIs for caudate volume between HC vs ASD.

```
Random-Effects Model (k = 7; tau^2 estimator: REML)

tau^2 (estimated amount of total heterogeneity): 0.0306 (SE = 0.0502)
tau (square root of estimated tau^2 value):      0.1750
I^2 (total heterogeneity / total variability):   35.30%
H^2 (total variability / sampling variability):  1.55

Test for Heterogeneity:
Q(df = 6) = 9.4740, p-val = 0.1486

Model Results:

estimate     se     zval    pval    ci.lb   ci.ub
 -0.0919  0.1138  -0.8075  0.4194  -0.3149  0.1311

---
Signif. codes:  0 ‘***’ 0.001 ‘**’ 0.01 ‘*’ 0.05 ‘.’ 0.1 ‘ ’ 1
```

(f) Meta-analytic results for differences in LIs for putamen volume between HC vs ASD.

```
Random-Effects Model (k = 7; tau^2 estimator: REML)

tau^2 (estimated amount of total heterogeneity): 0.0000 (SE = 0.0284)
tau (square root of estimated tau^2 value):      0.0015
I^2 (total heterogeneity / total variability):   0.00%
```

H^2 (total variability / sampling variability): 1.00

Test for Heterogeneity:
Q(df = 6) = 6.4652, p-val = 0.3731

Model Results:

estimate	se	zval	pval	ci.lb	ci.ub
0.0409	0.0869	0.4713	0.6374	-0.1293	0.2112

Signif. codes: 0 '***' 0.001 '**' 0.01 '*' 0.05 '.' 0.1 ' ' 1

(g) Meta-analytic results for differences in LIs for pallidum volume between HC vs ASD.

Random-Effects Model (k = 7; tau^2 estimator: REML)

tau^2 (estimated amount of total heterogeneity): 0.0170 (SE = 0.0404)
tau (square root of estimated tau^2 value):   0.1304
I^2 (total heterogeneity / total variability):   23.46%
H^2 (total variability / sampling variability): 1.31

Test for Heterogeneity:
Q(df = 6) = 7.1136, p-val = 0.3105

Model Results:

estimate	se	zval	pval	ci.lb	ci.ub
0.1232	0.1027	1.2000	0.2301	-0.0780	0.3244

Signif. codes: 0 '***' 0.001 '**' 0.01 '*' 0.05 '.' 0.1 ' ' 1

(h) Meta-analytic results for differences in LIs for lateral ventricle volume between HC vs ASD.

Random-Effects Model (k = 7; tau^2 estimator: REML)

tau^2 (estimated amount of total heterogeneity): 0.0000 (SE = 0.0285)
tau (square root of estimated tau^2 value):   0.0019
I^2 (total heterogeneity / total variability):   0.01%
H^2 (total variability / sampling variability): 1.00

Test for Heterogeneity:
Q(df = 6) = 7.8595, p-val = 0.2486

Model Results:

estimate	se	zval	pval	ci.lb	ci.ub
0.0940	0.0871	1.0798	0.2802	-0.0766	0.2647

Signif. codes: 0 '***' 0.001 '**' 0.01 '*' 0.05 '.' 0.1 ' ' 1

Supplementary Table 17: Association between clustering results and mean z-scores of each regional volume.

Cluster	N	Lhippo	Rhippo	Lamyg	Ramyg	Lthal	Rthal	Laccumb	Raccumb	Lcaud	Rcaud	Lput	Rput	Lpal	Rpal	LLatVent	RLatVent
A	110	-2.76	-2.53	-1.16	-0.78	-1.47	-1.28	-1.41	-1.03	0.50	0.23	-0.21	-0.42	0.36	-0.43	5.23	5.03
B	566	-1.74	-1.69	-0.89	-0.85	-1.05	-1.06	-0.78	-0.86	-0.13	-0.03	-0.43	-0.50	0.18	-0.25	1.69	1.64
C	679	-0.97	-0.95	-0.26	-0.22	-0.18	-0.05	-0.01	0.01	1.05	1.07	1.07	1.14	1.37	1.15	0.92	0.89
D	796	-0.64	-0.70	-0.67	-0.70	-0.41	-0.60	-0.77	-0.84	-0.90	-0.84	-1.05	-1.06	-0.77	-0.87	-0.05	-0.03
E	1,365	-0.38	-0.36	-0.24	-0.19	-0.48	-0.40	0.00	-0.02	0.04	0.06	0.09	0.04	0.25	0.02	0.14	0.12
F	985	0.62	0.61	0.65	0.67	0.48	0.60	0.79	0.76	0.72	0.70	1.01	1.11	0.81	0.84	-0.31	-0.29
G	1,101	0.56	0.57	0.31	0.33	0.56	0.41	-0.14	-0.14	-0.31	-0.36	-0.24	-0.13	-0.38	-0.22	-0.41	-0.42
H	1	-3.44	-4.32	-1.13	-1.17	-6.96	-7.40	-4.00	-5.10	-7.10	-7.31	-2.56	-2.99	-3.66	-3.52	-3.27	-2.66
I	1	-5.67	-6.62	0.88	1.67	-9.62	-7.56	2.72	2.42	-6.39	-6.51	-1.52	0.33	-1.69	-2.02	-5.95	-3.90

Abbreviations: Lhippo, left hippocampus; Rhippo, right hippocampus; Lamyg, left amygdala; Ramyg, right amygdala; Lthal, left thalamus; Rthal, right thalamus; Laccumb, left accumbens; Raccumb, right accumbens; Lcaud, left caudate; Rcaud, right caudate; Lput, left putamen; Rput, right putamen; Lpal, left pallidum; Rpal, right pallidum; LLatVent, left lateral ventricle; RLatVent, right lateral ventricle.

Supplementary Table 18: Subject numbers based on diagnostic groups in each cluster.

Cluster	Diagnositic group					Total
	HC	SZ	BP	MDD	ASD	
A	13	69	9	16	3	110
B	163	273	28	87	15	566
C	177	389	34	64	15	679
D	464	142	38	116	36	796
E	835	309	57	118	46	1,365
F	619	203	33	97	33	985
G	806	115	36	99	45	1,101
H	1	0	0	0	0	1
I	0	0	0	1	0	1
Total	3,078	1,500	235	598	193	5,604

Abbreviations: HC, healthy control; SZ, schizophrenia; BP, bipolar disorder; MDD, major depressive disorder;ASD, autism spectrum disorder.

Supplementary Table 19: Subject numbers based on diagnostic groups in each cluster in the cognitive function analysis.

Cluster	Diagnositic group					Total
	HC	SZ	BP	MDD	ASD	
A	1	11	0	0	1	13
B	54	43	0	1	1	99
C	59	52	0	2	3	116
D	143	35	1	3	5	187
E	250	50	0	3	5	308
F	197	36	0	1	4	238
G	233	17	0	0	7	257
Total	937	244	1	10	26	1,218

Abbreviations: HC, healthy control; SZ, schizophrenia; BP, bipolar disorder; MDD, major depressive disorder; ASD, autism spectrum disorder.

Supplementary Table 20: Subject numbers based on diagnostic groups in each cluster in the social function analysis

Cluster	Diagnositic group					Total
	HC	SZ	BP	MDD	ASD	
A	1	2	0	0	1	4
B	29	24	0	1	0	54
C	27	28	0	1	0	56
D	67	16	0	2	2	87
E	127	26	0	1	0	154
F	107	23	0	0	0	130
G	119	12	0	0	0	131
Total	477	131	0	5	3	616

Abbreviations: HC, healthy control; SZ, schizophrenia; BP, bipolar disorder; MDD, major depressive disorder; ASD, autism spectrum disorder.
